# Nitrene transfer from a sterically confined copper nitrenoid dipyrrin complex†

**DOI:** 10.1039/d3sc03641c

**Published:** 2023-09-26

**Authors:** Kurtis M. Carsch, Sasha C. North, Ida M. DiMucci, Andrei Iliescu, Petra Vojáčková, Thomas Khazanov, Shao-Liang Zheng, Thomas R. Cundari, Kyle M. Lancaster, Theodore A. Betley

**Affiliations:** a Department of Chemistry and Chemical Biology, Harvard University Cambridge MA 02138 USA betley@chemistry.harvard.edu; b Center for Advanced Scientific Computing and Modeling (CASCaM), Department of Chemistry, University of North Texas, Denton TX 76203 USA; c Department of Chemistry and Chemical Biology, Baker Laboratory, Cornell University Ithaca New York 14853 USA

## Abstract

Despite the myriad Cu-catalyzed nitrene transfer methodologies to form new C–N bonds (*e.g.*, amination, aziridination), the critical reaction intermediates have largely eluded direct characterization due to their inherent reactivity. Herein, we report the synthesis of dipyrrin-supported Cu nitrenoid adducts, investigate their spectroscopic features, and probe their nitrene transfer chemistry through detailed mechanistic analyses. Treatment of the dipyrrin Cu^I^ complexes with substituted organoazides affords terminally ligated organoazide adducts with minimal activation of the azide unit as evidenced by vibrational spectroscopy and single crystal X-ray diffraction. The Cu nitrenoid, with an electronic structure most consistent with a triplet nitrene adduct of Cu^I^, is accessed following geometric rearrangement of the azide adduct from κ^1^-N terminal ligation to κ^1^-N internal ligation with subsequent expulsion of N_2_. For perfluorinated arylazides, stoichiometric and catalytic C–H amination and aziridination was observed. Mechanistic analysis employing substrate competition reveals an enthalpically-controlled, electrophilic nitrene transfer for primary and secondary C–H bonds. Kinetic analyses for catalytic amination using tetrahydrofuran as a model substrate reveal pseudo-first order kinetics under relevant amination conditions with a first-order dependence on both Cu and organoazide. Activation parameters determined from Eyring analysis (Δ*H*^‡^ = 9.2(2) kcal mol^−1^, Δ*S*^‡^ = −42(2) cal mol^−1^ K^−1^, Δ*G*^‡^_298K_ = 21.7(2) kcal mol^−1^) and parallel kinetic isotope effect measurements (1.10(2)) are consistent with rate-limiting Cu nitrenoid formation, followed by a proposed stepwise hydrogen-atom abstraction and rapid radical recombination to furnish the resulting C–N bond. The proposed mechanism and experimental analysis are further corroborated by density functional theory calculations. Multiconfigurational calculations provide insight into the electronic structure of the catalytically relevant Cu nitrene intermediates. The findings presented herein will assist in the development of future methodology for Cu-mediated C–N bond forming catalysis.

## Abstract 2

A dipyrrin-supported copper complex mediates C–H bond amination and aziridination of exogenous substrates using electron-deficient arylazides, proceeding through copper-nitrene adducts.

## Graphical abstract

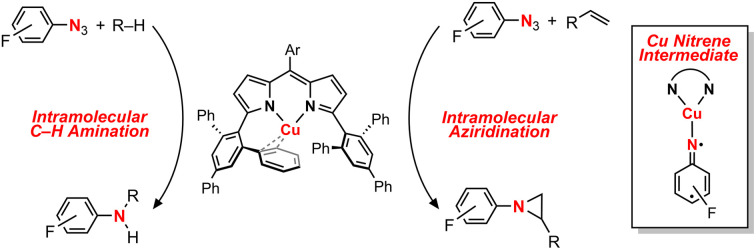

## 1 Introduction

**Fig. 1:**
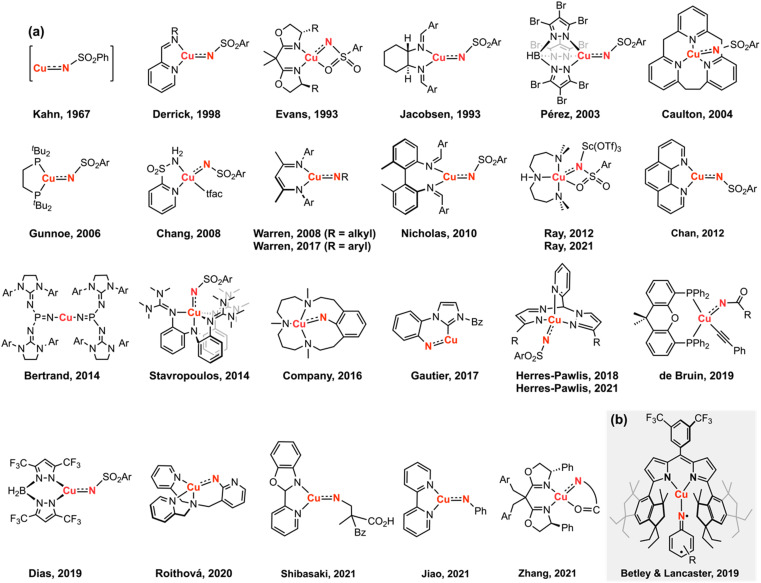
(a) Invoked Cu nitrenoid intermediates for H-atom abstraction, C–H amination, nitrene transfer, and aziridination transformations. (b) The isolation of a Cu nitrenoid represents the first structurally validated and spectroscopically authenticated example competent for nitrene transfer. Charges are omitted for clarity in which all presented Cu complexes are formally Cu(iii).

Copper-catalyzed amination and aziridination are powerful methodologies for C–H activation with applications in the elaboration of simple chemical feedstocks and the construction of various nitrogen-containing natural products.^1,2^ Furthermore, the earth-abundance and low toxicity of Cu relative to precious metals render its employment in stereospecific catalysis attractive from an environmental perspective.^3^ New methods for direct C–H functionalization *in lieu* of functional group exchange provide the potential for achieving high atom economy and also establish new opportunities for late-stage diversification of complex molecules.^4^ Notable Cu-mediated C–N bond construction reactions include C–H amination of inert alkanes,^5^ heterocycle ring-expansion,^6^ alkyne elaboration to isothiazoles,^7^ and asymmetric aziridination.^8–10^ The bulk of these transformations have been developed for the *N*-tosylnitrene transfer reagent [(phenylsulfonyl)imino]phenyliodinane (PhINTs), based on the ease of precursor synthesis and the capacity for detosylation to deprotect amines or aziridines.^11^ In addition, protected amines and aroyl azides have been incorporated into aziridination^12^ and amidation^13^ reactions. Cu nitrenoid (Cu–NR) species are commonly invoked as the reactive intermediate for C–H bond activation and alkene aziridination (Fig. 1);^5,10,14–36^ however, the fleeting nature of these highly reactive intermediates has precluded their spectroscopic observation and direct analysis of their reactivity profiles. In the absence of structural authentication and rigorous characterization, the intermediacy of a copper nitrenoid can be inferred from computational support^37,38^ and through kinetic analysis of enantioselective aziridination.^39^ Reaction optimization is further complicated by the potential redox non-innocence of the nitrene fragment and the capacity of Cu to reside in a variety of spin states, potentially giving rise to different reactivity profiles based on the participation of the nitrenoid in the frontier orbital manifold. Consequently, understanding the electronic structure of the Cu nitrenoid intermediate and the distribution of electrons across the Cu–N bond is paramount to understanding its reactivity.

We previously reported structural characterization of the first *bona fide* copper nitrenoid complex, derived from treatment of a mononuclear Cu(N_2_) complex with electron-rich arylazides.^40^ The terminology nitrenoid refers to a general monosubstituted nitrogen motif with no specific claims regarding the valency of the nitrogen center. By contrast, imido (NR^2−^), imidyl (^2^NR^1−^), and nitrene (^3^NR or ^1^NR) denote specific claims regarding the valency of the nitrene; consequently, prior to acquisition of rigorous spectroscopic data, we elect to employ the descriptor nitrenoid. Due to the heightened reactivity of the resultant Cu nitrene, all syntheses and manipulations were conducted in passivated glassware using solvents with strong C–H bonds and without allowing reaction mixtures to exceed room temperature. Specifically, the Cu nitrene species were found to degrade *via* azoarene formation when solutions were stored at 40 °C or left standing in C_6_D_6_ at room temperature over multiple days. The structural metrics of the nitrene adduct as determined by single-crystal X-ray diffraction revealed dearomatization of the nitrene aryl substituent with bond lengths akin to isolated Cu diketimide complexes obtained by reductive coupling with C(sp^3^)-hybridization at the *para*-aryl carbon reported by Warren and coworkers^25^ and by us.^40^ Multinuclear X-ray absorption spectroscopy, including Cu K/L_2,3_-edge^41^ and N K-edge XANES^42^ with further corroboration by multiconfigurational calculations, revealed the most appropriate electron description as a cuprous triplet nitrene adduct (*i.e.*, Cu^I^(^3^NAr)), *in lieu* of a higher valent cupric imidyl redox isomer (*i.e.*, Cu^II^(^2^NAr)), or cupryl imido complex (*i.e.*, Cu^III^(NAr)).^43^ While the hydrindacene-substituted dipyrrin allowed for isolation and characterization of the Cu nitrene, modification of the dipyrrin with a less sterically-occluded flanking group allowed for the stoichiometric nitrene transfer reactivity to be rendered catalytic.

The employment of electron-deficient arylazides afforded analogous paramagnetically-shifted ^1^H NMR resonances to those observed for isolated Cu nitrenoids (^EMind^L)Cu[N(C_6_H_4_O^*t*^Bu)] (3-O*^t^*Bu) and (^EMind^L)Cu[N(C_6_H_4_^*t*^Bu)] (3-*^t^*Bu), suggesting similar bond connectivity, and afforded productive intermolecular C–H amination and aziridination of various substrates.

We herein describe the reactivity profile of a sterically unencumbered Cu complex competent for C–H amination and aziridination using perfluorinated electron-deficient arylazides, with catalysis exhibited for select substrates including alkenes and C–H bonds adjacent to heteroatoms and arenes. Mechanistic experimental and computational studies are consistent with a stepwise H-atom abstraction and radical recombination with a rate-limiting step assigned as Cu nitrenoid formation, which is in contrast with other dipyrrin amination catalysts which exhibit rate-limiting substrate activation through hydrogen-atom abstraction.

## 2 Results and discussion

### 2.1. Copper nitrene construction

To isolate an authentic mononuclear Cu nitrenoid species, we rationalized an anionic ligand scaffold with sufficient steric protection would provide adequate lifetimes for spectroscopic characterization. Accordingly, we selected the dipyrrin platform, noting the capacity of the ligand platform to support low-coordinate mid/late 3d metal nitrenoid complexes^44–49^ as well as the acute bite angle with respect to the flanking substituents.^50^ In particular, we considered EMind (1,1,7,7-tetraethyl-1,2,3,5,6,7-hexahydro-3,3,5,5-tetramethyl-*s*-indacene)^51^ due to the ease of synthesis on multigram scales and opportunities to modify the steric profile of the substituents as deemed necessary. The peralkylated hydrindacene EMind was selected for its ample steric protection, noting the spatial rigidity from of the methyl substituents would mitigate dealkylation or C–H activation pathways commonly observed with similarly sterically encumbering supermesityl (1,3,5-tri(*tert*-butyl)benzene).^52^ Moreover, the employment of the EMind flanking unit would prevent intramolecular metal-arene interactions, which have been observed for trityl^53^ and quadrophenyl^54^ steric protection.

**Scheme 1:**
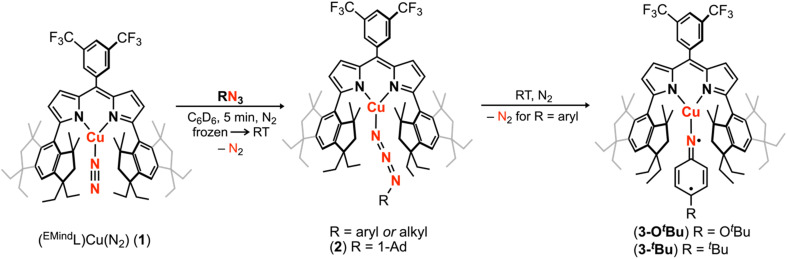
Cu nitrenoid synthesis from (^EMind^L)Cu(N_2_) (1) through intermittency of an observable, diamagnetic azide adduct (^EMind^L)Cu(N_3_R), isolable for (^EMind^L)Cu(N_3_Ad) (2). For arylazides, extrusion of N_2_ affords the corresponding Cu nitrenoid product (^EMind^L)Cu[N(C_6_H_4_O^*t*^Bu)] (3-O*^t^*Bu) or (^EMind^L)Cu[N(C_6_H_4_^*t*^Bu)] (3-*^t^*Bu).^40^

Treatment of (^EMind^L)H with mesitylcopper in anhydrous benzene at elevated temperatures afforded (^EMind^L)Cu(C_6_H_6_) with clean conversion to (^EMind^L)Cu(N_2_) (1) upon removal of excess solvent and recrystallization from a concentrated pentane solution under N_2_ at −35 °C.^40,55^ The N_2_ adduct in 1 represents one of the least activated isolable metal-dinitrogen complexes (*ν*_N_2__ = 2242 cm^−1^), reflecting an energetic mismatch between the Cu^I^ ion and N_2_ π*-orbitals due to poor energetic and spatial overlap and, therefore, minimal π-backbonding. Given the minimal activation of N_2_, we rationalized facile ligand substitution of N_2_ for organoazides would be feasible. Treatment of 1 in hexanes with substituted organoazide substrates is accompanied by rapid effervescence and a color change from carrot-orange to red-orange. Analysis by multinuclear NMR spectroscopy (^1^H/^13^C{^1^H}/^19^F) revealed quantitative consumption of 1 in the presence of arylazides to form a new diamagnetic species, with gradual consumption of this intermediate over several hours to yield the corresponding copper nitrenoids (^EMind^L)Cu[N(C_6_H_4_O^*t*^Bu)] (3-O*^t^*Bu) and (^EMind^L)Cu[N(C_6_H_4_^*t*^Bu)] (3-*^t^*Bu) as identified by single crystal X-ray diffraction (Scheme 1).^40^ The intermediate prior to copper nitrenoid formation is ascribed to an azide adduct (^EMind^L)Cu(N_3_Ar) based on notable perturbations in the azide stretching frequencies. In particular, treatment of 1 with (4-^*t*^Bu)C_6_H_6_N_3_ results in a significant changes by infrared spectroscopy (*ν*_N_3_Ar_ = 2121, 2087 cm^−1^) compared to the free arylazide (*ν*_N_3_Ar_ = 2127, 2088 cm^−1^) (Fig. S76†). The employment of the ^15^N_α_ isotopologue (4-^*t*^Bu)C_6_H_6_^15^NNN corroborated the identity of these resonances (*ν*_NN^15^NAr_ = 2113 cm^−1^; free *ν*_NN^15^NAr_ = 2110, 2066 cm^−1^) (Fig. S12†). Although the thermal instability of (^EMind^L)Cu(N_3_Ar) to yield the subsequent Cu nitrenoid precludes structural characterization and identification of an internal (N_α_-bound) or terminal (N_γ_-bound) motif, employment of the sterically encumbered alkyl azide 1-azidoadamantane (N_3_Ad) facilitated isolation of the thermally and photolytically robust (^EMind^L)Cu(N_3_Ad) (2) with κ^1^-ligation to the terminal nitrogen atom (N_γ_) (Fig. 3a). Employing alkyl azides without quaternary substitution on the carbon adjacent to the nitrogen resulted in rapid formation of a diamagnetic species, consistent with α-elimination to yield the corresponding imine adduct, which has been observed elsewhere for putative late transition metal nitrenoid complexes.^44,45,56^ Minimal elongation is observed within the azide unit of 2 (N_γ_–N_β_ = 1.129(8) Å, 1.231(9) Å), in accord with the observed infrared spectroscopy data (*ν*_N_3_Ad_ = 2134, 2107 cm^−1^; free *ν*_N_3_Ad_ = 2140, 2088 cm^−1^) (Fig. S14 and S15†). By analogy, the observed diamagnetic species prior to formation of the Cu nitrenoid intermediate is attributed to a terminal N_γ_-ligated azide adduct, which undergoes gradual rearrangement to an internal N_α_-ligation with subsequent N_2_ expulsion to yield the resulting Cu nitrene.

### 2.2. X-ray absorption spectroscopy

**Fig. 2:**
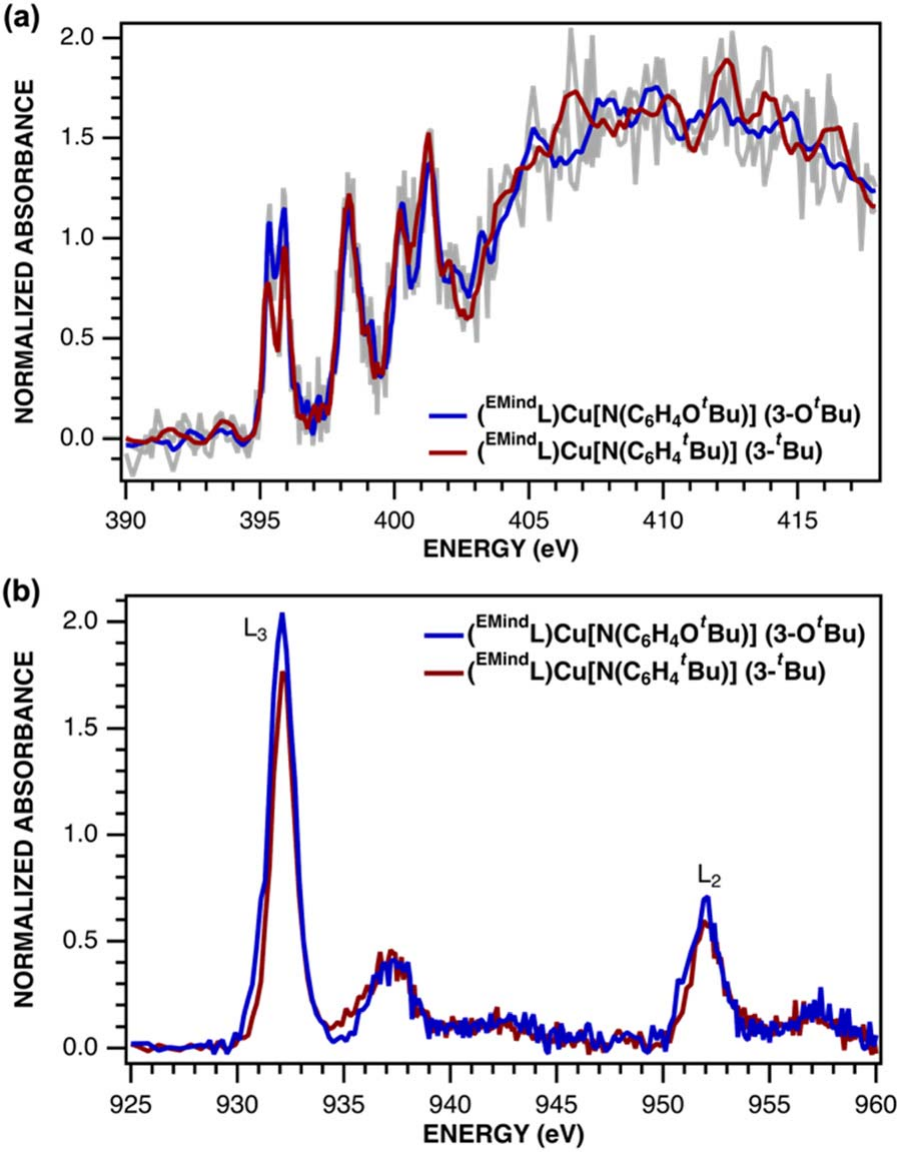
(a) N K-edge and (b) Cu L_2,3_-edge XANES spectra for (^EMind^L)Cu[N(C_6_H_4_O^*t*^Bu)] (3-O*^t^*Bu, blue) and (^EMind^L)Cu[N(C_6_H_4_^*t*^Bu)] (3-*^t^*Bu, red).

The electronic structures of triplet copper nitrenoid complexes 3-O*^t^*Bu and 3-*^t^*Bu were ascertained by multinuclear X-ray absorption (XAS) spectroscopy (Fig. 2). The N K-edge XAS data reveal two low-energy pre-edge features at 395.31 eV and 395.91 eV in the case of 3-*^t^*Bu, and at 395.36 eV and 395.91 eV in the case of 3-O*^t^*Bu (Fig. 2A). The splitting of this pre-edge feature is consistent with two holes localized to the N of the aryl nitrenoid motif,^42^ indicating a cuprous triplet nitrene adduct Cu^I^(^3^NAr) as the best description of the ground states of both 3-*^t^*Bu and 3-O*^t^*Bu. Our assignment is further corroborated by Cu L_2,3_-edge XAS (Fig. 2B). The L_3_- and L_2_-edge main lines occur at approximately 931.5 eV and 951.3 eV, respectively, for both 3-*^t^*Bu and 3-O*^t^*Bu. Experimental estimations of the Cu 3d character in the acceptor orbitals derived from integration of the L_3_- and L_2_-edge main lines (Fig. S85 and S86†) as previously described^43^ place the average 3d character per hole at 21% for 3-*^t^*Bu and 25% for 3-O*^t^*Bu. The attenuation of Cu 3d character in the acceptor orbitals is consistent with majority hole character contained in N-localized orbitals and a physical oxidation state of Cu^I^. Similar integration of the pre-edge features arising from the nitrene in the N K-edge spectrum is consistent with more N 2p character in the acceptor orbitals in the case of 3-O*^t^*Bu, as well (Fig. S83 and S84†). Taken together, the N K-edge and Cu L_2,3_-edge XAS suggest that the influence of the *tert*-butoxy oxygen heteroatom results in more spin density localized to the Cu–N unit, but that both complexes are best described as Cu^I^(^3^NAr) adducts.

**Fig. 3:**
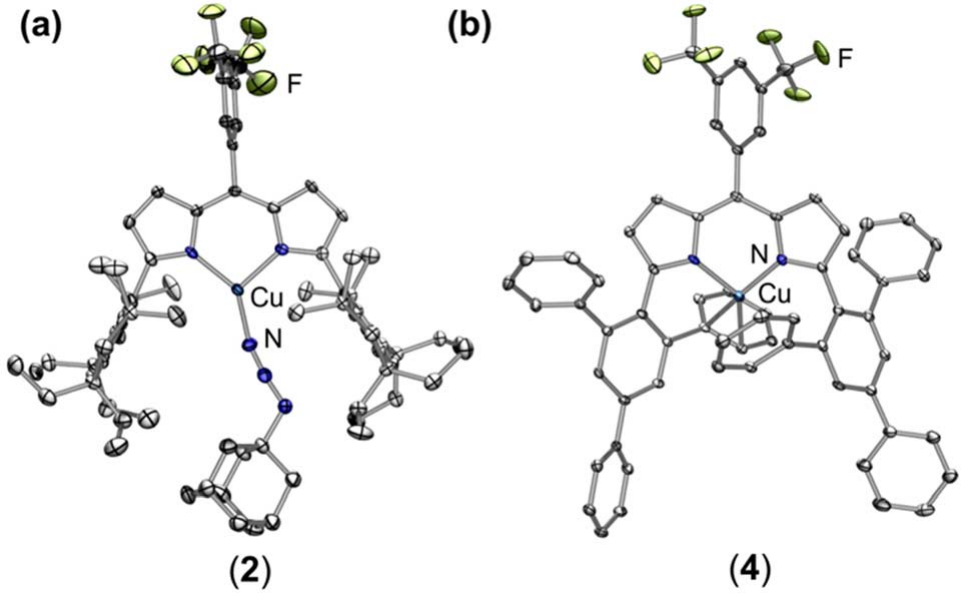
Solid state molecular structures of (a) (^EMind^L)Cu(N_3_Ad) (2) and (b) (^ArF^L)Cu (4) at 50% ellipsoid probability. Color scheme: Cu (cobalt blue), F (yellow-green), N (blue). Solvent molecules, structural disorder, and hydrogen atoms are omitted for clarity.

### 2.3. Intermolecular amination and aziridination

**Scheme 2:**
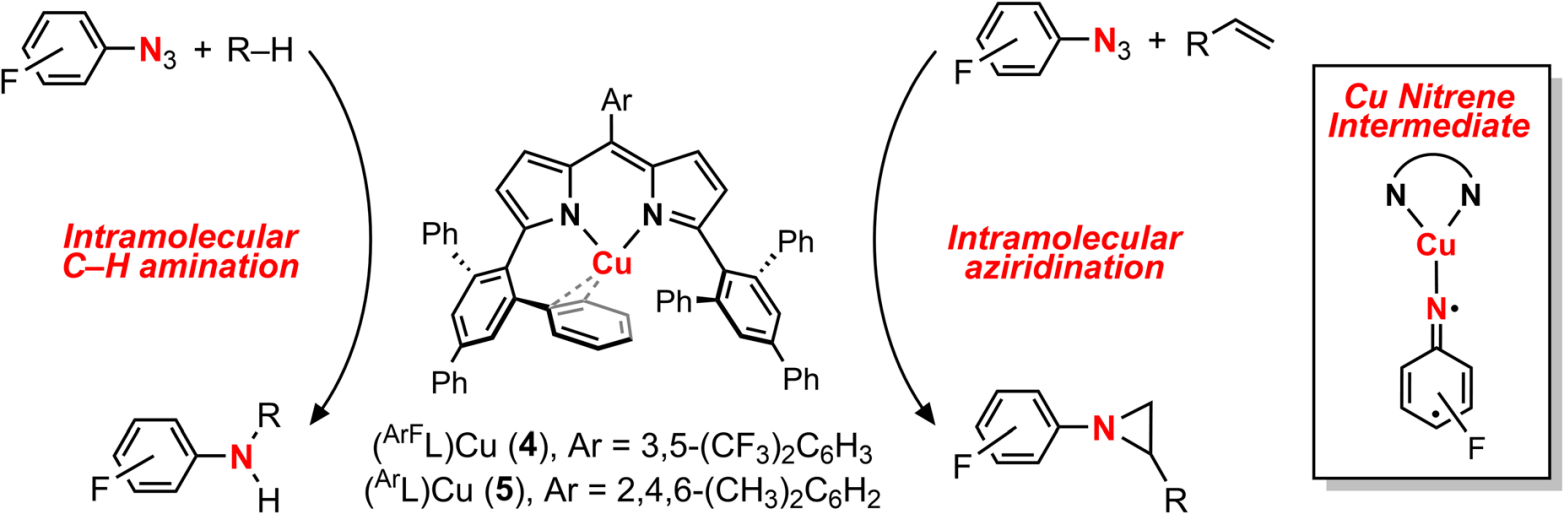
Nitrene transfer reactivity modes from (^ArF^L)Cu (4) and (^Ar^L)Cu (5)^54^ with Cu nitrene intermediate.

Whereas 3-O*^t^*Bu and 3-*^t^*Bu were competent for aromatization of 1,4-cyclohexadiene (BDE_C–H_ = 76 kcal mol^−1^)^57^ to yield benzene and the corresponding aniline adduct (^EMind^L)Cu(H_2_NAr) as identified by independent synthesis, C–H amination was not observed from the Cu nitrene adducts for more inert substrates such as toluene or cyclohexane. This absence of C–H amination is attributed to steric preclusion of substrates from the EMind substituents and the weak N–H bond of the subsequent Cu anilido intermediate. Thus, we targeted electron-deficient nitrene sources for greater N–H bond strengths to enhance the efficacy of nitrene transfer. Treatment of 1 with stoichiometric pentafluorophenyl azide (C_6_F_5_N_3_) in thawing toluene afforded the corresponding benzylic aminated species, albeit in diminished yield (10%) with the predominant organic product identified C_6_F_5_NH_2_ (55%) following demetallation through acidification and quantification by ^19^F NMR spectroscopy. Inspection of the crude reaction mixture by EPR spectroscopy reveals formation of independently isolated cupric anilido product (^EMind^L)Cu(NH(C_6_F_5_)), resulting in poor mass balance.^40^ We proposed the limited nitrene transfer reactivity was attributable to the steric pressure about the Cu center. Thus, we selected the more sterically exposed variant (^ArF^L)Cu (4) with rotationally flexible 2,4,6-triphenyl(aryl) flanking substituents while maintaining the dipyrrin methine 3,5-bis-trifluoromethyl aryl substituent to match the electronic nature of 1 and provide a ^19^F NMR handle to assess Cu speciation during nitrene transfer reactions. The synthesis of the dipyrrin platform can be conducted on multi-gram scales with metalation effected by mesitylcopper under prolonged heating (80 °C, 14 h) in benzene. The Cu center of 4 exhibits an intramolecular η^2^-arene with one of the phenyl substituents, albeit without significant elongation of the C–C bond (1.390(2) Å) (Fig. 3b). A fluxional interaction with the arene ring is evident by symmetric dipyrrin C–H resonances by ^1^H NMR spectroscopy. Although 4 is structurally similar to previously reported (^Ar^L)Cu (5),^54^ anodic shifts of the Cu^II/I^ redox couple by *ca*. 100 mV are observed by electrochemistry (4: *E*_1/2_ = 420 mV *vs.* Fc/Fc^1+^; 5: *E*_1/2_ = 310 mV *vs.* Fc/Fc^1+^), attributed to the more electron-deficient fluorinated substituent (Fig. S79†). The electron-deficient nature of 4 yields air-stability, in contrast to 5 which partially ligates O_2_ prior to the onset of decomposition into oligomeric Cu-containing species.^54^ The exchange of the *meso* arene for 4 relative to 5 yields marked changes by UV/vis spectroscopy (Fig. S77 and S78†), which has been previously observed by us in a series of ferrous dipyrrin coordination complexes.^58^ Gratifyingly, treatment of 4 with C_6_F_5_N_3_ in thawing toluene afforded the corresponding aminated toluene product (62%) by ^19^F NMR spectroscopy as the sole organic species, slightly augmented relative to that of 5 (45%), with the mass-balance identified as Cu^II^ species by integration of the incorporated ^19^F NMR ligand substituent. Treatment of either 4 or 5 with (4-^*t*^Bu)C_6_H_6_N_3_ afforded rapid detection of paramagnetic ^1^H NMR spectroscopy resonances akin to those of 3-*^t^*Bu without evidence of an organoazide adduct, suggesting Cu nitrene intermediates as viable intermediates in this transformation (Fig. S36†) (Scheme 2).

### 2.4. Stoichiometric mechanistic analysis

#### 2.4.1 Hammett analysis

**Fig. 4:**
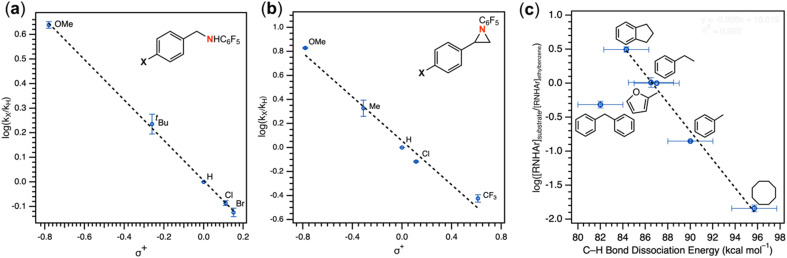
(a) Intermolecular competition Hammett amination with neat equimolar toluene and *para*-substituted toluene substrates with (^ArF^L)Cu (4). (b) Intermolecular competition Hammett aziridination with equimolar styrene and *para*-substituted styrene substrates in C_6_D_6_. (c) Competition amination experiments in neat equimolar ethylbenzene and substrate.

The mechanism of nitrene transfer from 4 was probed. For ease of assessing Cu speciation and quantifying product formation, nitrene transfer was monitored by ^19^F NMR spectroscopy using C_6_F_5_N_3_ as the arylazide source. Intermolecular competition amination experiments with 4 and stoichiometric C_6_F_5_N_3_ in an equimolar mixture of toluene and *para*-substituted toluene derivatives reveals an amination preference for electron-rich substrates (*ρ* = −0.82(1) against Hammett *σ*^+^ values, Fig. 4a and Table S1†) with minimal change in amination yield as a function of toluene substitution. Amination products containing the pentafluorophenyl amine moiety were independently synthesized through nucleophilic aromatic substitution of hexafluorobenzene with the corresponding alkyl amine, allowing for facile product identification by direct comparison of multinuclear (^1^H/^13^C{^1^H}/^19^F) NMR resonances. Similar negative *ρ* values have been attributed for electrophilic nitrene transfer with accumulation of positive charge at the putative nitrene fragment for intermolecular Co,^59^ Cu,^16^ and Rh^60–62^ C–H amination. A similar preference for consumption of electron-rich styrenes over electron-deficient styrenes is observed for intermolecular competition aziridination experiments from 4 (*ρ* = −0.92(2) against Hammett *σ*^+^ values, Fig. 4b and Table S2†), akin to values observed for Cu aziridination with iodoimine substrates.^20^ The linear correlations against *σ*^+^ values for amination (*R*^2^ = 0.99) and aziridination (*R*^2^ = 0.98) contrast those observed for intermolecular nitrene transfer from dipyrrin (^^*t*^Bu^L)FeCl(Et_2_O) and aziridination both by brominated tris(pyrazolyl)borate and tripodal guanidinato Cu complexes, which required employment of radical-delocalization parameters (*σ*_JJ_, *σ*_mb_) for satisfactory linear correlations.^16,63,64^

#### 2.4.2 Linear free energy relationship

Intermolecular amination experiments using 4 with neat equimolar toluene and competing substrate of various bond dissociation energies reveal a Bell–Evans–Polanyi relationship^65,66^ based on the linear relationship between reaction rate and bond dissociation energy, indicating substrate preference to be dictated by bond strength and not by other factors such as oxidation potential or substrate acidity (Fig. 4c).^67^ Nonetheless, the substrate steric profile was observed to contribute to substrate preference in competition experiments as evidenced by the lower-than-anticipated consumption of diphenylmethane (BDE_C–H_ = 84 kcal mol^−1^)^68^ to the corresponding amine, attributed to an entropic penalty for large substrates given the large dipyrrin aryl flanking substituents. In support of the hypothesis that sterically hindered substrates afford lower reactivity due to their steric bulk, attempted amination of tertiary C–H bonds for triphenylmethane (BDE_C–H_ = 81 kcal mol^−1^)^57^ and cumene (BDE_C–H_ = 85 kcal mol^−1^)^69^ resulted in no observed product formation by ^19^F NMR spectroscopy, attributed to the steric profile of the substrates (Fig. S49†). Moreover, amination of 2-methyltetrahydrofuran with 4 proceeds with exclusive amination of the less substituted α-ethereal carbon in a 2.2 : 1.0 diastereomeric ratio, further supporting a steric preference (Table 1, entry 1). The catalytic amination of 2-methyltetrahydrofuran employing 5 (*vide infra*) favors formation of the opposite diastereomer as evident by ^1^H NMR spectroscopy (1.0 : 1.7), illustrating an influential role of the *meso* arene on the resulting chemistry.

**Table 1:** Mechanistic probes for C–H amination and aziridination from (^ArF^L)Cu (4)^a^

Entry	Substrate	Product	Yield and commentary	Entry	Substrate	Product	Yield and commentary
1^b^	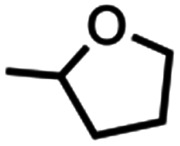	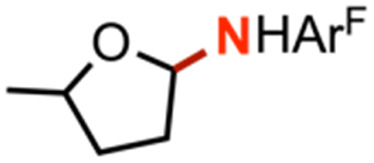	>99% 2° amination, no 3° amination	4^d^	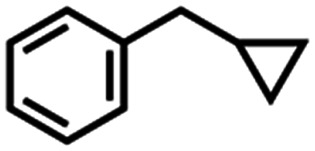	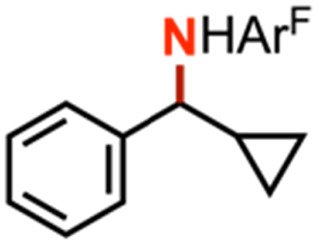	55(2)% no ring-opening
2^c^	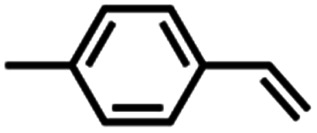	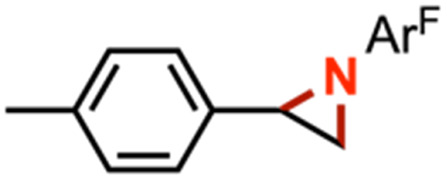	85(2)% aziridination, no amination	5^e^^,^^f^	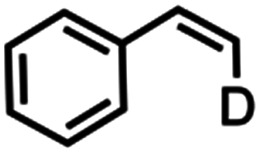	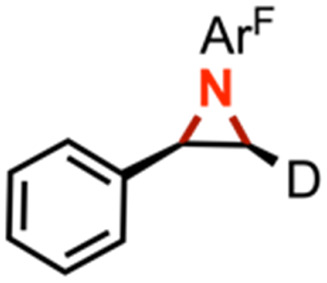	85(1)% detection of only single diastereomer
3^b^	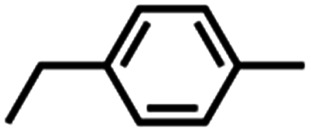	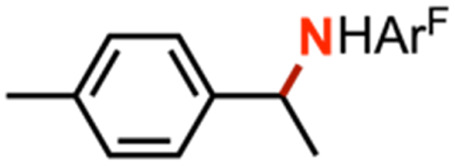	60(3)% enthalpy control 1.0(2°): 6.2(1°)	6^g^	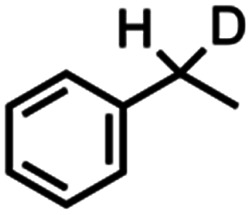	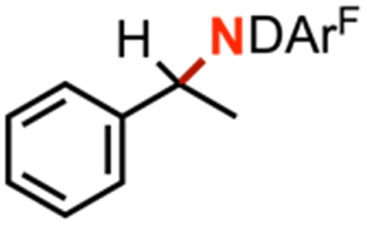	65(2)% sensitivity to H/D KIE 4.4(2)
		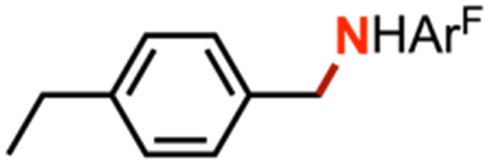				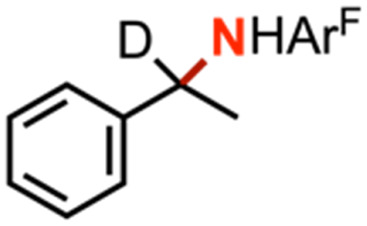	

a Yields determined by ^19^F NMR integration relative to fluorobenzene internal standard over a 16 h time frame from triplicate measurements in C_6_D_6_.

b Neat substrate.

c 5 equiv. substrate.

d 30 equiv. substrate.

e 10 equiv. substrate.

f Alternative diastereomer not observed by ^1^H NMR spectroscopy.

g 48 equiv. substrate. See ESI for reaction details.

#### 2.4.3 Substrate competition

Competition experiments further elucidated the electronic preference of 4 for C–H amination against aziridination. Treatment of 4 with C_6_F_5_N_3_ in the presence of 4-methylstyrene resulted in exclusive formation of the corresponding aziridine without any detectable benzylamine, indicative of a preference for aziridination over C–H amination (Table 1, entry 2). Treatment of 4 with C_6_F_5_N_3_ in neat 4-ethyltoluene resulted in a mixture of ethyl and methyl aminated products (1.0 : 6.2) favoring the weaker, more sterically precluded C–H bond (Table 1, entry 3). This ratio was similar to that observed for intermolecular amination of an equimolar mixture of toluene and ethylbenzene (1.0 : 7.1). In accord with the inability to functionalize cumene, competition amination using 4-isopropyltoluene proceeds without any detectable functionalization of the tertiary C–H site.

The mechanism of substrate activation was probed through radical clock experiments and kinetic isotope effect measurements. Amination of the radical clock phenyl(cyclopropyl)methane with either 4 or 5 furnishes the corresponding benzylic functionalized product with the cyclopropyl ring intact by ^1^H/^19^F NMR spectroscopy by comparison to an authentic amine sample (Fig. S52†). This observation is consistent with either a concerted amination process or a H-atom abstraction followed by the radical recombination step faster than the ring opening of the cyclopropyl unit.^70^ The absence of cyclopropyl ring-opening contrasts with amination observed from dimeric Cu β-diketiminate complexes with alkyl azides,^56^ attributed to either differences in radical clock lifetimes^71^ or mechanistic differences in C–N bond formation. Further consistent with the absence of long-lived radical intermediates, aziridination of (*Z*)-β-deuterostyrene^72^ proceeds with retention of stereochemistry in >20 : 1 values based on integration of ^1^H NMR spectroscopic resonances for the resulting aziridine with no diastereomer detected (Fig. S63†).

#### 2.4.4 Kinetic isotope effect

Kinetic isotope effect (KIE) measurements, including intermolecular competition amination with equimolar *h*_8_-toluene and *d*_8_-toluene as well as intramolecular competition amination with *d*_1_-ethylbenzene, resulted in values 9.0(7) and 4.4(2), respectively, and consistent with a stepwise hydrogen-atom abstraction step (Fig. S43–S45†). Minimal changes in intramolecular competition amination kinetic isotope effects were observed for *d*_1_-ethylbenzene with related arylazides 4-(CF_3_)C_6_F_4_N_3_, and 4-(CO_2_Me)C_6_F_4_N_3_, which respectively yielded values of 8.7(2) and 8.2(2). Amination of a neat equimolar mixture of *h*_12_-cyclohexane and *d*_12_-cyclohexane by 4 with C_6_F_5_N_3_ resulted in a measured intermolecular competition kinetic isotope effect of 8.4(4), suggesting no major KIE change as a function of C–H bond strength.

### 2.5. Catalytic nitrene transfer

To further understand the mechanism of nitrene transfer from 4, a kinetic analysis with 4 was conducted with catalytic loadings of 4. Although aziridination was observed with styrene for catalytic loadings of 4 (10 mol%), the ligation of styrene to 4 was observed to outcompete azide ligation based on ^1^H NMR spectroscopic resonances consistent with an adduct of styrene based on independent synthesis, resulting in prolonged reaction time of multiple days to achieve 3*t*_1/2_ conversion at 25 °C. Complex 4 (1.3 mM) was observed to selectively aminate the α-ethereal C–H bond of tetrahydrofuran (0.1 mol% 4) in neat substrate (0.7 mL) with C_6_F_5_N_3_ at 25 °C to yield the corresponding hemiaminal in 95(2)% yield by ^19^F NMR spectroscopy, with workup following extraction with cold pentane to remove 4 and cleanly isolate the resulting hemiaminal as an analytically pure species. Catalytic amination with 4 (5 mol%) could be similarity observed for substrates indane (64(1)% yield), 2-methyltetrahydrofuran (60(2)% yield), and diethyl ether (80(5)% yield) (Tables 2 and S4†), with a variety of electron-deficient arylazides. Most importantly, the background decomposition of 4 into the corresponding Cu^II^ anilide without substrate consumption is suppressed, allowing for a detailed kinetic analysis. For comparison, these yields could be improved by employing the more electron-rich analogue 5 for indane (96(1)% yield, 1 mol% 5), 2-methyltetrahydrofuran (>99% yield, 0.5 mol% 5), and tetrahydrofuran (>99% yield, 0.1 mol% 5). Related electron-deficient arylazides, including 3,5-(CF_3_)_2_C_6_H_3_N_3_, 4-(CF_3_)C_6_F_4_N_3_, and 4-(CO_2_Me)C_6_F_4_N_3_, were similarly observed to effect catalytic α-ethereal C–H bond amination of tetrahydrofuran (Table 2).

**Table 2:** Yields determined by ^19^F NMR integration relative to fluorobenzene internal standard over a 12 h time frame in neat substrate

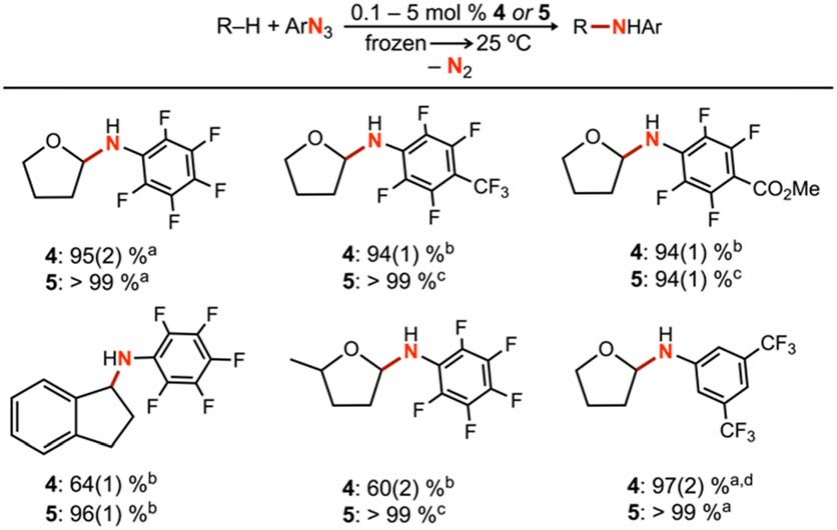

a 0.1 mol% catalyst loading for 36 h.

b 5.0 mol% catalyst loading.

c 0.5 mol% catalyst loading.

d Due to overlapping C–F resonances, the yield was determined by ^1^H NMR integration relative to an internal standard of fluoroanisole.

#### 2.5.1 Kinetic analysis

**Fig. 5:**
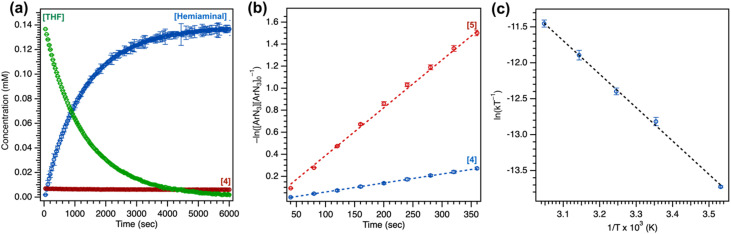
(a) Overview of consumption of tetrahydrofuran with (^ArF^L)Cu (4) (10 mol%) with C_6_F_5_N_3_ in THF to afford the corresponding hemiaminal. (b) Relative rates of tetrahydrofuran amination with 4 (10 mol%) and 5 (10 mol%). (c) Eyring analysis of tetrahydrofuran amination with 4 (10 mol%).

For kinetic analysis, consumption of C_6_F_5_N_3_ was monitored by *in situ*^19^F NMR spectroscopy with 4 or 5 (10 mol%), employing initial rates by monitoring the reaction to 10% consumption of arylazide to obviate potential issues for catalytic degradation (Fig. S55†) Nonetheless, 4 is retained at 98% with minimal conversion (<2%) to the Cu^II^ anilido species based on integration of *meso* arene trifluoromethyl substituents upon full consumption of C_6_F_5_N_3_, and reaction rates were observed to be identical measuring to greater extents of completion (Fig. 5a). Measurements were repeated in triplicate with average measurements reported and error bars representing the first standard deviation, and either fluorobenzene or 4-fluoroanisole was employed as an internal standard for ^1^H/^19^F NMR quantification. Monitoring the reaction by ^1^H/^19^F NMR spectroscopy in *d*_8_-tetrahydrofuran revealed 4 as the resting state, and titration experiments of 4 with variable quantities of *d*_8_-tetrahydrofuran in C_6_D_6_ revealed no noticeable changes in diamagnetic ^1^H NMR resonances, suggesting against a tetrahydrofuran adduct of 4 in accordance with the predicted low oxophilicity of the Cu^I^ oxidation state. The absolute rate of arylazide consumption was equal to the rate of hemiaminal production within error of ^19^F NMR measurements. The concentration of arylazide with respect to time was linearized by examining the logarithm of concentration, indicating a pseudo-first order overall reaction (Fig. 5b). Complex 4 aminates tetrahydrofuran at a slower rate than complex 5 (4: *k*_obs_ = 8.16(6) × 10^−4^ s^−1^ and *t*_1/2_ = 14.2 minutes; 5: *k*_obs_ = 4.34(9) × 10^−3^ s^−1^ and *t*_1/2_ = 2.7 minutes). Repeating tetrahydrofuran amination with 4 at various temperatures to extract activation parameters from Eyring analysis revealed a moderate positive enthalpy (Δ*H*^‡^ = 9.2(2) kcal mol^−1^) and a large negative entropy (Δ*S*^‡^ = −42(2) cal mol^−1^ K^−1^) indicative of a bimolecular rate-determining step (Fig. 5c, S66 and S67†). The free energy (Δ*G*^‡^_298K_ = 21.7(2) kcal mol^−1^) is in accord with the amination of tetrahydrofuran at room temperature.

#### 2.5.2 Order analysis

**Fig. 6:**
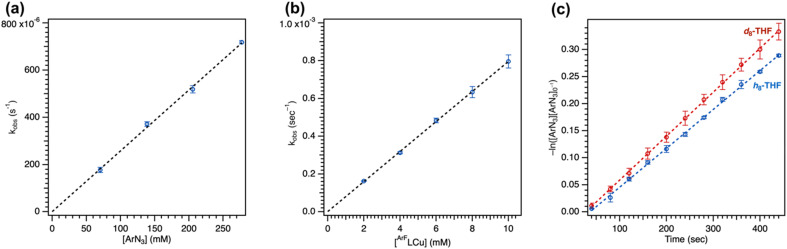
(a) Ordering analysis for variable C_6_F_5_N_3_ concentration. (b) Ordering analysis for variable (^ArF^L)Cu (4) concentration. (c) Kinetic isotope effect measurement with 4 (10 mol%) and C_6_F_5_N_3_ using *h*_8_-tetrahydrofuran or *d*_8_-tetrahydrofuran.

Plotting the slope of arylazide consumption as a function of 4 or arylazide reveals a linear relationship (Fig. 6a and b), indicating both first-order dependencies (Fig. S68–S71†). This observation is in accord with the apparent first-order decay of arylazide under the reaction conditions, given a constant concentration of 4 and the vast excess of substrate under catalysis. No change in C_6_F_5_N_3_ consumption was observed in the presence of excess tetrahydrofuran (>500 equiv.) (Fig. S72 and S73†). Nonetheless, employing reduced equivalents of tetrahydrofuran (<100 equiv.) in C_6_D_6_ resulted in an apparent increase in background degradation to yield the corresponding Cu^II^ anilido, preventing an assessment of reaction order on substrate under lower loadings of substrate.

#### 2.5.3 Kinetic isotope measurements

Kinetic isotope effect measurements were conducted to identify the involvement of substrate in the overall reaction profile. Intramolecular cyclization of alkyl azides by Fe,^73^ Co,^53,74^ and Ni^75^ dipyrrin complexes showed high sensitivity to the presence of a C–H or C–D bond with large non-classical kinetic isotope effect values. For intermolecular H-atom abstraction or amination reactivity, non-classical kinetic isotope effects were additionally observed from isolable metal nitrenoid species.^44,46,49^ By contrast, repeating parallel amination trials in neat *h*_8_-tetrahydrofuran and *d*_8_-tetrahydrofuran resulted in only a minimal change in the overall rate based on the observed kinetic isotope effect of 1.10(2), suggesting against rate-limiting H-atom abstraction, although concerted C–H insertion or an asymmetric transition state cannot be ruled out from this value (Fig. 6c).^76,77^ Nonetheless, measurement of competition intramolecular amination with 2,2-*d*_2_-tetrahydrofuran^78^ and competition intermolecular amination with an equimolar mixture of *h*_8_-tetrahydrofuran and *d*_8_-tetrahydrofuran revealed larger kinetic isotope effect values of 4.7(1) and 6.2(2) (Fig. S74†), indicative of a sensitivity of the overall reaction to identity of the C–H or C–D bond of the substrate. Curiously, a larger absolute kinetic isotope effect was observed from 5 (2.06(1)) with similar changes in the intramolecular and intermolecular amination of 8.1(1) and 10.7(4) (Fig. S75†), suggesting the mesityl substituent may impact the underlying kinetics and rate-determining step of the overall reaction.

Lastly, noting the capacity of sterically encumbered Cu β-diketiminate species to conduct C–H bond amidation of inert hydrocarbons with aroyl azides, the analogous transformation was targeted with 1 and 4. Interestingly, treatment of 1 (1 mol%) with 4-methoxybenzoyl azide in C_6_D_6_ at room temperature afforded the corresponding aryl isocyanate confirmed through independent synthesis. The reaction was completed with *ca.* 10 minutes in quantitative yield with recovery of 1 (Fig. S82†). By contrast, treatment of 4 with stoichiometric 4-methoxybenzoyl azide afforded full consumption of 4 and a distinct paramagnetic species as ascertained by ^1^H/^19^F NMR spectroscopy and EPR analysis, attributed to formation of the corresponding Cu^II^ amide species. These results underscore the importance of steric profile on reaction trajectory.

### 2.6. Computations

**Fig. 7:**
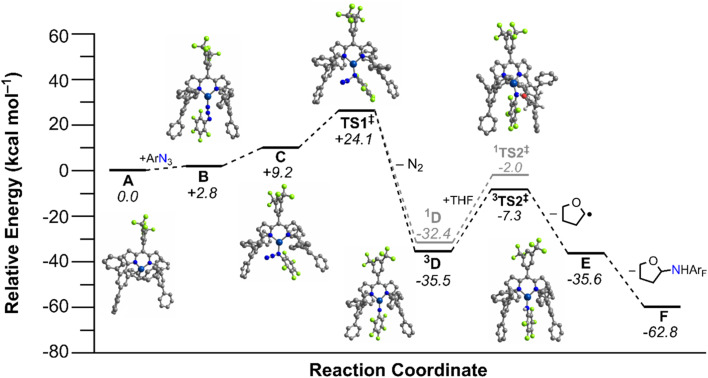
Calculated [ONIOM(B3LYP/6-311++G(d,p):UFF)] free energy diagram (kcal mol^−1^) from tetrahydrofuran amination mediated *via* (^ArF^L)Cu (4).

Calculations were conducted using the Gaussian 16 program^79^ to corroborate kinetic measurements for tetrahydrofuran amination by 4 and elucidate the underlying elementary steps (Fig. 7). Hybrid QM/MM calculations (see Fig. S93† in the ESI for the QM/MM partition scheme used) utilized the ONIOM method,^80^ with the Universal Force Field (the phenyl groups of the quadraphenyl substituent).^81^ The DFT partition utilized the B3LYP functional,^82–85^ and a two-step sequence involving a geometry optimization plus vibrational frequency step using the 6-31+G(d) basis set, followed by larger basis set single point calculations for more accurate energetics utilizing the 6-311++G(d,p) basis set.^86^ Calibration and additional details regarding this approach are described in the ESI.† In general, the results of the two-step scheme mirror the one-step approach (using exclusively the larger basis set) results well, with most free energies only varying by *ca.* ±1–2 kcal mol^−1^ (Table S13†).

The reference free energy was defined as the separate reactants, consisting of 4, pentafluorophenyl azide, and tetrahydrofuran (A, Fig. 7). Two isomers of the organoazide complex of the catalyst were considered, (^ArF^L)Cu(κ^1^-N_γ_-C_6_F_5_N_3_) (B) and (^ArF^L)Cu(κ^1^-N_α_-C_6_F_5_N_3_) (C). Calculations favor formation of more sterically exposed B, which is more easily accessible with a relative Gibbs free energy (*G*_rel_) that is 2.8 kcal mol^−1^ compared to A. Formation of the internal isomer C has *G*_rel_ = 9.2 kcal mol^−1^*versus*A.

A transition state (TS) for formation of a copper-nitrenoid intermediate *via* N_2_ elimination was considered next; this open-shell singlet TS1 has a free energy barrier of Δ*G*^‡^ = 24.1 kcal mol^−1^. The activation entropy (Δ*S*^‡^) for N_2_ elimination (*T* = 298.15 K) was calculated to be −40.0 cal mol^−1^ K^−1^, consistent with the activation entropy from Eyring analysis of −42(2) cal mol^−1^ K^−1^. The calculated activation enthalpy (Δ*H*^‡^) for N_2_ extrusion (12.2 kcal mol^−1^) was further consistent with the experimentally determined activation enthalpy through Eyring analysis (9.2(2) kcal mol^−1^).

Removal of N_2_ affords a copper-nitrenoid intermediate, for which both singlet and triplet spin states were evaluated, ^3^[Cu] = NAr (^3^D) and ^1^[Cu] = NAr (^1^D). The *G*_rel_ for closed-shell singlet ^1^D and triplet ^3^D are respectively Δ*G* = −32.4 kcal mol^−1^ and −35.5 kcal mol^−1^. Hence, the triplet is predicted to be more stable by *ca.* 3 kcal mol^−1^. Additional B3LYP/6-311++G(d) geometry optimizations with Gaussian 16 and ORCA 4.2.1 ^87^ suggest that the singlet state of ^1^D is a closed-shell singlet with unsuccessful attempts to isolate an open-shell singlet analogue of ^3^D.

The singlet and triplet transition state for C–H bond activation of tetrahydrofuran have either *G*_rel_ = −2.0 kcal mol^−1^ (^1^TS2^‡^) or −7.3 kcal mol^−1^ (^3^TS2^‡^), Fig. 7. These free energies represent a calculated C–H activation barrier of Δ*G*^‡^ = 28.3 kcal mol^−1^ (Δ*S*^‡^ = 48.4 cal mol^−1^ K^−1^ at 298 K) for the more stable triplet surface relative to the nitrene intermediate. To activate the C–H bond of THF, a hydrogen is abstracted from the substrate resulting in an anilido (^2^[Cu]–NHAr) and the activated substrate radical (E). Two radicals were initially considered: one with the C–H activation site proximal to the oxygen atom in THF, and one with the activation site distal to the oxygen atom. The relative free energy for the separated amide and each radical are −35.6 kcal mol^−1^ and −32.7 kcal mol^−1^, respectively. This indicates that the activation at the 2 position *via* C–H activation is thermodynamically preferred, consistent with the experimentally observed selectivity. The last step considered in the reaction coordinate is the radical rebound of the amide complex to the organic radical generated by H-atom abstraction of THF, yielding the desired amination product, and recovery of the isolated catalyst. Formation of the amination product results in a relative energy of −62.8 kcal mol^−1^*versus* separated catalyst, organoazide and THF reactants (F). Importantly, we were unable to locate a transition state prior to recombination of the alkyl radical, suggesting a barrierless transition state for radical capture.

To further corroborate the results of the B3LYP calculations, the relative Gibbs free energy was obtained for all stationary points in Fig. 7 using the wB97XD functional,^88^ except for the N_2_ elimination transition state, which we were unable to get to converge. The wB97XD functional uses a version of Grimme's D2 dispersion model^89^ and therefore corrects the long-range behavior of the functional. The results obtained with the wB97XD functional are given in Table S15 in the ESI.† While the *G*_rel_ for most of the stationary points in the reaction coordinate become lower in energy by 5–10 kcal mol^−1^, the energy shift was similar across many of the stationary points, and thus the conclusions remain the same as for the B3LYP calculations. For example, the triplet copper-nitrenoid intermediate, ^3^D, is still predicted to be lower in energy than the closed-shell singlet, ^1^D, in this case, by 14 kcal mol^−1^ (*G*_rel_ (^3^D) = −40.6 kcal mol^−1^ and *G*_rel_ (^1^D) = −26.8 kcal mol^−1^) using the wB97XD functional. Additionally, the effects of implicit solvation in THF were considered *via* DFT calculations. These calculations used the Polarizable Continuum Model (PCM)^90,91^ for implicit solvation,^92^ as implemented in the Gaussian 16 program.^93^ The continuum solvent calculations utilized the B3LYP functional, and geometry optimizations were initiated from the previously obtained gas-phase B3LYP geometries. As for the calculations done with the wB97XD functional, some of the stationary points, such as the separated amide and THF radicals, have relative energies that are lower than those obtained with B3LYP (see Table S13 in the ESI†). Again however, all previous conclusions regarding the more stable transition states, *etc*., remain consistent in the solvation calculations as compared to the energies obtained using B3LYP. For example, the triplet-spin transition state for C–H bond activation of tetrahydrofuran remains lower in energy (*G*_rel_ = −19.4 kcal mol^−1^) than the unrestricted singlet-spin N_2_-elimination transition state (*G*_rel_ = 25.9 kcal mol^−1^), but when implicit solvation is accounted for, this difference is more pronounced (Δ*G* = 45.3 kcal mol^−1^ when implicit solvation is considered, *versus* Δ*G* = 31.8 kcal mol^−1^ without solvation effects).

### 2.7. Electronic structure analysis

**Fig. 8:**
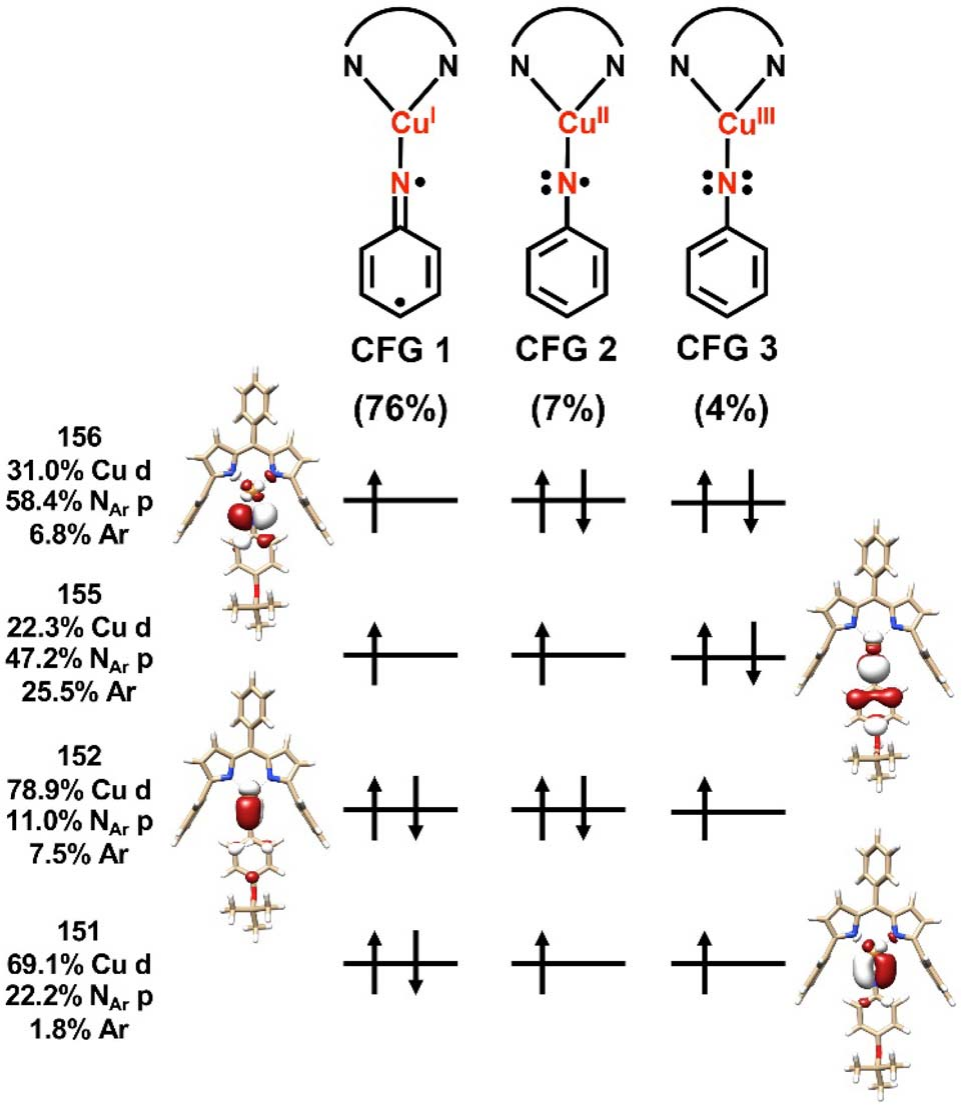
Leading configurations comprising (6,4) subspace of SORCI calculation on a truncated model of 3-O*^t^*Bu. Averaged atomic natural orbitals are plotted at an isovalue of 0.03 au.

Spectroscopy-oriented configuration interaction (SORCI) calculations based on a complete active space self-consistent field (CASSCF) reference were first carried out on a truncated model derived from the crystallographic coordinates of 3-O*^t^*Bu (Fig. 8). Hydrogen atom positions were optimized with the B3LYP density functional. The SORCI results indicate a multiconfigurational triplet ground state (Fig. S89†) in agreement with previous investigations into the electronic structure of 3-*^t^*Bu.^40^ The Cu^I^-triplet nitrene configuration (CFG 1) is the most significant contributor to the ground state (76%) in which the unpaired electrons reside in the N 2p_*x*_ and 2p_*y*_ orbitals (MOs 155 and 156) of the coordinated nitrene. Two other low-weight configurations can be identified as a ferromagnetic Cu^II^-iminyl configuration (CFG 2; 7%) and a Cu^III^-imido configuration (CFG 3; 4%); all other configurations contribute less than 1% each to the ground state.

The computed electronic structure of 3-O*^t^*Bu corroborates the XAS-derived formulation of the ground state and further supports the assignment of 3-O*^t^*Bu as a Cu^I^(^3^NAr) adduct, as opposed to higher valent Cu^II^ or Cu^III^ species. The average 3d character per hole estimated from configurations with greater than 1% contribution to the ground state is 27%, consistent with the intensity of the observed Cu L_3_- and L_2_-edge main lines and assignment of the metal center as physically Cu^I^ (Table 3). Similar summation over the calculated N 2p contributions to the acceptor orbitals from configurations with greater than 1% contribution to the ground state are consistent with the slight increase in intensity of the N K-edge pre-edge features observed with 3-O*^t^*Bu compared to 3-*^t^*Bu. The unpaired electron residing in the N 2p_*x*_ orbital is conjugated with the aryl nitrene (MO 155) while the unpaired electron resides in the N 2p_*y*_ orbital, which is orthogonal to the aryl π system, further lifting the degeneracy of the N 2p_*x*_ and 2p_*y*_ orbitals. The asymmetry of the N K-edge pre-edge features arising from the nitrene can be understood in terms of simple single-electron transitions into the N 2p_*x*_ and 2p_*y*_ orbitals, explaining both the difference in energy and relative intensity of the observed features.

**Table 3:** Calculated estimations for average Cu and N character per hole^a^

	3-O*^t^*Bu^b^	3-*^t^*Bu^b^^,^^d^	3-O*^t^*Bu^c^	3-*^t^*Bu^c^	(^ArF^L)Cu(NC_6_F_5_)^c^
Calculated average Cu 3d character per hole	27%	21%	26%	35%	24%
Calculated average N_Ar_ 2p character per hole	43%	44%	44%	37%	39%

a Only configurations contributing greater than 1% to the ground state are considered.

b Coordinates employed from truncated solid-state structure.

c Coordinates employed from truncated geometry optimized structure.

d Average Cu 3d and N_Ar_ 2p character per hole estimated from the two major configurations previously published.

Due to the reactive nature of 4, we were unsuccessful at the detection of reactive intermediates by NMR spectroscopy with C_6_F_5_N_3_ as the azide source. Additional SORCI calculations were conducted to gain insight into the impact of the perfluoroarene substituent on the ground state electronic structure. Similar to 3-O*^t^*Bu and 3-*^t^*Bu, a triplet ground state in which the unpaired electrons principally reside in the N 2p_*x*_ and 2p_*y*_ orbitals was predicted (Fig. S90†). As 4 could not be isolated, it was necessary to obtain the structure from density functional theory (DFT) geometry optimization. Because the ground states of 3-O*^t^*Bu and 3-*^t^*Bu are highly multiconfigurational and thus not well described by DFT, we also performed geometry optimizations on these structures and repeated the CASSCF/SORCI procedure for truncated models derived from the DFT-optimized structures (Fig. S91 and S92†). In both cases, the electronic structure was best described as a Cu^I^(^3^NAr) adduct, though the composition of the acceptor orbitals was in weaker quantitative agreement with experiment. Taking this into consideration, a clear trend in arene functionalization and electronic structure could not be unambiguously identified across the series of three Cu^I^(^3^NAr) adducts from the multiconfigurational calculations alone. In all three cases, however, an inverted ligand field was indicated in which the Cu valence orbitals were lower in energy than the corresponding nitrene valence orbitals, resulting in redox-active MOs of predominantly nitrene character in what has been termed ligand field inversion.^43,94^

## 3 Conclusions

**Fig. 9:**
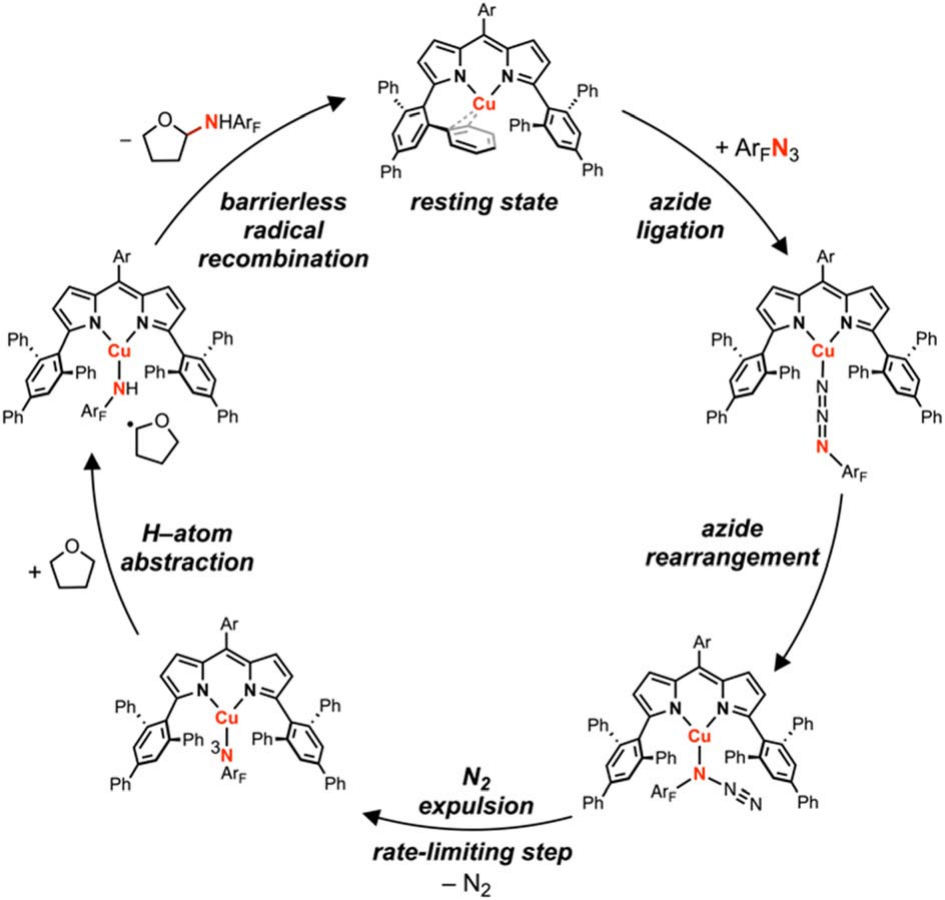
Proposed catalytic cycle for tetrahydrofuran amination by (^ArF^L)Cu (4) and (^Ar^L)Cu (5).

A dipyrrin-supported Cu^I^ synthon was demonstrated to mediate C–H bond amination and aziridination of exogeneous substrates using electron-deficient arylazides, proposed to proceed through an intermediate Cu nitrene species (Fig. 9). Arylazide activation involves ligation through the sterically exposed terminal nitrogen (N_γ_), followed by rearrangement to the internal nitrogen (N_α_) with subsequent irreversible expulsion of N_2_. Hammett analyses reveal the Cu nitrenoid intermediate, observable by ^1^H NMR spectroscopy for certain electron-deficient arylazides, behaves as an electrophilic nitrene reagent, akin to other putative amination reactive intermediates.^28^ Amination proceeds with a Bell–Evans–Polanyi relationship, although sterically precluded tertiary C–H bonds remain unfunctionalized, likely due to steric clashing with the quadraphenyl motifs of the ligand scaffold. Nitrene transfer to tetrahydrofuran as a model substrate revealed pseudo-first order decay in arylazide under reaction conditions with order dependence on both Cu and arylazide. Eyring analysis and computations are consistent with rate-limiting Cu nitrenoid formation, contrasting previous dipyrrin amination catalysis in which hydrogen-atom abstraction is measured as the rate-determining step. This discrepancy can be attributed to the difference in reduction potential between Cu^I^ and more reducing metal ions such as Ni^I^ or Co^I^. The large barrier may be in part due to the absence of metal–ligand multiple bonding formation during N_2_ extrusion. Furthermore, the subsequent radical recombination step is barrierless for Cu, contrasting a Ni intramolecular amination catalyst which exhibit loss of stereochemical information due to a non-negligible radical recombination barrier.^95^ These results will guide the improvement of future amination catalysts, with detailed computations addressing the impact of the nitrene aryl substituent on the resulting electronic structure underway.

## Data availability

All experimental and computational data is included in the ESI file.†2 CCDC Identifier: 2080873; 4 CCDC Identifier: 2080874.

## Author contributions

K. M. C. and T. A. B. conceived the experimental design, executed syntheses, performed kinetic analysis, and assessed both catalytic and stoichiometric nitrene transfer reactivity. S. N. and T. R. C. conducted computational analysis of nitrene free energy landscape using density functional theory. I. D. M., T. K., and K. M. L. conducted XANES measurements and multiconfigurational calculations. A. I. and P. V. assisted K. M. C. in substrate synthesis and independent preparation of organic products. S. L. Z. assisted K. M. C. in the structural refinement of 2. All authors contributed to the construction of this manuscript.

## Conflicts of interest

There are no conflicts to declare.

## Supplementary Material

SC-014-D3SC03641C-s001

SC-014-D3SC03641C-s002
