# Supplementary material for: Nitrene transfer from a sterically confined copper nitrenoid dipyrrin complex
Source: Chem Sci. 2023 Sep 26;14(39):10847–60. doi: 10.1039/d3sc03641c (PMC10566472; doi:10.1039/d3sc03641c)

Electronic Supplementary Material (ESI) for Chemical Science.
This journal is © The Royal Society of Chemistry 2023

Nitrene Transfer from a Sterically Confined Copper Nitrenoid Dipyrrin Complex

Kurtis M. Carsch,[†] Sasha C. North,[§] Andrei Iliescu,[†] Ida M. DiMucci,[‡] Petra Vojackova,[†] Shao Liang-Zheng,[†] Thomas R. Cundari,[§] Kyle M. Lancaster,[‡] Theodore A. Betley[†]

[†] Department of Chemistry and Chemical Biology
Harvard University
Cambridge, MA 02138

[‡] Department of Chemistry and Chemical Biology
Baker Laboratory
Cornell University
Ithaca, New York 14853

[§] Center for Advanced Scientific Computing and Modeling (CASCaM)
Department of Chemistry
University of North Texas
Denton, TX 76203, USA

Section	Page
**Table of Contents**	**S2**
**Materials and Methods**	**S5**
General Considerations	S5
Characterization and Physical Measurements	S6
**Supporting Ligand and Metal Complex Syntheses**	**S7**
(^{EMind}L)Cu(N_3Ad) (**2**) Synthesis	S7
Figure S1. ^{1}H NMR Spectrum of (^{EMind}L)Cu(N_3Ad) (**2**)	S8
Figure S2. ^{13}C NMR Spectrum of (^{EMind}L)Cu(N_3Ad) (**2**)	S8
Figure S3. ^{19}F NMR Spectrum of (^{EMind}L)Cu(N_3Ad) (**2**)	S8
Figure S4–S5. IR Spectra of (^{EMind}L)Cu(N_3Ad) (**2**)	S9
(^{Ar_F}L)H Synthesis	S11
Figure S6. ^{1}H NMR Spectrum of (^{Ar_F}L)H	S12
Figure S7. ^{13}C NMR Spectrum of (^{Ar_F}L)H	S12
Figure S8. ^{19}F NMR Spectrum of (^{Ar_F}L)H	S12
(^{Ar_F}L)Cu (**4**) Synthesis	S13
Figure S9. ^{1}H NMR Spectrum of (^{Ar_F}L)Cu (**4**)	S14
Figure S10. ^{13}C NMR Spectrum of (^{Ar_F}L)Cu (**4**)	S14
Figure S11. ^{19}F NMR Spectrum of (^{Ar_F}L)Cu (**4**)	S14
**Additional Organic Syntheses**	**S15**
1-(azido-1-^{15}*N*)-4-(tert-butyl)benzene Synthesis	S15
Figure S12–S13. Supporting IR Spectra of (4-^{t}Bu)C_6H_4)N_3	S16
1-(azido-1-^{15}*N*)-adamantane Synthesis	S18
Figure S14–S15. Supporting IR Spectra of AdN_3	S19
N-(cyclopropyl(phenyl)methyl)-2,3,4,5,6-pentafluoroaniline Synthesis	S21
Figure S16. ^{1}H NMR Spectrum of *N*-(cyclopropyl(phenyl)methyl)-2,3,4,5,6-pentafluoroaniline	S22
Figure S17. ^{13}C NMR Spectrum of *N*-(cyclopropyl(phenyl)methyl)-2,3,4,5,6-pentafluoroaniline	S22
Figure S18. ^{19}F NMR Spectrum of *N*-(cyclopropyl(phenyl)methyl)-2,3,4,5,6-pentafluoroaniline	S22
N-(2,3,5,6-tetrafluoro-4-(trifluoromethyl)phenyl)tetrahydrofuran-2-amine Synthesis	S23
Figure S19. ^{1}H NMR Spectrum of Aminated Tetrahydrofuran	S24
Figure S20. ^{13}C NMR Spectrum of Aminated Tetrahydrofuran	S24
Figure S21. ^{19}F NMR Spectrum of Aminated Tetrahydrofuran	S24
Methyl 2,3,5,6-tetrafluoro-4-((tetrahydrofuran-2-yl)amino)benzoate Synthesis	S25
Figure S22. ^{1}H NMR Spectrum of Aminated Tetrahydrofuran	S26
Figure S23. ^{13}C NMR Spectrum of Aminated Tetrahydrofuran	S26
Figure S24. ^{19}F NMR Spectrum of Aminated Tetrahydrofuran	S26
5-methyl-*N*-(perfluorophenyl)tetrahydrofuran-2-amine Synthesis	S15
Figure S25. ^{1}H NMR Spectrum of 5-methyl-*N*-(perfluorophenyl)tetrahydrofuran-2-amine	S27
Figure S26. ^{13}C NMR Spectrum of 5-methyl-*N*-(perfluorophenyl)tetrahydrofuran-2-amine	S28
Figure S27. ^{19}F NMR Spectrum of 5-methyl-*N*-(perfluorophenyl)tetrahydrofuran-2-amine	S28
N-(perfluorophenyl)furan-2-amine Synthesis	S29
Figure S28. ^{1}H NMR Spectrum of *N*-(perfluorophenyl)furan-2-amine	S30
Figure S29. ^{13}C NMR Spectrum of *N*-(perfluorophenyl)furan-2-amine	S30
Figure S30. ^{19}F NMR Spectrum of *N*-(perfluorophenyl)furan-2-amine	S30
N-benzhydryl-2,3,4,5,6-pentafluoroaniline Synthesis	S31
Figure S31. ^{1}H NMR Spectrum of *N*-benzhydryl-2,3,4,5,6-pentafluoroaniline	S18
Figure S32. ^{13}C NMR Spectrum of *N*-benzhydryl-2,3,4,5,6-pentafluoroaniline	S18

N-(4-ethylbenzyl)-2,3,4,5,6-pentafluoroaniline & 2,3,4,5,6-pentafluoro-*N*-(1-(*p*-tolyl)ethyl)aniline Syntheses	S33
Figure S34–S35. ^{19}F NMR Spectrum of Aminated Alkylbenzene	S34
**Stoichiometric Mechanistic Analysis**	**S35**
Figure S36. Detection of Copper Nitrene Species by ^{1}H NMR Spectroscopy	S35
Figure S37–S38, Table S1. Competition Styrene Aziridination Experiments	S36
Figure S39–S40, Table S2. Competition Toluene Amination Experiments	S38
Figure S41–S42, Table S3. Competition Substrate Amination Experiments	S40
Figure S43–S44. Intermolecular h_8–toluene/d_8–toluene Competition KIE Measurement	S42
Figure S45. Intramolecular d_1-ethylbenene Competition KIE Measurement	S44
Figure S46. Intramolecular Aziridination *vs.* Amination Experiment	S45
Figure S47–S48. Intramolecular Benzylic Primary *vs.* Secondary Amination Experiment	S46
Figure S49. Intramolecular Cumene Amination Experiment	S48
Figure S50–S51. Intramolecular 2-methyltetrahydrofuran Amination Experiment	S49
Figure S52. Radical Clock Amination	S50
Figure S53–S54. Stoichiometric Background Decomposition Measurement	S51
**Catalytic Mechanistic Analysis**	**S53**
Table S4. Catalytic Mechanistic Analysis	S53
Figure S55. ^{19}F NMR Timecourse of Tetrahydrofuran Amination with (^{Ar_F}L)Cu (**4**)	S54
Figure S56. ^{19}F NMR of Tetrahydrofuran Amination with (^{Ar}L)Cu (**5**)	S54
Figure S57–S58. Catalytic Intramolecular Kinetic Isotope Effect Measurement	S55
Figure S59–S62. Catalytic Intermolecular Kinetic Isotope Effect Measurement	S57
Figure S63. Catalytic (Z)–β–deuterostyrene aziridination Measurement	S60
**Kinetic Analysis**	**S61**
Figure S64–S65, Table S5. Tetrahydrofuran Amination with (^{Ar_F}L)Cu (**4**) and (^{Ar}L)Cu (**5**)	S62
Figure S66–S67, Table S6. Eyring Analysis for Tetrahydrofuran Amination with (^{Ar_F}L)Cu (**4**)	S64
Figure S68–S69, Table S7. Catalyst Loading Analysis with (^{Ar_F}L)Cu (**4**)	S67
Figure S70–S71, Table S8. Aryl Azide Loading Analysis with (^{Ar_F}L)Cu (**4**)	S70
Figure S72–S73, Table S9. Substrate Loading Analysis with (^{Ar_F}L)Cu (**4**)	S73
Figure S74–S75. Parallel Kinetic Isotope Effect Measurement with (^{Ar_F}L)Cu (**4**) and (^{Ar}L)Cu (**5**)	S76
**Additional Physical Characterization and Spectroscopic Characterization**	**S79**
Figure S76. Comparison of IR Spectra for (^{EMind}L)Cu($N_3C_6H_4{}^tBu$)	S79
Figure S77–S78. UV/Vis and Optical Spectra of (^{Ar_F}L)H, (^{Ar_F}L)Cu (**4**), (^{Ar}L)H, and (^{Ar}L)Cu (**5**)	S80
Figure S79. Electrochemical Comparison of (^{Ar_F}L)Cu (**4**) and (^{Ar}L)Cu (**5**)	S82
Figure S80–S81. Post-Amination EPR Analyses	S83
Figure S82. Catalytic Curtius Rearrangement of Aroyl Azides	S85
**X-ray Absorption (XAS) Spectra**	**S86**
Figure S83–S86. Gaussian Fit of Absorption Spectra	S86
**X-ray Diffraction Experiment Details**	**S88**
Table S10. XRD Experimental Details and Refinement Protocol	S88
Figure S87. Solid-state Structure of (^{EMind}L)Cu(N_3Ad) (**2**)	S91
Table S11. Pertinent Bond Metrics of (^{EMind}L)Cu(N_3Ad) (**2**)	S91
Figure S88. Solid-state Structure of (^{Ar_F}L)Cu (**4**)	S92
Table S12. Pertinent Bond Metrics of (^{Ar_F}L)Cu (**4**)	S92
**Additional SORCI Calculations**	**S93**
Figure S89–S92. Leading Configurations of Copper Nitrenoid Configurations	S93
**Density Functional Theory Calculations**	**S97**
Figure S93. QM/MM Calculation Partition	S97

Table S13. Comparison of Relative Gibbs Free Energies Using One- and Two-Step Calculations S98
Table S14. CPU Time Comparison for One-Step and Two-Step Computations S98
Table S15. Relative Gibbs Free Energies Obtained Using the wB97XD Functional S98
Table S16. Relative Gibbs Free Energies Obtained Using the PCM and B3LYP functional S99
Geometry Output Cartesian Coordinates S99
Respective Contributions S121
References S122
CheckCIF Output S125

Materials and Methods.

General Considerations. All manipulations were carried out in the absence of water and dioxygen using standard Schlenk techniques or in an MBraun drybox under an inert dinitrogen atmosphere, unless specified otherwise. All glassware was oven dried at 150 ºC for a minimum of 12 h and cooled in an evacuated antechamber for a minimum of 15 minutes prior to use in the drybox. Benzene, diethyl ether, dichloromethane (DCM), acetonitrile (MeCN), hexanes, pentane, toluene, and tetrahydrofuran (THF) were dried over 4 Å molecular sieves (Strem) prior to use. When applicable, solvents were tested with a deep violet solution of sodium benzophenone ketyl in tetrahydrofuran (prepared by stirring 10 mg benzophenone in 10 mL THF with excess metallic sodium for 12 h) to confirm effective oxygen and moisture removal. Chloroform-d_1 was purchased from Cambridge Isotope Labs and stored over anhydrous potassium carbonate with exclusion of light. Benzene-d_6 and tetrahydrofuran-d_8 were purchased from Cambridge Isotope Labs, degassed, and stored over 4 Å molecular sieves prior to use. Mesitylcopper was prepared according to a literature protocol and recrystallized three times from slow cooling of a warm toluene solution, followed by a cold diethyl ether wash.[1] Isotopically labelled d_1–ethylbenzene was prepared by treatment of α–phenylethylpotassium[2] with stoichiometric D_2O in thawing pentane, followed by filtration, removal of pentane, and desiccation. Ammonium chloride (^{15}N, 99 %) was purchased from Cambridge Isotope Labs and used as received. Complexes ($^{EMind}L$)Cu(N_2) (**1**), ($^{EMind}L$)Cu(N($C_6H_4O^tBu$)) (**3**), and (^{Ar}L)Cu (**5**) were prepared according to literature procedures.[3] Pyridinium p-toluenesulfonate (PPTS), hexafluorobenzene, octafluorotoluene, furfuryl amine, methyl pentafluorobenzoate, 2,3-dichloro-5,6-dicyano-1,4-benzoquinone (DDQ), and were purchased from Sigma-Aldrich and used as received. Adamantyl azide (1-azidoadamantane) was purchased from Sigma-Aldrich and filtered through silica in the dybox with pentane prior to use. Cyclopropyl(phenyl)methanamine•HCl was purchased from VWR and used as received. NaK (sodium-potassium alloy) was prepared by heating a 1:3 wt% Na^0/K^0 mixture until homogeneous, followed by pipetting the interior volume to remove surface oxides.

All amination and aziridination substrates were purchased from commercial vendors, if available, and were distilled from calcium hydride, degassed under vacuum, passed through alumina or silica, and stored in the freezer in the drybox at –35 ºC over molecular sieves prior to use.

Celite® 545 (J. T. Baker) was dried in a Schlenk flask for 24 h under dynamic vacuum while heating to at least 150 °C prior to use in a drybox. Activated alumina was dried in a Schlenk flask for 48 h under dynamic vacuum at 200 ºC prior to use in a drybox. Silica gel 32-63 μ (AIC, Framingham, MA) was used as received.

When appropriate, glassware was silanized with a 10% v/v dichlorodimethylsilane ($SiCl_2Me_2$, Aldrich)/anhydrous hexanes solution under air for 30 minutes, followed by an additional hexane rinsing, quenching with absolute ethanol, and oven drying at 150 ºC for 12 h prior to use.

Caution!! Organic azides are known to be potentially explosive compounds.[4] While we did not encounter any issues during their syntheses or manipulations, proper precautions were taken. All reactions involving organic azides at elevated temperatures were conducted behind a blast shield.

All organic azides were stored under nitrogen in a –35 ºC freezer and filtered through silica prior to use.

Characterization and Physical Measurements.

NMR Spectroscopy. ^{1}H, ^{13}C[5], ^{31}P, and ^{19}F NMR spectra were recorded on Varian Unity/Inova 400, 500, or 600 MHz spectrometers. ^{1}H and ^{13}C[5] NMR chemical shifts are reported relative to $SiMe_4$ using the chemical shift of residual solvent peaks as reference. ^{19}F NMR chemical shifts are reported relative to an external standard of boron $BF_3(OEt_2)$ (δ –153.00 ppm). ^{31}P NMR chemical shifts are referenced to an external standard of 85 % H_3PO_4 (δ 0.00 ppm). Multiplicity assignments are abbreviated as follows: s = singlet, d = doublet, t = triplet, q = quartet, m = multiplet, br = broad, v. br = very broad.

IR Analysis. Infrared spectra were acquired on a Varian 1000 FT-IR spectrometer through use of KBr pellet samples (*ca.* 2-3 mg of solid sample homogenized with ca. 90–100 mg of anhydrous KBr). Data acquisition was conducted under positive pressure of dinitrogen gas.

NMR Kinetics. ^{19}F NMR kinetic measurements were performed on a 500 MHz spectrometer with silanized J. Young NMR tubes. Sample preparation, including addition of an internal standard, was conducted in a liquid-nitrogen chilled cold well using volumetric syringes to ensure exact concentrations. Following sample preparation, the J. Young NMR tube was sealed, exported from the drybox, placed in a dry-ice acetone bath, and allowed to thaw immediately prior to array acquisition. Each measurement was conducted in triplicate with averages reported following starting material consumand error bars referring to a single standard deviation.

Optical Spectroscopy. UV/Visible/NIR spectra were recorded on a Varian Cary 50 UV/Vis spectrometer using air-free quartz cuvettes (0.10 mm path length) and a scan rate of 600 nm/min. Samples were prepared using serial dilution with volumetric glassware to obtain accurate concentrations. Extinction coefficients were determined from three concentrations per sample and were calculated by a linear regression fit of the absorbance vs. concentration data.

Electron Paramagnetic Resonance Spectroscopy. EPR spectra were obtained on a Bruker EleXsys E-500 CW-EPR spectrometer. Spectra containing *ca.* 5–10 mg sample were measured as frozen toluene glasses, frozen 2-methyltetrahydrofuran glasses, and/or in solution at a microwave power of 0.6325–2 mW. Effective g-values, g-strain (σ), and hyperfine coupling constants (A) were obtained from spectral simulations of $S = {}^{1}/_{2}$ systems using the program Easyspin.[6]

Electrochemistry. Cyclic voltammetry and differential pulse voltammetry measurements were performed with a CHI660d potentiostat using a three-electrode cell with a glassy carbon working electrode, a platinum wire as the counter electrode, and a $Ag/AgNO_3$ reference electrode. All of the potentials are referenced to the $[Cp_2Fe]^{+/0}$ couple. Saturated tetrabutylammonium hexafluorophosphate ($TBAPF_6$) solutions of 0.2 M in tetrahydrofuran under dinitrogen were prepared before each experiment. No background reaction upon addition of excess $TBAPF_6$ in tetrahydrofuran was observed for all complexes.

Combustion Analysis. Elemental analysis (%CHN) was conducted at Harvard University on a PerkinElmer 2400 Series II CHNS/O Analyzer using bulk recrystallized compounds. Samples

were prepared in a dinitrogen-filled drybox. In several cases, satisfactory elemental analyses were obtained by considering the presence of solvent molecules intercalated within the unit cell as ascertained by single-crystal X-ray diffraction and ^{1}H NMR spectroscopy.

Syntheses of Supporting Ligands and Transition Metal Complexes.

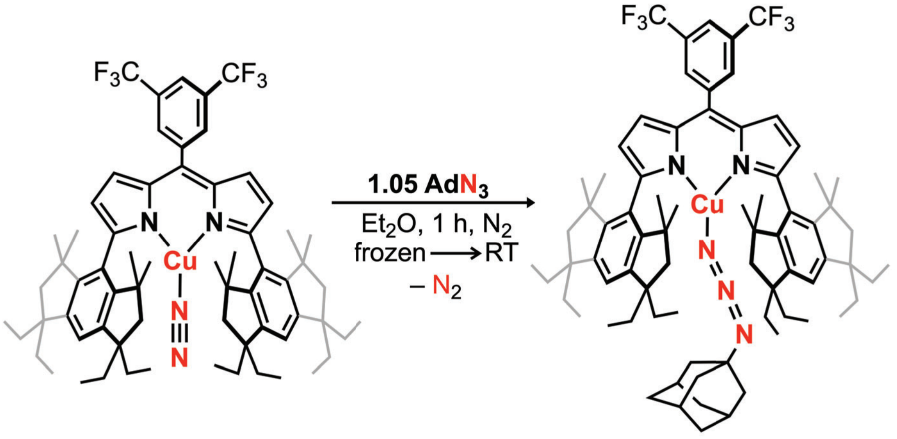

(**1**) (**2**)

(^{EMind}L)Cu(N_3Ad) (**2**). In a dinitrogen-filled drybox, crystalline (^{EMind}L)Cu(N_2) (**1**; 0.041 g, 0.038 mmol, 1.00 equiv.)[3] was dissolved in diethyl ether (0.5 mL) and frozen in a liquid-nitrogen chilled cold well. Upon thawing, 1-azidoadamantane (AdN_3; 0.007 g, 0.039 mmol, 1.05 equiv.) in chilled diethyl ether (0.5 mL) was added dropwise, resulting in a slight reddening of the solution and the gradual precipitation of an insoluble orange solid. After stirring for 1 h, the suspension was filtered through Celite. The filtrate was discarded, and the remaining solids were dissolved in 2-methyltetrahydrofuran to afford (^{EMind}L)Cu(N_3Ad) (**2**) as a bright orange solid (0.028 g, 60 %). Crystals suitable for single-crystal X-ray diffraction were obtained by allowing a concentrated solution of **2** in a 3:1 diethyl ether/2-methyltetrahydrofuran mixture to stand at –35 ºC over 72 h. ^{1}H NMR (500 MHz, C_6D_6): δ 7.89 (s, 2H, fluorinated aryl C–*H*), 7.84 (s, 1H, fluorinated aryl C–*H*), 6.97 (s, 2H, EMind aryl C–*H*), 6.46 (d, J = 3.9 Hz, 2H, dipyrrin C–H), 6.23 (d, J = 4.1 Hz, 2H, dipyrrin C–H), 1.76 – 1.92 (12H, overlapping singlet 8*H* from EMind aliphatic C–H and multiplet 4*H* from EMind ethyl C– H), 1.59 – 1.76 (8H, overlapping multiplet from EMind ethyl C–*H*), 1.56 (s, 6H, adamantyl C–*H*), 1.36 (m, 6H, adamantyl C–*H*), 1.31 (s, 24*H*, EMind methyl C–*H*), 1.20 – 1.28 (m, 3H, adamantyl C–*H*), 0.82 – 0.98 (24H, overlapping triplet from EMind ethyl C–H). ^{13}C[5] NMR (100 MHz, C_6D_6): δ 159.47, 148.47, 142.95, 139.31, 131.46, 130.82, 130.57, 130.24, 129.93, 124.85, 124.79, 122.14, 121.40, 120.17, 53.96, 47.80, 43.98, 41.28, 36.08, 35.56, 34.67, 34.58, 32.17, 31.67, 29.72, 29.12, 25.33, 22.76, 20.58, 18.64, 14.06, 11.37, 9.15. ^{19}F NMR (470 MHz, C_6D_6): δ –62.35 (s, fluorinated aryl C*F*$_3$). IR (KBr): ν_{NNN} = 2134, 2107 cm^{-1}; ν_{NAd} = 1241 cm^{-1}). Anal. Calc. for $C_{75}H_{96}CuF_6N_5$: C 72.35, H 7.77, N 5.62; Found: C 72.06, H 7.67, N 5.48.

Note: The corresponding ^{15}N isotopologue (^{EMind}L)Cu($^{14}N^{14}N^{15}N$Ad) was prepared using 1-(azido-1-^{15}*N*)-adamantane. IR (KBr): $\nu_{^{15}NNN}$ = 2119, 2080 cm^{-1}; $\nu_{^{15}NAd}$ = 1227 cm^{-1}). Using a diatomic harmonic oscillator approximation, the anticipated shifts are $\nu_{^{15}NNN}$ = 2098, 2072 cm^{-1}; $\nu_{^{15}NAd}$ = 1222 cm^{-1}.

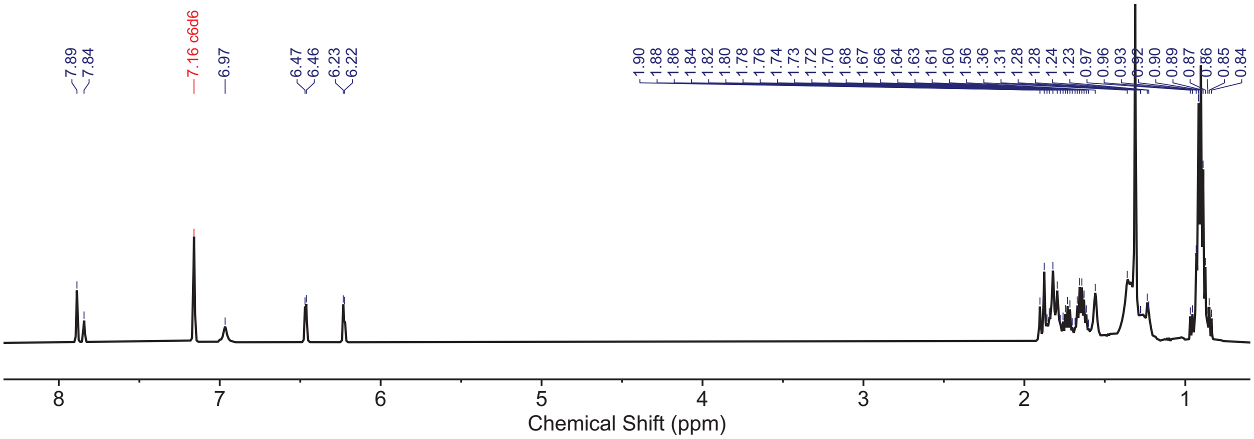

Figure S1. ^{1}H NMR spectrum of (^{EMind}L)Cu(N_3Ad) (**2**), (500 MHz, C_6D_6).

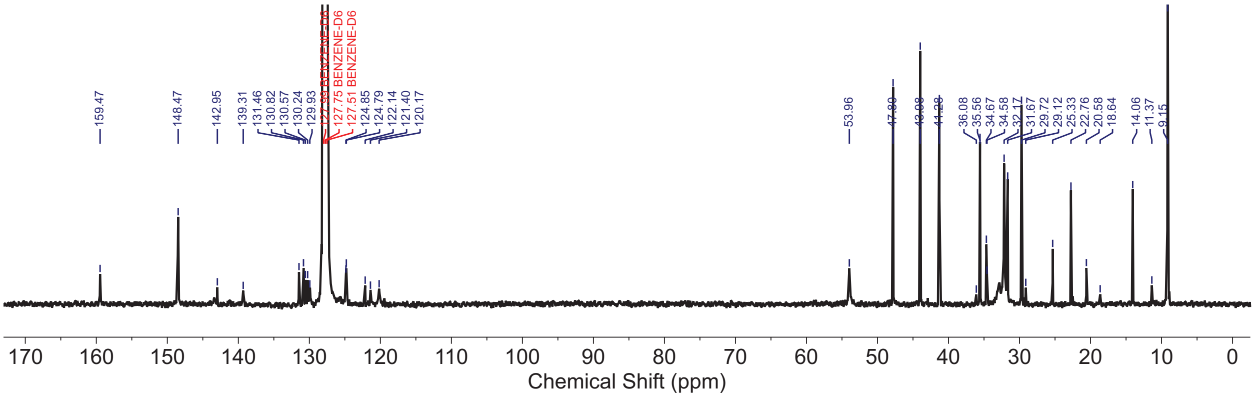

Figure S2. ^{13}C^{5} NMR spectrum of (^{EMind}L)Cu(N_3Ad) (**2**), (100 MHz, C_6D_6).

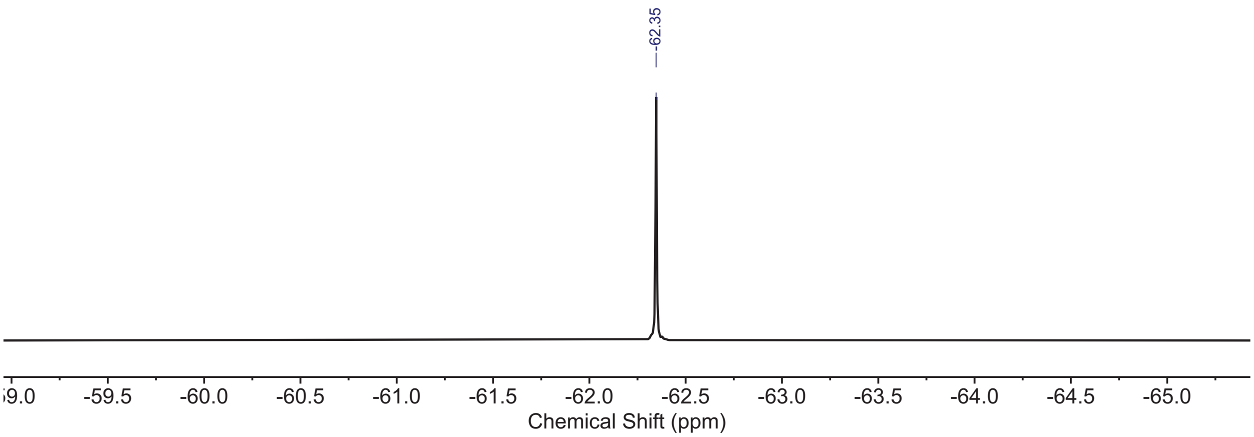

Figure S3. ^{19}F NMR spectrum of (^{EMind}L)Cu(N_3Ad) (**2**), (470 MHz, C_6D_6).

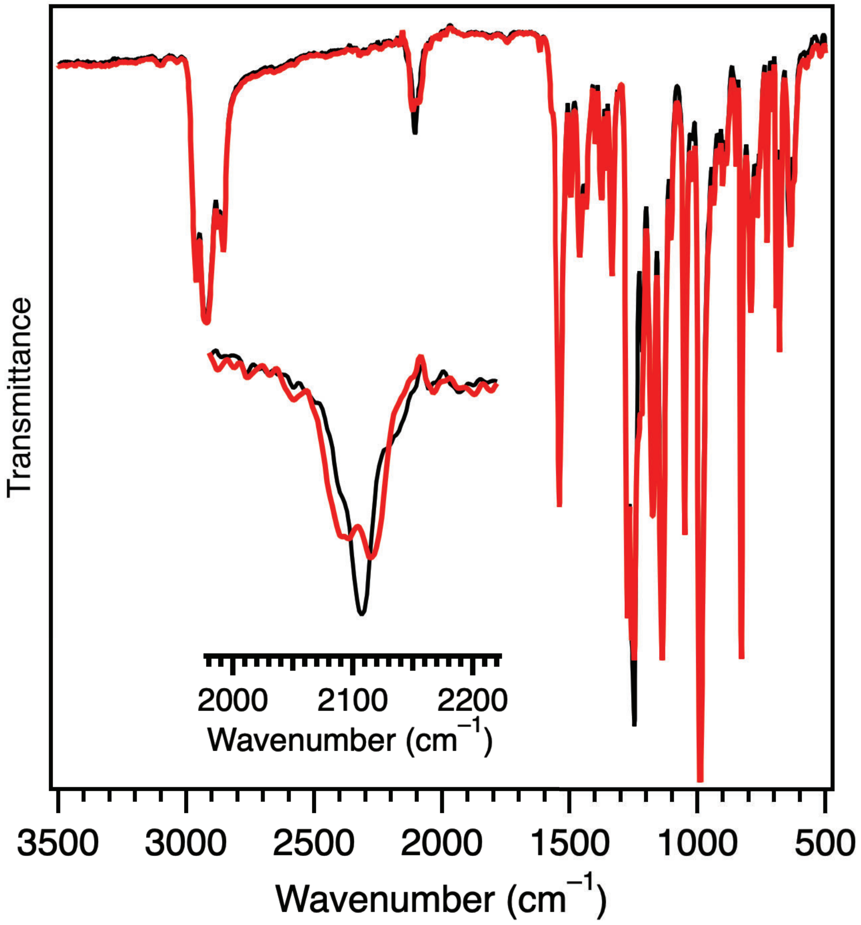

Figure S4. Infrared spectra overlay of (^{EMind}L)Cu(N_3Ad) (**2**, *red*) and (^{EMind}L)Cu(NN^{15}NAd) (**2–$^{15}N_3$Ad**, *black*); (*Inset*) Truncated IR spectra highlighting differences in the azide frequency frequency as a function of the N_α isotope.

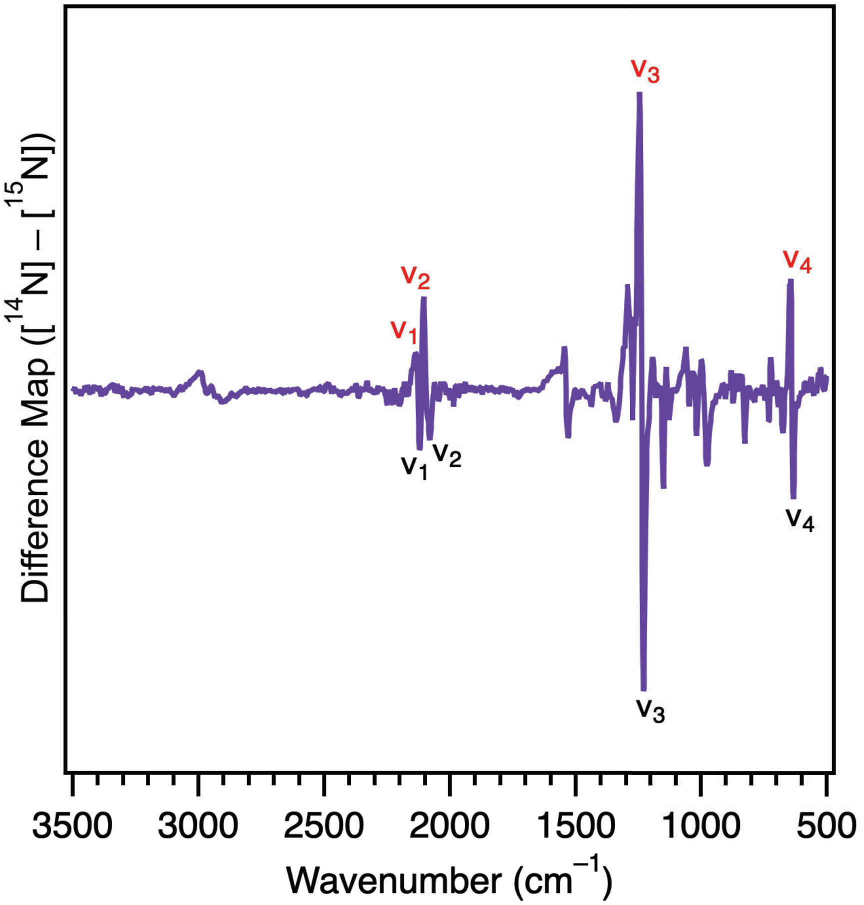

Figure S5. Difference infrared spectrum of (^{EMind}L)Cu(N_3Ad) (**2**, *red*) and (^{EMind}L)Cu(NN^{15}NAd) (**2–^{15}N$_3$Ad**, *black*), revealing stretching frequencies sensitive to the substitution of the N_α isotope. The ν_1 and ν_2 frequencies are attributed to the azide stretch, and the ν_3 frequency is attributed to the N–C_{Ad} stretch. The low-energy ν_4 frequency is attributed to an azide wagging motion.

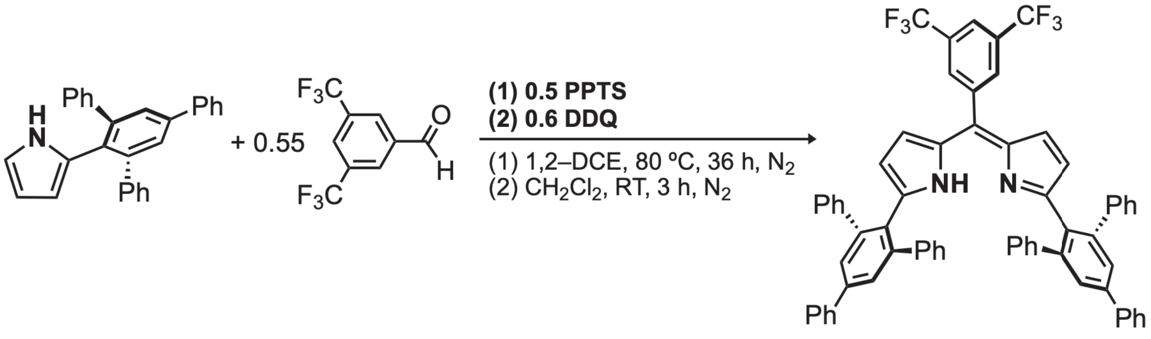

(^{ArF}LH)

(^{ArF}L)H. Adapting from a literature procedure,[7] in a dinitrogen-filled drybox, to a Schlenk tube was charged with solid 2-(2,4,6-triphenylphenyl)-1*H*-pyrrole (1.63 g, 4.39 mmol, 1.00 equiv.) was added distilled 3,5-bis(trifluoromethyl)benzaldehyde (0.59 g, 2.43 mmol, 0.55 equiv.) and pyridinium p-toluenesulfonate (PPTS; 0.55 g, 2.19 mmol, 0.50 equiv.) in 1,2-dichloroethane (10 mL), resulting in a light pink suspension. The Schlenk tube was sealed, removed from the drybox, and heated to 80 ºC for 36 h, accompanied by homogenization and a color change from faint pink to deep purple. The mixture was cooled to room temperature, exposed to air, and filtered through a plug of silica on top of a coarse porosity frit, followed by extensive rinsing with dichloromethane (200 mL) until the red eluent became colorless. Solvent was removed in vacuo to afford a flocculent pink solid, which was subsequently dissolved in anhydrous dichloromethane (150 mL). Under an atmosphere of nitrogen, 2,3-dichloro-5,6-dicyano-1,4-benzoquinone (DDQ; 0.60 g, 2.64 mmol, 0.6 equiv.) was added as a solid in a single portion, resulting in an immediate color change to dark purple. After 3 h, addition of excess triethylamine (3.1 mL, 5.0 equiv.) afforded a rapid color change to bright red. Following removal of solvent *in vacuo*, the residual solid was triturated twice in hexanes, followed by elution through a plug of silica with toluene until the eluent became colorless. Following removal of solvent *in vacuo*, the residual solids were triturated twice in hexanes to remove residual toluene. The residual solids were suspended in minimal pentane, held at –20 ºC for 1 h, and then filtered over a coarse porosity frit. The solids were rinsed with boiling methanol (2 x 20 mL) and additional pentane (10 mL) to afford (^{ArF}L)H (1.00 g, 47 %) as a bright red powder, which was lyophilized with anhydrous benzene in the drybox prior to subsequent metallation with mesitylcopper to remove trace water. ^{1}H NMR (500 MHz, C_6D_6): δ 12.14 (s, 1*H*, dipyrrin N–*H*), δ 7.73 (s, 1*H*, fluorinated aryl C–*H*), 7.64 (s, 4*H*, aryl C–*H*), 7.45 – 7.47 (m, 4*H*, aryl C–*H*), 7.41 (s, 2*H*, fluorinated aryl C–*H*), 7.31 – 7.32 (m, 4*H*, aryl C–*H*), 7.17 – 7.21 (m, 2*H*, aryl C–*H*), 7.12 – 7.15 (m, 8*H*, aryl C–*H*), 7.02 – 7.09 (m, 12*H*, aryl C–*H*), 5.88 (d, *J* = 4.2 Hz, 2*H*, dipyrrin C–*H*), 5.79 (dd, *J* = 4.2 Hz, 2*H*, dipyrrin C–*H*). ^{13}C{^{1}H} NMR (100 MHz, C_6D_6): δ 153.96, 143.23, 142.05, 141.59, 140.25, 140.17, 139.26, 134.90, 130.95, 130.79, 130.62, 129.48, 128.95, 128.69, 128.38, 128.26, 128.08, 127.75, 127.31, 127.17, 127.04, 125.97, 124.61, 122.32, 121.90. ^{19}F NMR (500 MHz, C_6D_6): δ –62.51 (s, fluorinated aryl C*F*$_3$). UV/vis (C_6H_6) λ_{max}/cm^{-1} (ε/M^{-1}cm^{-1}): 530 (19,500), 330 (19,300). HRMS (ESI^{+}): *m/z* Calc. 965.3325 $[C_{65}H_{42}F_6N + H]^+$, Found 965.3311 $[M + H]^+$.

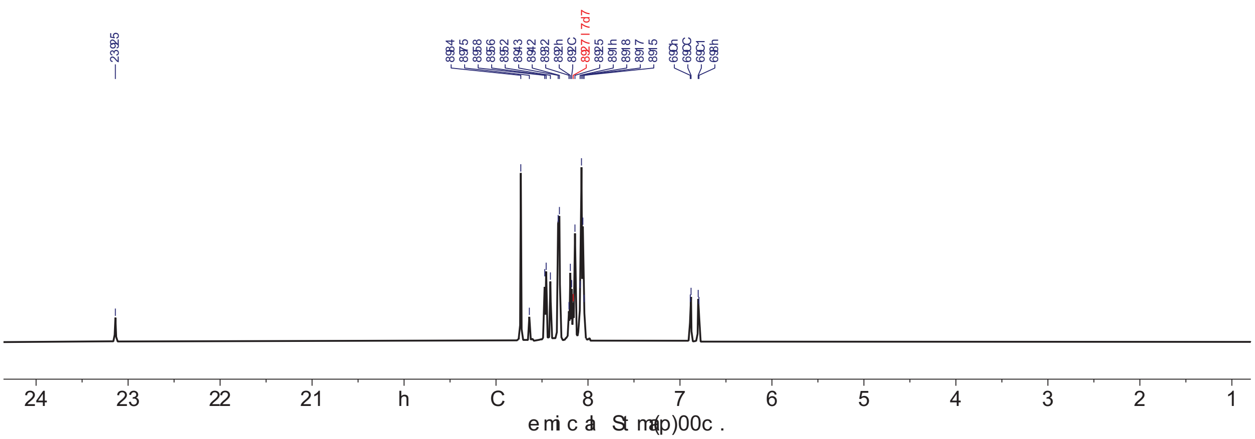

Figure S6. ^{1}H NMR spectrum (500 MHz, C_6D_6) of (^{ArF}L)H.

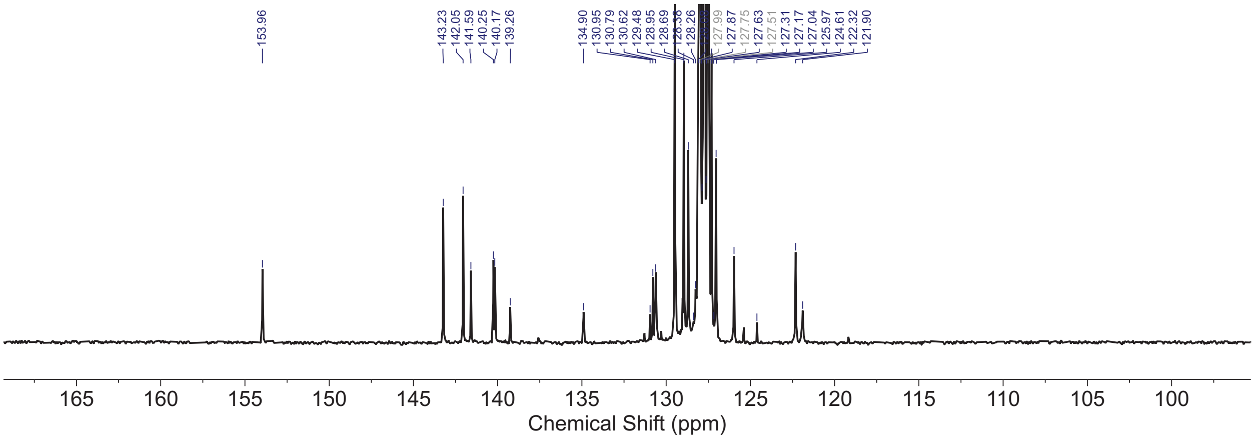

Figure S7. ^{13}C{^{1}H} NMR spectrum (100 MHz, C_6D_6) of (^{ArF}L)H.

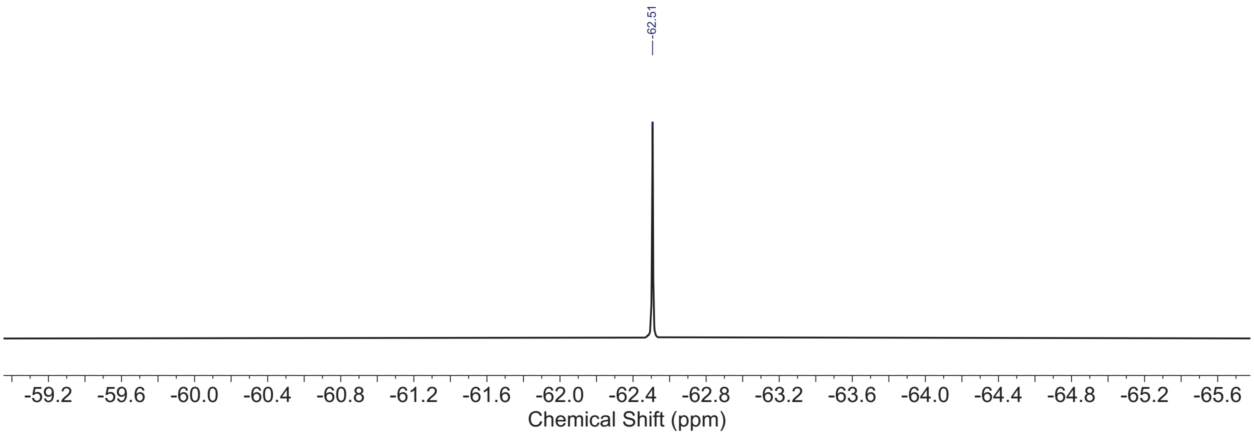

Figure S8. ^{19}F NMR spectrum (470 MHz, C_6D_6) of (^{ArF}L)H.

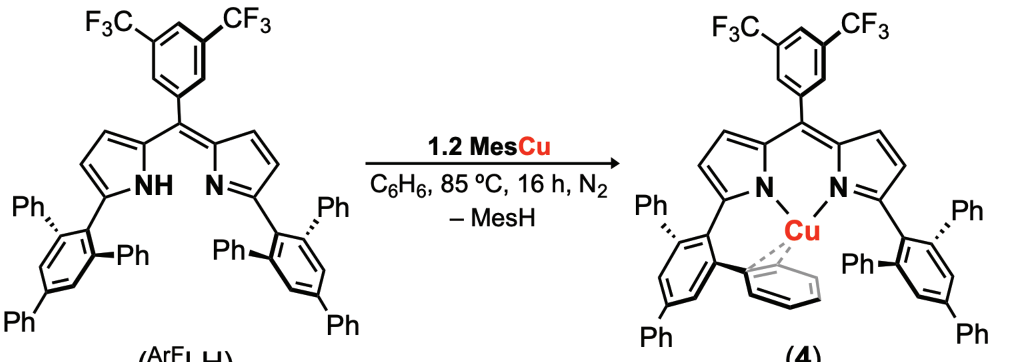

(^{ArF}L)Cu (**4**). In the drybox, to a Schlenk tube charged with a benzene solution (2 mL) of (^{ArF}L)H (0.254 g, 0.264 mmol, 1.0 equiv.) was added mesitylcopper (0.058 g, 0.316 mmol, 1.2 equiv.) in benzene (1 mL). The Schlenk tube was placed under partial vacuum, sealed, exported from the drybox, and heated to 85 ºC for 16 h, accompanied by a color change from bright red-pink to indigo. The Schlenk tube was re-imported into the drybox and lyophilized to afford a flocculent purple powder, which was suspended in minimal cold pentane and filtered over Celite. After rinsing with ample warm diethyl ether (3 x 5 mL) and minimal just-thawed benzene (*ca*. 2 mL) such that the filtrate became faint purple, the solids were eluted with ample boiling benzene (*ca*. 10 mL) and lyophilized to afford (^{ArF}L)Cu (**4**) (0.180 g, 67 %) as an indigo solid. Crystals suitable for single crystal X-ray diffraction were obtained by allowing diethyl ether to diffuse into a saturated benzene solution of **4** at room temperature over 48 h, followed by allowing the mixture to stand overnight at –35 ºC. ^{1}H NMR (500 MHz, C_6D_6): δ 7.69 (s, 1*H*, fluorinated aryl C–*H*), 7.68 (s, 4*H,* aryl C–*H*), 7.49 – 7.53 (m, 4*H*, aryl C–*H*), 7.44 (s, 2*H*, fluorinated aryl C–*H*), 7.22 – 7.28 (m, 4*H*, aryl C–*H*), 7.18 – 7.21 (m, 2*H*, aryl C–*H*), 7.11 – 7.14 (m, 8 *H*, aryl C–*H*), 6.97 – 7.01 (m, 8*H*, aryl C–*H*), 6.91 – 6.96 (m, 4*H*, aryl C–*H*), 5.94 (dd, *J* = 4.2, 1.1 Hz, 2*H*, dipyrrin C–*H*), 5.88 (dd, *J* = 4.2, 1.1 Hz, 2*H*, dipyrrin C–*H*). ^{13}C{^{1}H} NMR (125 MHz, C_6D_6): δ 156.76, 142.61, 141.84, 141.40, 140.86, 140.41, 140.09, 136.65, 133.21, 130.28, 129.52, 128.89, 128.46, 128.09, 127.95, 127.77, 127.70, 127.57, 127.12, 126.03, 124.94, 122.14. ^{19}F NMR (470 MHz, C_6D_6): –62.51 (s, fluorinated aryl C*F*$_3$). UV/vis (C_6H_6) λ_{max}/cm^{-1} (ε/M^{-1}cm^{-1}): 590 (55,00), 540 (22,300), 430 (2,700), 345 (30,600). Anal. Calc. for $C_{65}H_{41}CuF_6N_2$: C 75.98, H 4.02, N 2.73; Found: C 76.27, H 3.95, N 2.72.

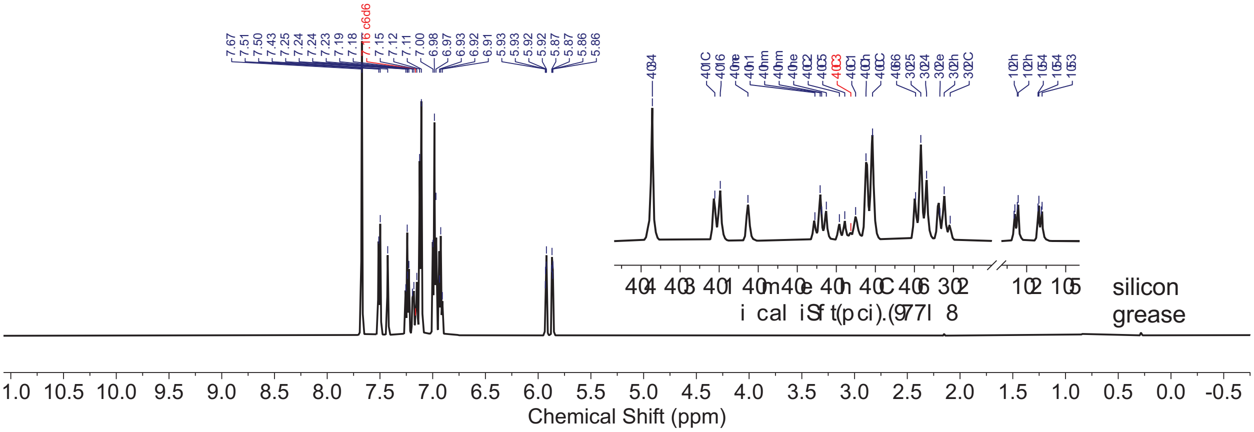

Figure S9. ^{1}H NMR spectrum (500 MHz, C_6D_6) of (^{ArF}L)Cu (**4**); (Inset) Aryl C–*H* resonances and dipyrrin C–*H* resonances.

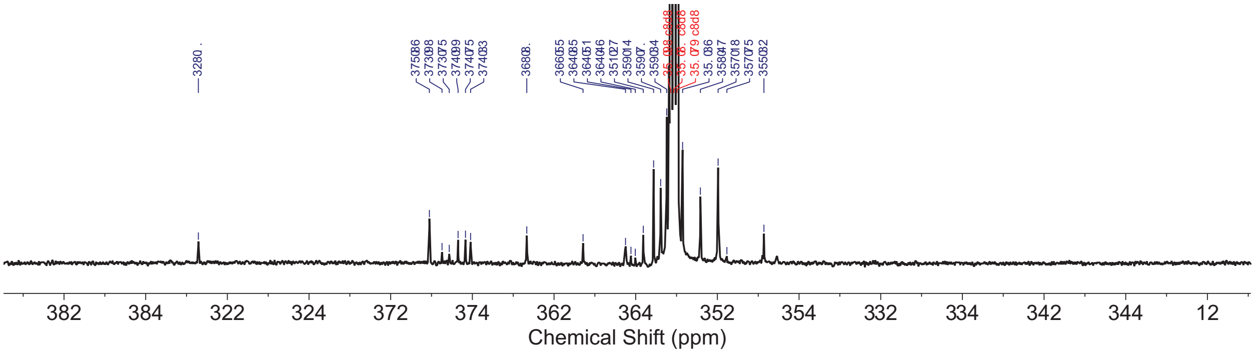

Figure S10. ^{13}C{^{1}H} NMR spectrum (125 MHz, C_6D_6) of (^{ArF}L)Cu (**4**).

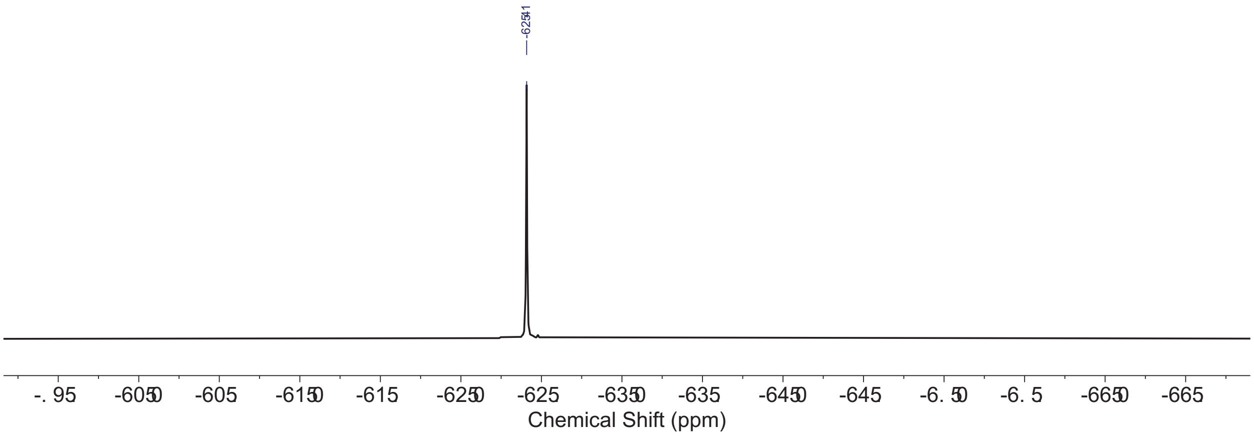

Figure S11. ^{19}F NMR spectrum (470 MHz, C_6D_6) of (^{ArF}L)Cu (**4**).

Additional Organic Syntheses.

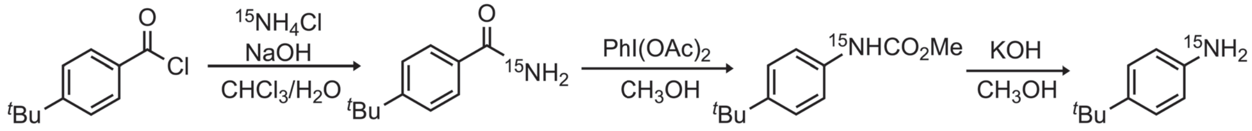

4-(*tert*-butyl)benzamide-^{15}*N*. The title compound was prepared according to a literature procedure,[8] employing commercial ^{15}N-ammonium chloride (99 % isotopically enriched).

Methyl (4-(tert-butyl)phenyl)carbamate-^{15}*N*. Under air, 4-(*tert*-butyl)benzamide-^{15}N (2.21 g, 0.012 mol, 1.00 equiv.) was dissolved in methanol (50 mL), followed by addition of potassium hydroxide (1.75 g, 0.031 mol, 2.50 equiv.). The solution was cooled to 0 ºC, followed by addition of stoichiometric diacetoxyiodobenzene ($PhI(OAc)_2$; 4.14 g, 0.012 mol, 1.00 equiv.) in one portion with rapid stirring.[9] After 14 h, solvent was removed *in vacuo*, with subsequent aqueous workup and extraction with CH_2Cl_2, followed by desiccation and purification by column chromatography (silica, 95:5 hexanes/ethyl acetate). Following partial removal of solvent, pentane addition precipitated a crystalline white solid which was collected by filtration and rinsed with pentane (*ca.* 40 mL) to afford the title compound (1.95 g, 76 %) with characterization akin to literature reports.[10]

4-(tert-butyl)aniline-^{15}*N*. Under N_2, methyl (4-(tert-butyl)phenyl)carbamate-^{15}N (2.00 g, 0.010 mol, 1.00 equiv.) was dissolved in methanol (30 mL), followed by addition of potassium hydroxide (*ca.* 10 equiv.) in water (10 mL), resulting in immediate precipitation of a white solid.[9] The mixture was heated to reflux for 4 h, followed by extraction with hexanes and desiccation to afford the title compound as a red oil in quantitative yield, which was employed without further purification.

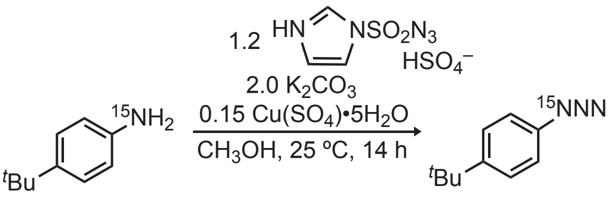

1-(azido-1-^{15}*N*)-4-(*tert*-butyl)benzene. Under air, 4-(tert-butyl)aniline-^{15}N (1.38 g, 0.009 mol, 1.00 equiv.) was dissolved in methanol (20 mL), followed by addition of imidazole-1-sulfonyl azide hydrogen sulfate (2.99 g, 0.011 mol, 1.20 equiv.),[11] copper sulfate pentahydrate (0.35 g, 0.001 mol, 0.15 equiv.) and potassium carbonate (2.54 g, 0.018 mol, 2.00 equiv.). The thick green slurry was stirred for 14 h with a subtle darkening of the solution. Solvent was removed *in vacuo*, with subsequent aqueous workup and extraction with hexanes, followed by desiccation and purification by column chromatography (silica, hexanes) to afford the title complex as a light-yellow oil (0.63 g, 41 %) with NMR spectroscopy characterization similar to the ^{14}N-parent analog. HRMS (ESI$^+$) *m/z* Calc. 199.0972 $[C_{10}H_{13}N_2{}^{15}N + Na]^+$, Found 199.0963 $[M + Na]^+$.

IR ($^{14}N^{14}N^{14}N(4\text{–}{}^tBu)C_6H_4$, KBr): ν_{NNN} = 2127, 2088 cm^{-1}; $\nu_{C\text{–}N}$ = 1298 cm^{-1}.
IR ($^{14}N^{14}N^{15}N(4\text{–}{}^tBu)C_6H_4$, KBr): $\nu_{NN^{15}N}$ = 2110, 2066 cm^{-1}; $\nu_{C\text{–}{}^{15}N}$ = 1270 cm^{-1}.

Using a diatomic harmonic oscillator approximation, the anticipated shifts for the ^{15}N analog are $\nu_{^{15}NNN}$ = 2092, 2053 cm^{-1}; $\nu_{^{15}NAd}$ = 1276 cm^{-1}.

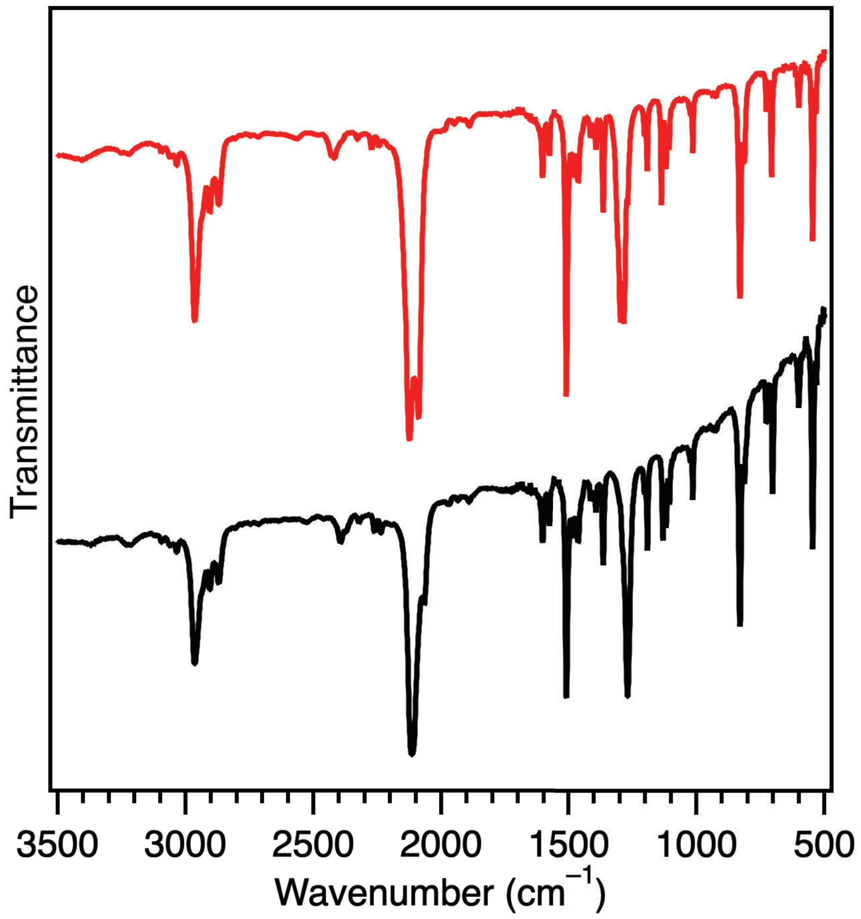

Figure S12. Infrared spectra overlay of NNN(4–^{t}Bu)C_6H_4 (top, *red*) and NN^{15}N(4–^{t}Bu)C_6H_4 (bottom, *black*).

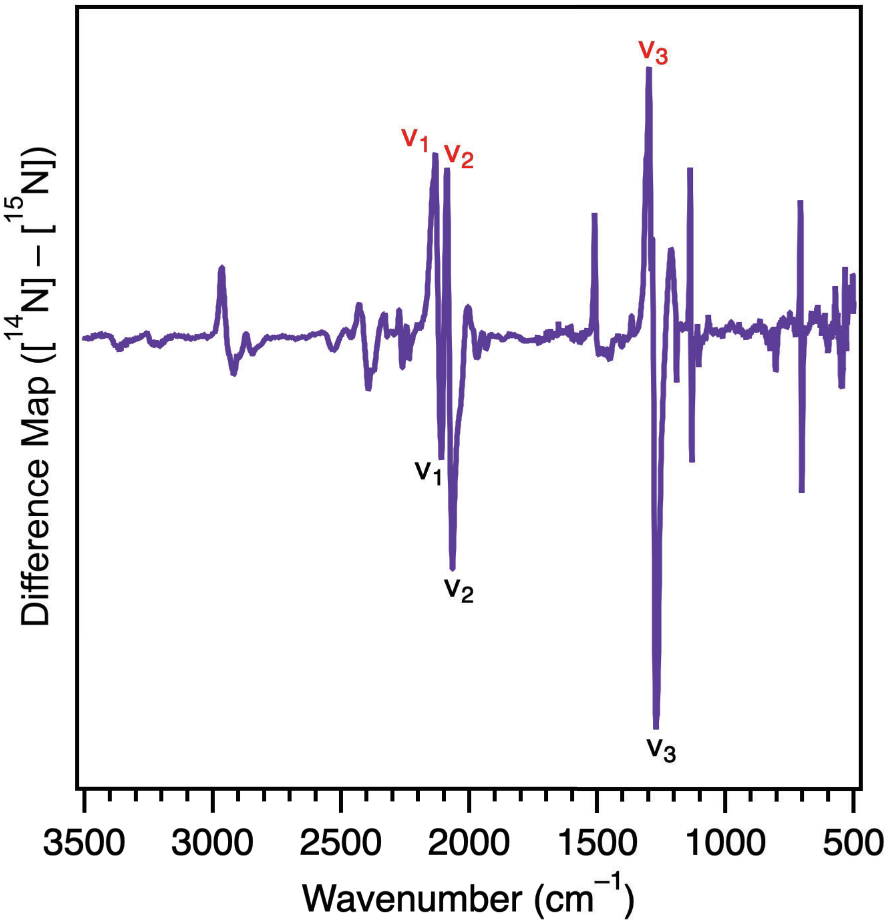

Figure S13. Difference infrared spectrum of NNN(4–^{t}Bu)C_6H_4 (*red*) and $NN^{15}N$(4–^{t}Bu)C_6H_4 (*black*). Azide stretching frequencies (ν_1, ν_2) and N–C_{Ar} stretching frequency (ν_3) are noted.

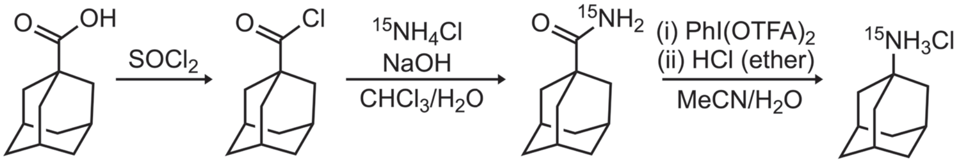

1-Adamantylamine-^{15}*N* Hydrochloride. Adapting from a literature procedure,[12] addition of deprotonated ^{15}*N*-ammonium chloride to *in situ* generated 1-adamantyl acetyl chloride (prepared by treatment of the corresponding carboxylic acid with neat thionyl chloride) afforded the corresponding benzamide, which was converted to the hydrochloride salt of the amine following Hoffman rearrangement using (bis(trifluoroacetoxy)iodo)benzene, acidification with ethereal HCl, and filtration. Characterization is in accord with literature reports.

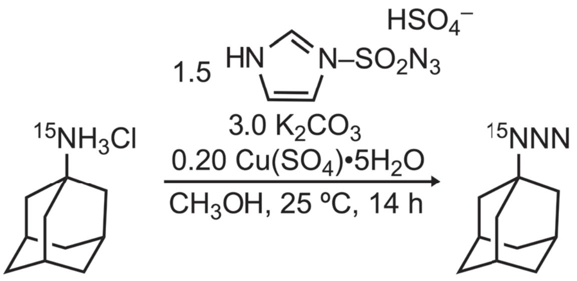

1-(Azido-1-^{15}*N*)-adamantane. Adapting from a literature procedure,[11] under air, 1-adamantylamine-^{15}*N* hydrochloride (1.38 g, 0.007 mol, 1.00 equiv.) was dissolved in methanol (20 mL), followed by addition of imidazole-1-sulfonyl azide hydrogen sulfate (2.38 g, 0.009 mol, 1.20 equiv.),[11] copper sulfate pentahydrate (0.35 g, 0.001 mol, 0.15 equiv.) and potassium carbonate (2.90 g, 0.021 mol, 3.00 equiv.). The thick green slurry was stirred for 14 h with a subtle darkening of the solution. Solvent was removed *in vacuo*, with subsequent aqueous workup and extraction with hexanes, followed by desiccation and purification by column chromatography (silica, hexanes) to afford the title complex as a white solid (0.69 g, 62 %) with ^{1}H NMR spectroscopy akin to the ^{14}*N*-parent analog.

Note: In contrast to the previous syntheses of the title compound with sodium ^{15}*N*-azide,[13] the employment of ^{15}*N*-ammonium chloride allows for the analogous preparation with complete ^{15}*N* enrichment of the α-nitrogen.

IR ($^{14}N^{14}N^{14}NAd$, KBr): ν_{NNN} = 2140, 2088 cm^{-1}; $\nu_{C\text{–}N}$ = 1254 cm^{-1}.
IR ($^{14}N^{14}N^{15}NAd$, KBr): $\nu_{NN^{15}N}$ = 2113, 2080 cm^{-1}; $\nu_{C\text{–}^{15}N}$ = 1229 cm^{-1}.

Using a diatomic harmonic oscillator approximation, the anticipated shifts for the ^{15}N analog are $\nu_{^{15}NNN}$ = 2104, 2053 cm^{-1}; $\nu_{^{15}NAd}$ = 1232 cm^{-1}

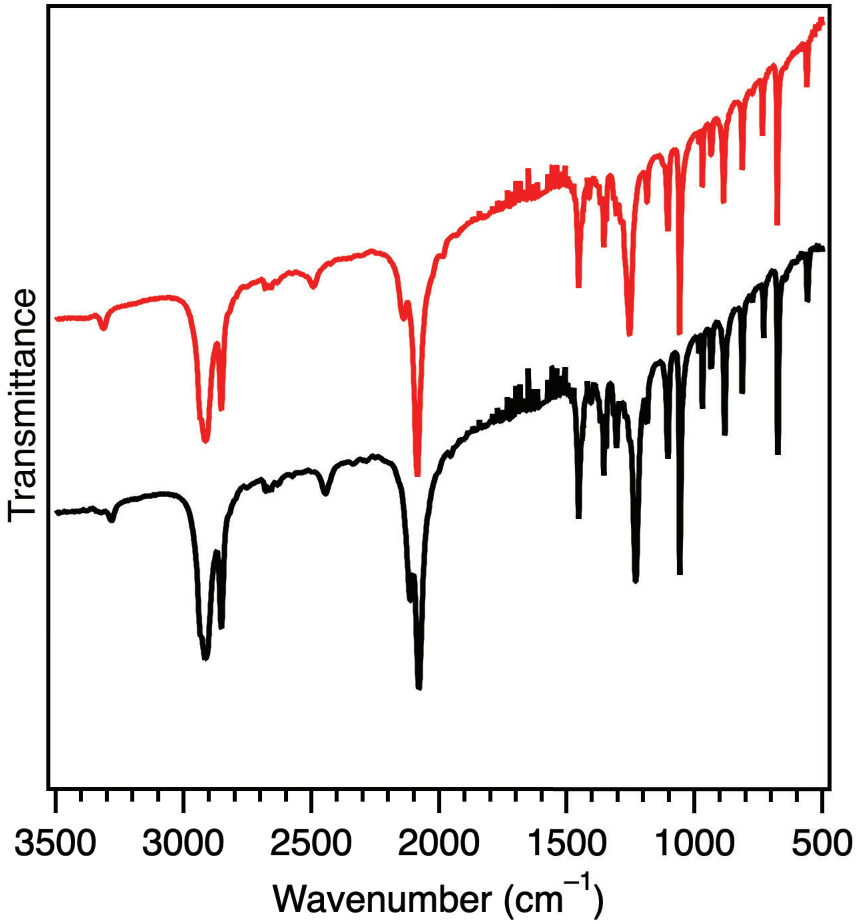

Figure S14. Infrared spectra overlay of NNNAd (top*, red*) and NN^{15}NAd (bottom, *black*).

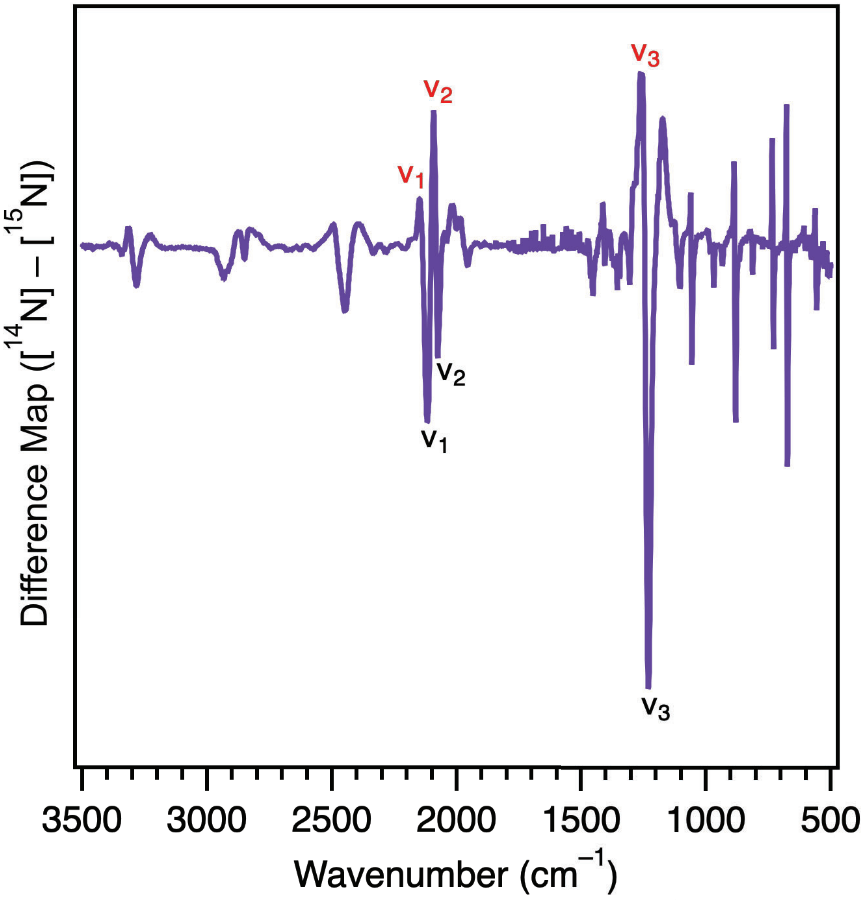

Figure S15. Difference infrared spectrum of NNNAd (*red*) and $NN^{15}NAd$ (*black*). Azide stretching frequencies (ν_1, ν_2) and N–C_{Ad} stretching frequency (ν_3) are noted.

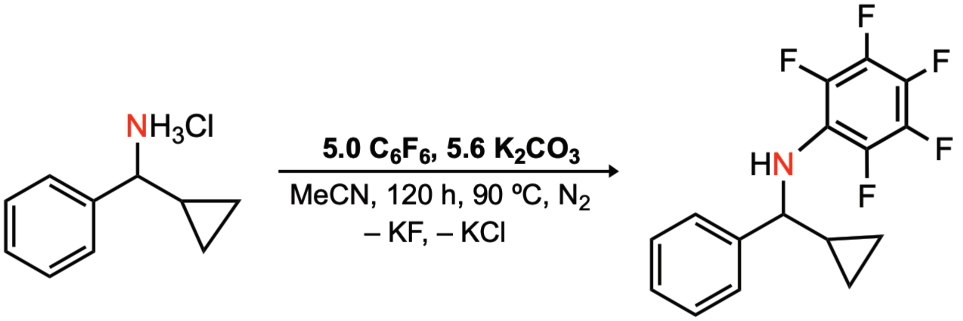

N-(cyclopropyl(phenyl)methyl)-2,3,4,5,6-pentafluoroaniline. Adapting from a literature procedure,[5] addition of racemic cyclopropyl(phenyl)methanamine•HCl (0.207 g, 1.13 mmol, 1.0 equiv.) and hexafluorobenzene (1.05 g, 5.64 mmol, 5.0 equiv.) to a Schlenk tube containing K_2CO_3 (0.87 g, 6.30 mmol, 5.6 equiv.) in anhydrous acetonitrile (15 mL) under positive dinitrogen pressure afforded the title compound as a yellow oil following heating at 90 ºC for five days, removal of solvent, and extraction with dichloromethane (0.150 g, 42 %). ^{1}H NMR (500 MHz, $CDCl_3$): δ 7.43 (d, *J* = 7.6 Hz, 2*H*, *ortho* aryl C–*H*), 7.36 (t, *J* = 7.5 Hz, 2*H*, *meta* aryl C–*H*), 7.28 (d, *J* = 7.2 Hz, 1*H*, *para* aryl C–*H*), 3.23 (d, *J* = 8.6 Hz, 1*H,* benzylic C–*H*), 1.65 (br, 1*H*, aniline N–*H*), 1.29, 1.12, 0.89, 0.62, 0.49, 0.33 (m, 5*H*, aliphatic cyclopropyl C–*H*). ^{13}C{^{1}H} NMR (125 MHz, $CDCl_3$): δ 145.71, 128.53, 128.36, 127.53, 126.98, 126.48, 126.40, 60.81, 19.87, 4.00, 3.16. ^{19}F NMR (470 MHz, $CDCl_3$): δ –158.27 (m, 2*F*, fluorinated aryl C–*F*), –164.67 (m, 2*F*, fluorinated aryl C–*F*), –170.75 (m, 1*F*, fluorinated aryl C–*F*). HRMS (ESI$^+$) *m/z* Calc. 314.0963 $[C_{15}H_{12}F_5N + H]^+$, Found 314.0963 $[M + H]^+$.

Note I: The crude synthesis of the title compound yields a trace diamagnetic byproduct by ^{1}H NMR spectroscopy (< 5 % presence by integration). Attempts to further purify the title complex by column chromatography or distillation have resulted in decomposition.

Note II: The title compound was accessed by addition of (cyclopropylmethyl)benzene and pentafluorophenyl azide to (^{ArF}L)Cu (**4**), resulting in selective amination at the secondary benzylic position.

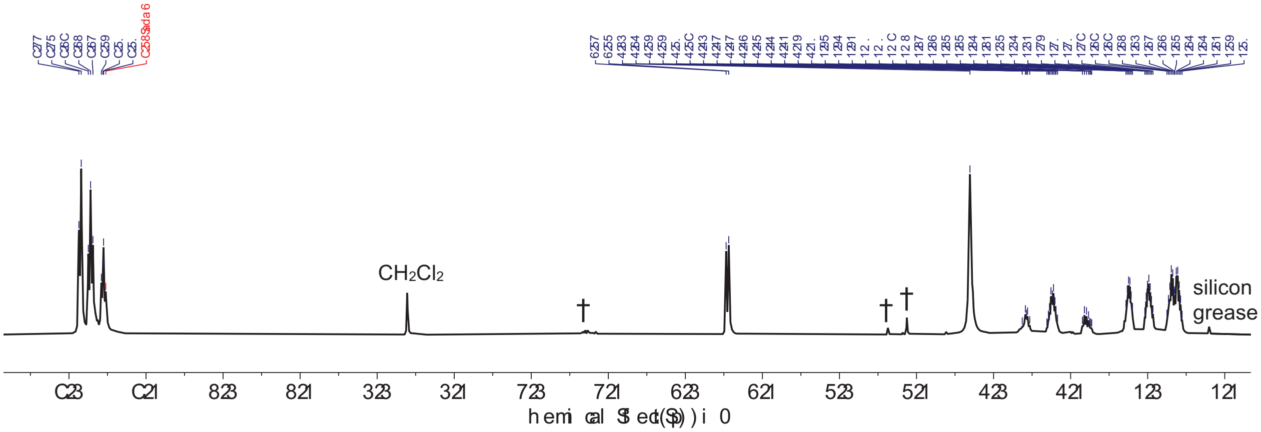

Figure S16. ^{1}H NMR spectrum (500 MHz, $CDCl_3$) of *N*-(cyclopropyl(phenyl)methyl)-2,3,4,5,6-pentafluoroaniline. Inseparable impurities are denoted with an obelisk (†).

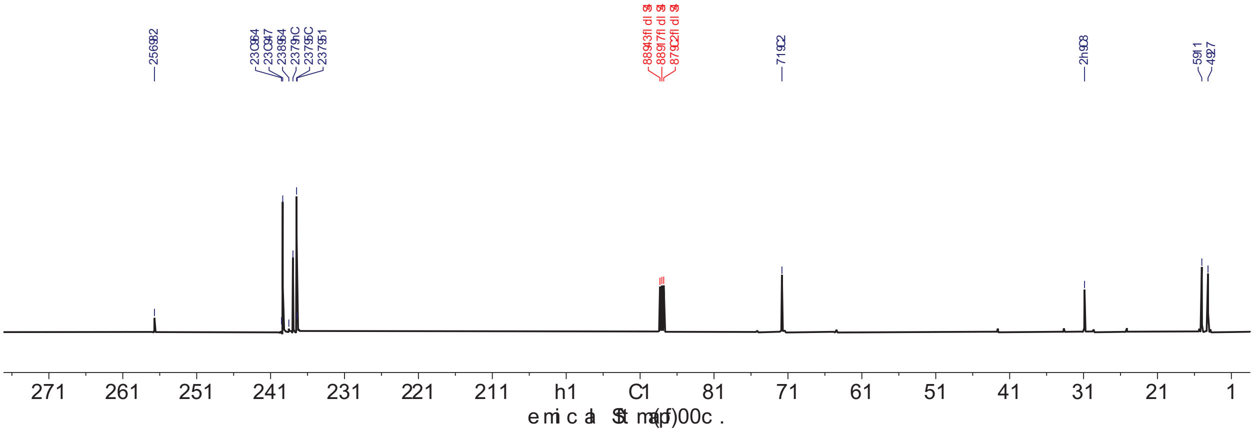

Figure S17. $^{13}C\{^{1}H\}$ NMR spectrum (125 MHz, $CDCl_3$) of *N*-(cyclopropyl(phenyl)methyl)-2,3,4,5,6-pentafluoroaniline.

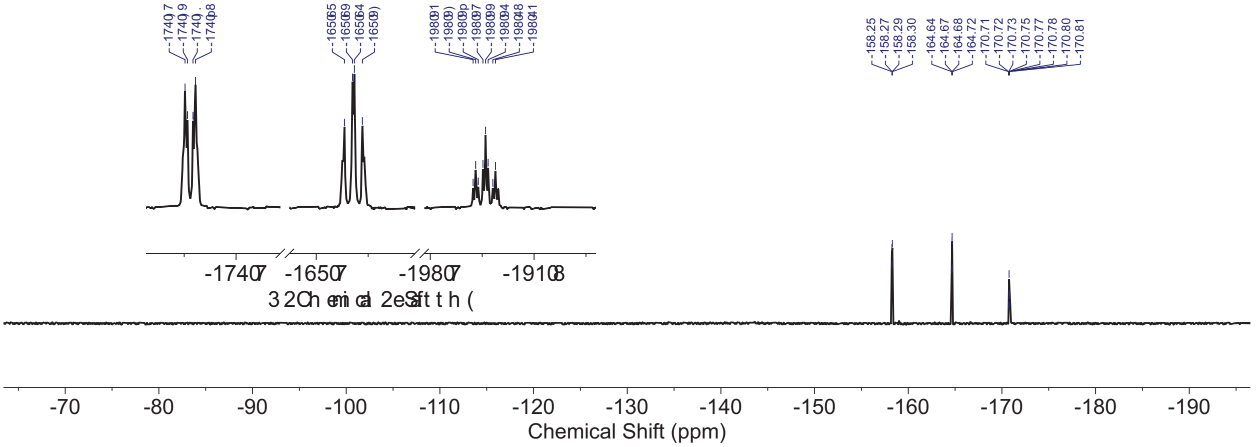

Figure S18. ^{19}F NMR spectrum (470 MHz, $CDCl_3$) of *N*-(cyclopropyl(phenyl)methyl)-2,3,4,5,6-pentafluoroaniline. Inset highlights aryl C–*F* resonances.

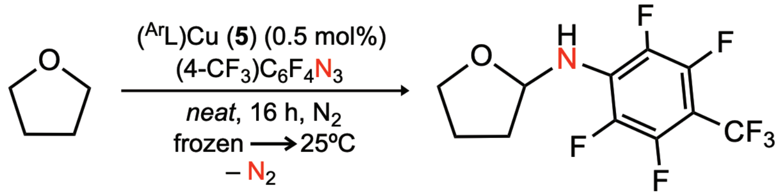

***N*-(2,3,5,6-tetrafluoro-4-(trifluoromethyl)phenyl)tetrahydrofuran-2-amine**. Treatment of (4-trifluoromethyl)tetrafluorophenyl azide[14] (0.200 g, 0.231 mmol, 200.0 equiv.) with (^{Ar}L)Cu (**5**; 0.001 g, 0.001 mmol, 1.0 equiv.) in neat tetrahydrofuran (5 mL) over 16 h afforded the title complex in quantitative yield, which was purified by removal of solvent *in vacuo* and extraction through Celite with cold pentane to remove **5**. ^{1}H NMR (400 MHz, C_6D_6): δ 5.32 (s, 1*H*, amine N–*H*), 3.93 (d, *J* = 9.2 Hz, 2*H*, α–ethereal secondary C–*H*), 3.39 (t, *J* = 7.0 Hz, 1*H*, α–ethereal tertiary C–*H*), 1.54 (dq, *J* = 14.0, 7.2 Hz, 1*H*, β–ethereal C–*H*), 1.21 (p, *J* = 7.3 Hz, 2*H*, β–ethereal C–*H*), 0.66 – 1.02 (m, 1*H,* β–ethereal C–*H*). ^{13}C{^{1}H} NMR (100 MHz, C_6D_6): δ 85.30, 66.44, 32.57, 24.24 (unresolved *C*–F). ^{19}F NMR (375 MHz, C_6D_6): δ –54.76 (t, *J* = 20.8 Hz, 3*F*, trifluoromethyl C–F), –143.47 (m, 2*F*, aryl C–*F*), –157.66 (d, *J* = 28.2 Hz, 2*F*, aryl C–*F*). HRMS (ESI^{+}) *m/z* Calc. 304.0567 [$C_{11}H_8F_7NO$ + H]$^{+}$, Found 304.0565 [M + H]$^{+}$.

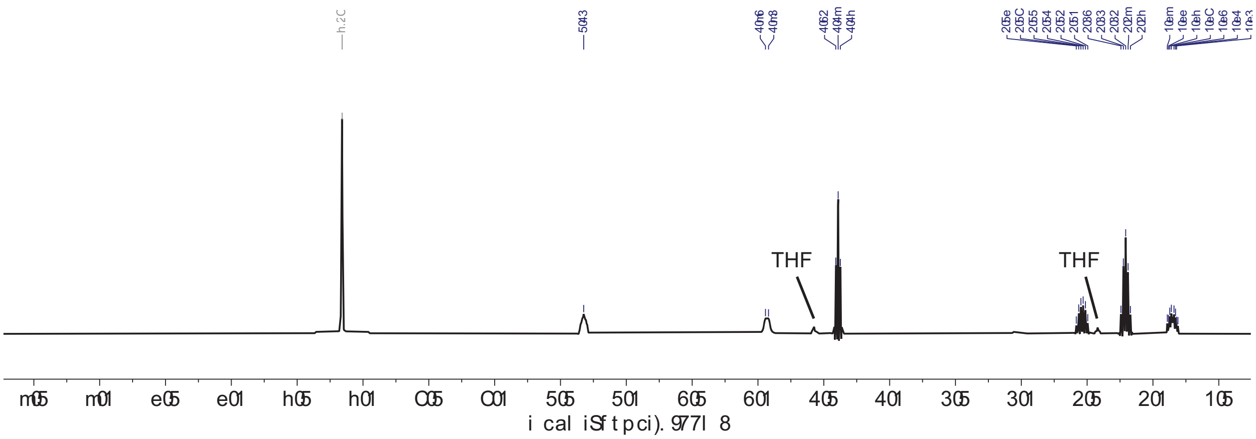

Figure S19. ^{1}H NMR spectrum (400 MHz, C_6D_6) of *N*-(2,3,5,6-tetrafluoro-4-(trifluoromethyl)phenyl)tetrahydrofuran-2-amine.

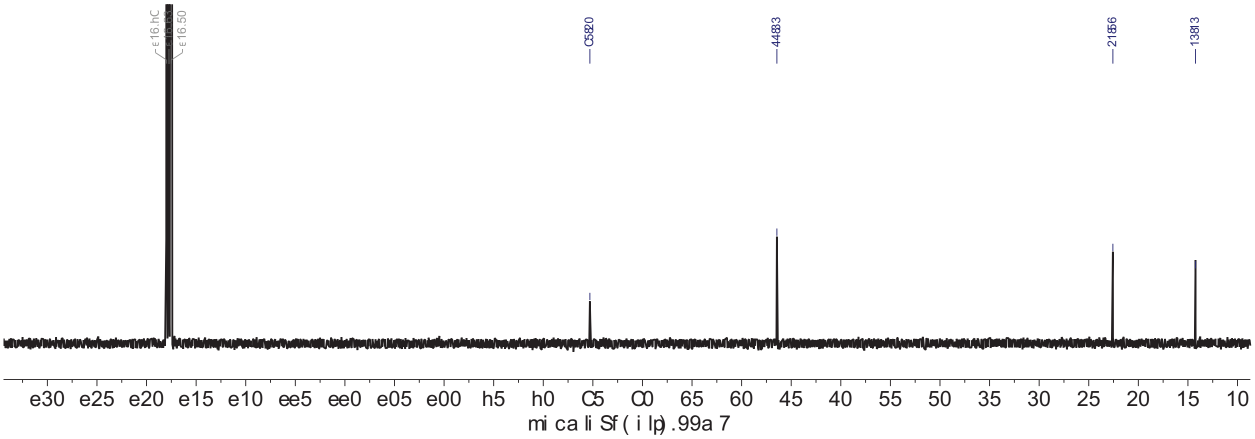

Figure S20. $^{13}C\{^{1}H\}$ NMR spectrum (100 MHz, C_6D_6) of *N*-(2,3,5,6-tetrafluoro-4-(trifluoromethyl)phenyl)tetrahydrofuran-2-amine.

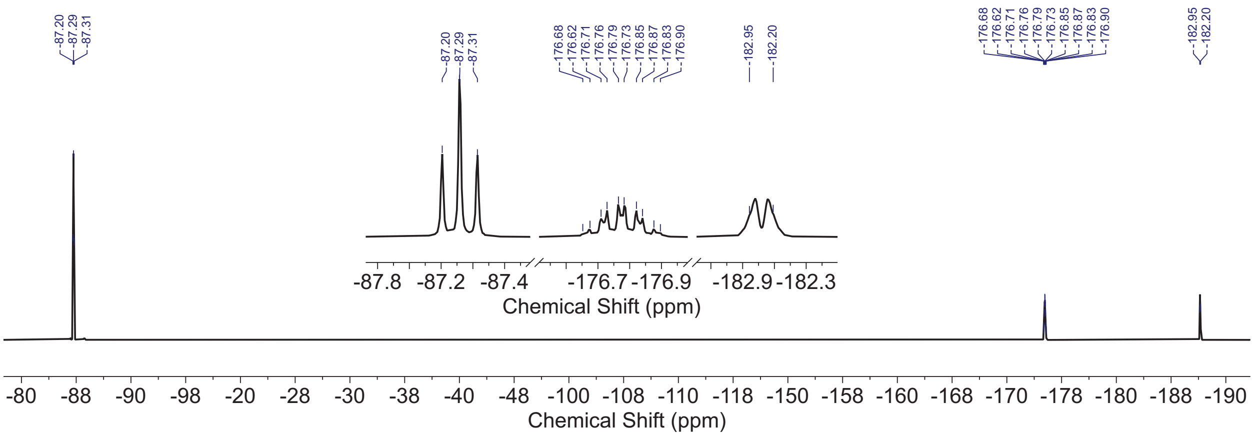

Figure S21. ^{19}F NMR spectrum (375 MHz, C_6D_6) of *N*-(2,3,5,6-tetrafluoro-4-(trifluoromethyl)phenyl)tetrahydrofuran-2-amine. Inset highlights aryl C–*F* resonances.

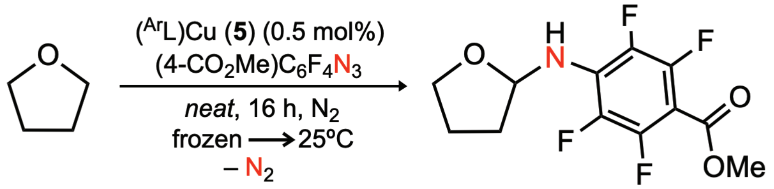

Methyl 2,3,5,6-tetrafluoro-4-((tetrahydrofuran-2-yl)amino)benzoate. Treatment of (4-methylbenzoate)tetrafluorophenyl azide[14] with (^{Ar}L)Cu (**5**; 1.0 mol%) in neat tetrahydrofuran (5 mL) over 16 h afforded the title complex, which was purified by removal of solvent *in vacuo* and extraction with cold pentane to remove **5** (*ca*. 95 % yield). ^{1}H NMR (400 MHz, C_6D_6): δ 5.39 (s, 1*H*, amine N–*H*), 4.04 (d, *J* = 9.6 Hz, 2*H*, α–ethereal secondary C–*H*), 3.43 (s, 3*H*, methoxy *C*–H), 3.42 (t, *J* = 7.0 Hz, 1*H*, α–ethereal tertiary C–*H*), 1.53 – 1.61 (m, 1*H*, β–ethereal C–*H*), 1.18 – 1.28 (m, 1*H*, β–ethereal C–*H*), 0.88 – 0.96 (m, 1*H*, β–ethereal C–*H*). ^{13}C{^{1}H} NMR (100 MHz, C_6D_6): δ 85.47, 66.38, 51.74, 32.55, 24.23 (unresolved *C*–F). ^{19}F NMR (375 MHz, C_6D_6): δ –158.38 (d, *J* = 19.9 Hz), –140.49 (d, *J* = 14.4 Hz). HRMS (ESI$^+$) *m/z* Calc. 294.0748 [$C_{12}H_{11}F_4NO_3$ + H]$^+$, Found 294.0745 [M + H]$^+$.

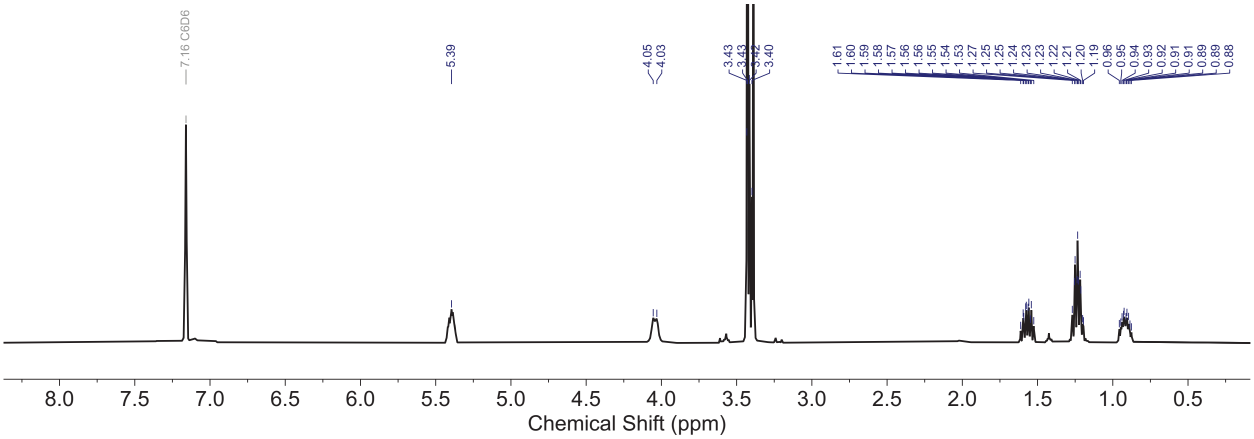

Figure S22. ¹H NMR spectrum (500 MHz, C_6D_6) of methyl 2,3,5,6-tetrafluoro-4-((tetrahydrofuran-2-yl)amino)benzoate.

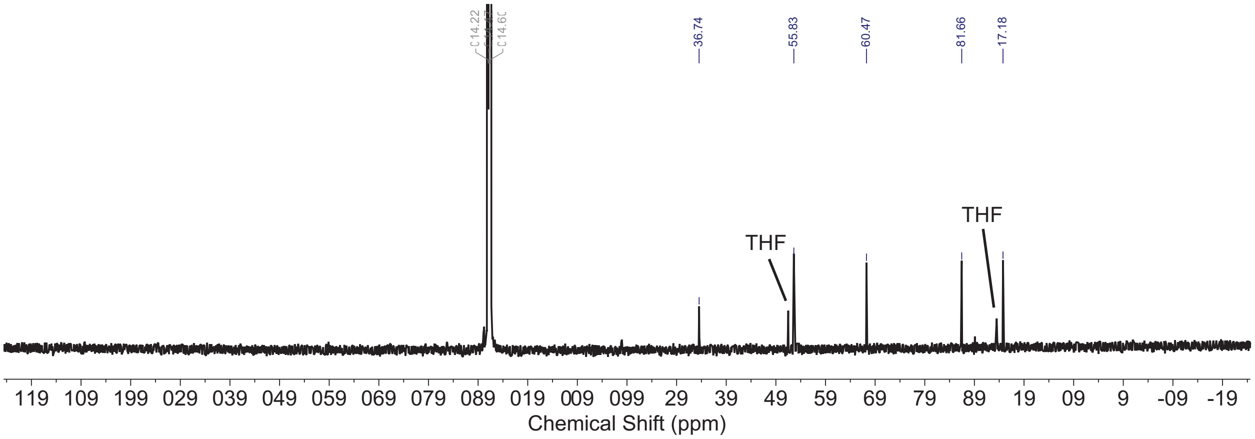

Figure S23. $^{13}C\{^1H\}$ NMR spectrum (100 MHz, C_6D_6) of methyl 2,3,5,6-tetrafluoro-4-((tetrahydrofuran-2-yl)amino)benzoate.

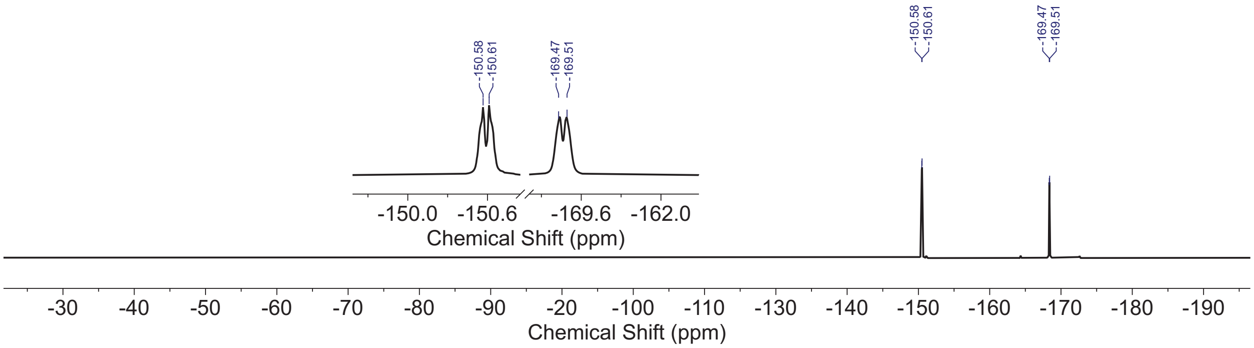

Figure S24. ¹⁹F NMR spectrum (375 MHz, C_6D_6) of methyl 2,3,5,6-tetrafluoro-4-((tetrahydrofuran-2-yl)amino)benzoate. Inset highlights aryl C–*F* resonances.

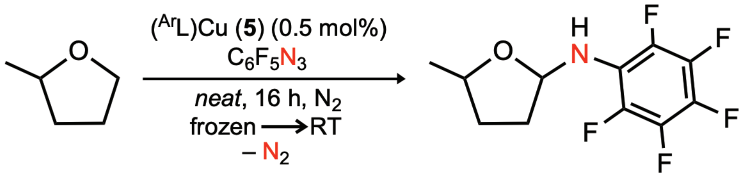

5-methyl-*N*-(perfluorophenyl)tetrahydrofuran-2-amine. Treatment of pentafluorophenyl azide with (^{Ar}L)Cu (**5**; 0.5 mol%) in neat 2-methyltetrahydrofuran (5 mL) over 16 h afforded the title complex, which was purified to afford an inseparable mixture of diastereomeric species by removal of solvent *in vacuo* and extraction with cold pentane to remove **5**. *Minor Diastereomer*: ^{1}H NMR (400 MHz, C_6D_6): δ 5.39 (s, 1*H*, amine N–*H*), 3.64 – 3.77 (m, 1*H*, overlapping α–ethereal tertiary C–*H*), 1.60 – 1.74 (m, 1*H,* β–ethereal C–*H*), 1.21 – 1.29 (m, 1*H,* β–ethereal C–*H*), 1.20 – 1.29 (m, 2*H,* overlapping β–ethereal C–*H*), 1.02 (d, *J* = 6.1 Hz, 3*H*, methyl C–*H*). ^{13}C{^{1}H} NMR (100 MHz, C_6D_6) δ 86.90, 75.11, 32.68, 31.43, 21.83 (unresolved *C*–F). ^{19}F NMR (400 MHz, C_6D_6): δ –139.56 (m, 2*F*, *ortho* aryl C–*F*), –151.43 (m, 2*F*, *para* aryl C–*F*), –158.43 (m, 2*F*, *meta* aryl C–*F*). *Major Diastereomer*: ^{1}H NMR (400 MHz, C_6D_6): δ 5.23 (s, 1*H*, amine N–*H*), 3.64 – 3.77 (m, 1*H*, overlapping α–ethereal tertiary C–*H*), 3.58 (d, *J* = 10.2 Hz, 1*H*, α–ethereal tertiary C–*H*), 1.76 – 1.85 (m, 1*H,* β–ethereal C–*H*), 1.34 – 1.49 (m, 2*H,* overlapping β–ethereal C–*H*), 1.00 (d, *J* = 6.1 Hz, 3*H*, methyl C–*H*). ^{13}C{^{1}H} NMR (100 MHz, C_6D_6): δ 86.63, 73.31, 33.12, 32.39, 20.42 (unresolved *C*–F). ^{19}F NMR (375 MHz, C_6D_6): δ –140.55 (m, 2*F*, *ortho* aryl C–*F*), –151.43 (m, 2*F*, *para* aryl C–*F*), –158.43 (m, 2*F*, *meta* aryl C–*F*). HRMS (ESI$^+$) *m/z* Calc. 268.0755 $[C_{11}H_{10}F_5NO + H]^+$, Found 268.0754 $[M + H]^+$.

Note: The diastereomeric ratio of aminated products using catalytic loadings of (^{ArF}L)Cu (**4**) is calculated as 2.2:1.0, whereas the diastereomeric ratio of aminated products using catalytic loadings of (^{Ar}L)Cu (**5**) is 1.0:1.7.

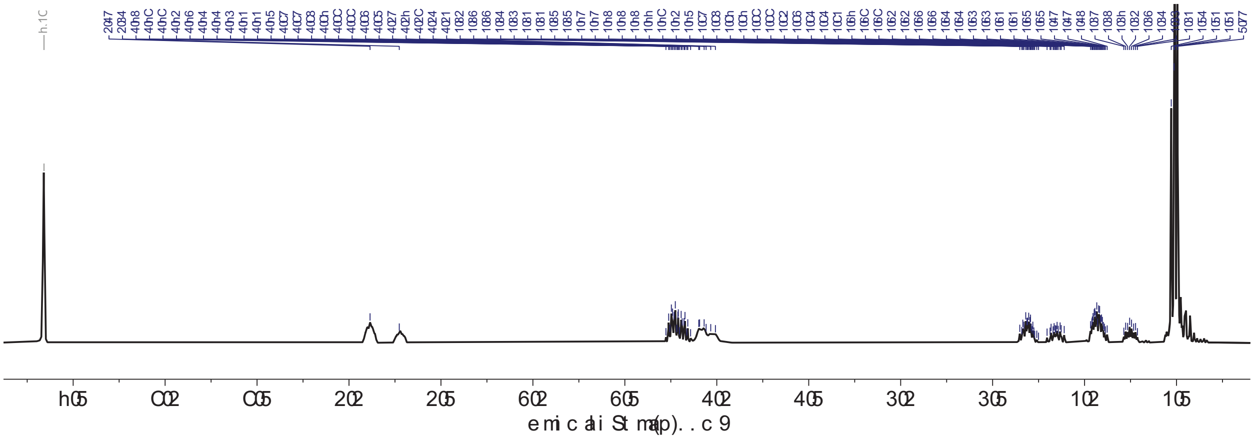

Figure S25. ^{1}H NMR spectrum (400 MHz, C_6D_6) of 5-methyl-*N*-(perfluorophenyl)tetrahydrofuran-2-amine.

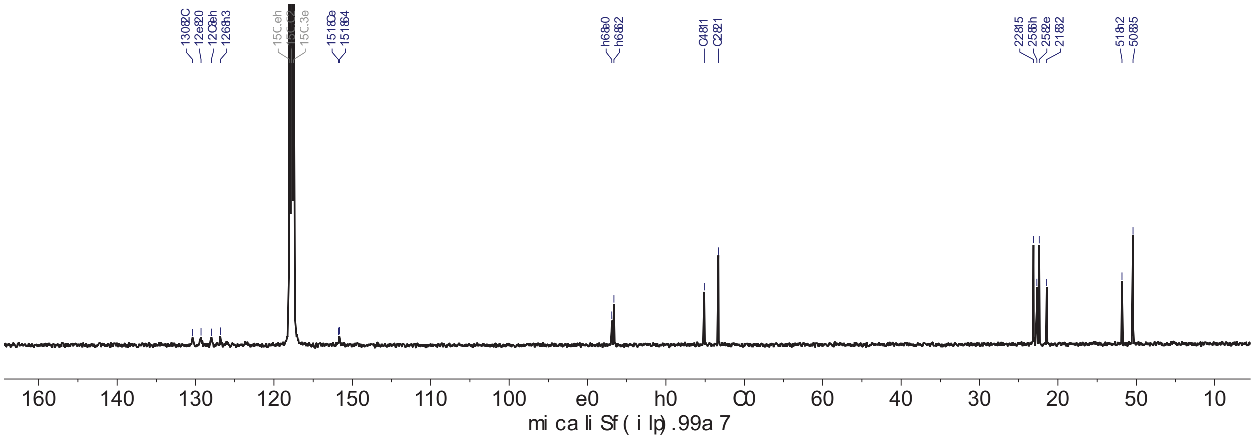

Figure S26. $^{13}C\{^{1}H\}$ NMR spectrum (100 MHz, C_6D_6) of 5-methyl-*N*-(perfluorophenyl) tetrahydrofuran-2-amine.

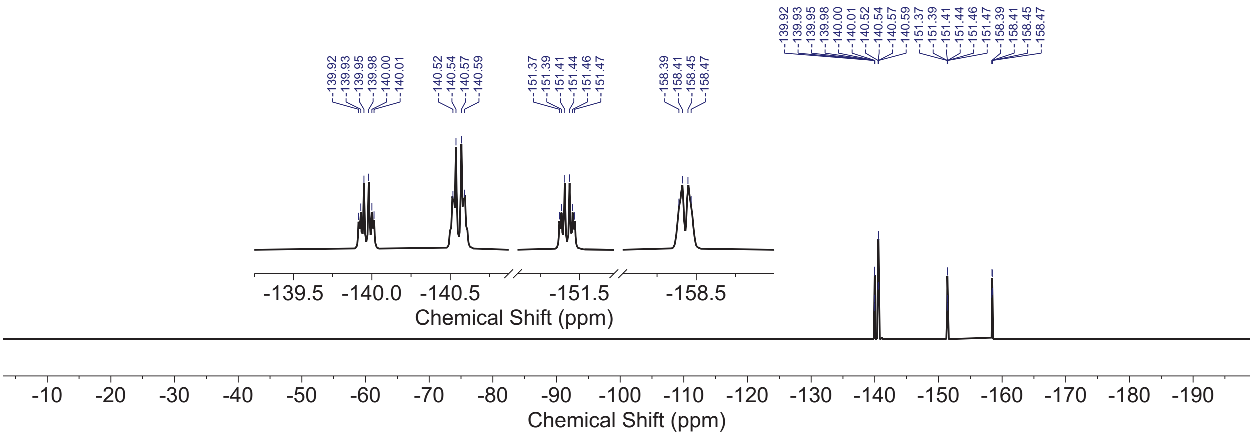

Figure S27. ^{19}F NMR spectrum (375 MHz, C_6D_6) of 5-methyl-*N*-(perfluorophenyl) tetrahydrofuran-2-amine. Inset highlights aryl C–*F* resonances.

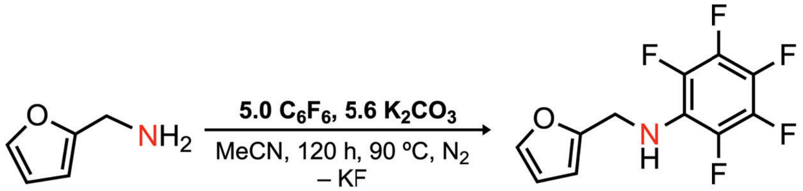

N-(perfluorophenyl)furan-2-amine. Adapting from a literature procedure,[5] addition of furfurylamine (0.300 g, 3.09 mmol, 1.0 equiv.) and hexafluorobenzene (2.89 g, 15.44 mmol, 5.0 equiv.) to a Schlenk tube containing K_2CO_3 (2.4 g, 17.3 mmol, 5.6 equiv.) in anhydrous acetonitrile (20 mL) under positive dinitrogen pressure afforded the title compound as a yellow oil following heating at 90 ºC for five days, removal of solvent, and extraction with dichloromethane (0.700 g, 87 %). ^{1}H NMR (400 MHz, C_6D_6): δ 6.97 (m, 1*H*, furanyl C–*H*), 5.95 (m, 1*H*, furanyl C–*H*), 5.87 (m, 1*H*, furanyl C–*H*), 3.96 (d, *J* = 6.8 Hz, 1*H*, benzylic C–*H*), 3.48 (br, 1*H*, aniline N–*H*). ^{13}C{^{1}H} NMR (100 MHz, C_6D_6): δ 152.07, 142.30, 139.42, 139.37, 139.33, 139.24, 137.09, 137.03, 136.95, 136.91, 136.87, 136.82, 136.77, 136.63, 135.20, 135.06, 135.01, 132.78, 132.68, 132.63, 132.49, 127.93, 127.69, 127.45, 122.90, 122.78, 122.65, 110.20, 107.48, 42.41. ^{19}F NMR (375 MHz, C_6D_6): δ –159.01 (d, *J* = 22.2 Hz, 2*F*, fluorinated aryl C–*F*), –164.89 (t, *J* = 22.0 Hz, 2*F*, fluorinated aryl C–*F*), –171.13 (t, *J* = 22.0 Hz, 1*F*, fluorinated aryl C–*F*). HRMS (ESI$^+$) *m/z* Calc. 206.0442 $[C_{11}H_6F_5NO + H]^+$, Found 206.0445 $[M + H]^+$.

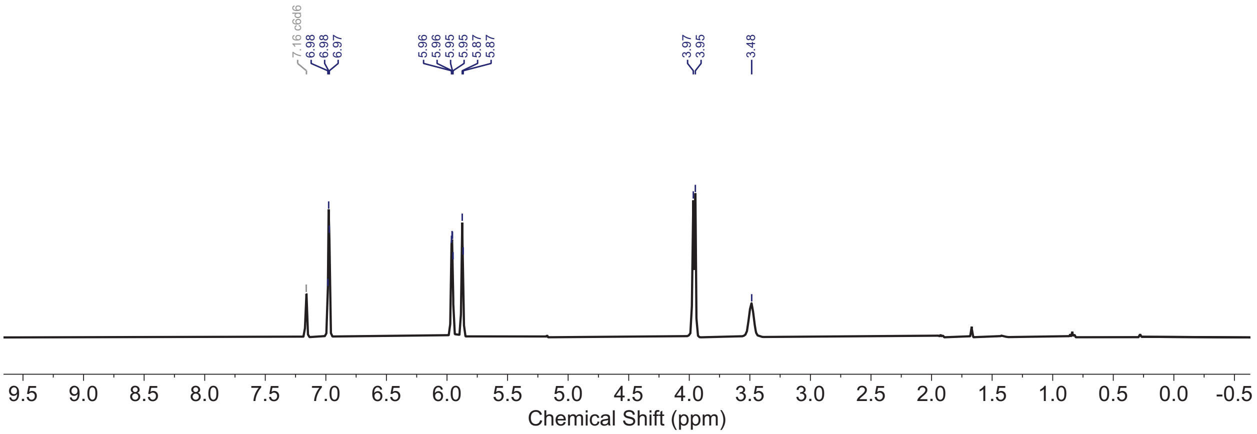

Figure S28. ^{1}H NMR spectrum (400 MHz, C_6D_6) of *N*-(perfluorophenyl)furan-2-amine.

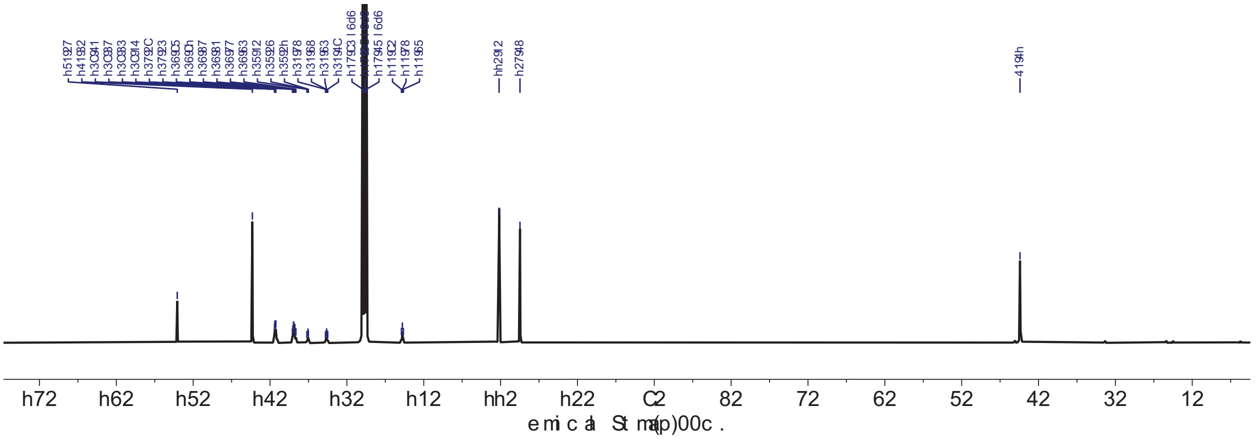

Figure S29. ^{13}C{^{1}H} NMR spectrum (100 MHz, C_6D_6) of *N*-(perfluorophenyl)furan-2-amine.

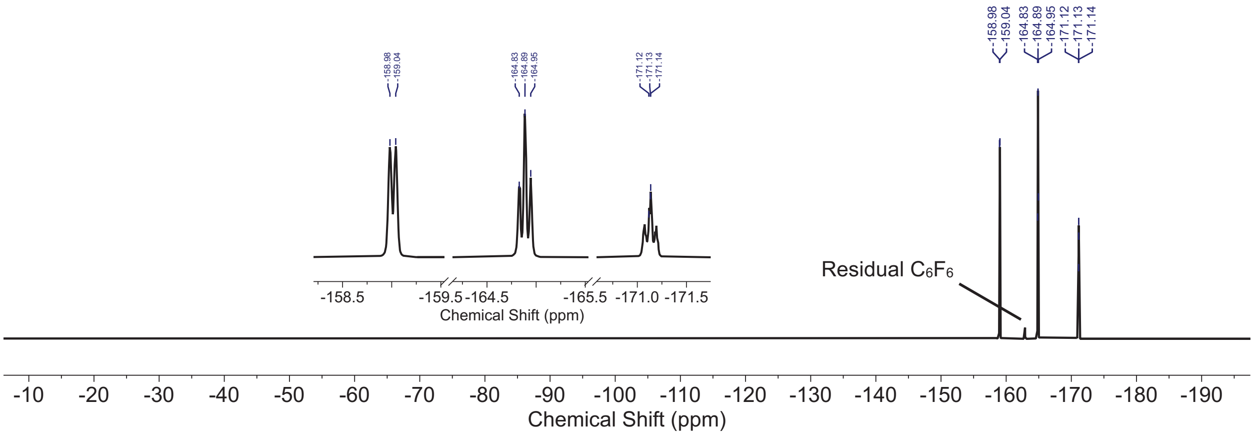

Figure S30. ^{19}F NMR spectrum (375 MHz, C_6D_6) of *N*-(perfluorophenyl)furan-2-amine.

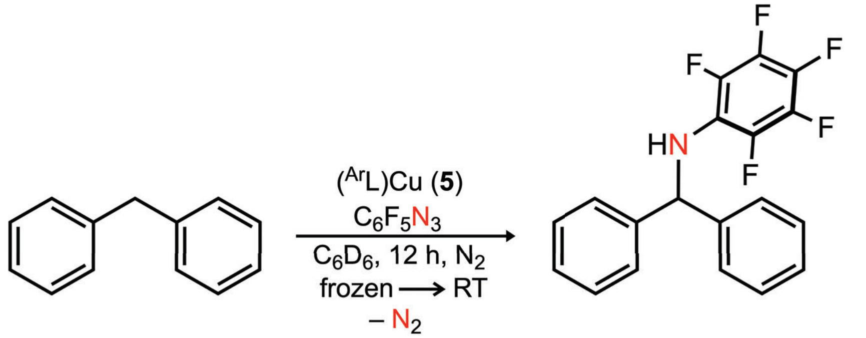

N-benzhydryl-2,3,4,5,6-pentafluoroaniline. Treatment of pentafluorophenyl azide (0.044 g, 0.212 mmol, 2.0 equiv.) with (^{Ar}L)Cu (**5**; 0.099 g, 0.106 mmol, 1.0 equiv.) in the presence of diphenylmethane (0.535 g, 3.184 mmol, 30 equiv.) in C_6D_6 (1 mL) over 16 h afforded the title complex in 41(2) % yield by ^{19}F NMR spectroscopy, which was purified by column chromatography (silica, hexanes/ethyl acetate following elution with neat hexanes to remove unreacted substrate) followed by acetonitrile trituration and filtration to yield the product as a dark oil. ^{1}H NMR (400 MHz, C_6D_6): 7.25 – 7.30 (m, 4*H*, *ortho* aryl C–*H*), 7.07 – 7.14 (m, 4*H*, *meta* aryl C–*H*), 7.00 – 7.06 (m, 2*H*, *para* aryl C–*H*), 5.46 (m, 1*H*, benzylic C–*H*), 3.96 (br, 1*H*, aniline N–*H*). ^{13}C{^{1}H} NMR (100 MHz, C_6D_6): 144.51, 128.27, 127.53, 126.69, 75.89 (unresolved C–*F*). ^{19}F NMR (375 MHz, C_6D_6): δ –157.58 (d, *J* = 22.5 Hz, 2*F*, fluorinated aryl C–*F*), –164.89 (td, *J* 23.4, 4.7, 2*F*, fluorinated aryl C–*F*), –171.13 (tt, *J* = 15.6 Hz, 4.2, 1*F*, fluorinated aryl C–*F*). HRMS (ESI$^+$): *m/z* Calc. 350.0963 [$C_{19}H_{12}F_5N$ + H]$^+$, Found 350.0957 [M + H]$^+$.

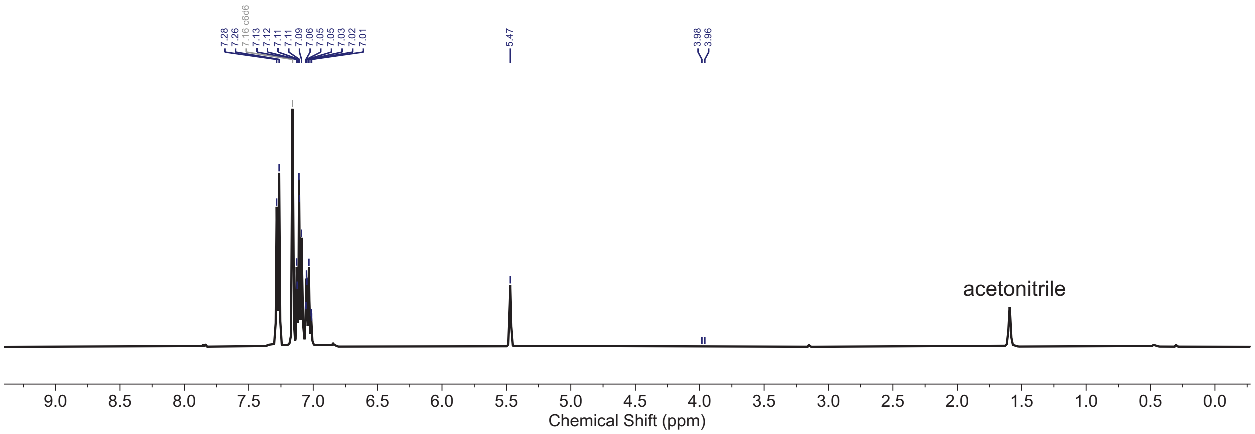

Figure S31. ^{1}H NMR spectrum (400 MHz, C_6D_6) of *N*-benzhydryl-2,3,4,5,6-pentafluoroaniline.

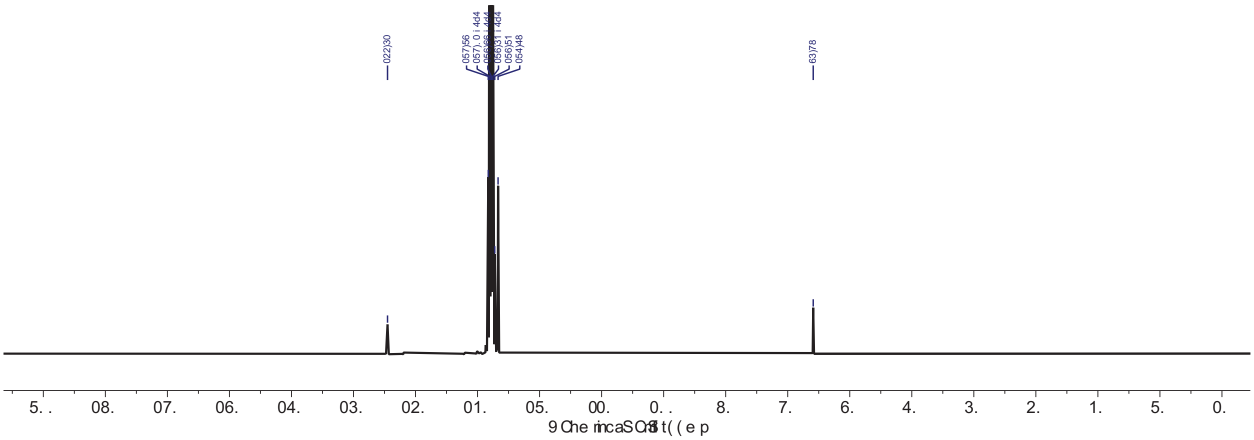

Figure S32. $^{13}C\{^{1}H\}$ NMR spectrum (100 MHz, C_6D_6) of *N*-benzhydryl-2,3,4,5,6-pentafluoroaniline.

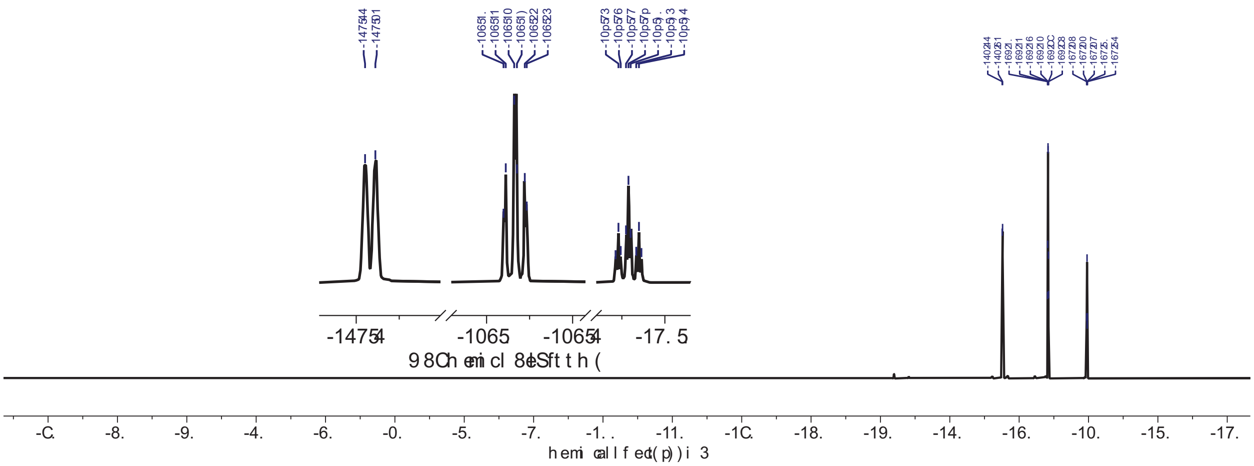

Figure S33. ^{19}F NMR spectrum (375 MHz, C_6D_6) of *N*-benzhydryl-2,3,4,5,6-pentafluoroaniline.

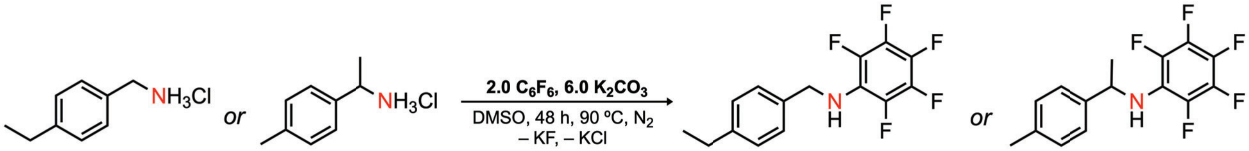

Adapting from a literature procedure,[5] either (4-ethylphenyl)methanamine•HCl or 1-(*p*-tolyl)ethan-1-amine•HCl (0.050 g, 0.291 mmol, 1.0 equiv.) was combined with hexafluorobenzene (0.110 g, 0.583 mmol, 2.0 equiv.) in a DMSO (4 mL) in a Schlenk tube. Following addition of K_2CO_3 (0.241 g, 1.746 mmol, 6.0 equiv.) under positive dinitrogen pressure, the mixture was heated at 90 ºC for five days with subsequent removal of solvent and extraction with hexanes to afford the corresponding aniline products. Repeated reactions revealed incomplete consumption of starting material with onset decomposition with prolonged stirring; the NMR shifts of the product are reported below.

***N*-(4-ethylbenzyl)-2,3,4,5,6-pentafluoroaniline.** ^{1}H NMR (500 MHz, C_6D_6): δ 7.13 (d, *J* = 7.8 Hz, 1*H*, aryl C–*H*), 7.05 (d, *J* = 7.7 Hz, 1*H*, aryl C–*H*), 3.58 (s, 2*H*, substituted benzylic C–H), 3.52 (br. s, 1*H*, aniline N–*H*), 2.48 (q, *J* = 7.6 Hz, 2*H*, benzylic C–*H*), 1.11 (t, *J* = 7.5 Hz, 2*H*, terminal methyl C–*H*). ^{13}C{^{1}H} (125 MHz, C_6D_6): δ 143.52, 136.14, 128.43, 128.08, 127.36, 127.11. ^{19}F NMR (470 MHz, C_6D_6): δ –159.43 (m, 2*F*, *ortho* aryl C–*F*), –164.92 (m, 2*F*, *para* aryl C–*F*), –172.08 (m, 2*F*, *meta* aryl C–*F*). HRMS (ESI$^+$) *m/z* Calc. 302.0963 [$C_{15}H_{12}F_5N$ + H]$^+$, Found 302.0973 [M + H]$^+$.

2,3,4,5,6-pentafluoro-*N*-(1-(*p*-tolyl)ethyl)aniline. ^{1}H NMR (500 MHz, C_6D_6): δ 7.19 (d, *J* = 7.8 Hz, 2*H,* aryl C–*H*), 7.02 (d, *J* = 7.8 Hz, 2*H,* aryl C–*H*), 3.84 (q, *J* = 6.6 Hz, 1*H*, substituted benzylic C–*H*), 3.64 (br. s, 1*H*, aniline N–*H*), 2.15 (s, 3*H*, benzylic C–*H*), 1.20 (d, *J* = 6.6 Hz, 2*H*, methyl C–*H*). ^{13}C{^{1}H} (125 MHz, C_6D_6): δ 145.27, 135.69, 129.25, 128.87, 125.73, 125.41, 50.95, 25.85, 20.66 (unresolved C–*F*). ^{19}F NMR (470 MHz, C_6D_6): δ –158.23 (m, 2*F*, *ortho* aryl C–*F*), –164.66 (m, 2*F*, *para* aryl C–*F*), –170.74 (m, 2*F*, *meta* aryl C–*F*). HRMS (ESI$^+$) *m/z* Calc. 340.0521 [$C_{15}H_{12}F_5N$ + K]$^+$, Found 340.0532 [M + K]$^+$.

Note: The title complexes could be access as an inseparable mixture following addition of pentafluorophenyl azide to a solution of (^{ArF}L)Cu (**4**) or (^{ArF}L)Cu (**5**) containing 4-ethyltoluene.

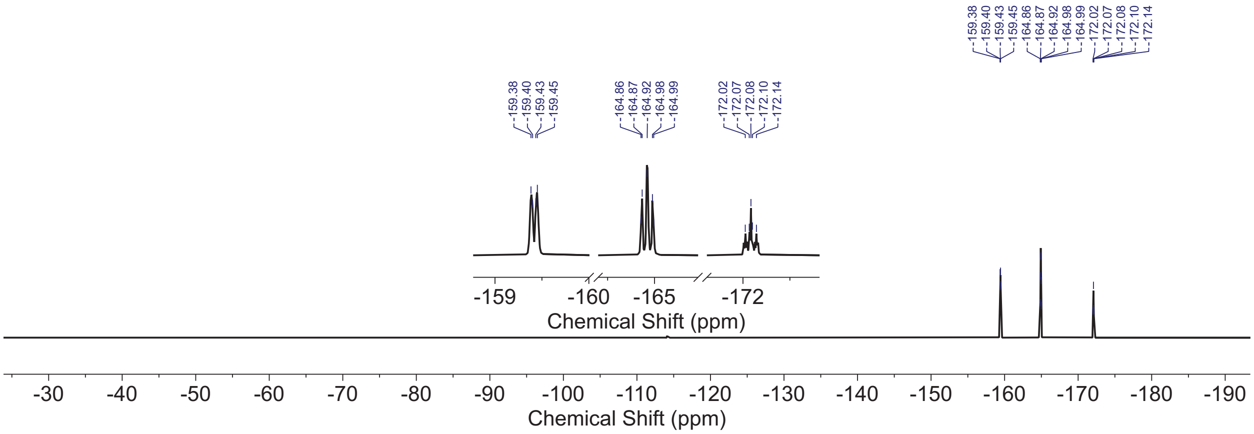

Figure S34. ^{19}F NMR spectrum (375 MHz, C_6D_6) of *N*-(4-ethylbenzyl)-2,3,4,5,6-pentafluoroaniline. Inset highlights aryl C–*F* resonances.

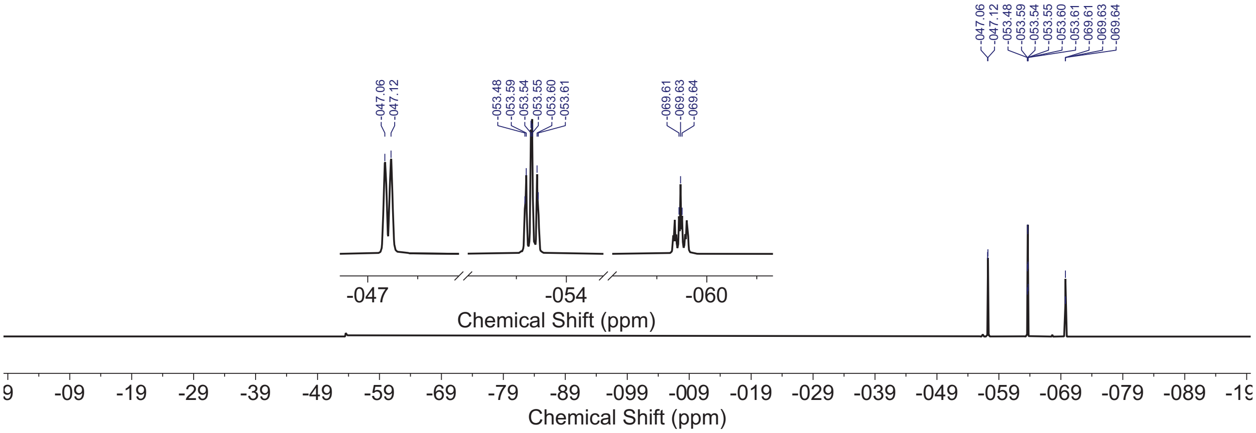

Figure S35. ^{19}F NMR spectrum (375 MHz, C_6D_6) of 2,3,4,5,6-pentafluoro-*N*-(1-(*p*-tolyl)ethyl)aniline. Inset highlights aryl C–*F* resonances.

Stoichiometric Mechanistic Analysis.

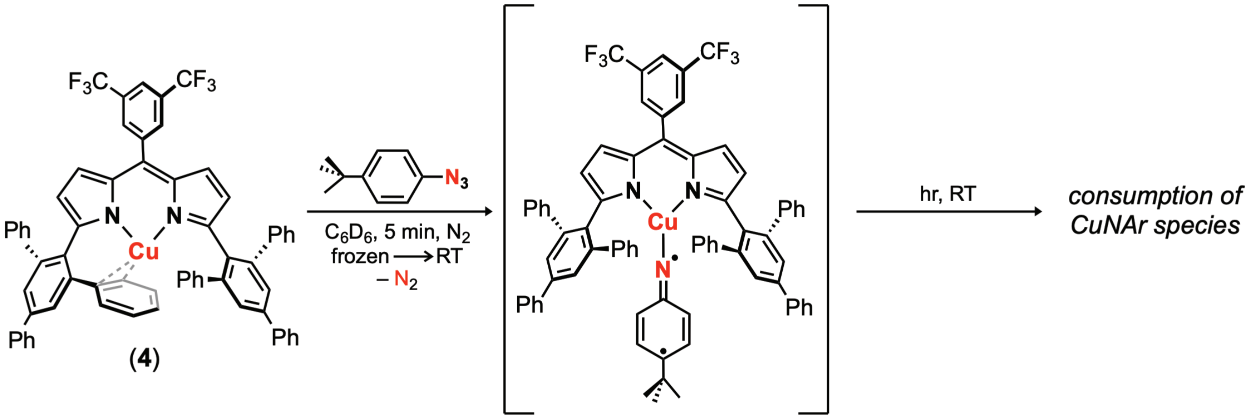

Detection of Copper Nitrene Species by ^{1}H NMR Spectroscopy. *All manipulations were conducted using silanized glassware.* In a dinitrogen-filled drybox, to a thawing solution of (^{ArF}L)Cu (**4**) in C_6D_6 in a J. Young NMR tube was added 4-(tert-butyl)phenyl azide.[15] Within 5 minutes, the mixture was analyzed by multinuclear NMR spectroscopy, revealing diagnostic ^{1}H resonances[3] attributed to (^{ArF}L)Cu(N($C_6H_4{}^t$Bu)), with the majority species in solution as unreacted **4**. Re-analysis of the reaction mixture after 4 h revealed consumption of (^{ArF}L)Cu(N($C_6H_4{}^t$Bu)), attributed to the poor thermal stability.

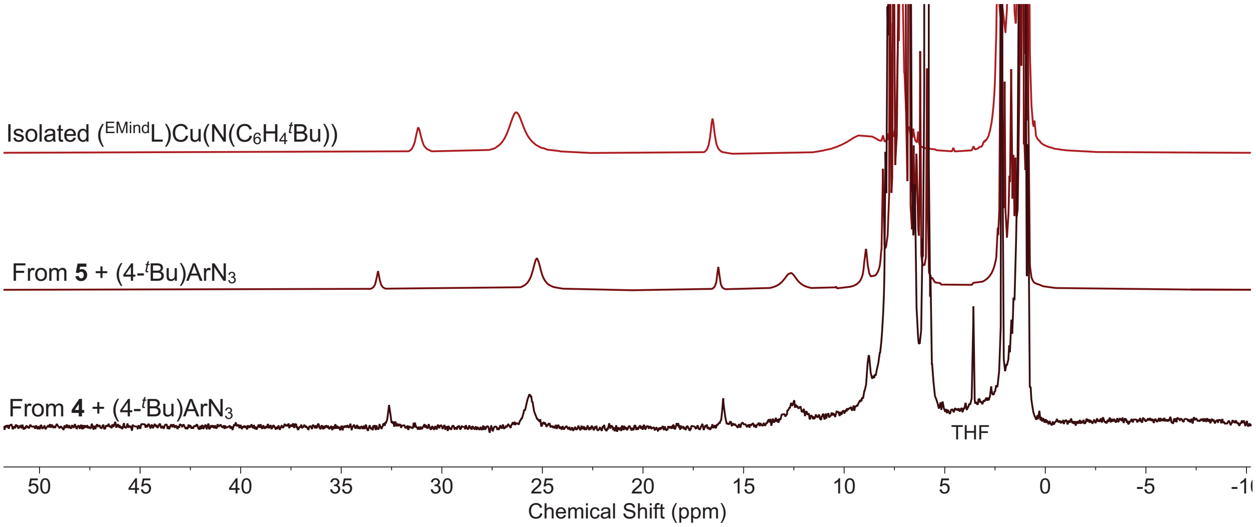

Figure S36. Juxtaposed ^{1}H NMR spectra (500 MHz, C_6D_6) of copper nitrene species with distinct ligand platforms. Preparations from addition of 4-(tert-butyl)phenyl azide from (^{ArF}L)Cu (**4**) (*bottom*) and (^{ArF}L)Cu (**5**) reveal analogous ^{1}H resonances to authentic (^{EMind}L)Cu(N($C_6H_4{}^t$Bu)).

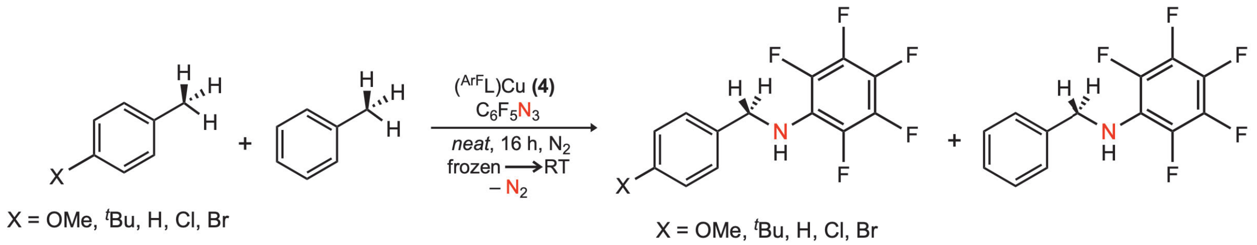

Competition Toluene Amination Experiments. *All manipulations were conducted using silanized glassware.* In a dinitrogen-filled drybox, an equimolar solution of toluene (0.400 g, 4.341 mmol) and the *para*-substitued toluene (4.341 mmol) was prepared and homogenized. This mixture was added to precise quantity of pentafluorophenyl azide[16] ($C_6F_5N_3$; 0.002 g, 0.009 mmol, 1.0 equiv.) and frozen in a liquid-nitrogen chilled cold well. Upon thawing, the mixture was added to solid (^{ArF}L)Cu (**4**) (0.009 g, 0.009 mmol, 1.0 equiv.) and subsequently transferred to a J. Young NMR tube. After *ca*. 16 h, the mixture was checked by ^{19}F NMR spectroscopy to ensure complete consumption of $C_6F_5N_3$ and to quantify the relative ratios of aminated toluene products, which were prepared independently as authentic ^{19}F NMR spectroscopy references.[5] Average values are reported with error bars denoting the first standard deviation.

For data processing, the integration values of isostructural C–*F* were compared and plotted as a logarithmic ratio. Analysis of ligand resonances from (^{ArF}L)Cu support near near-identical yields between toluene competition experiments.

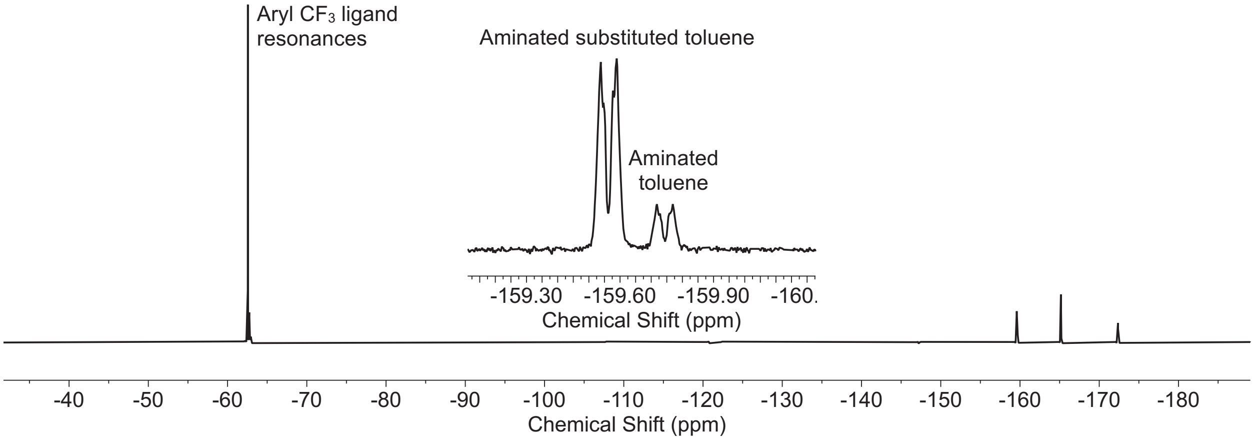

Figure S37. Representative competition Hammett ^{19}F NMR spectrum (470 MHz, C_6D_6) of a mixture of toluene and (4–^{t}Bu)toluene, revealing full consumption of $C_6F_5N_3$. Inspect depicts truncated ^{19}F NMR spectrum (470 MHz, C_6D_6) focusing on two sets of ^{19}F resonances attributed to similar species.

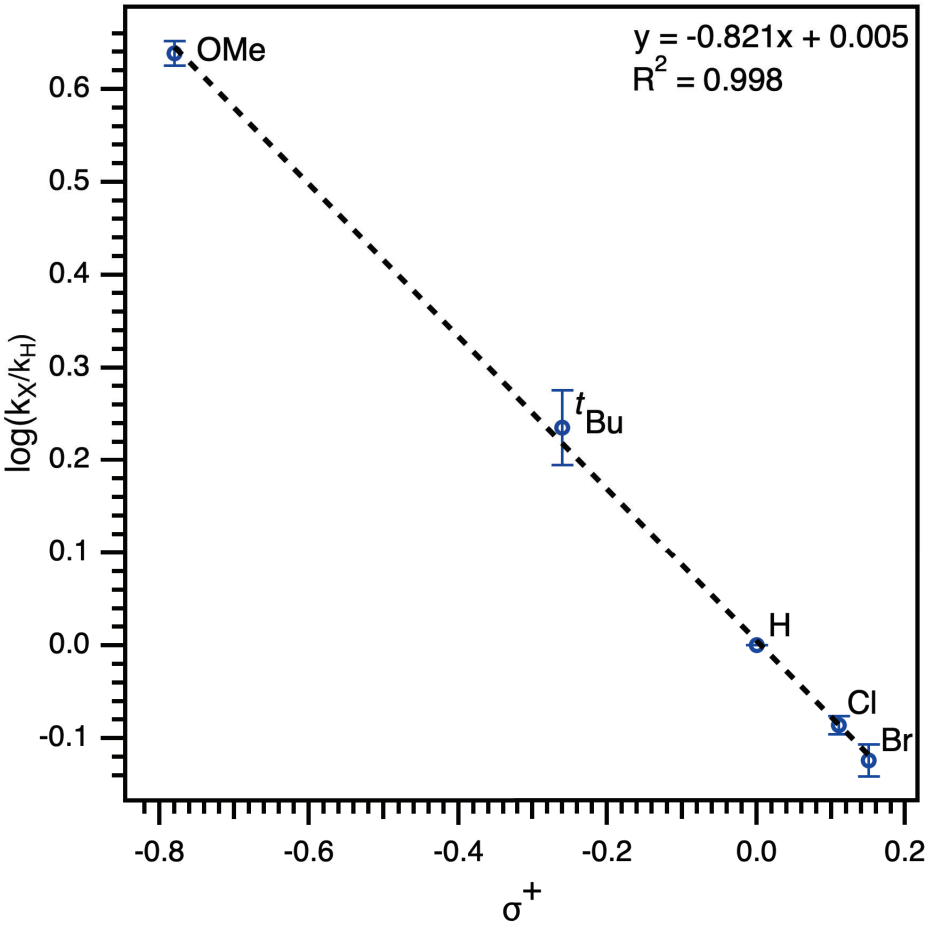

Figure S38. Competition Hammett analysis for toluene amination, plotted as a function of σ^+. Because substrate activation and functionalization are irreversible, $\log(k_X/k_H)$ is equal to the logarithm of the ratio of the aminated product yields, determined by relative ^{19}F NMR integration.

Table S1. Competition Hammett Experiment for Toluene Amination.

***Para* Substitution**	**σ^+ Value[17]**	**$\log(k_X/k_H)$**	**Standard Deviation**
H	0.00 (by definition)	0 (by definition)	0 (by definition)
Cl	0.11	–0.087	0.011
Br	0.15	–0.124	0.017
^{t}Bu	–0.26	0.236	0.044
4OMe	–0.78	0.638	0.013

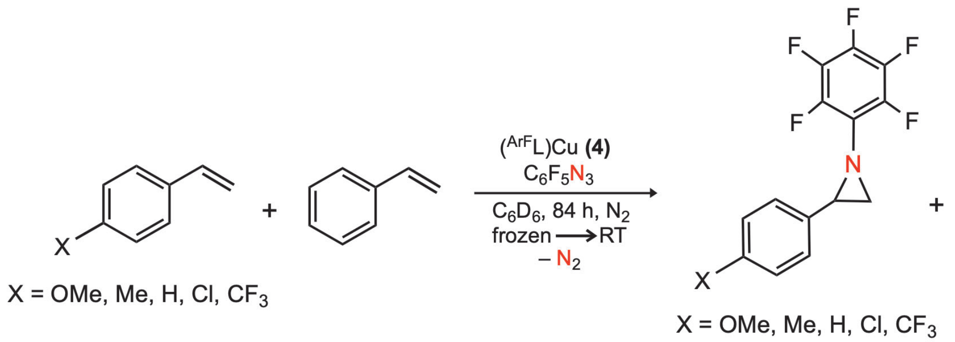

Competition Styrene Aziridination Experiments. *All manipulations were conducted using silanized glassware.* In a dinitrogen-filled drybox, an equimolar solution of freshly purified styrene (0.045 mmol, 10.0 equiv.) and and *para*-substituted styrene (0.045 mmol, 10.0 equiv.) was prepared and dilute with C_6D_6 (1.0 mL). This mixture was added to precise quantity of pentafluorophenyl azide ($C_6F_5N_3$; 0.002 g, 0.009 mmol, 1.0 equiv.) and frozen in a liquid-nitrogen chilled cold well. Upon thawing, the mixture was added to solid (^{ArF}L)Cu (**4**) (0.009 g, 0.009 mmol, 1.0 equiv.) and subsequently transferred to a J. Young NMR tube. After *ca*. 84 h, the mixture was checked by ^{19}F NMR spectroscopy to ensure complete consumption of $C_6F_5N_3$ and to quantify the relative ratios of aziridinated styrene products, which were prepared independently as authentic ^{19}F NMR spectroscopy references.[18-19] Average values are reported with error bars denoting the first standard deviation.

For data processing, the integration values of isostructural C–*F* were compared and plotted as a logarithmic ratio. Analysis of ligand resonances from (^{ArF}L)Cu (**4**) support near near-identical yields between toluene competition experiments.

Note: although sluggish kinetics are exhibited due to competitive coordination of pentafluorophenyl azide versus styrene, the relative ratio of the aziridinated products was constant throughout the reaction time course prior to complete consumption of aryl azide. In addition, decreasing the number of equivalents of styrene resulted in identical product distributions with the reaction complete in a shorter time frame (e.g., 40 h for full consumption of aryl azide in the presence of 5.0 equiv. of styrene and 5.0 equiv. of *para*-substituted styrene).

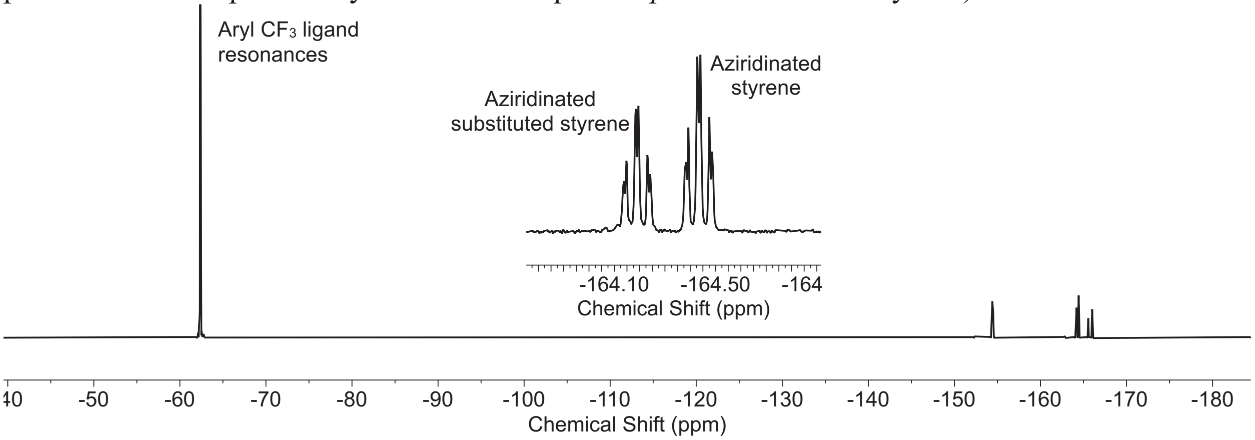

Figure S39. Representative competition Hammett ^{19}F NMR spectrum (470 MHz, C_6D_6) of a mixture of styrene and (4–Cl)styrene, revealing full consumption of $C_6F_5N_3$. Inspect depicts truncated ^{19}F NMR spectrum (470 MHz, C_6D_6) focusing on two sets of ^{19}F resonances attributed to competition aziridination.

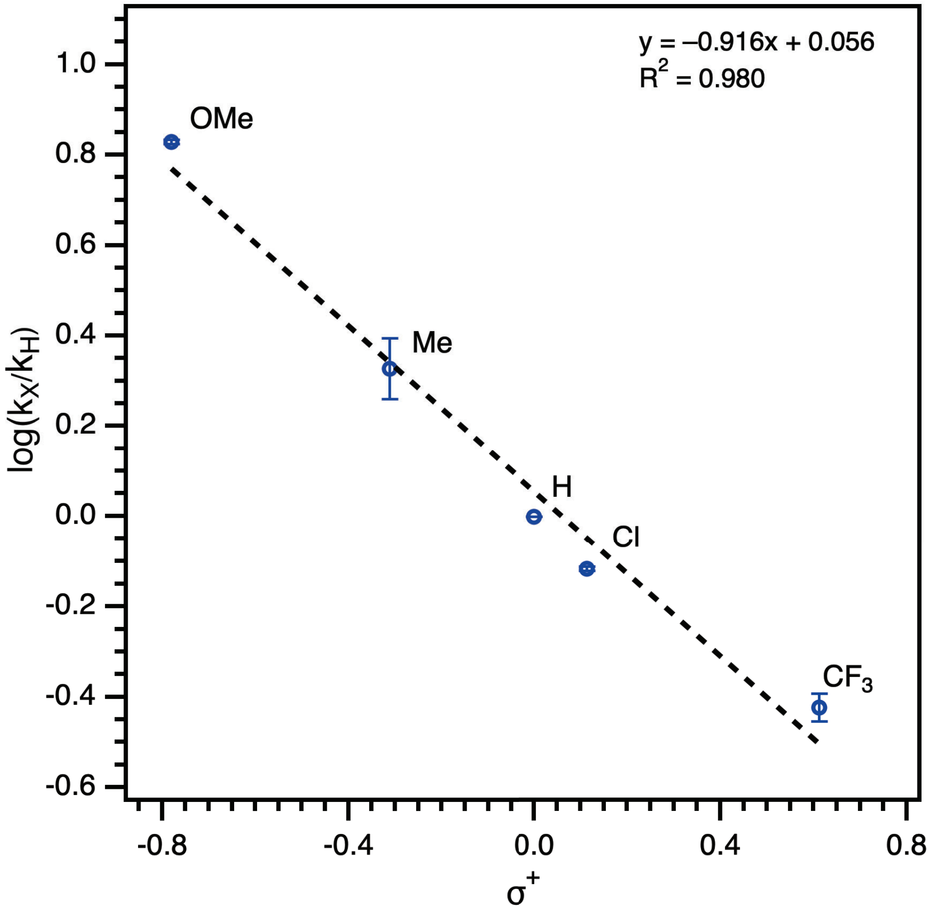

Figure S40. Competition Hammett analysis for styrene aziridination, plotted as a function of σ^+. Because substrate activation and functionalization are irreversible, $\log(k_X/k_H)$ is equal to the logarithm of the ratio of the aziridinated styrene products, determined by relative ^{19}F NMR integration.

Table S2. Competition Hammett Experiment for Styrene Aziridination.

Para Substitution	σ^+ Value[17]	$\log(k_X/k_H)$	Standard Deviation
H	0.00 (by definition)	0 (by definition)	0 (by definition)
Cl	0.11	–0.117	0.010
CF_3	0.61	–0.424	0.031
Me	–0.31	0.326	0.067
OMe	–0.78	0.829	0.011

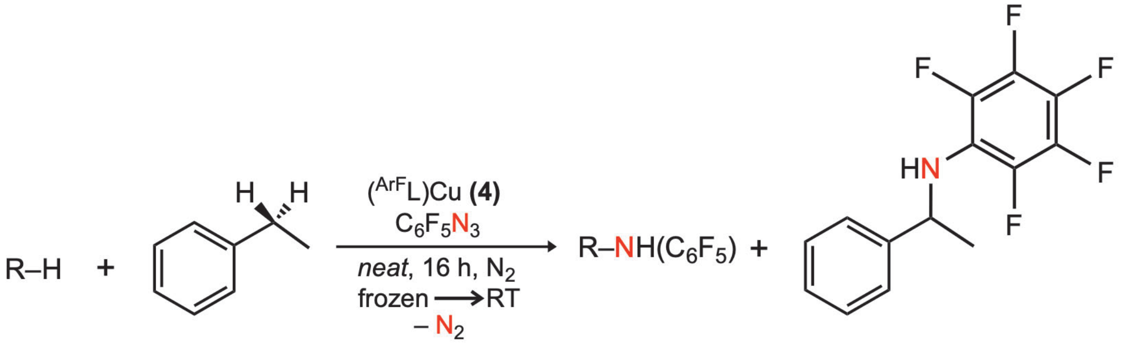

Competition Substrate Amination Experiments. *All manipulations were conducted using silanized glassware*. In a dinitrogen-filled drybox, an equimolar solution of ethylbenzene (0.461 g, 4.341 mmol) and competing amination substrate (4.341 mmol) was prepared and homogenized. This mixture was added to precise quantity of pentafluorophenyl azide ($C_6F_5N_3$; 0.002 g, 0.009 mmol, 1.0 equiv.) and frozen in a liquid-nitrogen chilled cold well. Upon thawing, the mixture was added to solid (^{ArF}L)Cu (**4**) (0.009 g, 0.009 mmol, 1.0 equiv.) and subsequently transferred to a J. Young NMR tube. After *ca*. 16 h, the mixture was checked by ^{19}F NMR spectroscopy to ensure complete consumption of $C_6F_5N_3$ and to quantify the relative ratios of aminated products, which were prepared independently as authentic ^{19}F NMR spectroscopy references.[18, 20] Average values are reported with error bars denoting the first standard deviation.

Note: although the data exhibits an apparent linear trend between C–H bond strength and relative rate of substrate consumption, we note that the *absolute* yield of functionalized substrate varies between specific amination trials. In particular, amination competition experiments with stronger C–H bonds present results in a decreased *absolute* yield of both products, attributed to competitive formation of the corresponding cupric anilide species as gauged from aryl CF_3 ligand resonances. Consequently, we strictly present this data to demonstrate the relative trend of amination rate against C–H bond strength, noting aminated products associated with weaker C–H bonds are observed in higher yields compared to aminated products with stronger C–H bonds.

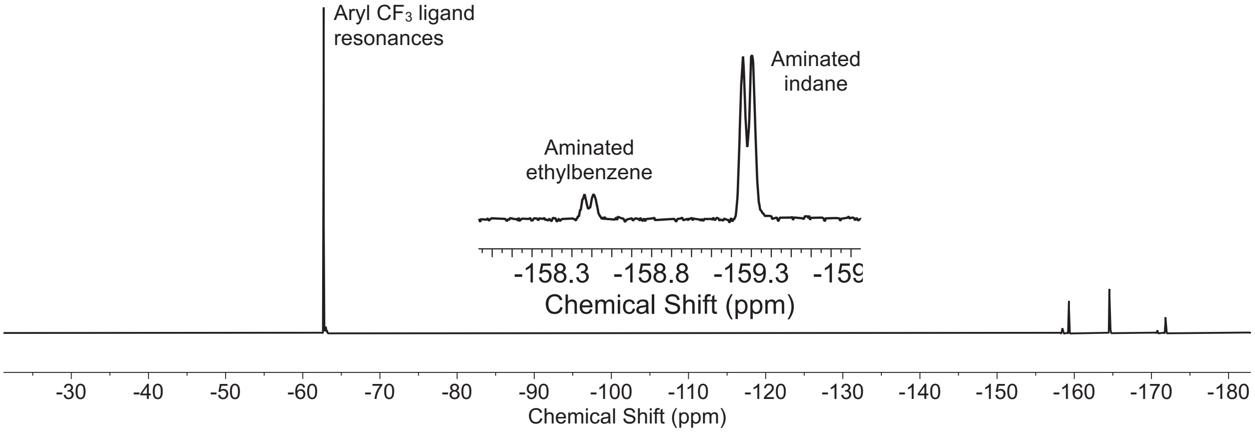

Figure S41. Representative competition Hammett ^{19}F NMR spectrum (470 MHz, C_6D_6) of a mixture of ethylbenzene and indane, revealing full consumption of $C_6F_5N_3$. Inspect depicts truncated ^{19}F NMR spectrum (470 MHz, C_6D_6) focusing on two sets of ^{19}F resonances attributed to competition amination.

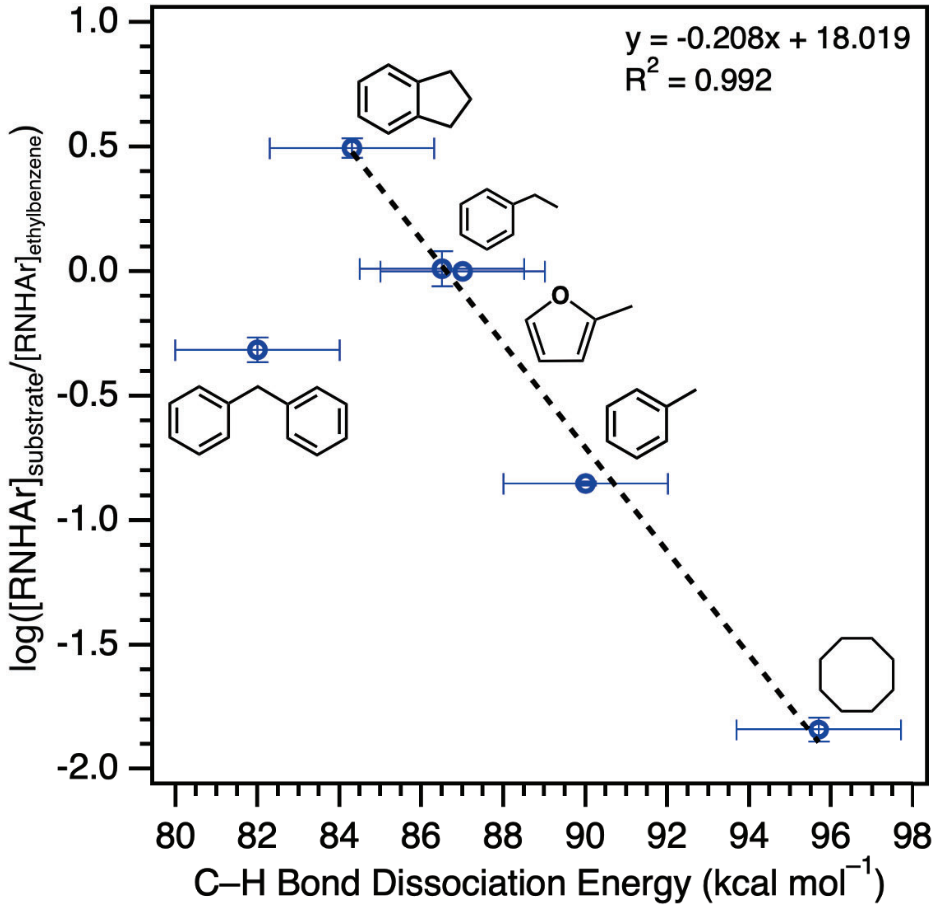

Figure S42. Competition Hammett analysis as a function of C–H bond dissociation energy, displaying increased yield of aminated product with decreasing C–H bond strength. The linear regression is conducted on all data points, excluding diphenylmethane with deviation attributed to the steric profile of the substrate. Ethylbenzene is set as the reference point.

Table S3. Competition Hammett Experiment for Competition Amination.[a]

Substrate	C–H BDE (kcal mol^{-1})[21]	$\log_{10}(pdt_2/pdt_1)$	Standard Deviation
Indane	84.3	0.494	0.040
Ethylbenzene	87.0	0 (by definition)	0 (by definition)
2-methylfuran	86.5	0.010	0.070
Toluene	90.0	–0.852	0.009
Cyclooctane	95.7	–1.841	0.050
Diphenylmethane	82.0	–0.316	0.053

[a]Each C–H bond strength is bracketed with a 2 kcal mol^{-1} error bar.[22-23]

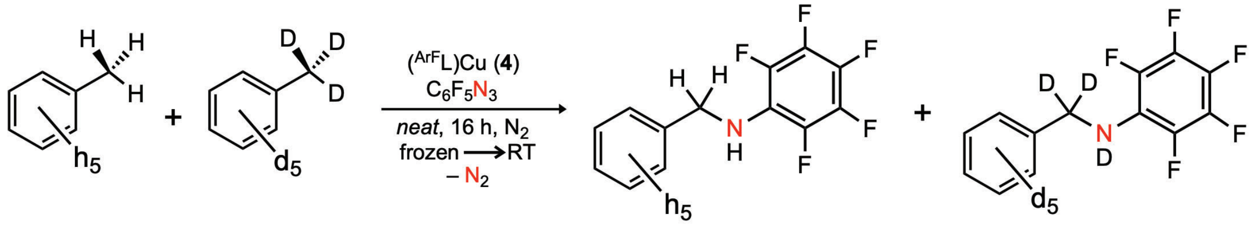

Intermolecular h_8–toluene/d_8–toluene Competition Kinetic Isotope Effect (KIE) Measurement. *All manipulations were conducted using silanized glassware*. In a dinitrogen-filled drybox, a precise quantity of pentafluorophenyl azide ($C_6F_5N_3$; 0.002 g, 0.010 mmol, 1.0 equiv.) was combined with an equimolar mixture of h_8–toluene (0.400 g, 4.34 mmol) and d_8–toluene (0.435 g, 4.34 mmol). The solution was frozen in a liquid-nitrogen chilled cold well. Upon thawing, the mixture was added to solid (^{ArF}L)Cu (**4**) (0.010 g, 0.010 mmol, 1.0 equiv.) and transferred to a scintillation vial to stir for *ca*. 16 h. Following lyophilization and prolonged vacuum exposure to ensure removal of unreacted solvent, the residual solid was dissolved in C_6D_6 containing an internal standard of 4–fluoroanisole (10 μL) and checked by multinuclear NMR spectroscopy to determine the kinetic isotope effect (see derivation below). The measured KIE was 9.0(7) following triplicate measurements.

Derivative of KIE Formula: As the ^{19}F NMR resonances are equivalent between C–H activated and C–D activated products, we note that the ^{19}F NMR resonances integrate to the combined summed quantity of C–H activated and C–D activated products per resonance. We define α as the ratio of the summed functionalized product value to the internal standard (IS) concentration of 4-fluoroanisole (displaying a single fluorine atoms), which is evaluated by ^{19}F NMR spectroscopy and normalized to the number of fluorine atoms per resonance (eq. 1).

$$\alpha = \frac{\Sigma(pdt\ ^{19}F)}{[\Sigma(IS\ ^{19}F)} = \frac{([\mathrm{C}{-}H]\ activated\ ^{19}F + [\mathrm{C}{-}D]\ activated\ ^{19}F)/(\#\ of\ equivalent\ ^{19}F\ atoms)}{(internal\ standard\ ^{19}F)/1} \quad (1)$$

The number of equivalent ^{19}F atoms is dependent on which fluorine resonances are selected for integration: *ortho* and *meta* resonances display two chemically equivalent ^{19}F atoms each, and the *para* resonance is due to a unique ^{19}F atom.

We define β as the ratio of the normalized C–*H* resonance of the benzylic C–*H* resonance to the normalized C–*H* resonance of methoxy motif of the internal standard 4–fluoroanisole (exhibiting three equivalent protons), which is evaluated by ^{1}H NMR spectroscopy (eq. 2).

$$\beta = \frac{([\mathrm{C}{-}H]\ activated\ ^{1}H)/(\#\ of\ equivalent\ \ ^{1}H\ atoms)}{(IS\ ^{1}H)/3} \quad (2)$$

We find following, nothing the activatable substrate remains in vast excess over the entire reaction such that the KIE can be approximated as a relative concentration (eq. 3):

$$KIE = \frac{k_H}{k_D} \approx \frac{\left[[\mathrm{C}{-}H]\ activated\ pdt\right]}{\left[[\mathrm{C}{-}D]\ activated\ pdt\right]} = \frac{[C{-}H]\ activated\ pdt}{[C{-}D]\ activated\ pdt} = \frac{[\mathrm{C}{-}H]\ activated\ pdt}{\Sigma\left(pdt\ ^{19}F\right) - \ [\mathrm{C}{-}H]\ activated\ pdt}$$
$$= \frac{\beta}{\alpha - \beta} \Rightarrow \mathbf{KIE} = \frac{\boldsymbol{\beta}}{\boldsymbol{\alpha} - \boldsymbol{\beta}} \quad (3)$$

Worth noting and emphasizing is that the computed KIE is independent of (i) product yield, (ii) concentration of reagents in solution, and (iii) internal standard quantity. Nonetheless, a non-zero quantity of internal standard is necessary for quantification.

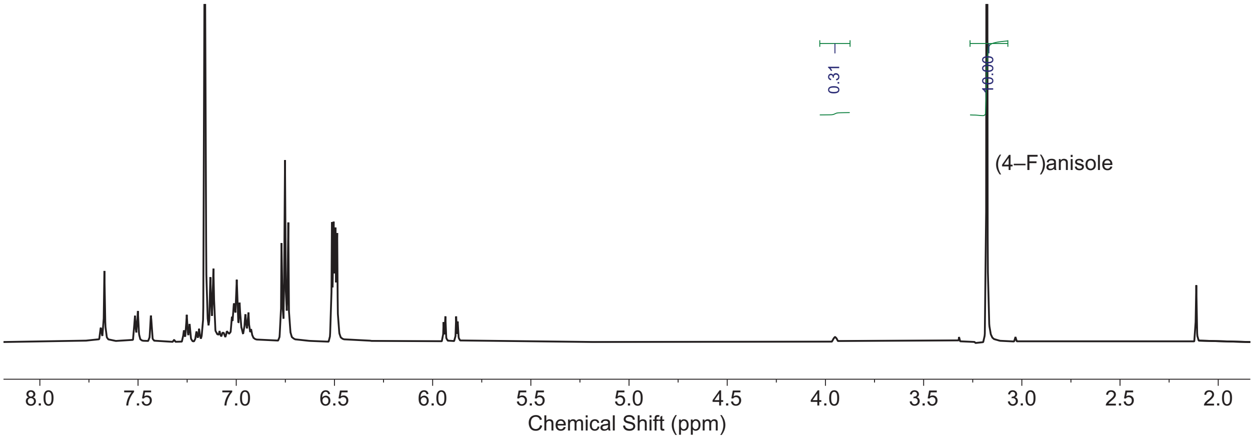

Figure S43. Representative ^{1}H NMR spectrum (500 MHz, C_6D_6) of crude intermolecular h_8–toluene/d_8–toluene competition KIE measurement with $C_6F_5N_3$.

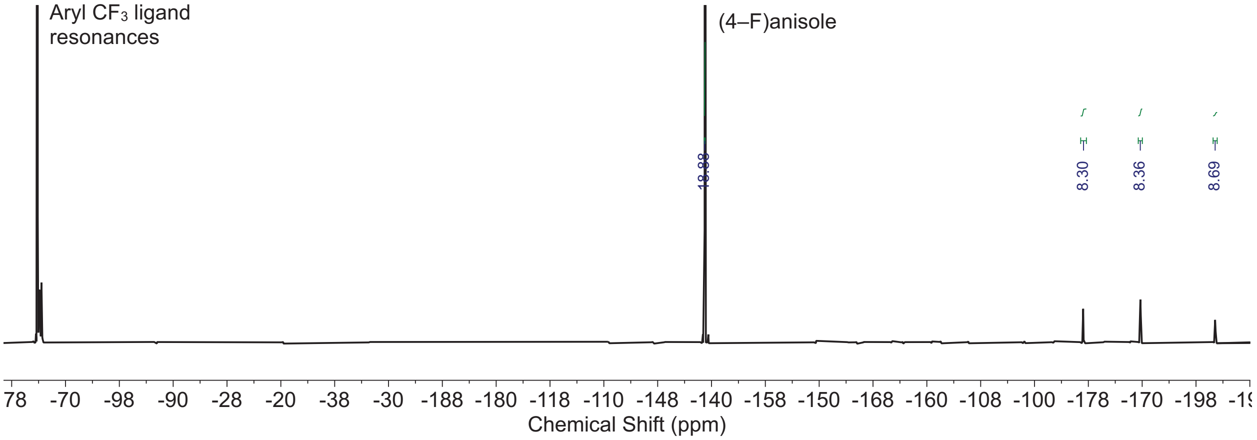

Figure S44. Representative ^{19}F NMR spectrum (470 MHz, C_6D_6) of crude intermolecular h_8–toluene/d_8–toluene competition KIE measurement with $C_6F_5N_3$.

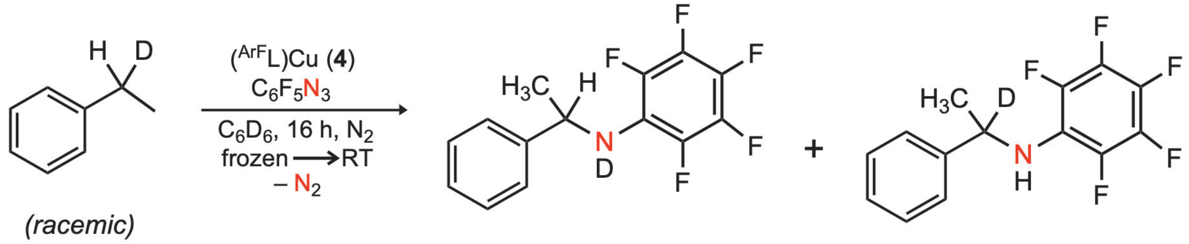

Intramolecular d_1-ethylbenene Competition Kinetic Isotope Effect (KIE) Measurement. *All manipulations were conducted using silanized glassware*. In a dinitrogen-filled drybox, a precise quantity of pentafluorophenyl azide ($C_6F_5N_3$; 0.002 g, 0.010 mmol, 1.0 equiv.) was combined with d_1-ethylbenzene (0.050 g, 0.467 mmol, 48.0 equiv.) in C_6D_6 (0.7 mL) and frozen in a liquid-nitrogen chilled cold well. Upon thawing, the mixture was added to solid (^{ArF}L)Cu (**4**) (0.010 g, 0.010 mmol, 1.0 equiv.) and transferred to a scintillation vial to stir for *ca*. 16 h. Following lyophilization and prolonged vacuum exposure to ensure removal of unreacted d_1–ethylbenzene, the residual solid was dissolved in C_6D_6 and checked by ^{1}H NMR spectroscopy to quantify the relative benzylic C–*H* resonance against the terminal methyl C–*H* resonance to determine the kinetic isotope effect. The measured KIE was 4.4(2).

Derivative of KIE Formula for d_1–ethylbenzene amination: Assigning x as the relative concentration of [C–*H*] activated to [C–*D*] activated d_1-ethylbenzene and α as the ratio of summed benzylic proton resonances to methyl proton resonances (which is evaluated by ^{1}H NMR spectroscopy), and we arrive at the following two equations (eq. 4, eq. 5).

$$KIE = \frac{k_H}{k_D} = \frac{[[\text{C}\!-H]\ activated\ pdt]}{[[\text{C}\!-D]\ activated\ pdt]} = \frac{[C\!-H]\ activated\ pdt}{[\text{C}\!-D]\ activated\ pdt} \equiv x \qquad (4)$$

$$\alpha = \frac{\Sigma(benzylic\ ^1H)}{\Sigma(methyl\ ^1H)} = \frac{x*1+1*0}{x*3+1*3} = \frac{x}{3x+3} \Rightarrow \mathbf{KIE} = \frac{\mathbf{3}\boldsymbol{a}}{\mathbf{1}-\mathbf{3}\boldsymbol{a}} \qquad (5)$$

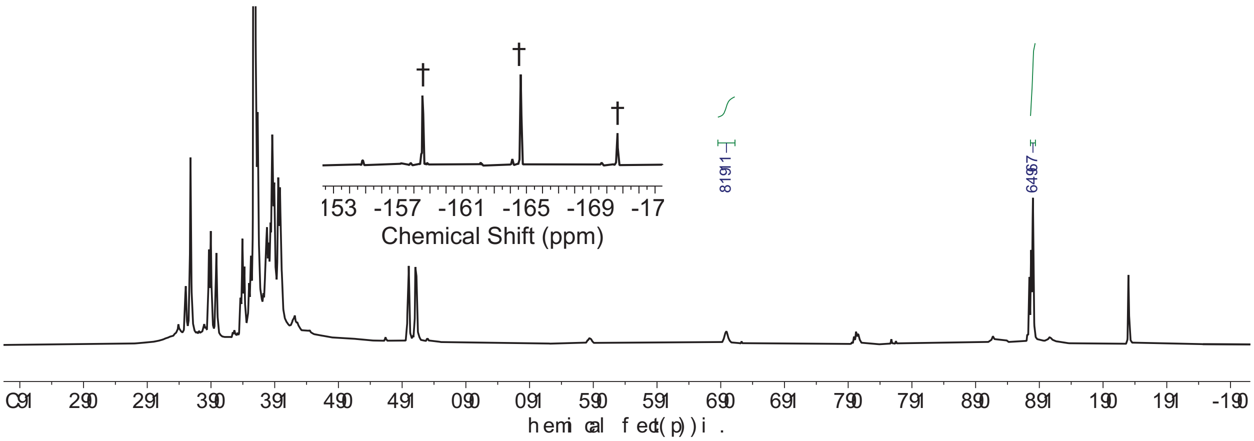

Figure S45. Representative ^{1}H NMR spectrum (500 MHz, C_6D_6) of crude intramolecular d_1-ethylbenene competition KIE measurement with aryl azide $C_6F_5N_3$. Inspect depicts truncated ^{19}F NMR spectrum (470 MHz, C_6D_6) of aryl C–F bond resonances of aminated ethylbenzene (†).

Competition Substrate Experiments.

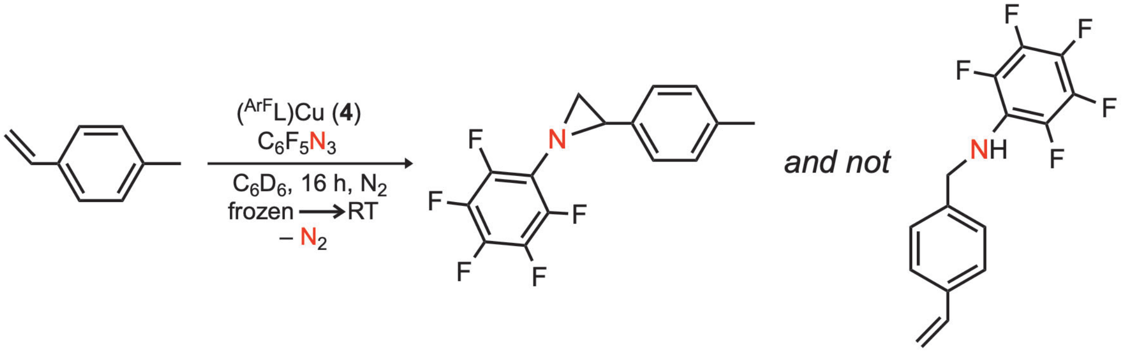

Competition: Styrene Aziridination *vs* Benzylic Amination. *All manipulations were conducted using silanized glassware*. In a dinitrogen-filled drybox, pentafluorophenyl azide ($C_6F_5N_3$; 0.002 g, 0.010 mmol, 1.0 equiv.) was combined with 4–methylstyrene (0.005 g, 0.045 mmol, 5.0 equiv.) with internal standard fluorobenzene in C_6D_6 (0.7 mL) and frozen in a liquid-nitrogen chilled cold well. Upon thawing, the mixture was added to solid (^{ArF}L)Cu (**4**) (0.010 g, 0.010 mmol, 1.0 equiv.) and transferred to a J. Young NMR tube for *ca*. 16 h. The reaction was subsequently checked by multinuclear $^{1}H/^{19}F$ NMR spectroscopy, revealing formation of the corresponding known aziridine[18] with no evidence of the corresponding benzylic aminated species. The formation of product was observed in 85(2) % yield over multiple trials with the mass balance attributed to formation of the cupric anilide species.

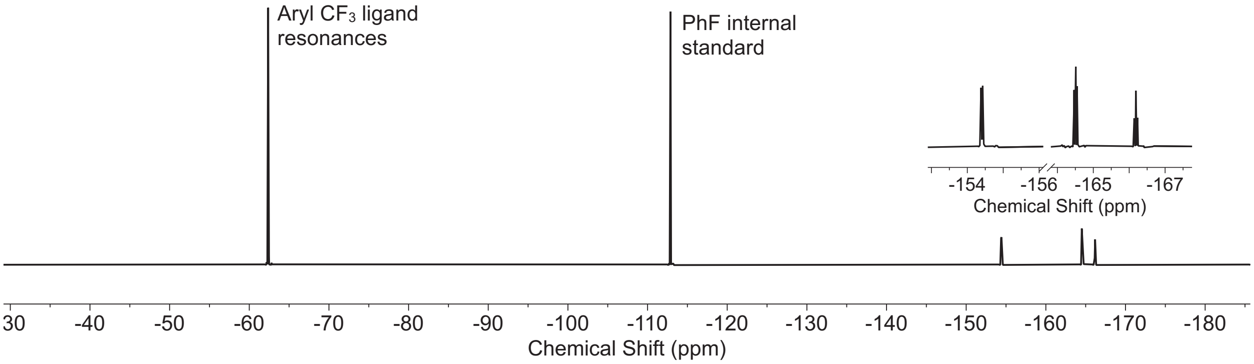

Figure S46. Representative ^{19}F NMR spectrum (470 MHz, C_6D_6) of crude 4–methylstyrene aziridination reaction, revealing exclusive formation of aziridine product as the organic aminated species. Inspect depicts truncated ^{19}F NMR spectrum (470 MHz, C_6D_6) of aryl C–F bond resonances of aziridine.

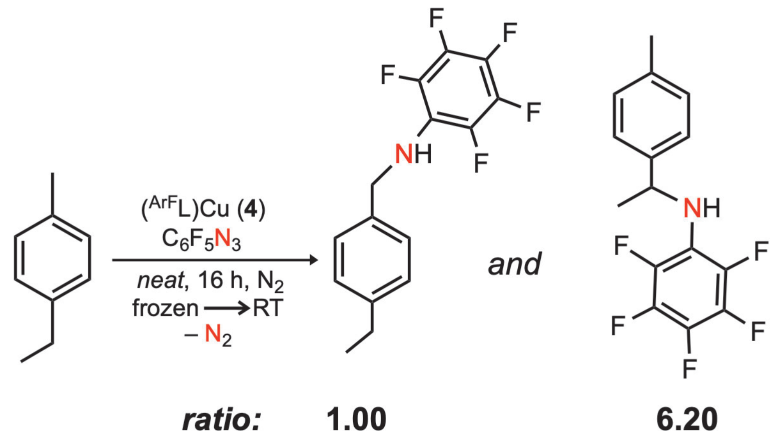

Competition: Primary Benzylic Amination *vs* Secondary Benzylic Amination. *All manipulations were conducted using silanized glassware*. In a dinitrogen-filled drybox, pentafluorophenyl azide ($C_6F_5N_3$; 0.002 g, 0.010 mmol, 1.0 equiv.) was dissolved in neat 4–ethyltoluene (0.7 mL) with internal standard fluorobenzene and frozen in a liquid-nitrogen chilled cold well. Upon thawing, the mixture was added to solid (^{ArF}L)Cu (**4**) (0.010 g, 0.010 mmol, 1.0 equiv.) and transferred to a J. Young NMR tube for *ca*. 16 h. ^{1}H/^{19}F NMR spectroscopy, revealed a 6.20:1.00 ratio of secondary benzylic amination to primary benzylic amination, akin to the amination results from an equimolar solution of ethylbenzene and toluene (7.11:1.00 ratio). The formation of product was observed in 60(3) % yield over multiple trials with the mass balance attributed to formation of the cupric anilide species.

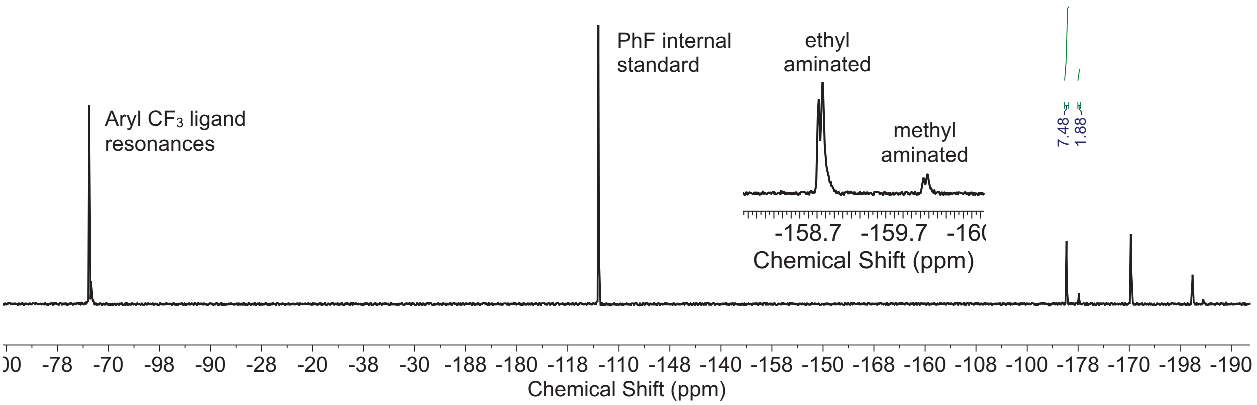

Figure S47. Representative ^{19}F NMR spectrum (470 MHz, C_6D_6) of crude 4-ethyltoluene amination reaction, revealing a mixture of primary benzylic and secondary benzylic aminated products. Inspect depicts truncated ^{19}F NMR spectrum (470 MHz, C_6D_6).

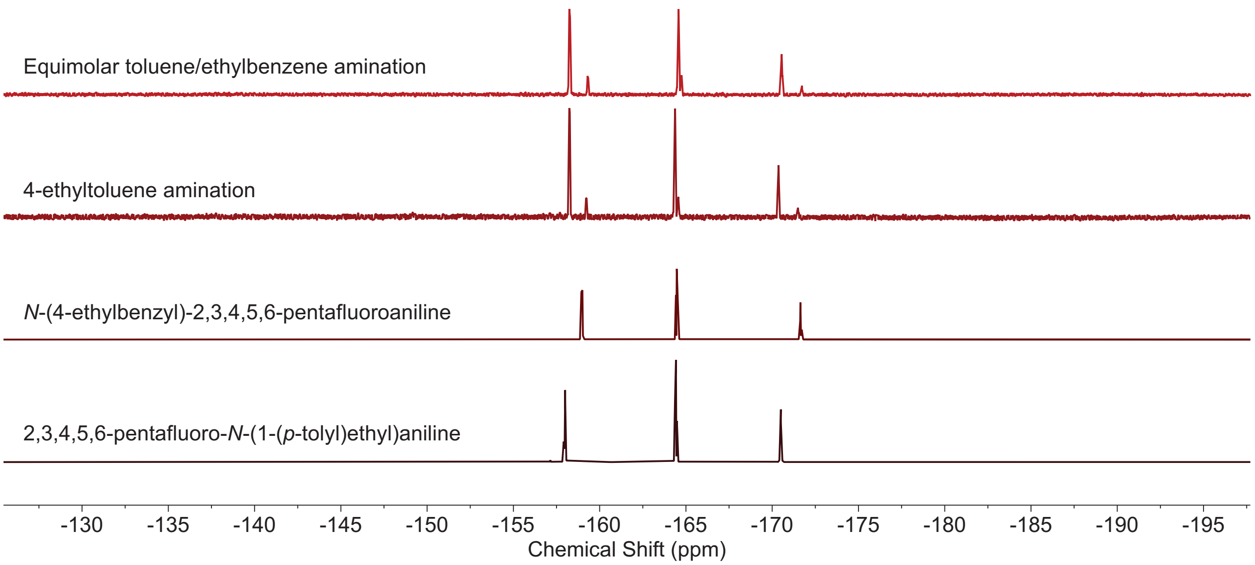

Figure S48. Stacked ^{19}F NMR spectra (470 MHz, C_6D_6) of 2,3,4,5,6-pentafluoro-*N*-(1-(*p*-tolyl)ethyl)aniline (*bottom*) and *N*-(4-ethylbenzyl)-2,3,4,5,6-pentafluoroaniline (*lower middle*), juxtaposed to crude 4-ethyltoluene amination reaction (*upper middle*) and competition amination reaction of equimolar toluene/ethylbenzene (*top*). The preference for amination at the more sterically congested, weaker C–H bond reveals the amination preference to be enthalpically driven. Crude spectra are obtained from (^{Ar_F}L)Cu (**4**) amination with $C_6F_5N_3$, and organic products are produced through independent synthesis.

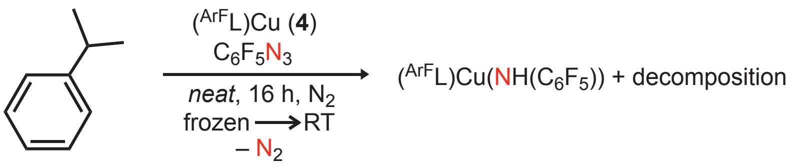

Competition: Tertiary Benzylic Amination *vs* Primary Aliphatic Amination. *All manipulations were conducted using silanized glassware*. In a dinitrogen-filled drybox, pentafluorophenyl azide ($C_6F_5N_3$; 0.002 g, 0.010 mmol, 1.0 equiv.) was dissolved in cumene (0.7 mL) with fluorobenzene as an internal standard. The mixture frozen in a liquid-nitrogen chilled cold well. Upon thawing, the mixture was added to solid (^{ArF}L)Cu (**4**) (0.010 g, 0.010 mmol, 1.0 equiv.) and transferred to a J. Young NMR tube for *ca*. 16 h. Upon consumption of the aryl azide, the resulting ^{19}F NMR spectrum displayed minimal organic ^{19}F NMR active species, with a broad ligand-containing species assigned as the doublet cupric anilide species. The inability to functionalize cumene is attributed to the sterically precluded tertiary C–*H* as well as the relatively inert primary C–*H* bond.

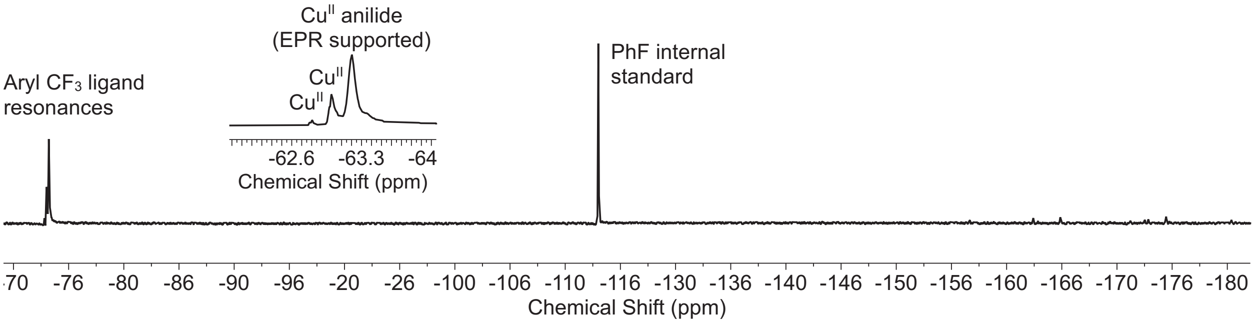

Figure S49. Representative ^{19}F NMR spectrum (470 MHz, C_6D_6) of crude cumene amination reaction, revealing no significant organic aryl C–*F* resonances. Inspect depicts truncated ^{19}F NMR spectrum (470 MHz, C_6D_6) showcasing broad ^{19}F ligand resonances attributed to the paramagnet Cu^{II} center, supported by EPR.

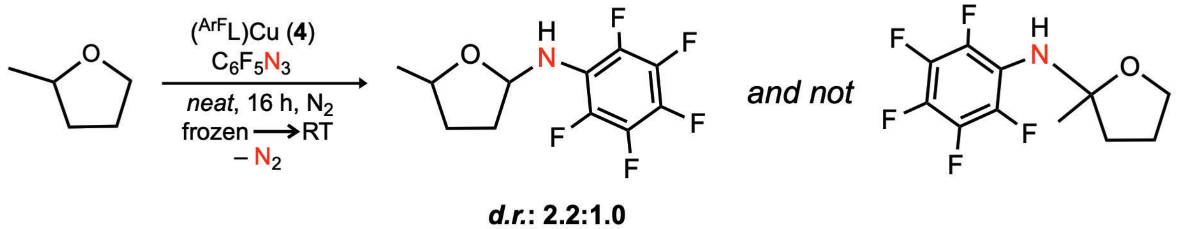

Competition: Secondary Ethereal Amination *vs* Tertiary Ethereal Amination. *All manipulations were conducted using silanized glassware.* In a dinitrogen-filled drybox, pentafluorophenyl azide ($C_6F_5N_3$; 0.002 g, 0.010 mmol, 1.0 equiv.) was dissolved in 2–methyltetrahydrofuran (0.7 mL). The mixture frozen in a liquid-nitrogen chilled cold well. Upon thawing, the mixture was added to solid (^{ArF}L)Cu (**4**) (0.010 g, 0.010 mmol, 1.0 equiv.) and transferred to scintillation vial for *ca*. 1 h. Following removal of solvent, the crude reaction was analyzed by multinuclear $^1H/^{19}F$ NMR spectroscopy to reveal exclusive amination of the secondary ethereal C–*H* bond in quantitative yield (diastereomeric ratio: 2.2:1).

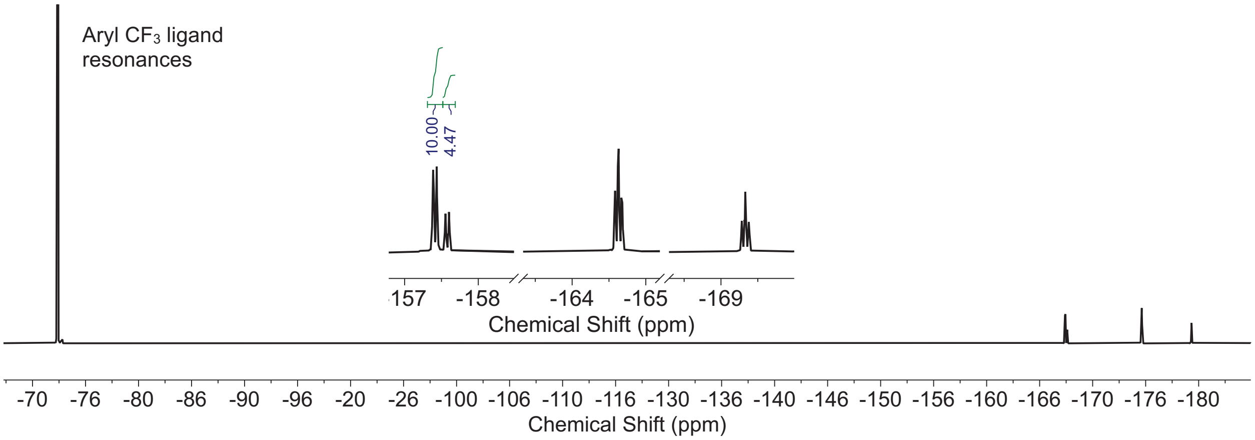

Figure S50. Representative ^{19}F NMR spectrum (470 MHz, C_6D_6) of crude 2–methyltetrahydrofuran amination reaction, revealing diastereoselective amination of the secondary ethereal C–H bond. Inspect depicts truncated ^{19}F NMR spectrum (470 MHz, C_6D_6) highlighting two sets of similar ^{19}F resonances, attributed to diastereomers.

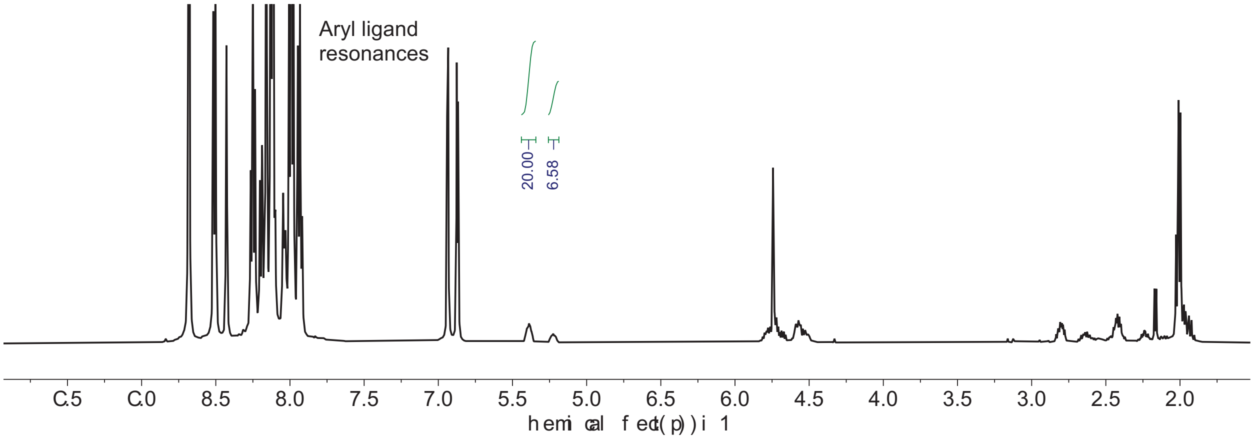

Figure S51. Representative ^1H NMR spectrum (500 MHz, C_6D_6) of crude 2–methyltetrahydrofuran amination reaction, revealing diastereoselective amination of the secondary ethereal C–*H* bond.

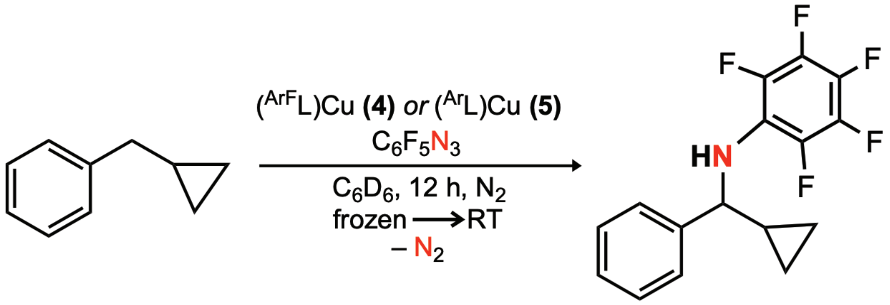

Radical Clock Amination. *All manipulations were conducted using silanized glassware*. In a dinitrogen-filled drybox, a precise quantity of pentafluorophenyl azide (0.004 g, 0.019 mmol, 1.1 equiv.) and (cyclopropylmethyl)benzene[24] (0.070 g, 0.526 mmol, 30.0 equiv.) were combined in C_6D_6 (0.7 mL) and frozen within a liquid-nitrogen chilled cold well. Upon thawing, the mixture was added to solid catalyst (0.018 mmol), either (^{ArF}L)Cu (**4**) or (^{Ar}L)Cu (**5**), and subsequent transferred to a J. Young NMR tube. After *ca*. 16 h, the mixture was checked by ^{19}F NMR spectroscopy to ensure complete consumption of $C_6F_5N_3$ and to quantify the relative ratios of aminated products, which were prepared independently as authentic ^{19}F NMR spectroscopy references.

The above reaction protocol was repeated with fluorobenzene as an internal standard to assess product yield. In both reaction mixtures, the only ^{19}F NMR active species was *N*-(cyclopropyl(phenyl)methyl)-2,3,4,5,6-pentafluoroaniline, signaling C–H amination without the corresponding ring-opened species. The non-quantitative yield (55(2) %) is attributed to formation of cupric anilide byproduct, which has been previously characterized as a ^{19}F NMR silent species.[3]

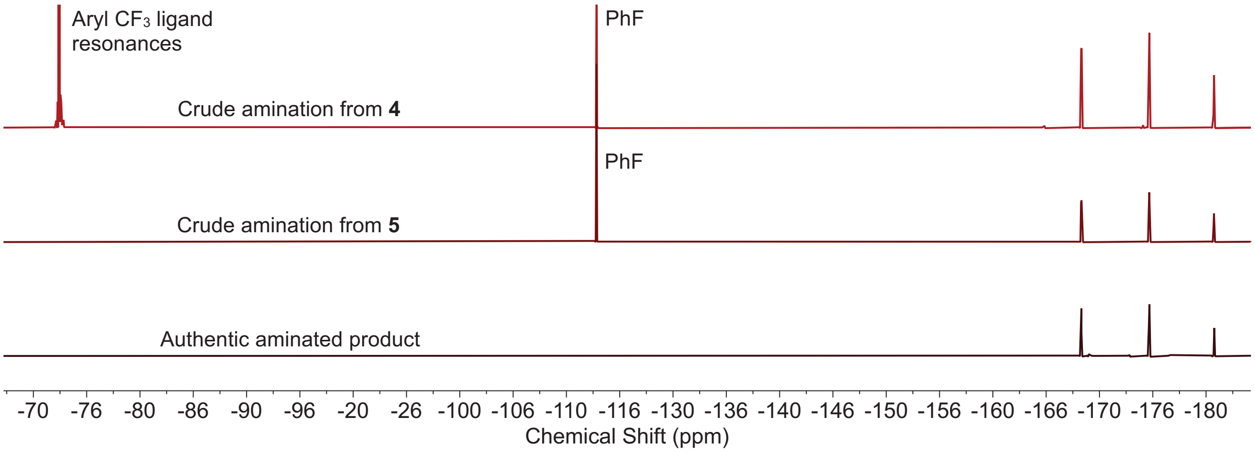

Figure S52. Crude ^{19}F NMR spectra (470 MHz, C_6D_6) of (cyclopropylmethyl)benzene amination with $C_6F_5N_3$ and (^{ArF}L)Cu (**4**) (*top*) and (^{Ar}L)Cu (**5**) (*middle*), juxtaposed with an authentic sample of *N*-(cyclopropyl(phenyl)methyl)-2,3,4,5,6-pentafluoroaniline (*bottom*), illustrating no evidence of ring-opening.

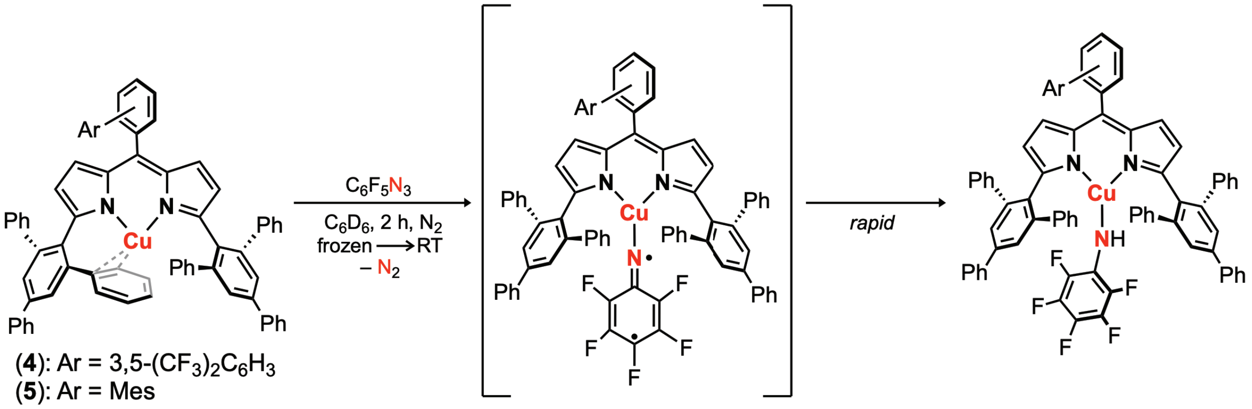

Stoichiometric Background Decomposition Measurement. *All manipulations were conducted using silanized glassware.* In a dinitrogen-filled drybox, excess pentafluorophenyl azide ($C_6F_5N_3$; 0.009 g, 0.045 mmol, 3.0 equiv.) with fluorobenzene (internal standard) was dissolved in C_6D_6 (0.20 mL) and added to a J. Young NMR tube. Additional C_6D_6 (0.20 mL) was used to ensure complete transfer of the azide solution. The solution was frozen in a liquid-nitrogen chilled cold well. Separately, (^{Ar_F}L)Cu (**4**) or (^{Ar_F}L)Cu (**5**) (0.015 mmol, 1.0 equiv.) was dissolved in C_6D_6 (0.2 mL) and frozen in the cold well. Once frozen, **4** or **5** was removed from the cold well and rapidly added to the J. Young tube. Addition thawing C_6D_6 (0.1 mL) was used to ensure complete transfer of **4** or **5** such that the total volume of tetrahydrofuran in the J. Young NMR tube was exactly 0.70 mL. The J. Young NMR tube was sealed, *rapidly* exported from the drybox, and quickly plunged into a dry ice/acetone bath to prevent thawing. The J. Young NMR tube was subsequently thawed and directly inserted into a pre-heated 500 MHz NMR spectrometer, followed by a ^{19}F NMR array collection (30 s intervals). For data processing of **4**, the integration values of unreacted **4** were normalized to the internal standard and compared against a broad ligand-containing resonance attributed to (^{Ar_F}L)Cu(NH(C_6F_5)) based on EPR analysis. For **5**, only consumption of aryl azide was monitored.

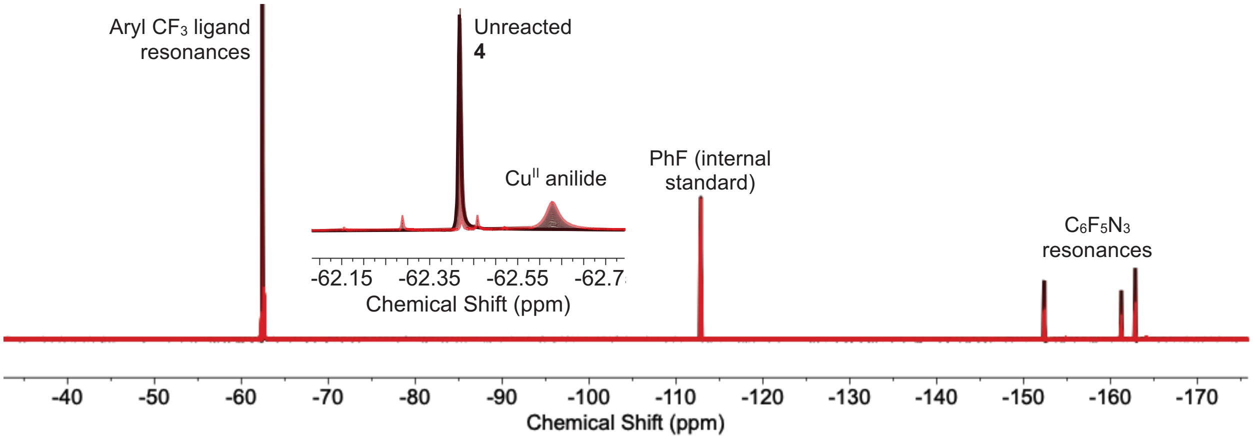

Figure S53. Superimposed ^{19}F NMR spectra (470 MHz, C_6D_6) time course (brown → red) revealing consumption of (^{Ar_F}L)Cu (**4**) and C_6F_5N without formation of new diamagnetic fluorine-containing species. Inspect depicts superimposed truncated ^{19}F NMR spectra (470 MHz, C_6D_6) of a ligand aryl CF_3 resonances, showcasing Cu^{II} paramagnetic species furnished upon stoichiometric consumption of $C_6F_5N_3$ in the absence of substrate.

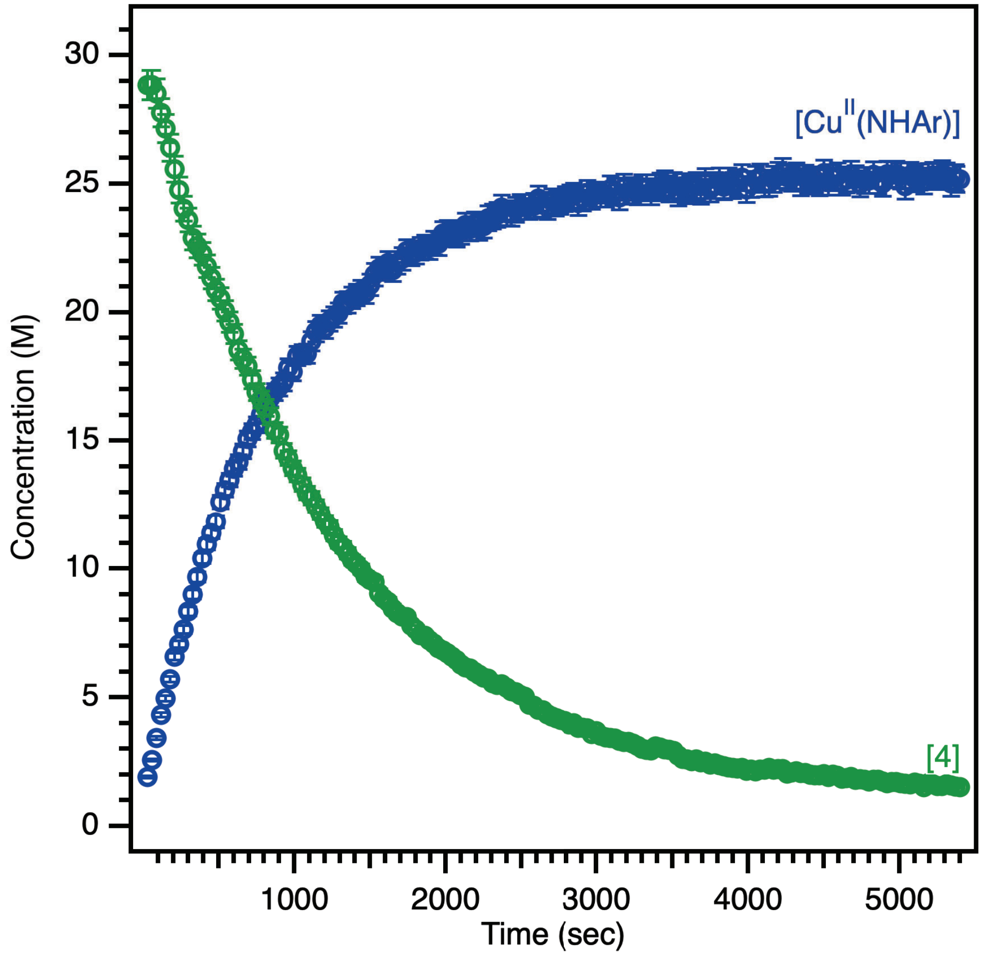

Figure S54. Kinetic monitoring of (^{ArF}L)Cu (**4**) concentration (*green*) upon addition of $C_6F_5N_3$ in C_6D_6, resulting in formation of a Cu^{II} catalytically inactive species (*blue*).

Catalytic Mechanistic Analysis.

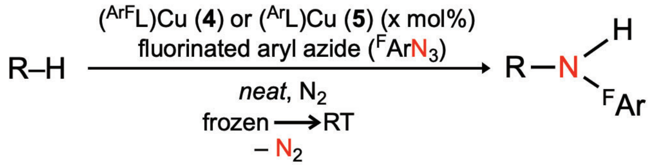

Catalytic Substrate Amination. *All manipulations were conducted using silanized glassware.* In a dinitrogen-filled drybox, aryl azide was combined with substrate (0.35 mL) and an internal standard (fluorobenzene) within a J. Young NMR tube. The mixture was analyzed by ^{19}F NMR spectroscopy to quantify initial ^{19}F integration values for unreacted aryl azide. To the thawing solution in the J. Young NMR tube was added a thawing solution of either (^{ArF}L)Cu (**4**) or (^{Ar}L)Cu (**5**) in substrate (0.35 mL). The J. Young NMR tube was inverted and monitored by ^{19}F NMR spectroscopy to quantify the formation of product until no additional consumption of aryl azide was consumed. This procedure was repeated in duplicate or triplicate with average yields reported. In a hypothetic quantitative C–H amination reaction, the integration values of the product resonances would equal those of the unreacted aryl azide resonances relative to the internal standard. Average yields are quantified by ^{19}F NMR spectroscopy relative to fluorobenzene, which was fixed at an integration value of 100.0 units during analysis. In amination reactions with **4**, inspection and integration of (^{ArF}L) ligand resonances allowed for assessment and quantification of (^{ArF}L) containing species in solution. Independent synthesis, GC/MS, and comparison of ^{1}H and ^{19}F NMR resonances with reported literature values corroborated organic product assignments.[20]

Table S4. Catalytic Substrate Amination Yields.[a]

Substrate	Catalyst (loading)	Aryl Azide	Avg. Yield (^{19}F NMR)[b]
Indane	**4** (5 mol%)	$C_6F_5N_3$	64(1) %
Indane	**5** (5 mol%)	$C_6F_5N_3$	96(1) %
2–MeTHF	**4** (5 mol%)	$C_6F_5N_3$	60(2) %
2–MeTHF	**5** (0.5 mol%)	$C_6F_5N_3$	> 99 %
Et_2O[c]	**4** (5 mol%)	$C_6F_5N_3$	80(5) %
THF	**4** (5 mol%)	$C_6F_5N_3$	98(2) %
THF	**5** (5 mol%)	$C_6F_5N_3$	> 99 %
THF	**4** (5 mol%)	(4–MeO_2C)$C_6F_4N_3$	94(1) %
THF	**5** (0.5 mol%)	(4–MeO_2C)$C_6F_4N_3$	94(1) %
THF	**4** (5 mol%)	(4–CF_3)$C_6F_4N_3$	94(2) %
THF	**5** (0.5 mol%)	(4–CF_3)$C_6F_4N_3$	> 99 %
THF[b]	**4** (0.1 mol%)	$C_6F_5N_3$	95(2) %
THF[b]	**5** (0.1 mol%)	$C_6F_5N_3$	> 99 %

[a]Triplicate runs. Secondary species were ^{19}F silent Cu^{II} species and/or unreacted aryl azide. [b]The reaction was allowed to proceed for 36 h. [c]The hemiaminal decomposed under attempted isolation and purification, which has been reported.[25] Partial characterization of *N*-(1-ethoxyethyl)-2,3,4,5,6-pentafluoroaniline is as follows: ^{1}H NMR (500 MHz, C_6D_6): δ 4.77 (br, 1*H*, amine N–*H*), 3.61 (m, 1*H*, tertiary C–*H*), 3.38 (m, 1*H*, secondary C–*H*), 3.20 (m, 1*H*, secondary C–*H*), 1.04 – 1.13 (overlapping 6*H* from ethyl terminal C–*H*). ^{19}F NMR (470 MHz, C_6D_6): δ –157.37 (m, 2*F*, fluorinated aryl C–*F*), –165.23 (m, 2*F*, fluorinated aryl C–*F*), –171.47 (m, 1*F*, fluorinated aryl C–*F*).

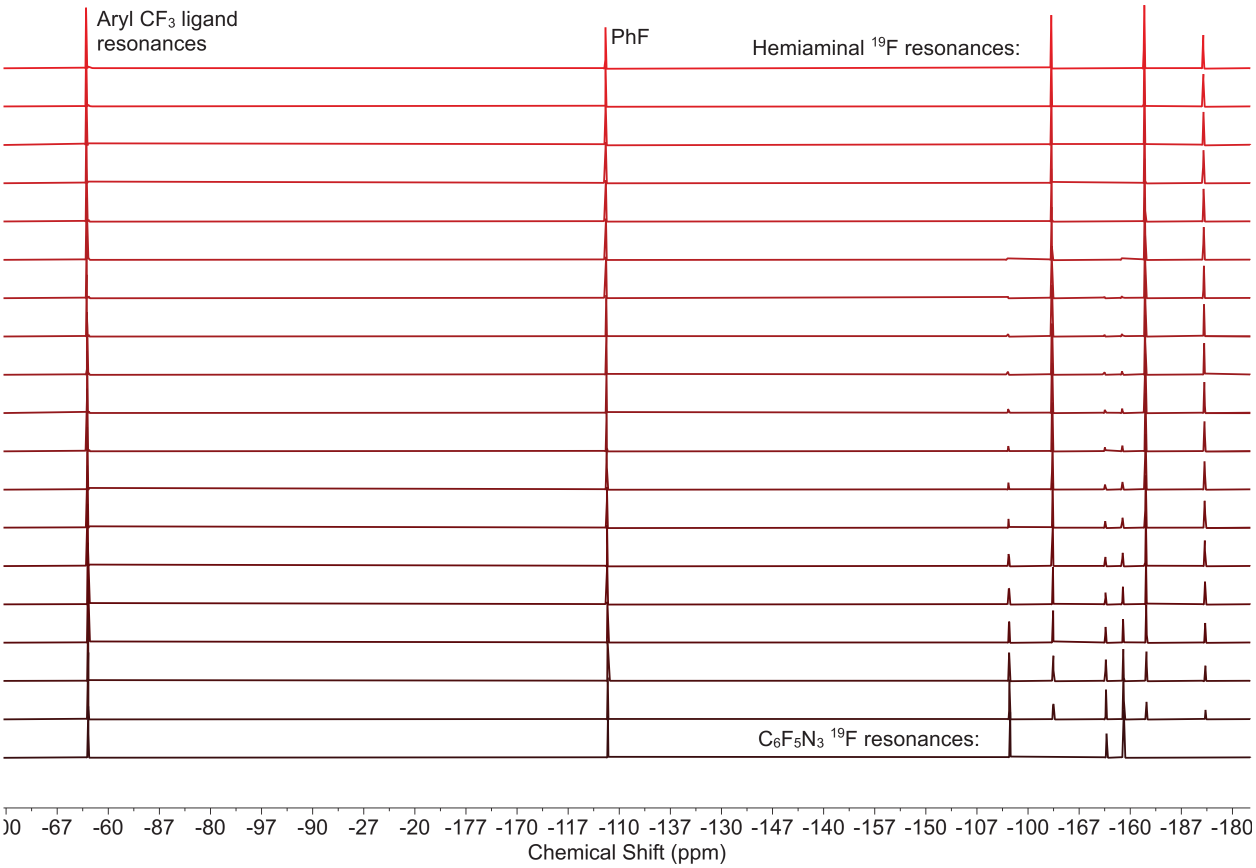

Figure S55. Representative ^{19}F NMR kinetics array (470 MHz, C_6D_6) depicting (^{ArF}L)Cu (**4**) amination (10 mol %) of tetrahydrofuran. Each entry represents a six-minute acquisition (total time, *ca*. 2 h), revealing consumption of $C_6F_5N_3$ (*bottom*) to afford the corresponding hemiaminal (*top*) in > 98 % yield based on integration relative to the internal standard (fluorobenzene).

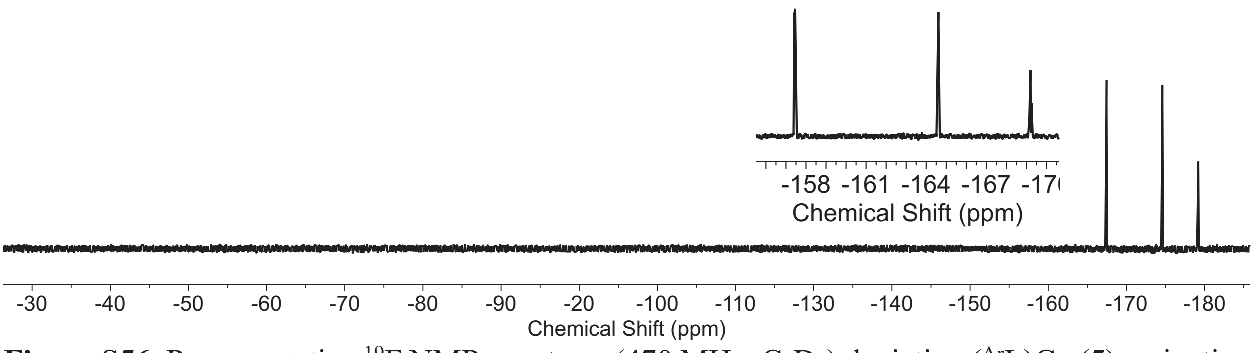

Figure S56. Representative ^{19}F NMR spectrum (470 MHz, C_6D_6) depicting (^{Ar}L)Cu (**5**) amination (0.1 mol %) of tetrahydrofuran after 36 h. Inspect depicts truncated ^{19}F NMR spectrum (470 MHz, C_6D_6) showcasing broad the absence of ^{19}F NMR active secondary species.

Catalytic Kinetic Isotope Effect (KIE) Measurements

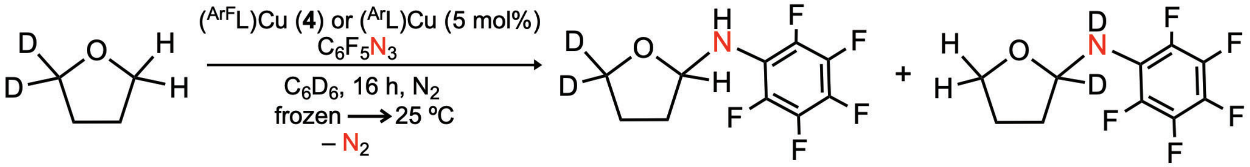

Intramolecular Catalytic 2,2–d_2–tetrahydrofuran Competition Kinetic Isotope Effect (KIE) Measurement. *All manipulations were conducted using silanized glassware.* In a dinitrogen-filled drybox, pentafluorophenyl azide ($C_6F_5N_3$; 0.023 g, 0.112 mmol, 20.0 equiv.) was combined with 2,2–d_2-tetrahydrofuran (0.101 g, 1.393 mmol, 250.0 equiv.) and C_6D_6 (0.7 mL). The mixture was frozen in a liquid nitrogen-chilled cold well. Upon thawing, the solution was added to a scintillation vial containing solid (**4**) or (^{Ar}L)Cu (**5**) (0.006 mmol, 1.0 equiv.) and allowed to stir. After 16 h, the pink solution was filtered through a pad of Celite, followed by removal of solvent *in vacuo*. The mixture was checked by ^{19}F NMR spectroscopy, followed by determination of the KIE value through ^{1}H NMR spectroscopy.

Note I: In our hands, the literature preparation of 2,2–d_2-tetrahydrofuran (d_2–THF) was unsuccessful, ultimately requiring the $LiBD_4$-mediated reduction to be conducted at 125 ºC for 48 h to observe majority consumption (albeit incomplete) of gamma-butyrolactone.[26] Following aqueous H_3PO_4-mediated cyclization, d_2-THF could readily be separated from impurities through distillation, followed by drying with NaK in a dinitrogen-filled drybox (***caution!!*** The addition of NaK to distilled d_2-THF is highly exothermic, attributed to presence of trace water. This desiccation should be performed through dropwise addition to thawing samples of d_2–THF).

The calculated KIE value for **4** was 4.7(1).
The calculated KIE value for **5** was 8.1(1).

Derivative of KIE Formula for 2,2–d_2-tetrahydrofuran amination: Assigning x as the relative concentration of [C–*H*] activated to [C–*D*] activated 2,2–d_2-tetrahydrofuran and γ as the ratio of a unique β–ethereal proton resonance to the summed α–ethereal proton resonances (which is evaluated by ^{1}H NMR spectroscopy), and we arrive at the following two equations (eq. 6, eq. 7).

$$KIE = \frac{k_H}{k_D} = \frac{[[\mathrm{C}-H]\ activated\ pdt]}{[[\mathrm{C}-D]\ activated\ pdt]} = \frac{[C-H]\ activated\ pdt}{[\mathrm{C}-D]\ activated\ pdt} \equiv x \qquad (6)$$

$$\gamma = \frac{\Sigma(unique\ \beta-ethereal\ {}^1H)}{\Sigma(\alpha-ethereal\ {}^1H)} = \frac{x*1+1*1}{x*1+1*2} = \frac{x+1}{x+2} \Rightarrow \mathbf{KIE} = \frac{\mathbf{2\gamma - 1}}{\mathbf{1-\gamma}} \qquad (7)$$

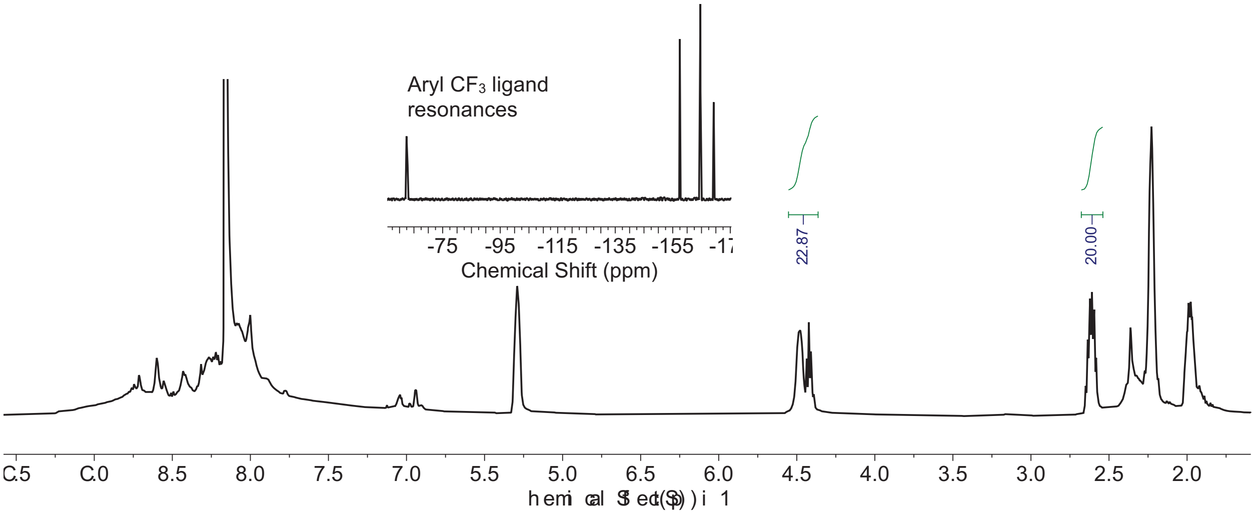

Figure S57. Crude ^{1}H NMR spectrum (500 MHz, C_6D_6) of (^{ArF}L)Cu (**4**) amination with $C_6F_5N_3$ using 2,2–d_2-tetrahydrofuran as the substrate. Integrated resonances are summed α–ethernal C–H resonances (*left*) and a unique β–ethereal proton resonance (*right*). Inset depicts ^{19}F NMR spectrum (470 MHz, C_6D_6) of crude reaction mixture, illustrating consumption of $C_6F_5N_3$ and the absence of secondary species.

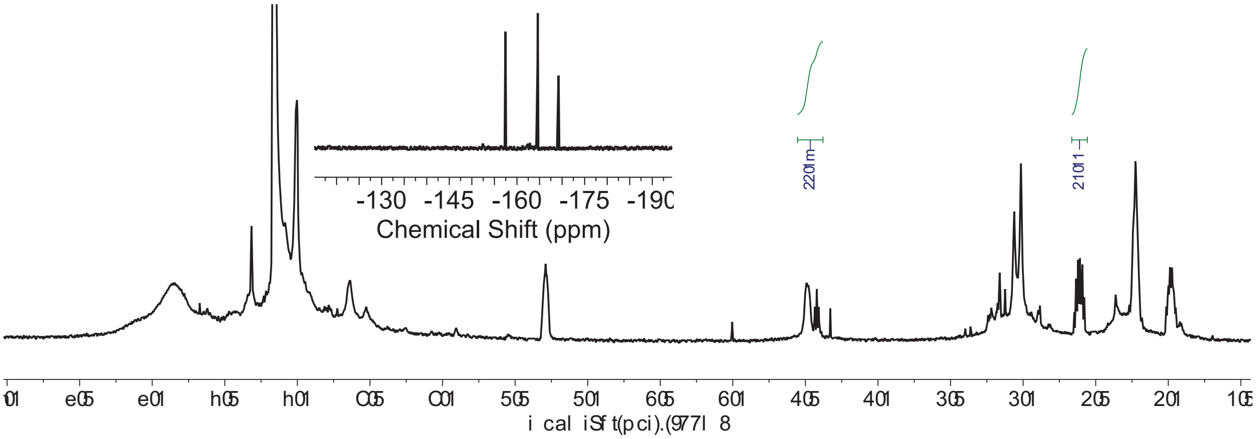

Figure S58. Crude ^{1}H NMR spectrum (500 MHz, C_6D_6) of (^{Ar}L)Cu (**5**) amination with $C_6F_5N_3$ using 2,2–d_2-tetrahydrofuran as the substrate. Integrated resonances are summed α–ethernal C–H resonances (*left*) and a unique β–ethereal proton resonance (*right*). Inset depicts ^{19}F NMR spectrum (470 MHz, C_6D_6) of crude reaction mixture, illustrating consumption of $C_6F_5N_3$ and the absence of secondary species.

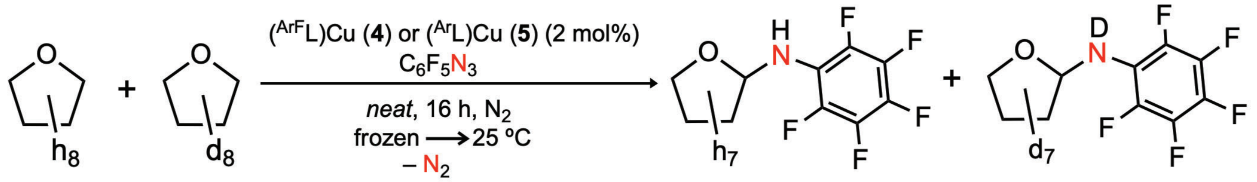

Intermolecular Catalytic h_8–tetrahydrofuran/d_8–tetrahydrofuran Competition Kinetic Isotope Effect (KIE) Measurement. *All manipulations were conducted using silanized glassware.* In a dinitrogen-filled drybox, pentafluorophenyl azide ($C_6F_5N_3$; 0.020 g, 0.195 mmol, 50.0 equiv.) was combined with an equimolar mixture of h_8–tetrahydrofuran (0.416 g, 5.769 mmol) and d_8–tetrahydrofuran (0.463 mmol, 5.769 mmol). The mixture was frozen in a liquid nitrogen-chilled cold well. Upon thawing, the solution was added to a scintillation vial containing solid (^{ArF}L)Cu (**4**) or (^{Ar}L)Cu (**5**) (0.006 mmol, 1.0 equiv.) and allowed to stir. After 16 h, the pink solution was filtered through a pad of Celite, followed by removal of solvent *in vacuo*. The residual solid was dissolved in C_6D_6, and an internal standard of 4-fluoroanisole (10 µL) was added. The mixture was checked by multinuclear NMR spectroscopy to determine the kinetic isotope effect (see derivation below).

The calculated KIE value for **4** was 6.2(2).
The calculated KIE value for **5** was 10.7(4).

Derivative of KIE Formula: As the ^{19}F NMR resonances are equivalent between C–H activated and C–D activated products, we note that the ^{19}F NMR resonances integrate to the combined summed quantity of C–H activated and C–D activated products per resonance. We define α as the ratio of the summed functionalized product value to the internal standard (IS) concentration of 4-fluoroanisole (displaying a single fluorine atoms), which is evaluated by ^{19}F NMR spectroscopy and normalized to the number of fluorine atoms per resonance (eq. 8).

$$\alpha = \frac{\Sigma(pdt\ ^{19}F)}{[\Sigma(IS\ ^{19}F)}= \frac{([\mathrm{C}\!-H]\ activated\ ^{19}F + [\mathrm{C}\!-D]\ activated\ ^{19}F)/(\#\ of\ equivalent\ ^{19}F\ atoms)}{(internal\ standard\ ^{19}F)/1} \quad (8)$$

The number of equivalent ^{19}F atoms is dependent on which fluorine resonances are selected for integration: *ortho* and *meta* resonances display two chemically equivalent ^{19}F atoms each, and the *para* resonance is due to a unique ^{19}F atom.

We define ε as the ratio of the C–*H* resonance of a unique β-ethereal C–*H* resonance to the normalized C–*H* resonance of the methoxy motif on the internal standard 4–fluoroanisole (exhibiting three equivalent protons), which is evaluated by ^{1}H NMR spectroscopy (eq. 9).

$$\varepsilon = \frac{([\mathrm{C}\!-H]\ activated\ ^{1}H)/(\#\ of\ equivalent\ \ ^{1}H\ atoms)}{(IS\ ^{1}H)/3} \quad (9)$$

We find the following, nothing that the activatable substrate remains in vast excess over the entire reaction such that the KIE can be approximated as a relative concentration (eq. 10):

$$KIE = \frac{k_H}{k_D} \approx \frac{\left[[\text{C}-H]\ activated\ pdt\right]}{\left[[\text{C}-D]\ activated\ pdt\right]} = \frac{[C-H]\ activated\ pdt}{[C-D]\ activated\ pdt}$$

$$= \frac{[\text{C}-H]\ activated\ pdt}{\Sigma\left(pdt\ ^{19}F\right) - \ [\text{C}-H]\ activated\ pdt} \ = \ \frac{\varepsilon}{\alpha - \varepsilon} \Rightarrow \mathbf{KIE} = \frac{\boldsymbol{\varepsilon}}{\boldsymbol{\alpha} - \boldsymbol{\varepsilon}} \qquad (10)$$

Worth noting is that the computed KIE is independent of (i) product yield, (ii) concentration of reagents in solution, and (iii) internal standard quantity, although non-zero quantity of internal standard is necessary.

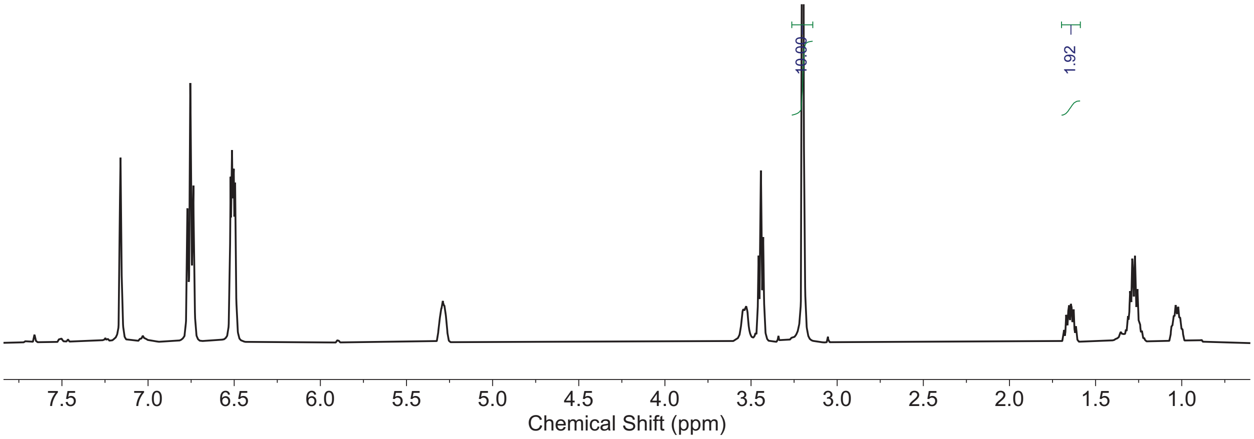

Figure S59. Crude ^{1}H NMR spectrum (500 MHz, C_6D_6) of (^{Ar_F}L)Cu (**4**) amination with $C_6F_5N_3$ using an equimolar solution of h_8–tetrahydrofuran and d_8–tetrahydrofuran as substrate. The highlighted resonances include a unique β-ethereal C–*H* resonance (*right*) and the methoxy resonance of the internal standard (*left*).

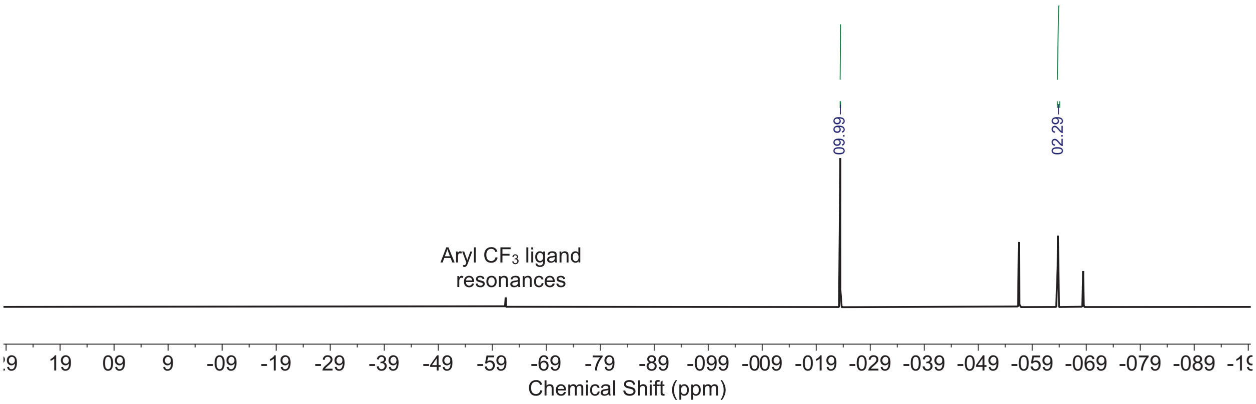

Figure S60. Crude ^{19}F NMR spectrum (500 MHz, C_6D_6) of (^{Ar_F}L)Cu (**4**) amination with $C_6F_5N_3$ using an equimolar solution of h_8–tetrahydrofuran and d_8–tetrahydrofuran as substrate. The highlighted resonances include the unique aryl C–*F* resonance of the internal standard (*left*) and a summed C–*F* resonance of the fluorinated hemiaminal (*right*).

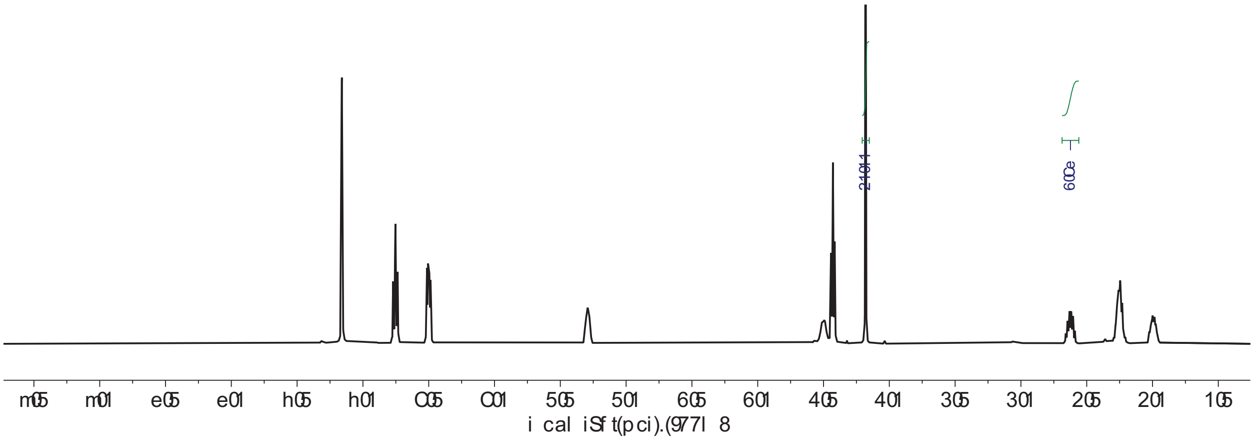

Figure S61. Crude ^{1}H NMR spectrum (500 MHz, C_6D_6) of (^{Ar}L)Cu (**5**) amination with $C_6F_5N_3$ using an equimolar solution of h_8–tetrahydrofuran and d_8–tetrahydrofuran as substrate. The highlighted resonances include a unique β-ethereal C–*H* resonance (*right*) and the methoxy resonance of the internal standard (*left*).

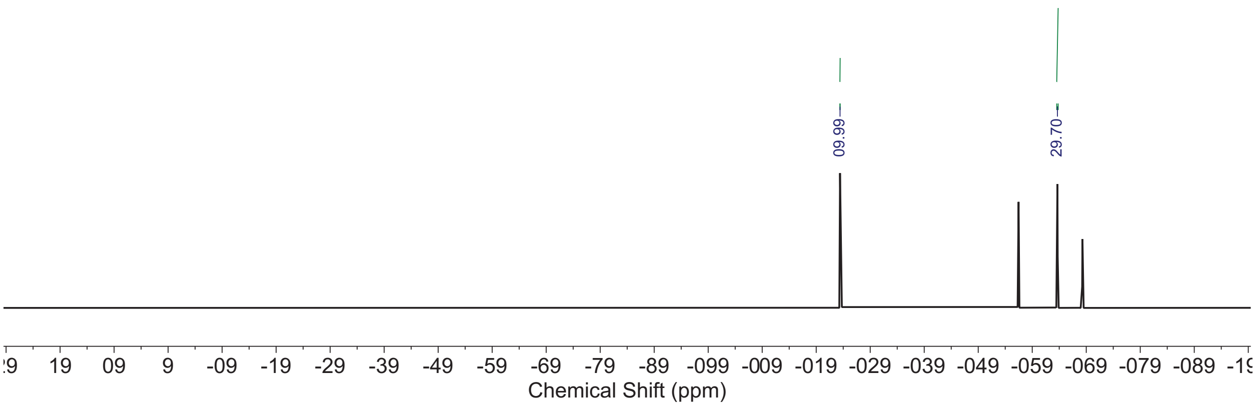

Figure S62. Crude ^{19}F NMR spectrum (500 MHz, C_6D_6) of (^{ArF}L)Cu (**4**) amination with $C_6F_5N_3$ using an equimolar solution of h_8–tetrahydrofuran and d_8–tetrahydrofuran as substrate. The highlighted resonances include the unique aryl C–*F* resonance of the internal standard (*left*) and a summed C–*F* resonance of the fluorinated hemiaminal (*right*).

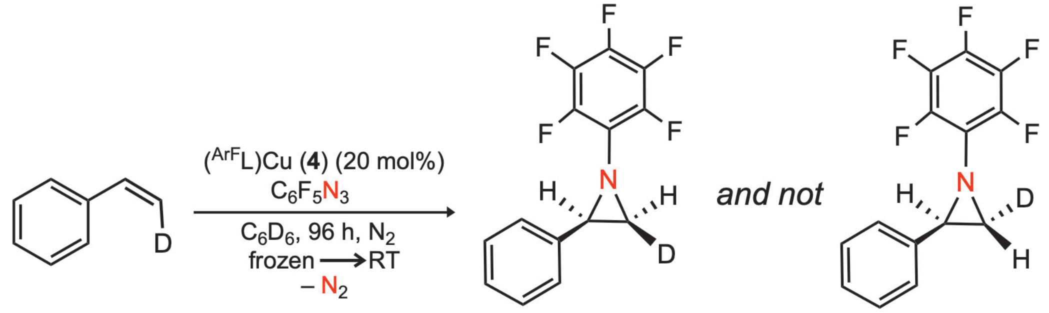

(Z)–β–deuterostyrene aziridination. *All manipulations were conducted using silanized glassware.* In a dinitrogen-filled drybox, pentafluorophenyl azide ($C_6F_5N_3$; 0.012 g, 0.049 mmol, 5.0 equiv.) was combined with (Z)–β–deuterostyrene[27] (0.010 g, 0.097 mmol, 10.0 equiv.) in C_6D_6 (0.7 mL). The mixture was frozen in a liquid nitrogen-chilled cold well. Upon thawing, the solution was added to solid (^{ArF}L)Cu (**4**) (0.010 g, 0.010 mmol, 0.2 equiv.) and transferred to a J. Young NMR tube. After 96 h, the pink solution was filtered through a pad of Celite, followed by removal of solvent *in vacuo*. The residual solid was dissolved in C_6D_6 and analyzed by multinuclear NMR spectroscopy, revealing formation of the corresponding aziridine by comparison to an authentic reference[18] with no $H_{\beta,cis}$/$H_{\beta,trans}$ scrambling based on 1.00:1.00 integration of H_α:$H_{\beta,trans}$.

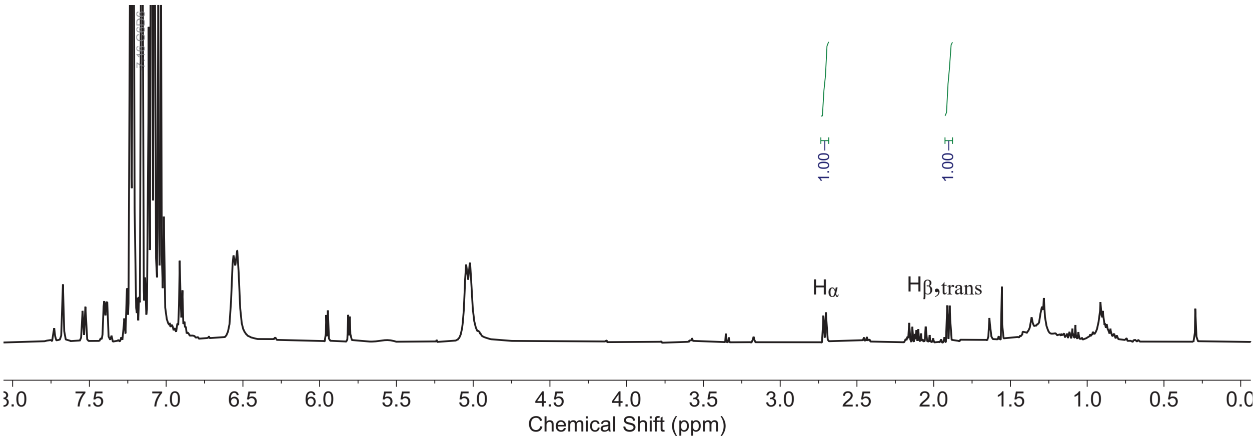

Figure S63. Crude ^{1}H NMR spectrum (500 MHz, C_6D_6) of (Z)–β–deuterostyrene aziridination with (^{ArF}L)Cu (**4**) and $C_6F_5N_3$, revealing stereoretention upon styrene aziridination.

Kinetic Analysis.

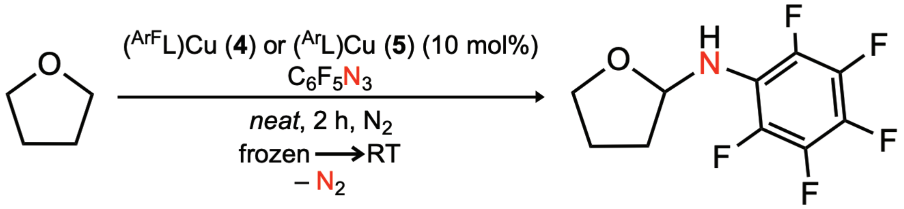

Effect of Catalyst Substation on Tetrahydrofuran Amination Rate. *All manipulations were conducted using silanized glassware.* In a dinitrogen-filled drybox, a precise quantity of pentafluorophenyl azide ($C_6F_5N_3$; 0.023 g, 0.195 mmol, 1.0 equiv.) with fluorobenzene (internal standard) was combined with tetrahydrofuran (0.20 mL) using a volumetric syringe and added to a J. Young NMR tube. Additional tetrahydrofuran (0.20 mL) was used to ensure complete transfer of the azide solution. The solution was frozen in a liquid-nitrogen chilled cold well. Separately, either (^{ArF}L)Cu (**4**) or (^{ArF}L)Cu (**5**) (0.010 mmol, 0.1 equiv.) was dissolved in tetrahydrofuran (0.2 mL) and frozen in the cold well. Once frozen, **4** or **5** was removed from the cold well and rapidly added to the J. Young tube. Addition thawing tetrahydrofuran (0.1 mL) was used to ensure complete transfer of **4** or **5** such that the total volume of tetrahydrofuran in the J. Young NMR tube was exactly 0.70 mL. The J. Young NMR tube was sealed, *rapidly* exported from the drybox, and quickly plunged into a liquid nitrogen bath to prevent thawing. The J. Young NMR tube was subsequently thawed and directly inserted into a pre-heated 500 MHz NMR spectrometer, followed by a ^{19}F NMR array collection (40 s intervals) for 2 h.

For data processing, the integration values of unreacted aryl azide and the hemiaminal were normalized to the internal standard. No significant degradation of **4** (98 % recovery of **4**) was calculated based on integration of aryl CF_3 resonances by ^{19}F NMR spectroscopy.

The rate of catalysis with **4** is 8.16(6) × 10^{-4} sec^{-1} ($t_{1/2}$ = 14.2 minutes).
The rate of catalysis with **5** is 4.34(9) × 10^{-3} sec^{-1} ($t_{1/2}$ = 2.7 minutes).

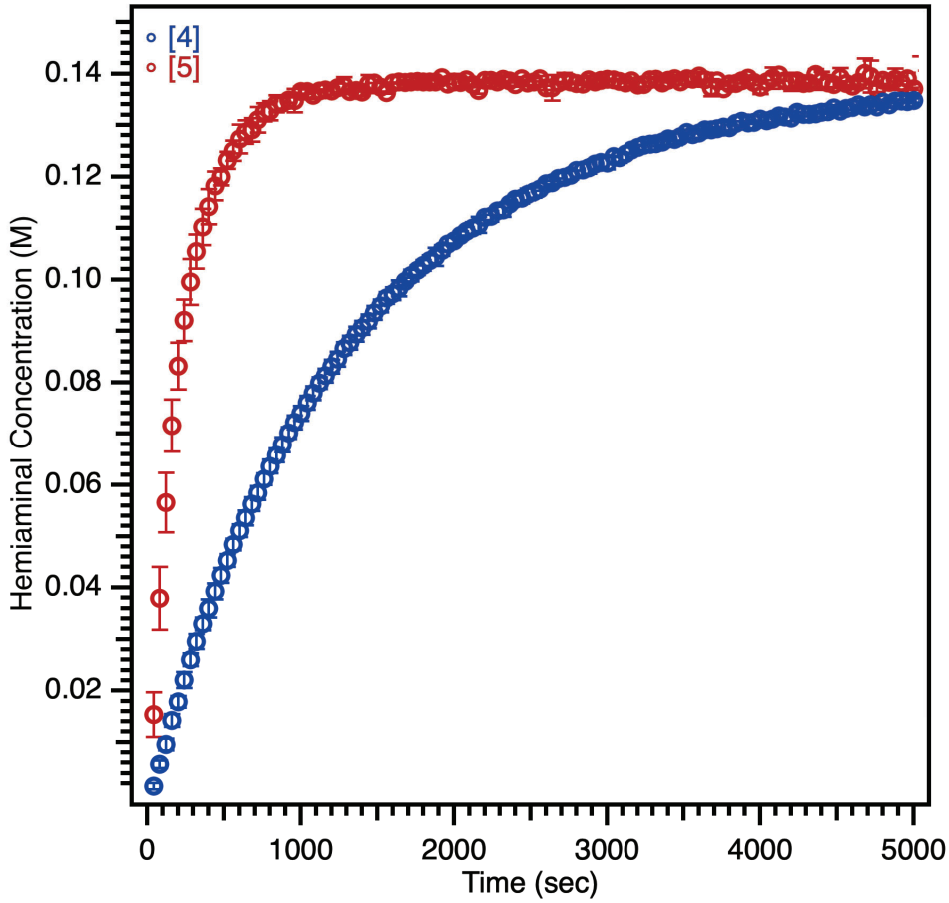

Figure S64. Comparison of tetrahydrofuran amination for 10 mol % (^{ArF}L)Cu (**4**) and (^{ArF}L)Cu (**5**), plotted as function of consumption.

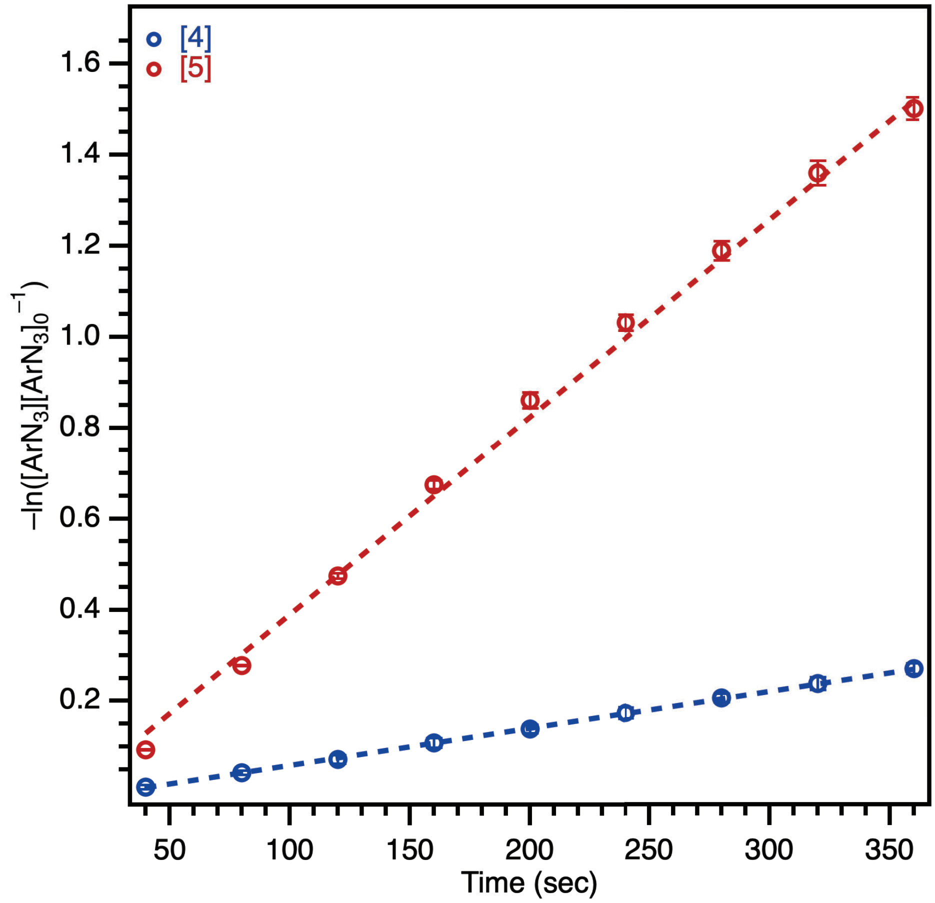

Figure S65. Comparison of tetrahydrofuran amination for 10 mol% (^{ArF}L)Cu (**4**) and (^{ArF}L)Cu (**5**), plotted as function of aryl azide consumption.

Table S5. Rate Constants for tetrahydrofuran amination by **4** and **5**.

Catalyst	Rate Constant (sec^{-1})
**4**	$8.16(6) \times 10^{-4}$
**5**	$4.34(9) \times 10^{-3}$

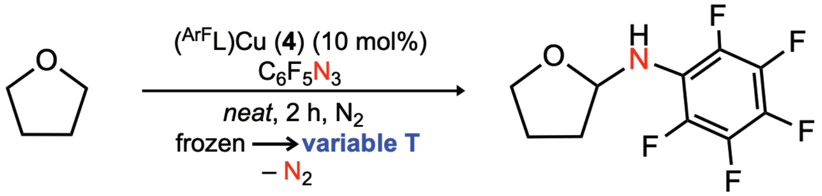

Erying Analysis. *All manipulations were conducted using silanized glassware*. In a dinitrogen-filled drybox, a precise quantity of pentafluorophenyl azide ($C_6F_5N_3$; 0.023 g, 0.195 mmol, 1.0 equiv.) with fluorobenzene (internal standard) was combined with tetrahydrofuran (0.20 mL) using a volumetric syringe and added to a J. Young NMR tube. Additional tetrahydrofuran (0.20 mL) was used to ensure complete transfer of the azide solution. The solution was frozen in a liquid-nitrogen chilled cold well. Separately, (^{ArF}L)Cu (**4**) (0.010 g, 0.010 mmol, 0.1 equiv.) was dissolved in tetrahydrofuran (0.2 mL) and frozen in the cold well. Once frozen, **4** was removed from the cold well and rapidly added to the J. Young tube (*NB*: the THF solution of **4** should freeze on the sides of the J. Young NMR tube and not dissolve the pre-frozen azide solution). Addition thawing tetrahydrofuran (0.1 mL) was used to ensure complete transfer of **4** such that the total volume of tetrahydrofuran in the J. Young NMR tube was exactly 0.70 mL. The J. Young NMR tube was sealed, *rapidly* exported from the drybox, and quickly plunged into a liquid nitrogen bath to prevent thawing. The J. Young NMR tube was subsequently thawed and directly inserted into a pre-heated 500 MHz NMR spectrometer, followed by a ^{19}F NMR array collection (40 s intervals) for 40 min.

For data processing, the integration values of unreacted aryl azide and the hemiaminal were normalized to the internal standard. Rate constants were measured using initial rates approach, monitoring consumption of $C_6F_5N_3$ to 10 % with no apparent degradation of **4** based on ^{19}F NMR spectroscopy.

Calculated thermodynamic values based on Erying Analysis are as follows, derived from the Erying equation ($\Delta H^‡$, $\Delta S^‡$) and the two-point form of the Arrhenius Equation (E_a):

$\Delta S^‡$ = –42(2) cal mol^{-1} K^{-1}
$\Delta H^‡$ = 9.2(2) kcal mol^{-1}
E_a = 9.8(1) kcal mol^{-1}

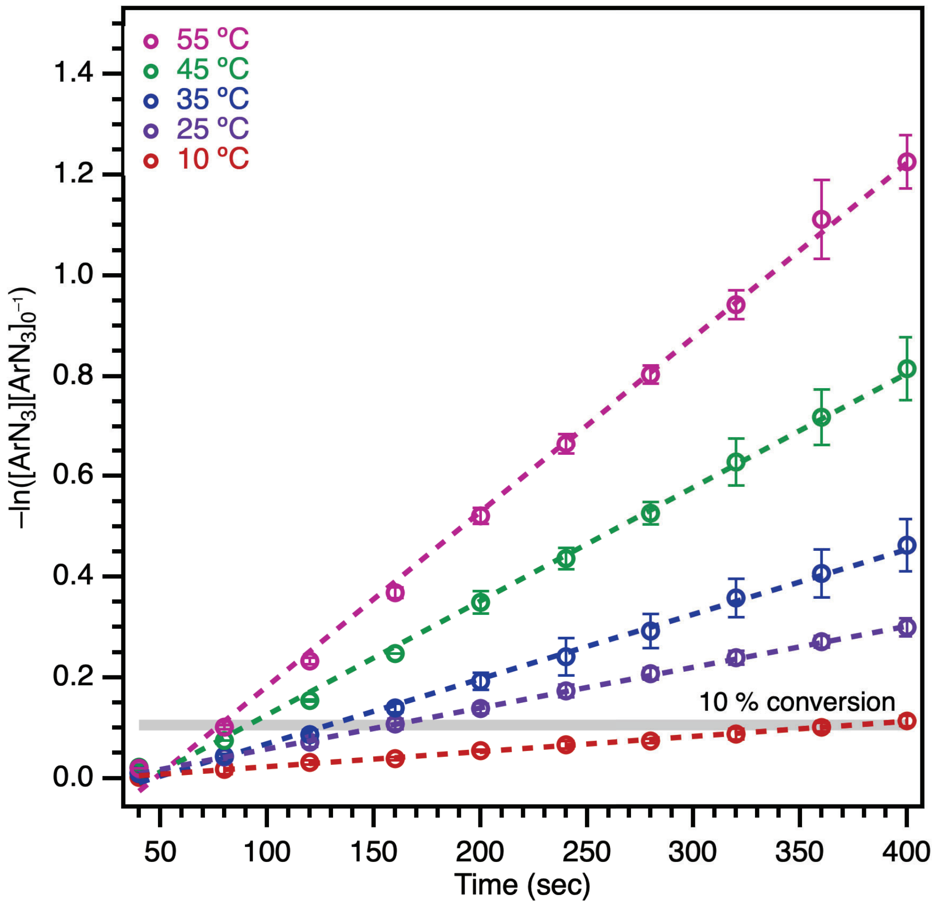

Figure S66. Erying analysis of tetrahydrofuran amination by (^{ArF}L)Cu (**4**) (10 mol%) at temperature measurements of 10 ºC (*red*), 25 ºC (*purple*), 35 ºC (*blue*), 45 ºC (*green*), and 55 ºC (pink).

Table S6. Rate Constants for Erying analysis of tetrahydrofuran amination.

Temperature	Rate Constant (sec^{-1})
10 ºC	$3.10(2) \times 10^{-4}$
25 ºC	$8.09(23) \times 10^{-4}$
35 ºC	$1.27(3) \times 10^{-3}$
45 ºC	$2.17(7) \times 10^{-3}$
55 ºC	$3.48(86) \times 10^{-3}$

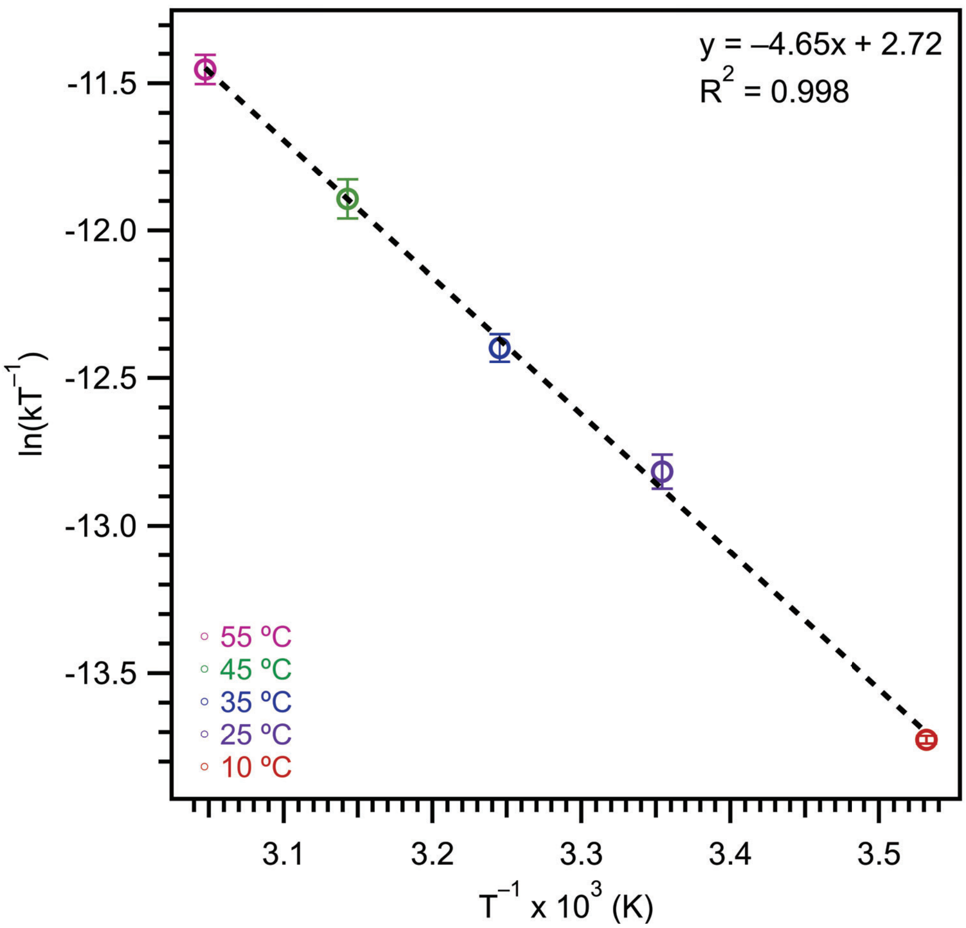

Figure S67. Erying analysis of tetrahydrofuran amination by (^{ArF}L)Cu (**4**) (10 mol%) at temperature measurements of 10 ºC (*red*), 25 ºC (*purple*), 35 ºC (*blue*), 45 ºC (*green*), and 55 ºC (pink).

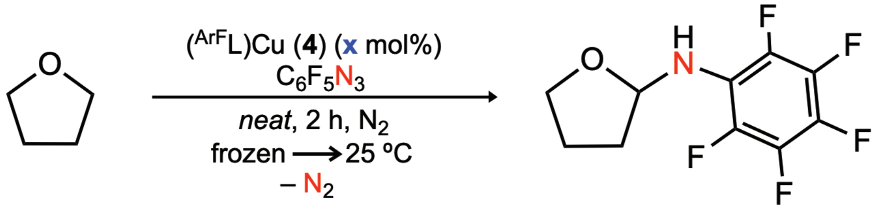

Cu–loading Analysis. *All manipulations were conducted using silanized glassware*. In a dinitrogen-filled drybox, a precise quantity of pentafluorophenyl azide ($C_6F_5N_3$; 0.023 g, 0.195 mmol, 1.0 equiv.) with fluorobenzene (internal standard) was combined with tetrahydrofuran (0.20 mL) using a volumetric syringe and added to a J. Young NMR tube. Additional tetrahydrofuran (0.20 mL) was used to ensure complete transfer of the azide solution. The solution was frozen in a liquid-nitrogen chilled cold well. Separately, the specified amount of (^{ArF}L)Cu (**4**) was dissolved in tetrahydrofuran (0.2 mL) and frozen in the cold well. Once frozen, **4** was removed from the cold well and rapidly added to the J. Young tube (*NB*: the THF solution of **4** should freeze on the sides of the J. Young NMR tube and not dissolve the pre-frozen azide solution). Addition thawing tetrahydrofuran (0.1 mL) was used to ensure complete transfer of **4** such that the total volume of tetrahydrofuran in the J. Young NMR tube was exactly 0.70 mL. The J. Young NMR tube was sealed, *rapidly* exported from the drybox, and quickly plunged into a liquid nitrogen bath to prevent thawing. The J. Young NMR tube was subsequently thawed and directly inserted into a pre-heated 500 MHz NMR spectrometer (25.0 ºC), followed by a ^{19}F NMR array collection (20 s intervals) for 2 h.

For data processing, the integration values of unreacted aryl azide and the hemiaminal were normalized to the internal standard. Rate constants were measured using initial rates approach, monitoring consumption of $C_6F_5N_3$ to 10 % with no apparent degradation of **4** based on ^{19}F NMR spectroscopy.

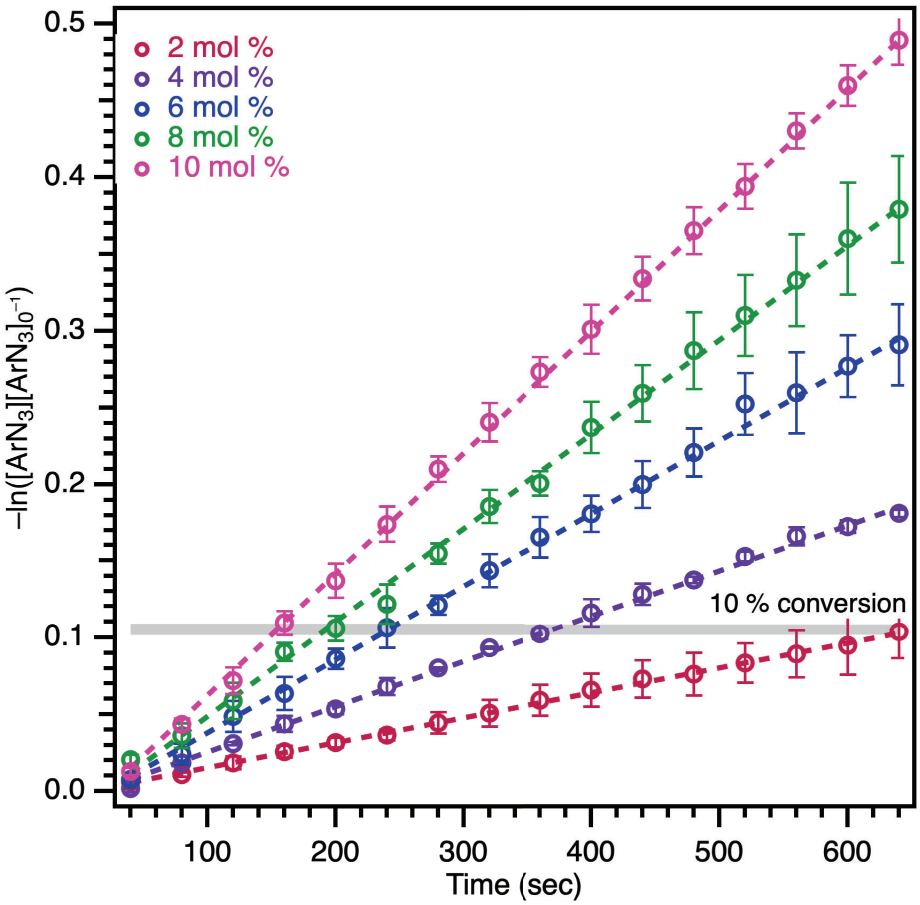

Figure S68. Kinetic analysis of tetrahydrofuran amination with (^{ArF}L)Cu (**4**) at variable Cu loadings: 2 mol% (*red*), 4 mol% (*purple*), 6 mol% (*blue*), 8 mol% (*green*), and 10 mol% (*pink*).

Table S7. Rate Constants for tetrahydrofuran amination with (^{ArF}L)Cu (**4**) at variable Cu loadings.

Cu Loading	Rate Constant (sec^{-1})
2 mol%	1.62(2) × 10^{-4}
4 mol%	3.12(5) × 10^{-4}
6 mol%	4.8(1) × 10^{-4}
8 mol%	6.3(3) × 10^{-4}
10 mol%	8.0(3) × 10^{-4}

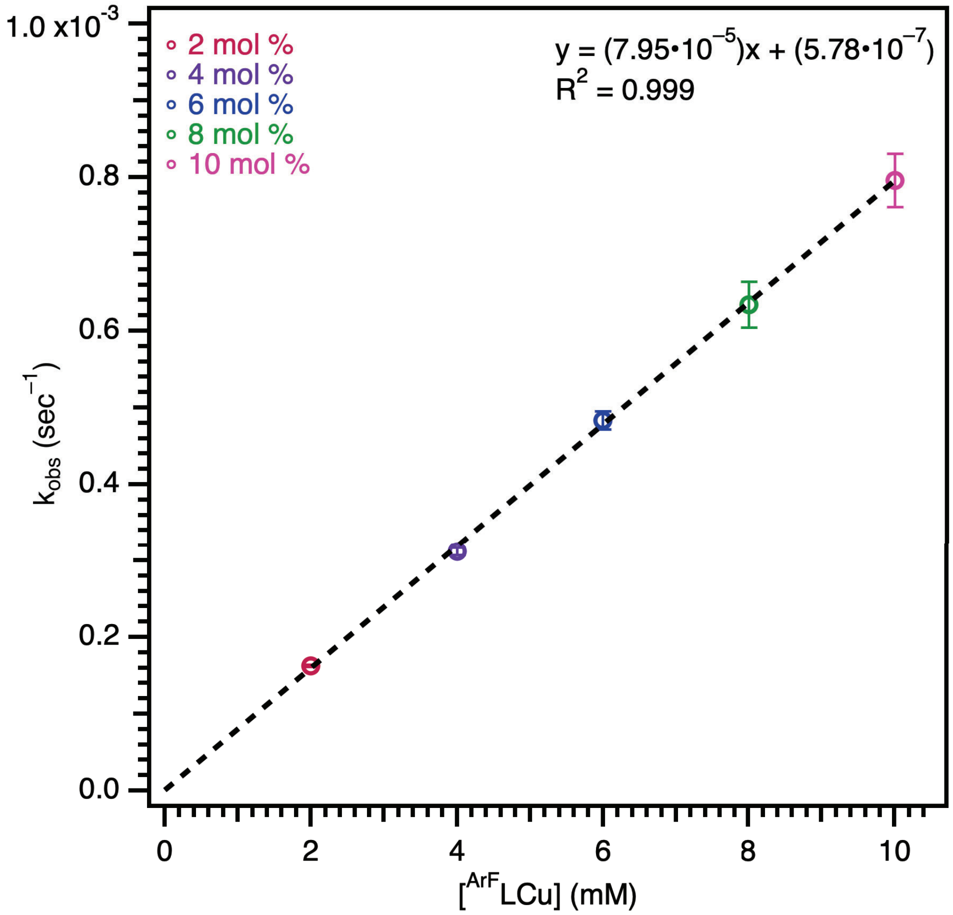

Figure S69. Kinetic analysis of tetrahydrofuran amination with (ArFL)Cu (**4**) at variable Cu loadings: 2 mol% (*red*), 4 mol% (*purple*), 6 mol% (*blue*), 8 mol% (*green*), and 10 mol% (*pink*). A first-order dependence on concentration of **4** is observed.

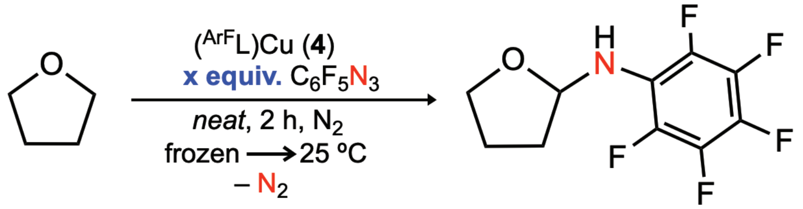

ArN$_3$–loading Analysis. *All manipulations were conducted using silanized glassware*. In a dinitrogen-filled drybox, a variable quantity of pentafluorophenyl azide with fluorobenzene (internal standard) was combined with tetrahydrofuran (0.20 mL) using a volumetric syringe and added to a J. Young NMR tube. Additional tetrahydrofuran (0.20 mL) was used to ensure complete transfer of the azide solution. The solution was frozen in a liquid-nitrogen chilled cold well. Separately, the specified amount of (^{ArF}L)Cu (**4**) (0.004 g, 0.004 mmol) was dissolved in tetrahydrofuran (0.2 mL) and frozen in the cold well. Once frozen, **4** was removed from the cold well and rapidly added to the J. Young tube (*NB*: the THF solution of **4** should freeze on the sides of the J. Young NMR tube and not dissolve the pre-frozen azide solution). Addition thawing tetrahydrofuran (0.1 mL) was used to ensure complete transfer of **4** such that the total volume of tetrahydrofuran in the J. Young NMR tube was exactly 0.70 mL. The J. Young NMR tube was sealed, *rapidly* exported from the drybox, and quickly plunged into a liquid nitrogen bath to prevent thawing. The J. Young NMR tube was subsequently thawed and directly inserted into a pre-heated 500 MHz NMR spectrometer (25.0 ºC), followed by a ^{19}F NMR array collection (20 s intervals) for 2 h.

For data processing, the integration values of unreacted aryl azide and the hemiaminal were normalized to the internal standard. Rate constants were measured using initial rates approach, monitoring consumption of $C_6F_5N_3$ to 10 % with no apparent degradation of **4** based on ^{19}F NMR spectroscopy.

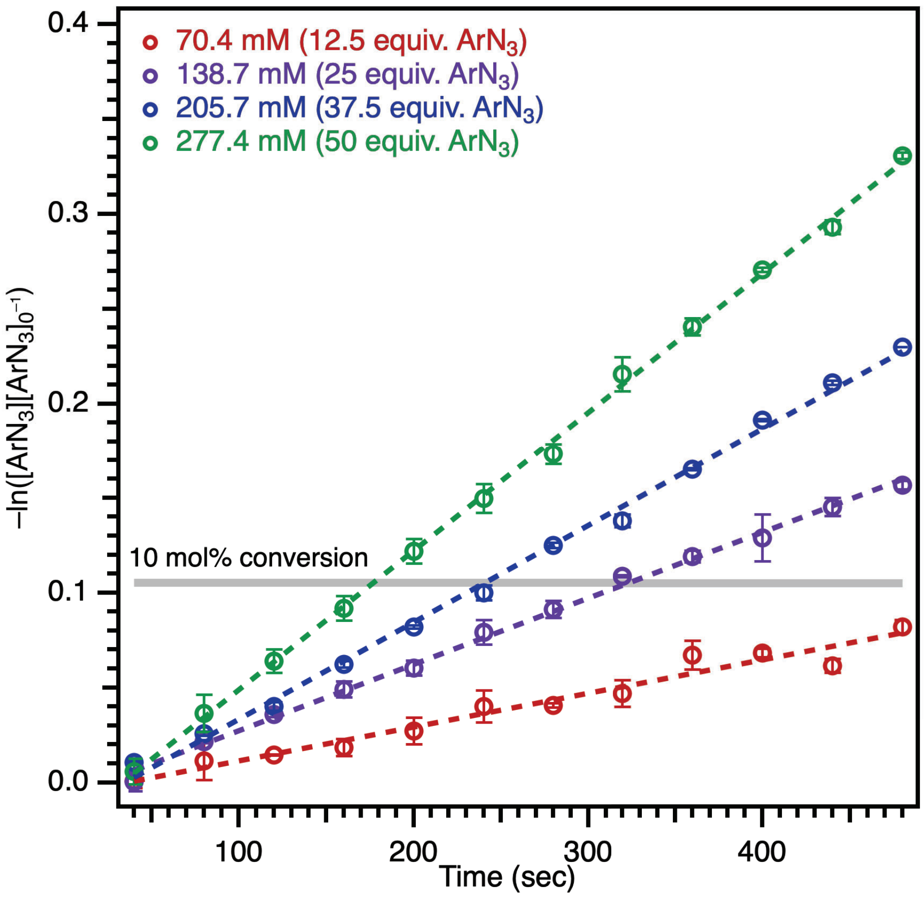

Figure S70. Kinetic analysis of tetrahydrofuran amination with (^{ArF}L)Cu (**4**) at variable ArN_3 loadings: 12.5 equiv. (*red*), 25.0 equiv. (*purple*), 37.5 equiv. (*blue*), 50.0 equiv. (*green*).

Table S8. Rate Constants for tetrahydrofuran amination with variable aryl azide loadings.

Aryl Azide Concentration (mM)	Rate Constant (sec^{-1})
70.4	$1.8(1) \times 10^{-4}$
138.7	$3.72(9) \times 10^{-4}$
205.7	$5.2(2) \times 10^{-4}$
277.4	$7.19(7) \times 10^{-4}$

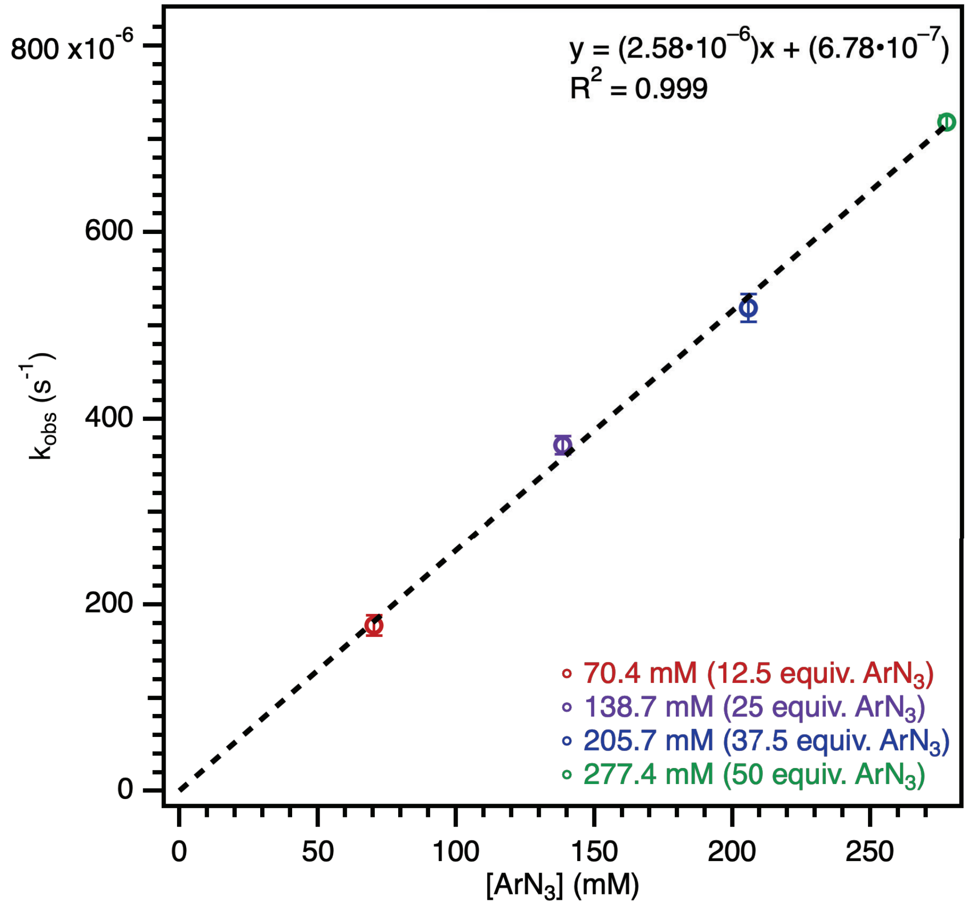

Figure S71. Kinetic analysis of tetrahydrofuran amination with (^{ArF}L)Cu (**4**) at variable ArN_3 loadings: 12.5 equiv. (*red*), 25.0 equiv. (*purple*), 37.5 equiv. (*blue*), 50.0 equiv. (*green*). A first-order dependence on concentration of aryl azide is observed. This observation is in accord with the apparent first-order depletion of aryl azide under catalytic conditions in which the concentration of **4** is constant, and the concentration of tetrahydrofuran is essentially constant due to its vast excess.

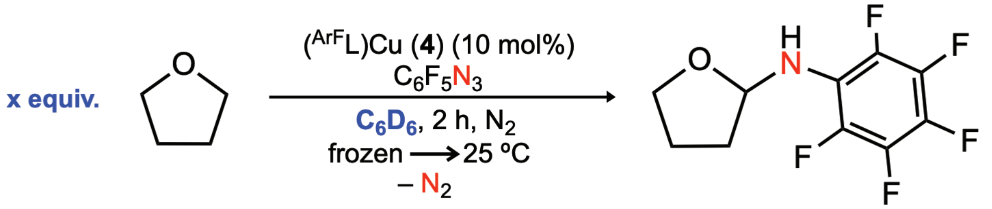

Substrate (tetrahydrofuran)–loading Analysis. *All manipulations were conducted using silanized glassware*. *All manipulations were conducted using silanized glassware*. In a dinitrogen-filled drybox, a precise quantity of pentafluorophenyl azide ($C_6F_5N_3$; 0.023 g, 0.195 mmol, 1.0 equiv.) with fluorobenzene (internal standard) was combined with 0.20 mL of a prepared solvent mixture comprised of tetrahydrofuran and d_6–benzene and transferred to a J. Young NMR tube. Additional solvent mixture (0.20 mL) was used to ensure complete transfer of the azide solution. The solution was frozen in a liquid-nitrogen chilled cold well. Separately, the specified amount of (^{ArF}L)Cu (**4**) (0.004 g, 0.004 mmol) was dissolved in the solvent mixture (0.2 mL) and frozen in the cold well. Once frozen, **4** was removed from the cold well and rapidly added to the J. Young tube (*NB*: the THF solution of **4** should freeze on the sides of the J. Young NMR tube and not dissolve the pre-frozen azide solution). Addition thawing solvent mixture (0.1 mL) was used to ensure complete transfer of **4** such that the total volume of the solvent mixture in the J. Young NMR tube was exactly 0.70 mL between all catalytic runs. The J. Young NMR tube was sealed, *rapidly* exported from the drybox, and quickly plunged into a liquid nitrogen bath to prevent thawing. The J. Young NMR tube was subsequently thawed and directly inserted into a pre-heated 500 MHz NMR spectrometer (25.0 ºC), followed by a ^{19}F NMR array collection (40 s intervals) for 2 h.

Note I: The resulting melting point of the solvent mixture was observed to vary as a function of relative THF/C_6D_6 concentration. Consequently, the initial time point recorded for the reaction kinetics was observed as highly variable between data runs, although the rate of consumption of aryl azide remained independent of THF/C_6D_6 concentration.

Note II: During routine measurements of tetrahydrofuran loadings, we observed diminished yields below *ca*. 100 equiv. of THF, accompanied by increased decomposition of the catalyst.

In the presence of a vast excess of THF, minimal changes are observed in the resulting rate of consumption of aryl azide, illustrating an overall zeroth order with respect to substrate concentration when in excess. We note that the order in THF may exhibit saturation kinetics, obfuscated by the background decomposition reaction at low quantities of substrate.

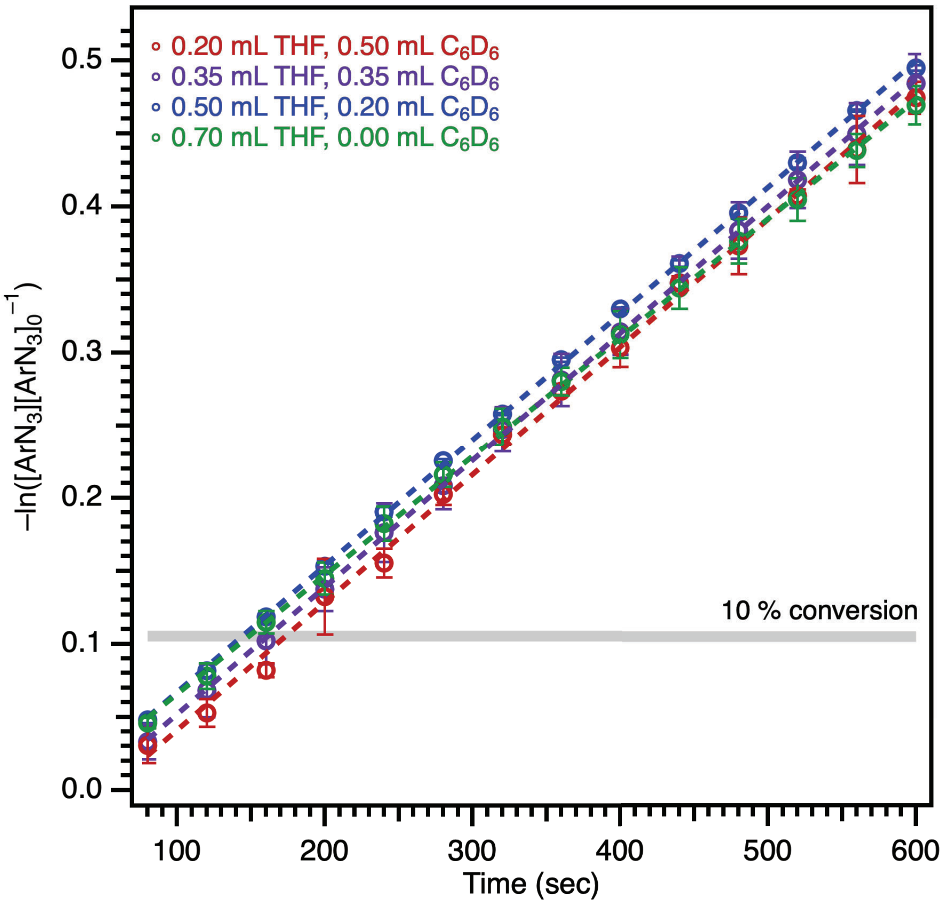

Figure S72. Kinetic analysis of tetrahydrofuran amination with (^{ArF}L)Cu (**4**) at variable tetrahydrofuran loadings: 0.20 mL THF (*red*), 0.35 mL THF (*purple*), 0.50 mL THF (*blue*), 0.70 mL THF (*green*). A zeroth-order dependence on concentration of tetrahydrofuran, when in vast excess, is observed.

Table S9. Rate Constants for tetrahydrofuran amination with variable substrate loading.

THF Loading	Rate Constant (sec^{-1})
0.20 mL THF, 0.50 mL C_6D_6	8.14(11) × 10^{-4}
0.35 mL THF, 0.35 mL C_6D_6	8.75(4) × 10^{-4}
0.50 mL THF, 0.20 mL C_6D_6	8.70(4) × 10^{-4}
0.70 mL THF, 0.00 mL C_6D_6	8.64(6) × 10^{-4}

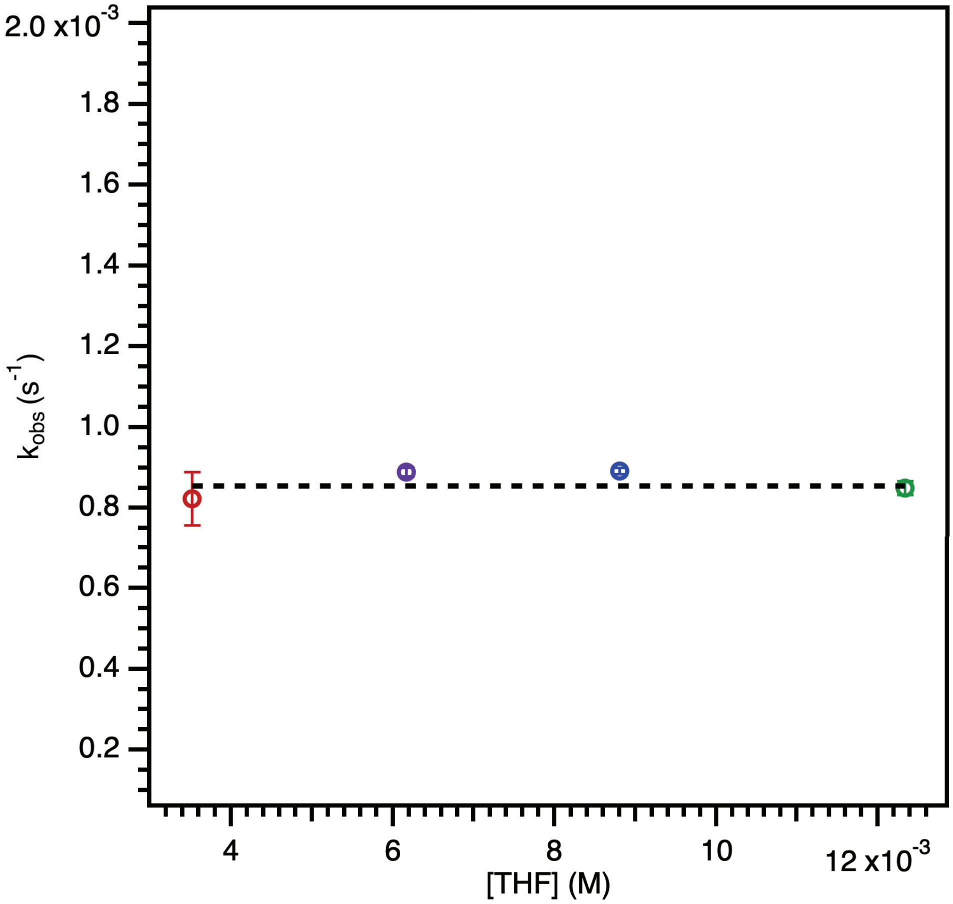

Figure S73. Kinetic analysis of tetrahydrofuran amination with (^{ArF}L)Cu (**4**) at variable tetrahydrofuran loadings: 0.20 mL THF (*red*), 0.35 mL THF (*purple*), 0.50 mL THF (*blue*), 0.70 mL THF (*green*). A constant k_{obs} value provides a satisfactory fit for the data points.

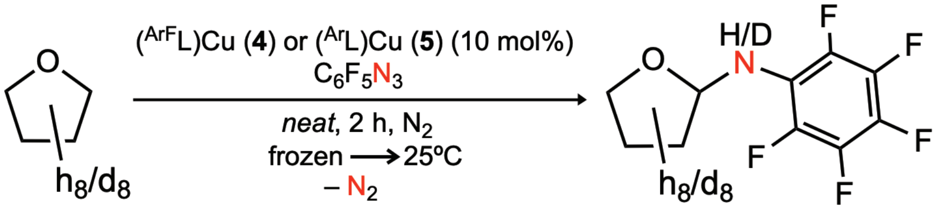

Absolute Catalytic *h*$_8$–tetrahydrofuran *d*$_8$–tetrahydrofuran Kinetic Isotope Effect (KIE) Measurement. *All manipulations were conducted using silanized glassware*. In a dinitrogen-filled drybox, pentafluorophenyl azide ($C_6F_5N_3$; 0.023 g, 0.195 mmol, 1.0 equiv.) with fluorobenzene (internal standard) was combined with *h*$_8$–tetrahydrofuran or *d*$_8$–tetrahydrofuran (0.20 mL) using a volumetric syringe and added to a J. Young NMR tube. Additional *h*$_8$ or *d*$_8$ tetrahydrofuran (0.20 mL) was used to ensure complete transfer of the azide solution. The solution was frozen in a liquid-nitrogen chilled cold well. Separately, either (^{ArF}L)Cu (**4**) or (^{ArF}L)Cu (**5**) (0.010 mmol, 0.1 equiv.) was dissolved in *h*$_8$ or *d*$_8$ tetrahydrofuran (0.2 mL) and frozen in the cold well. Once frozen, the solution containing the Cu catalyst was removed from the cold well and rapidly added to the J. Young tube. Addition thawing *h*$_8$ or *d*$_8$ tetrahydrofuran (0.1 mL) was used to ensure complete transfer of **4** such that the total solvent volume in the J. Young NMR tube was exactly 0.70 mL. The J. Young NMR tube was sealed, *rapidly* exported from the drybox, and quickly plunged into a liquid nitrogen bath to prevent thawing. The J. Young NMR tube was subsequently thawed and directly inserted into a pre-heated 500 MHz NMR spectrometer, followed by a ^{19}F NMR array collection (20 s intervals) for 2 h.

For data processing, the integration values of unreacted aryl azide and the hemiaminal were normalized to the internal standard. Rate constants were measured using initial rates approach, monitoring consumption of $C_6F_5N_3$ to 10 %.

The measured absolute KIE for **4** is 1.10(2).
The measured absolute KIE for **5** is 2.06(1).

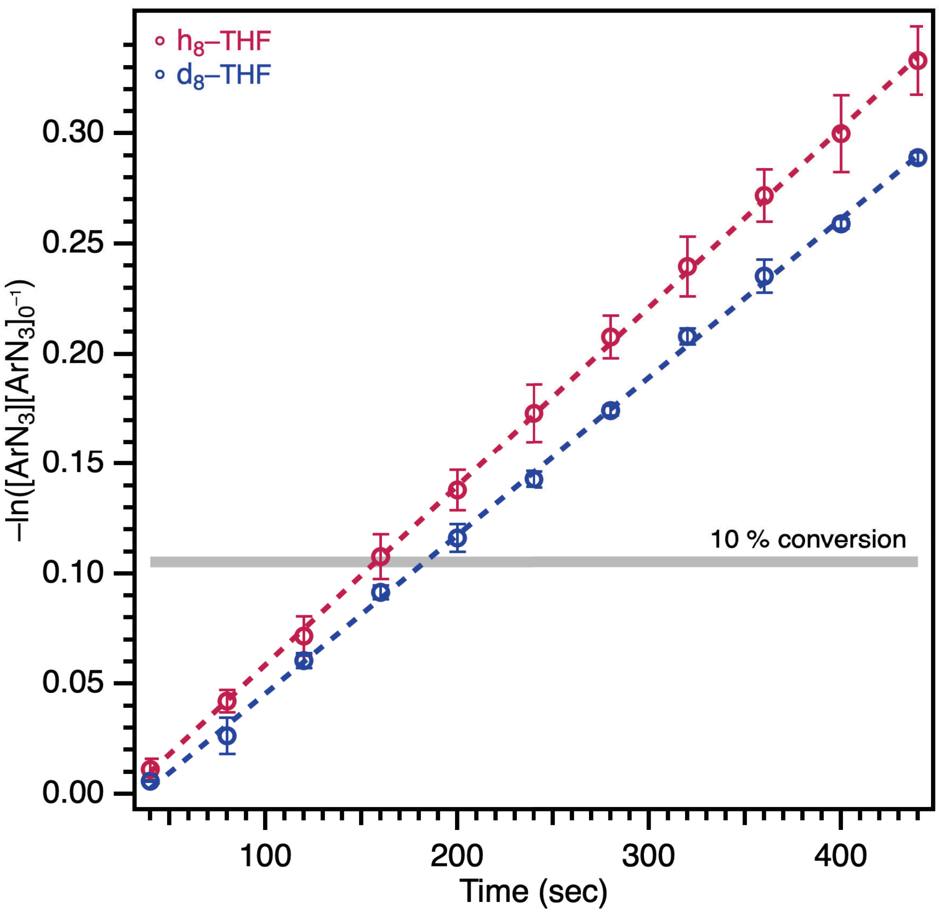

Figure S74. Absolute kinetic isotope effect (KIE) measurement of tetrahydrofuran amination with (^{ArF}L)Cu (**4**).

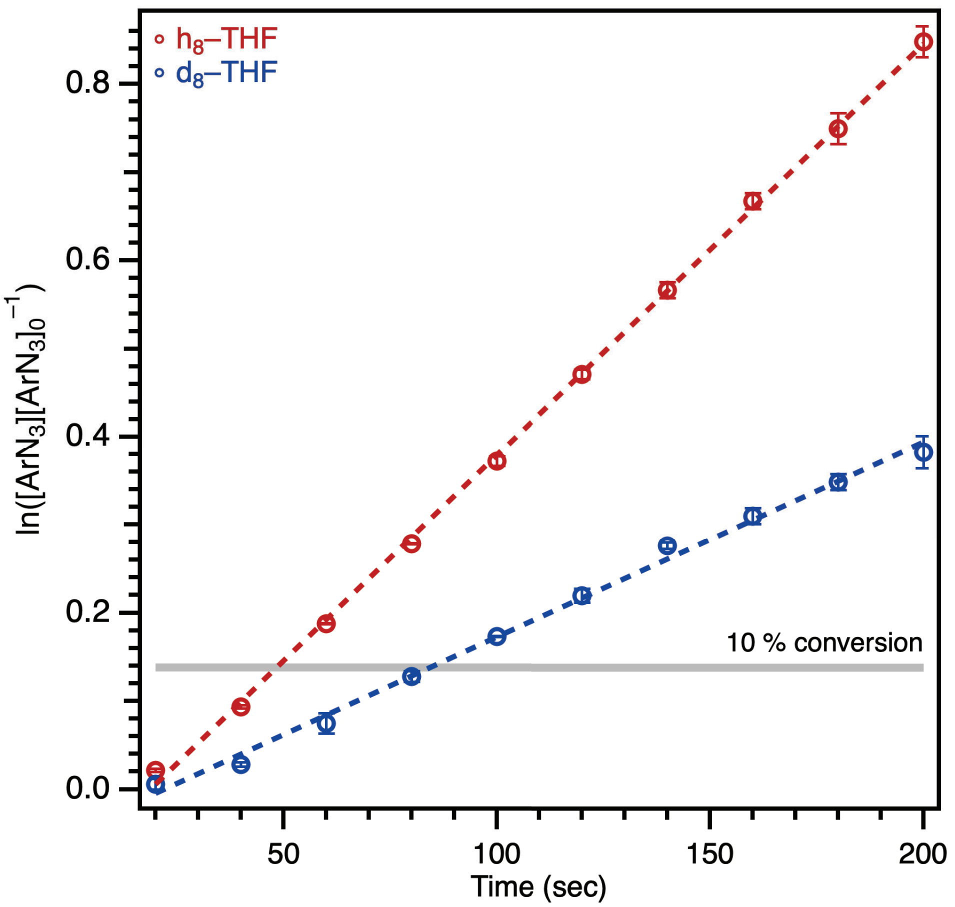

Figure S75. Absolute kinetic isotope effect (KIE) measurement of tetrahydrofuran amination with (^{Ar}L)Cu (**5**).

Additional Physical Characterization and Spectroscopic Characterization.

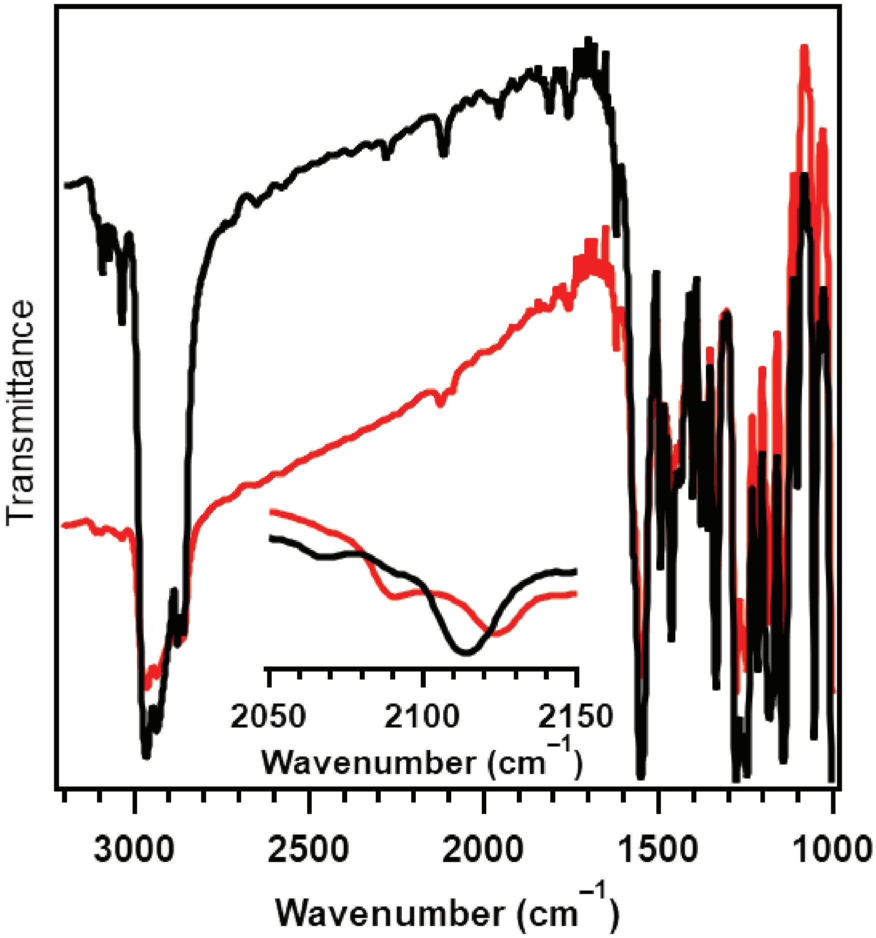

Figure S76. Infrared spectra overlay of (^{EMind}L)Cu($N_3C_6H_4{}^tBu$) (bottom, *red*) and (^{EMind}L)Cu($NN^{15}NC_6H_4{}^tBu$) (top, *black*); (*Inset*) Truncated IR spectra highlighting differences in the azide frequency as a function of the N_α isotope.

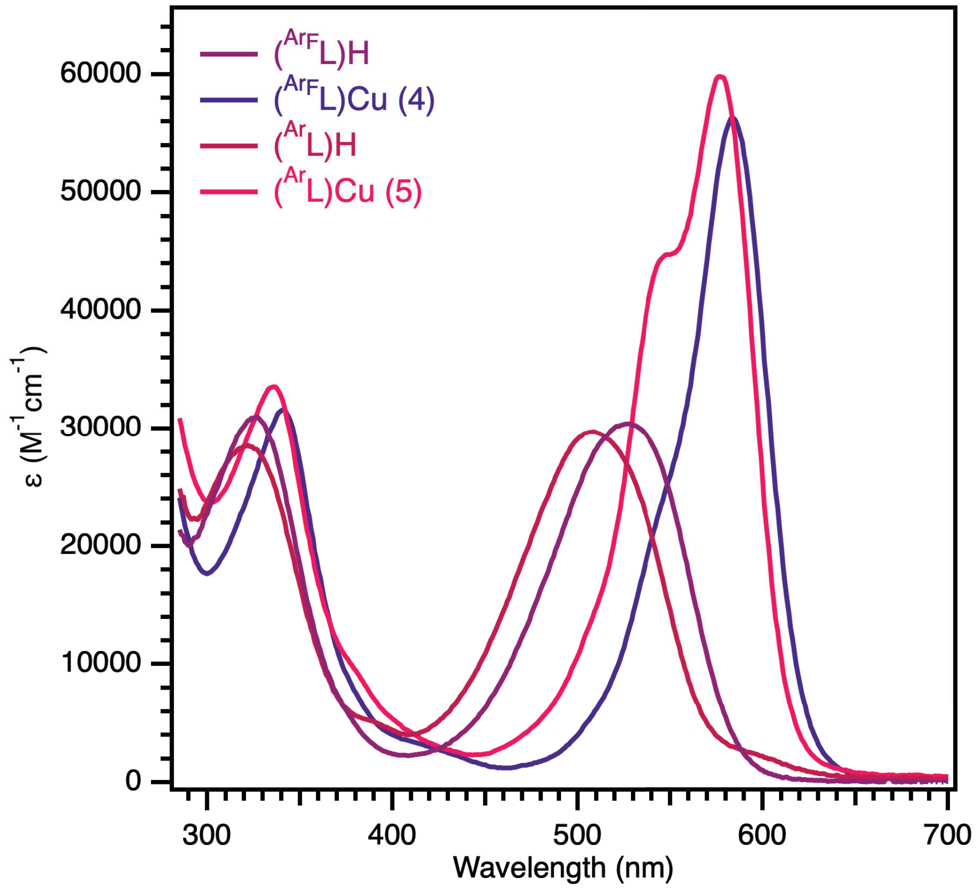

Figure S77. UV/Vis spectroscopy comparison of (^{ArF}L)H, (^{ArF}L)Cu (**4**), (^{Ar}L)H, and (^{Ar}L)Cu (**5**), displaying a red-shift in the Soret band upon fluorination of the *meso* arene.

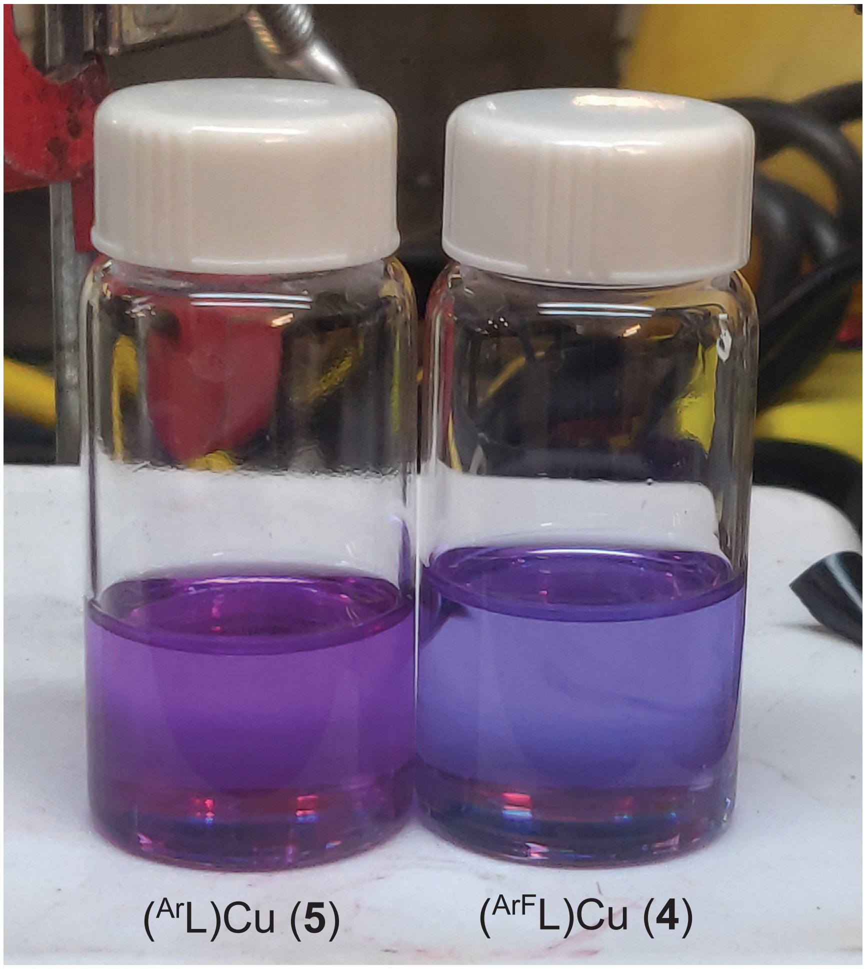

Figure S78. Visual comparison of (^{ArF}L)Cu (**4**) (*right*) and (^{Ar}L)Cu (**5**), displaying a visible shift in apparent color as a function of *meso* arene fluorination. Both solutions are 20 μM concentration in benzene.

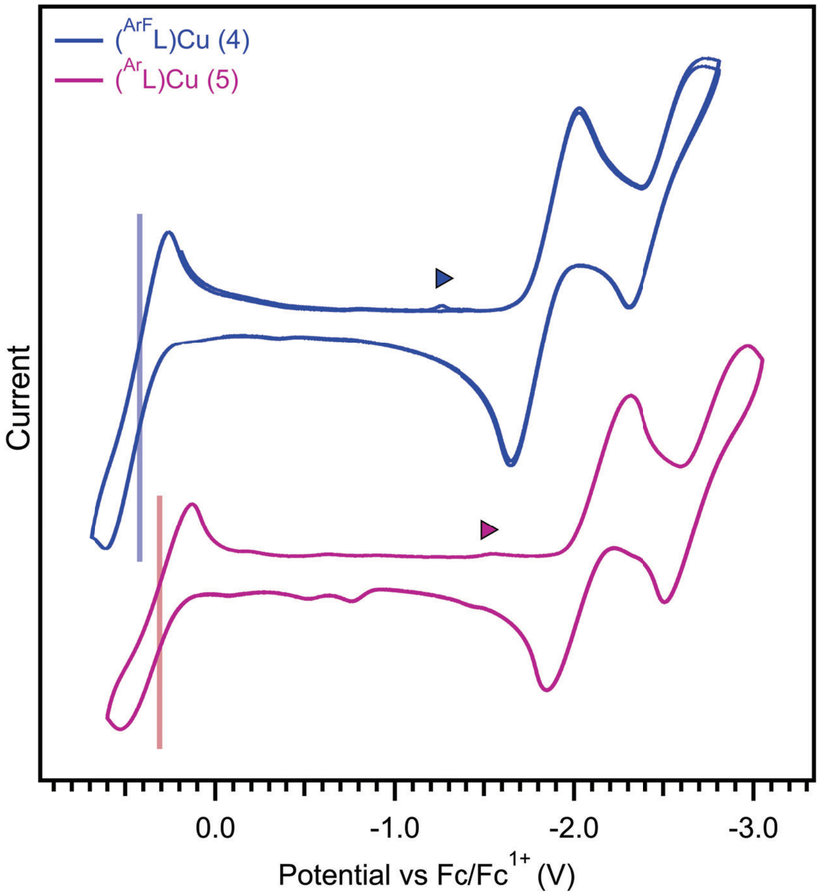

Figure S79. Quasi-reversible one-electron reduction of (^{ArF}L)Cu (**4**) ($E_{1/2}$ = 0.42 V, –1.86 V, –2.54 V vs. Fc/Fc^{1+}) and (^{ArF}L)Cu (**5**) ($E_{1/2}$ = 0.31 V, –2.10, –2.75 vs. Fc/Fc^{1+}) at a scan rate of 50 mV s^{-1}. The data was recorded in 1,2-difluorobenzene at a concentration of ca. 0.01 mM, with glassy carbon, Pt-wire, and Ag-wire as the working, counter, and reference electrodes, respectively. Arrows denote initial scan direction (reductive). Saturated tetrabutylammonium hexafluorophosphate ([^{n}Bu_4N][PF_6]) solutions of 0.2 M in tetrahydrofuran were prepared before each experiment in the presence of molecular sieves to remove trace water. No background reaction between **4** or **5** and the electrolyte was observed.

Translucent vertical bars denote Cu^{II}/Cu^{I} redox couple for **4** ($E_{1/2}$ = 0.42 V) and **5** ($E_{1/2}$ = 0.31 V).

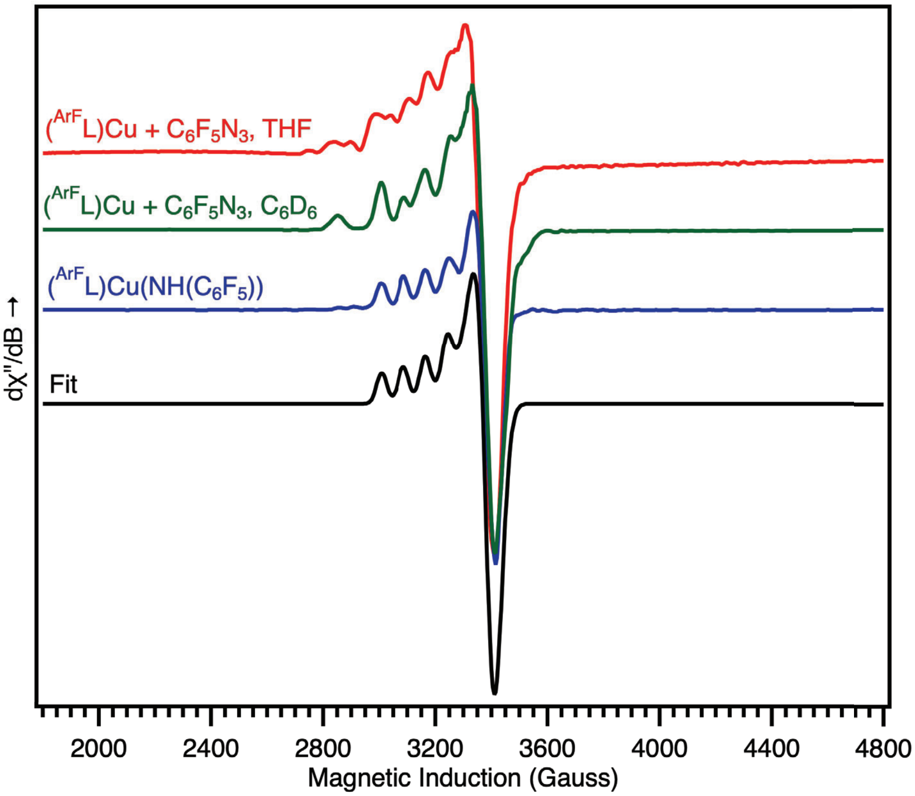

Figure S80. Frozen 2–methyltetrahydrofuran EPR spectra (77 K) of (^{ArF}L)Cu(NH(C_6F_5)), prepared by treatment of (^{ArF}L)Cu (**4**) with $C_6F_5N_3$ in the presence of 1,4-cyclohexadiene (*blue spectrum*) with corresponding fit (*black spectrum*), crude EPR spectrum upon treatment of **4** with $C_6F_5N_3$ in C_6D_6 (*green spectrum*), and crude EPR of tetrahydrofuran amination by **4** at 1 mol% Cu loading. The presence of the cupric anilide species is detected in the crude background amination reaction and the catalytic substrate amination, although additional resonances can be ascribed to unidentified Cu(II) byproducts, which are observed by ^{19}F NMR spectroscopy for ligand-related resonances (the corresponding resonance of the C_6F_5 motif are not observed for Cu(II) species).

Data collection parameters for each spectrum are as follows:
Cupric anilide (*blue spectrum*): frequency 9.647 GHz, microwave power 0.20 mW
Background azide consumption (*green spectrum*): frequency 9.649 GHz, microwave power 0.63 mW
Catalytic THF amination (*red spectrum*): frequency 9.648 GHz, microwave power 0.63 mW

Fitting parameters for the cupric anilide species are determined from Easyspin[6] and as follows for a doublet (S = ½) fit: $g_\perp$ = 2.035, $g_{||}$ = 2.205, $\sigma g_\perp$ = 0.045, $\sigma g_{||}$ = 0.029, $A_{||}(^{63}Cu)$ = 237 MHz.

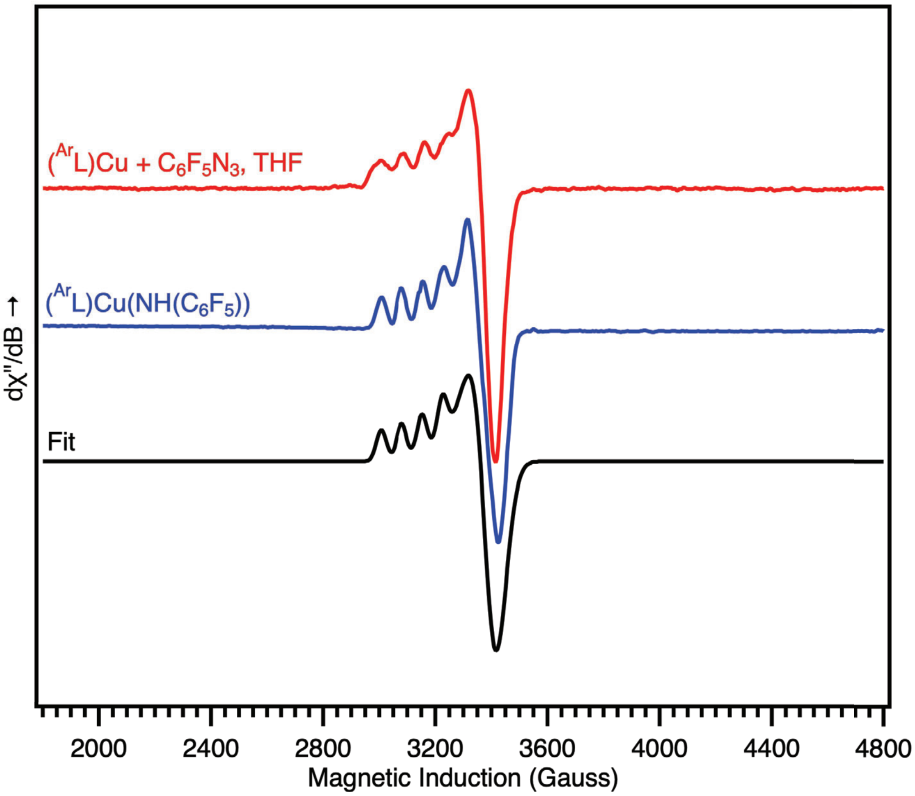

Figure S81. Frozen 2–methyltetrahydrofuran EPR spectra (77 K) of crude reaction mixture of treatment of (^{Ar}L)Cu (1 mol%, *red spectrum*) compared to authentic (^{Ar}L)Cu(NH(C_6F_5) (*red spectrum*)[3] with corresponding fit (*black spectrum*).

Data collection parameters for tetrahydrofuran amination are as follows: frequency 9.656 GHz, microwave power 0.20 mW.

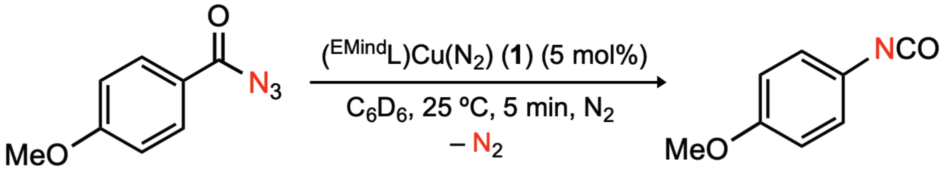

Catalytic Curtius Rearrangement of Aroyl Azides. In a dinitrogen-filled drybox, 4-methoxybenzoyl azide[28] (0.017 g, 0.094 mmol, 1.0 equiv.) in C_6D_6 (0.5 mL) was rapidly added to a thawing solution of (^{EMind}L)Cu(N_2) (**1**) (0.005 g, 0.005 mmol, 0.05 equiv.) in C_6D_6 (0.5 mL), resulting in rapid effervescence. After *ca*. 5 minutes, effervescence ceased, and the reaction was checked by multinuclear NMR, revealing full consumption of the aroyl azide and formation of the corresponding aryl isocyanate, 1-isocyanato-4-methoxybenzene[29] (96(2) % yield).

By contrast, trace aryl isocyanate (< 10 %) was observed from the analogous reactions using 5 mol% (^{ArF}L)Cu (**4**) or (^{ArF}L)Cu (**5**), attributed to formation of Cu^{II} species due to facile decomposition from the absence of steric preclusion from possible hydrogen-atom abstraction.

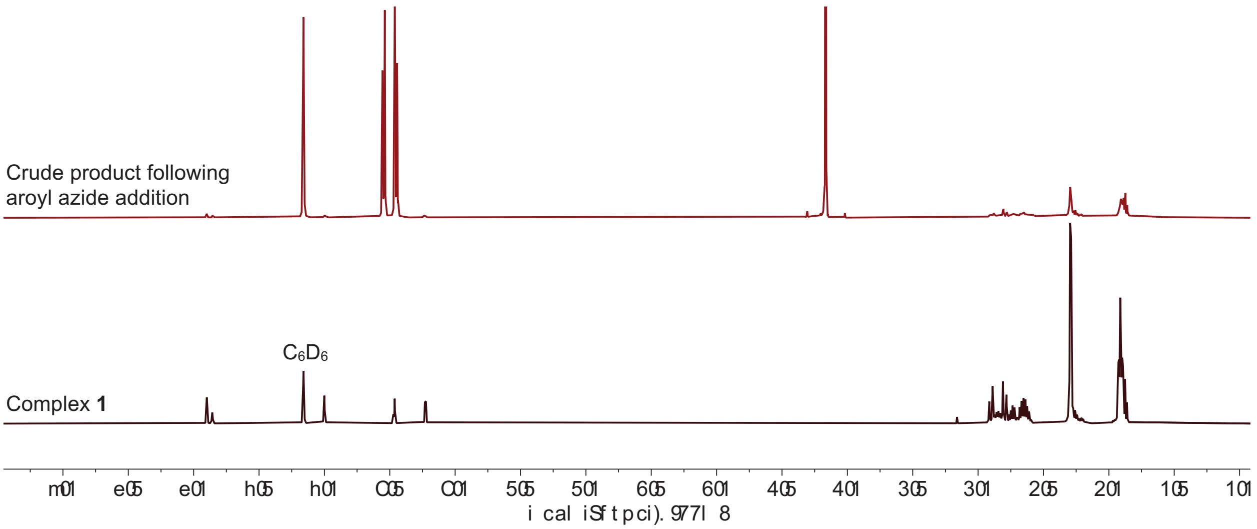

Figure S82. Stacked crude ^{1}H NMR spectra (500 MHz, C_6D_6) of reference (^{EMind}L)Cu(N_2) (**1**) (*bottom*) following by addition of 4-methoxybenzoyl azide, resulting in formation of the aryl isocyanate and recovery of **1** as evident by baseline ^{1}H resonances (*top*).

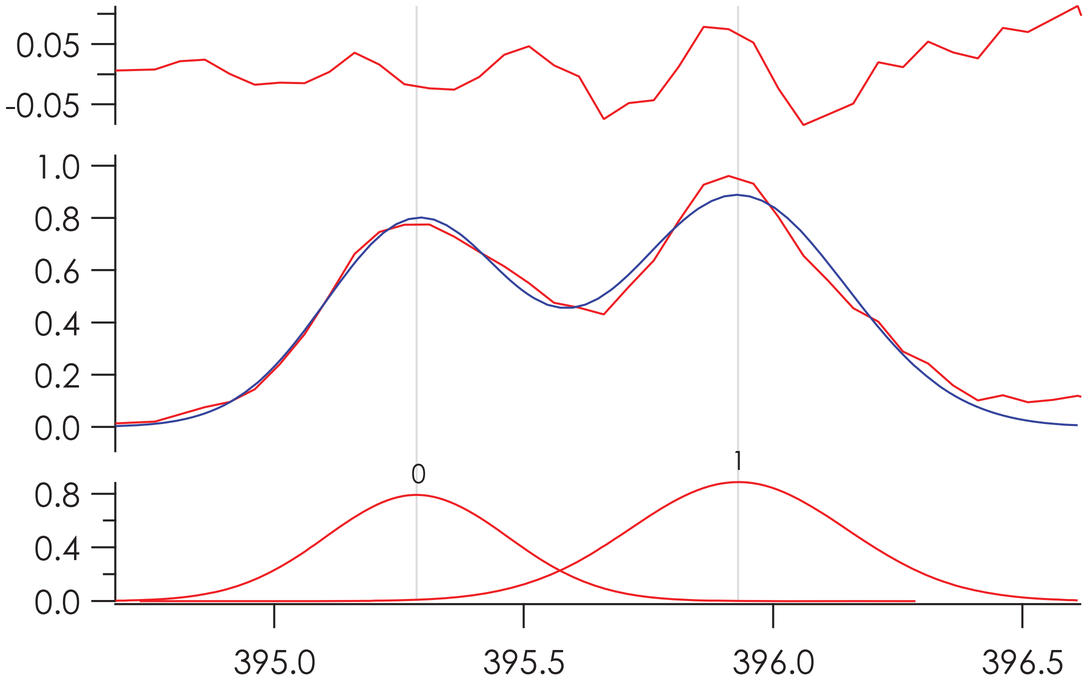

Figure S83. Gaussian fit of the nitrene pre-edge features in the (^{EMind}L)Cu[N($C_6H_4{}^tBu$)] (**3-^{t}Bu**) N K-edge spectrum as a function of incidental energy (eV). Total peak area: 0.84 ± 0.02.

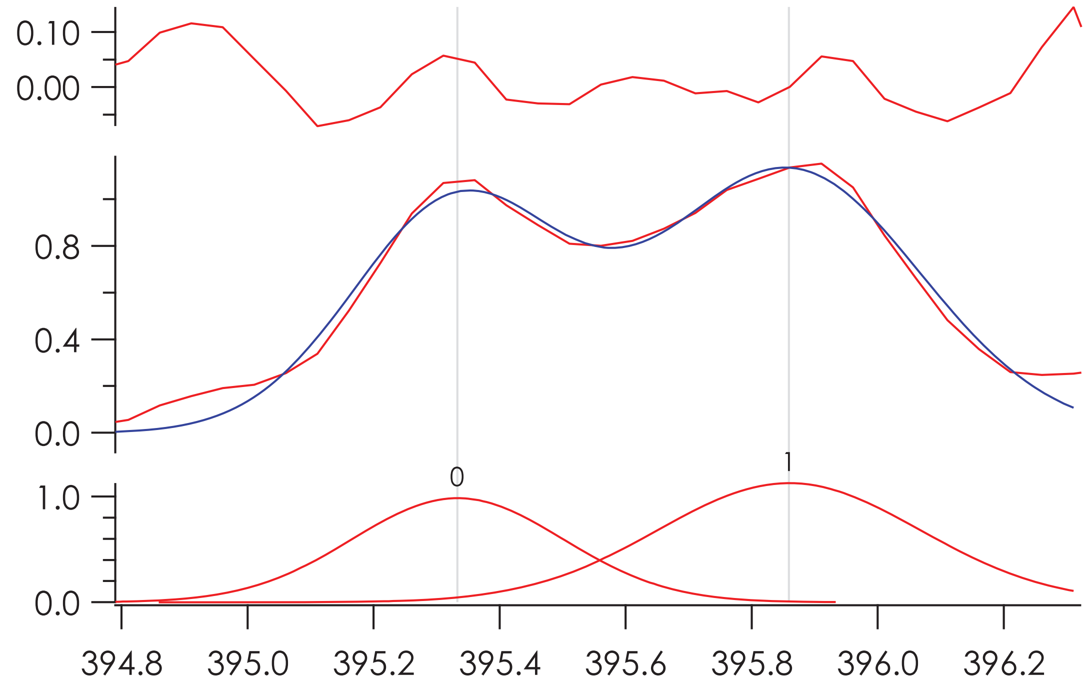

Figure S84. Gaussian fit of the nitrene pre-edge features in the (^{EMind}L)Cu[N($C_6H_4O^tBu$)] N K-edge spectrum as a function of incidental energy (eV). Total peak area: 1.00 ± 0.04.

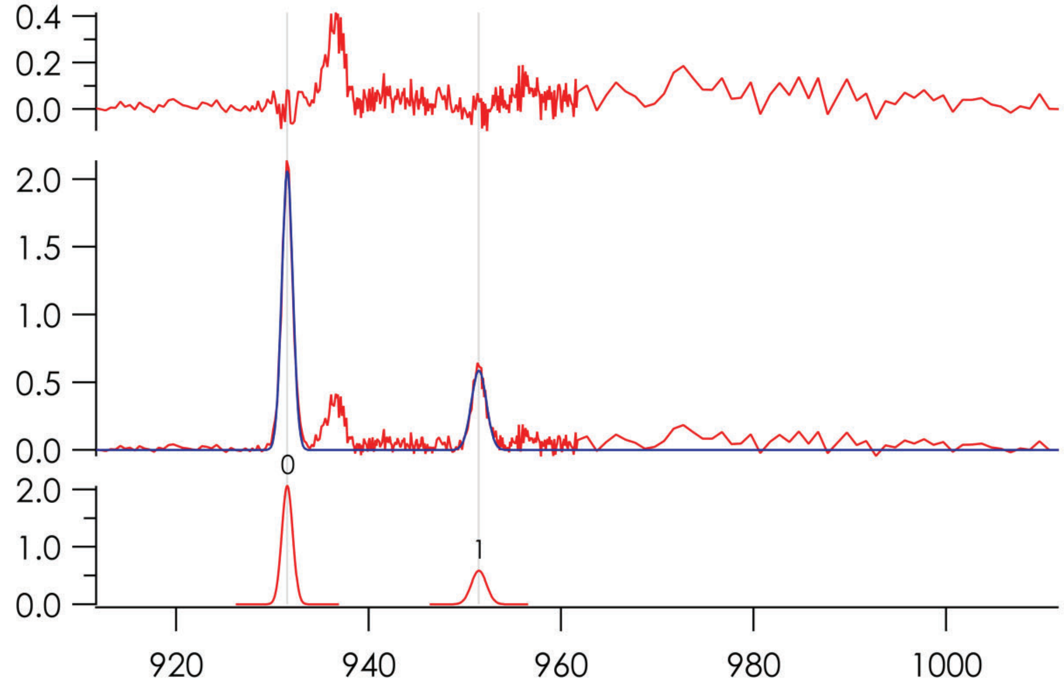

Figure S85. Gaussian fit of the Cu $L_{2,3}$-edge absorption spectra of (^{EMind}L)Cu[N($C_6H_4{}^t$Bu)] (**3-^{t}Bu**) as a function of incidental energy (eV). Total $L_2 + L_3$ main line area: 4.09 ± 0.11.

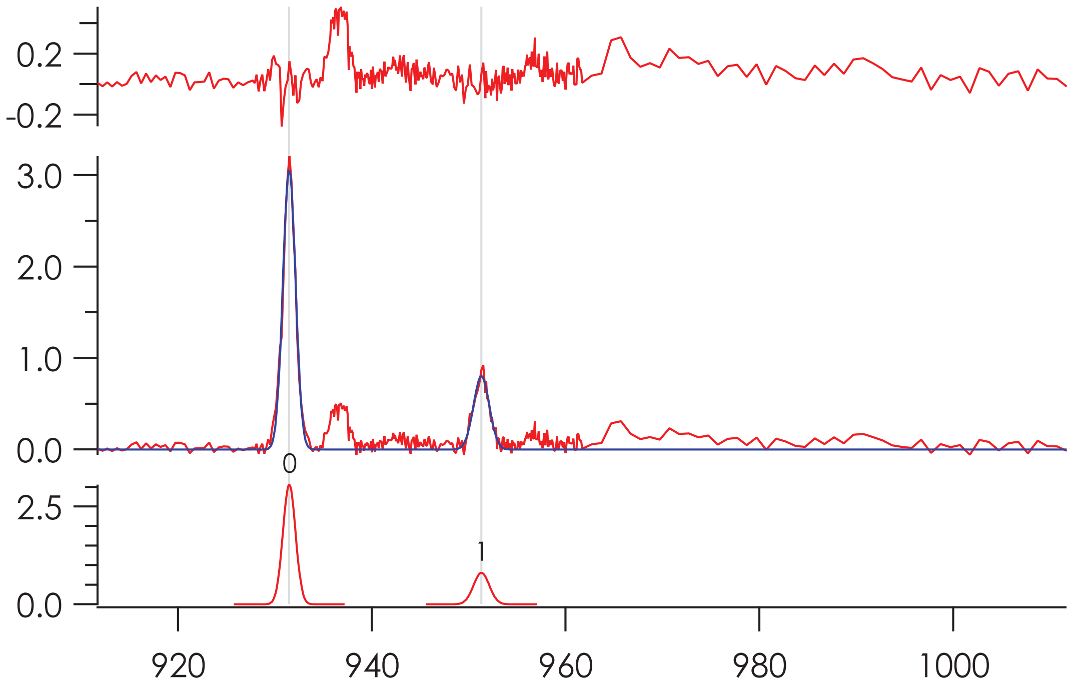

Figure S86. Gaussian fit of the Cu $L_{2,3}$-edge absorption spectra of (^{EMind}L)Cu[N($C_6H_4O{}^t$Bu)] (**3-O^{t}Bu**) as a function of incidental energy (eV). Total $L_2 + L_3$ main line area: 6.60 ± 0.16.

Table S10. X-ray diffraction experimental details.

	$(^{EMind})LCu(N_3Ad)$ (**2**)	$(^{Ar_F}L)Cu$ (**4**)
**CCDC Entry**	2080873	2080874
**Moiety Formula**	$C_{75}H_{96}CuF_6N_5 \bullet 2(C_4H_{10}O)$	$C_{65}H_{41}CuF_6N_2$
**Formula Weight**	1393.36	1027.54
$\boldsymbol{\lambda}$ (nm)	0.71073	0.71073
$\boldsymbol{T}$ (K)	100(2)	100(2)
**Crystal System**	Monoclinic	Monoclinic
**Space Group** (*Z*)	$P2_1/c$ (4)	$P2_1/c$ (4)
$\boldsymbol{a}$ (Å)	10.531(3)	14.053(4)
$\boldsymbol{b}$ (Å)	28.389(9)	14.168(3)
$\boldsymbol{c}$ (Å)	26.123(13)	25.657(5)
$\boldsymbol{\alpha}$ (°)	90	90
$\boldsymbol{\beta}$ (°)	98.954(17)	94.936(8)
$\boldsymbol{\gamma}$ (°)	90	90
**Volume** (Å^3)	7715(5)	5090(2)
**Calc.** $\boldsymbol{\rho}$ **(mg m^{-3})**	1.200	1.341
$\boldsymbol{\mu}$ (mm^{-1})	0.346	0.495
**Crystal Size** (mm)	0.39x0.59x0.84	0.10x0.12x0.40
**Reflections**	9453	5761
**Completeness** (to 2θ)	0.982	0.977
**GOF on F^2**	1.085	1.031
$\mathbf{R_1}$, $\boldsymbol{w}\mathbf{R_2}^a$ $[\boldsymbol{I} > 2\boldsymbol{\sigma}(\boldsymbol{I})]$	0.1095, 0.2643	0.0454, 0.1109

[a] $R1 = \sum||F_o| - |F_c|| / \sum|F_o|$, $wR2 = \{\sum[w(F_o^2 - F_c^2)^2] / \sum[w(F_o^2)^2]\}^{1/2}$

X-Ray Diffraction Techniques. All structures were collected on a Bruker three-circle platform goniometer equipped with an Apex II CCD and an Oxford cryostream-cooling device fixed at 100 K. Radiation was from a graphite fine focus sealed tube Mo Kα (λ = 0.71073 Å) source (**2**, **4**). The partial solubility of **2** in Paratone–*N* oil forced us to employ an alternative crystal mounting procedure. Crystals were suspended in minimal thawing Fluorolube (stored in a vacuum desiccator or the drybox to minimize water content), quickly transferred to a dry ice bath outside the drybox (< 10 seconds transit time) and rapidly transferred to a room temperature microscope slide (*ca*. 3 minute transit time) for crystal selection with a glass fiber pin. At room temperature, crystals were observed to darken rapidly and lose the ability to polarize incidental light, attributed to desolvation of the unit cell and gradual dissolution into the surrounding oil. Such behavior was common for crystals isolated from slow cooling of concentrated hydrocarbon solutions, pentane in particular. Consequently, the crystal selection process was rapidly conducted at low temperatures, considering crystal size and crystal quality. Crystals of **4** were mounted using Paratone–*N* oil. None of the crystals shows significant decay during the data collection.

Data was collected as a series of φ and/or ω scans. Data integration down to 0.84 Å resolution was carried out using SAINT V8.37 A[30] with reflection spot size optimization. Absorption corrections were made with the program SADABS.[31] Space group assignments were determined by examination of systematic absences, E-statistics, and successive refinement of the structures. The structure was solved by the Intrinsic Phasing methods and refined by least-squares methods against F^2 using SHELXT-2014[32] and SHELXL-2014[33] with the OLEX2 interface.[34] The program PLATON was employed to confirm the absence of higher symmetry space groups.[35] All non-H atoms, including the disorder fragments, were located in difference Fourier maps, and then refined anisotropically. The restraints on bond lengths and constraints of the atomic displacement parameters on each pair of disorder fragments (SADI/SAME and EADP instructions of SHELXL-2014)[33] as well as the restraints of the atomic displacement parameters (SIMU/RIGU instructions of SHELXL-2014), if necessary, have been applied for the disorder refinement.[36] All non-hydrogen atoms were refined anisotropically. Outlier reflections were omitted from refinement when appropriate. Hydrogen atoms on C atoms were placed at idealized positions and refined using a riding model. The isotropic displacement parameters of all hydrogen atoms were fixed to 1.2 times the atoms they are linked to (1.5 times for methyl groups). Crystallographic refinement details, including disorder modeling and software employed, have been delineated within in each crystallographic information file (*.*cif*).

Molecular graphics were generated using Povray V3.7.[37]

Further details on particular data collections are noted below:

(^{EMind}L)Cu(N_3Ad) (**2**). The structure was solved in the monoclinic space group $P2_1/c$ with one molecule of copper-containing complex per asymmetric unit and four molecules per unit cell. The adamantyl azide motif was positionally disordered and refined using similarity restraints and constraints. The fluorinated *meso* arene was positionally disordered and refined using similarity restraints and constraints. Two solvent molecules (diethyl ether) were located and refined using similarity restraints and constraints. Several reflections were coincidental with the beam stop and removed from the final data refinement. CCDC Identifier: 2080873.

(^{ArF}L)Cu (**4**). The structure was solved in the monoclinic space group P21/c with one molecule of copper-containing complex per asymmetric unit and four molecules per unit cell. Rotational and positional disorder for one trifluoromethyl substituent containing C16 was modeled using similarity constraints and restraints. The overlapping solvent molecules (assigned as benzene and hexane based on solvent crystallization conditions and apparent arrangement of electron density peaks) were disordered adjacent to an inversion center (special position), which resulted in an elongated U_{ij} tensor for the benzene motif. Consequently, the Olex2 solvent mask function was applied to remove contributions from the solvent molecules. CCDC Identifier: 2080874.

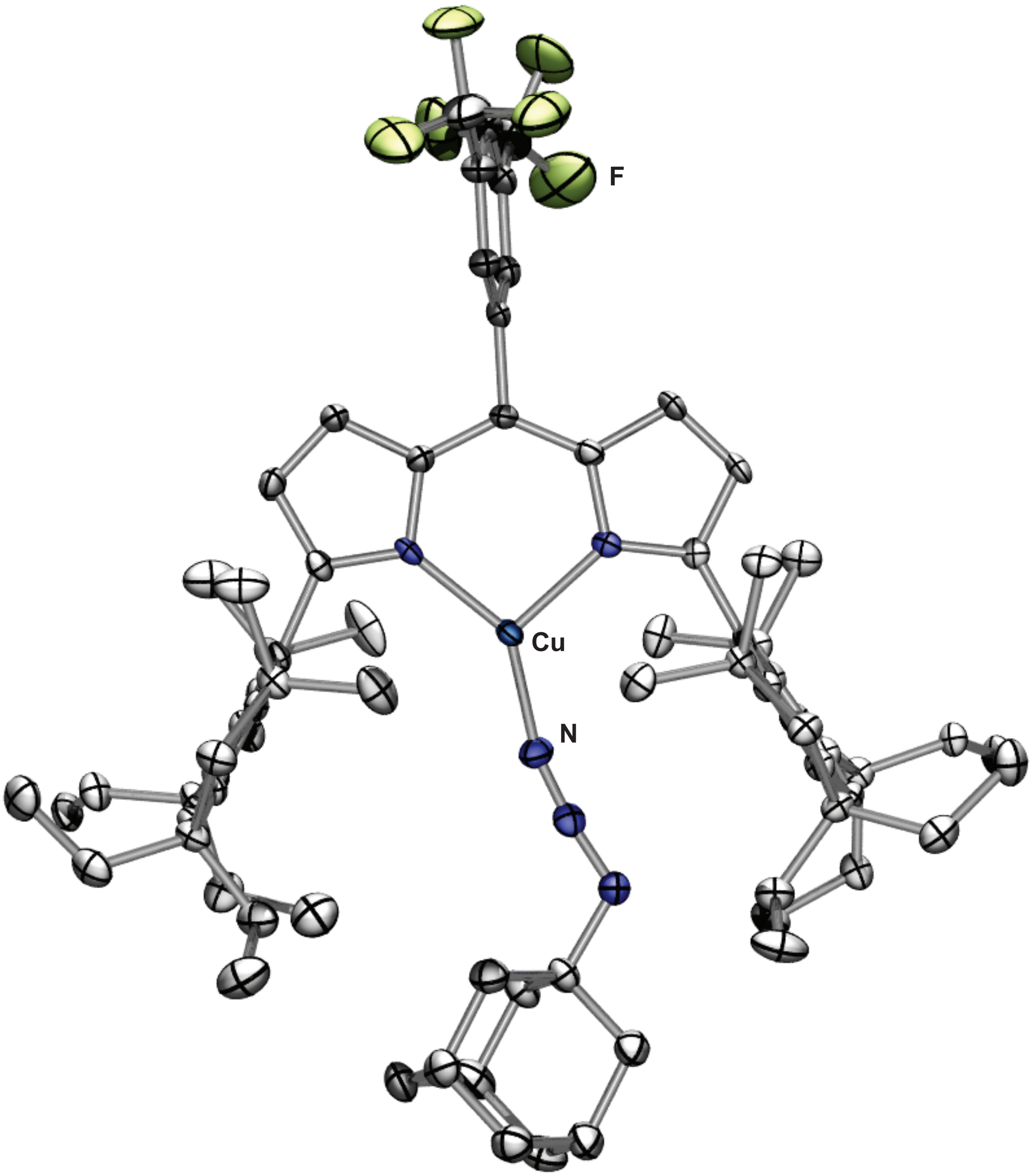

Figure S87. Solid-state molecular structure of (^{EMind}L)Cu(N_3Ad) (**2**) with displacement ellipsoids presented at 50 % probability level. Hydrogen atoms, structural disorder, and solvent molecules are omitted for clarity. Color scheme: Cu (*cobalt blue*), F (*yellow-green*), N (*blue*).

Table S11. Pertinent bond metrics for (^{EMind}L)Cu(N_3Ad) (**2**).

Cu1–N1	1.938(5) Å	N3–N4	1.129(8) Å
Cu1–N2	1.973(5) Å	N3–N5	1.231(9) Å
Cu1–N3	1.854(7) Å	Σ (∠Cu1)	360.0(5) º

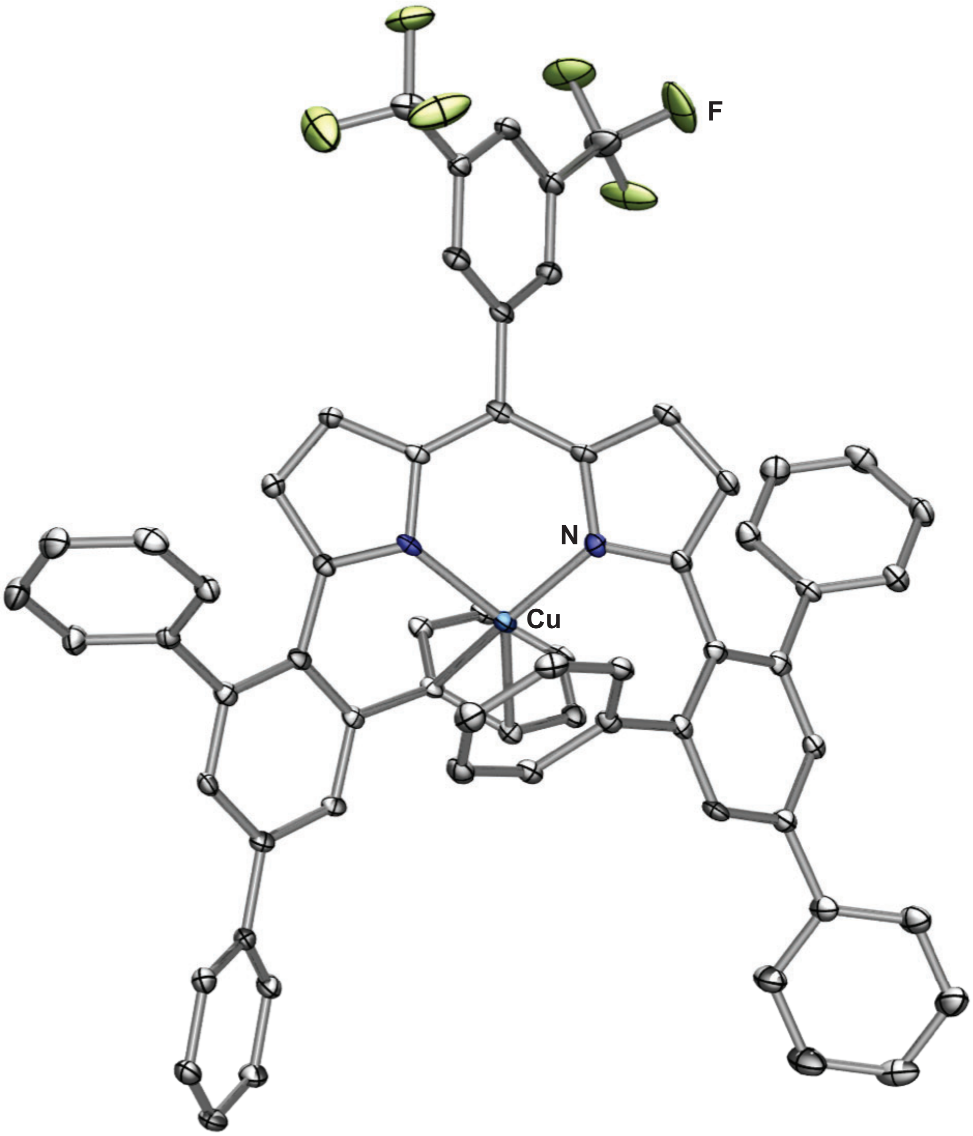

Figure S88. Solid-state molecular structure of (^{ArF}L)Cu (**4**) with displacement ellipsoids presented at 50 % probability level. Hydrogen atoms and structural disorder are omitted for clarity. Color scheme: Cu (*cobalt blue*), F (*yellow-green*), N (*blue*).

Table S12. Pertinent bond metrics for (^{ArF}L)Cu (**4**).

Cu1–N1	1.905(2) Å	Cu1–C36, Cu1–C41	2.007(2), 2.110(2) Å
Cu1–N2	1.946(2) Å	Cu1–(N1–C5–N2)**	0.528(3) Å
C36–C37*	1.390(2) Å	Σ (∠Cu1)	359.8(2) º

*C–C bond length denotes the η^2 arene interaction.
**Distance between dipyrrin plane (defined by both $N_{dipyrrin}$ and C_{meso}) and Cu1

Additional SORCI Calculations.

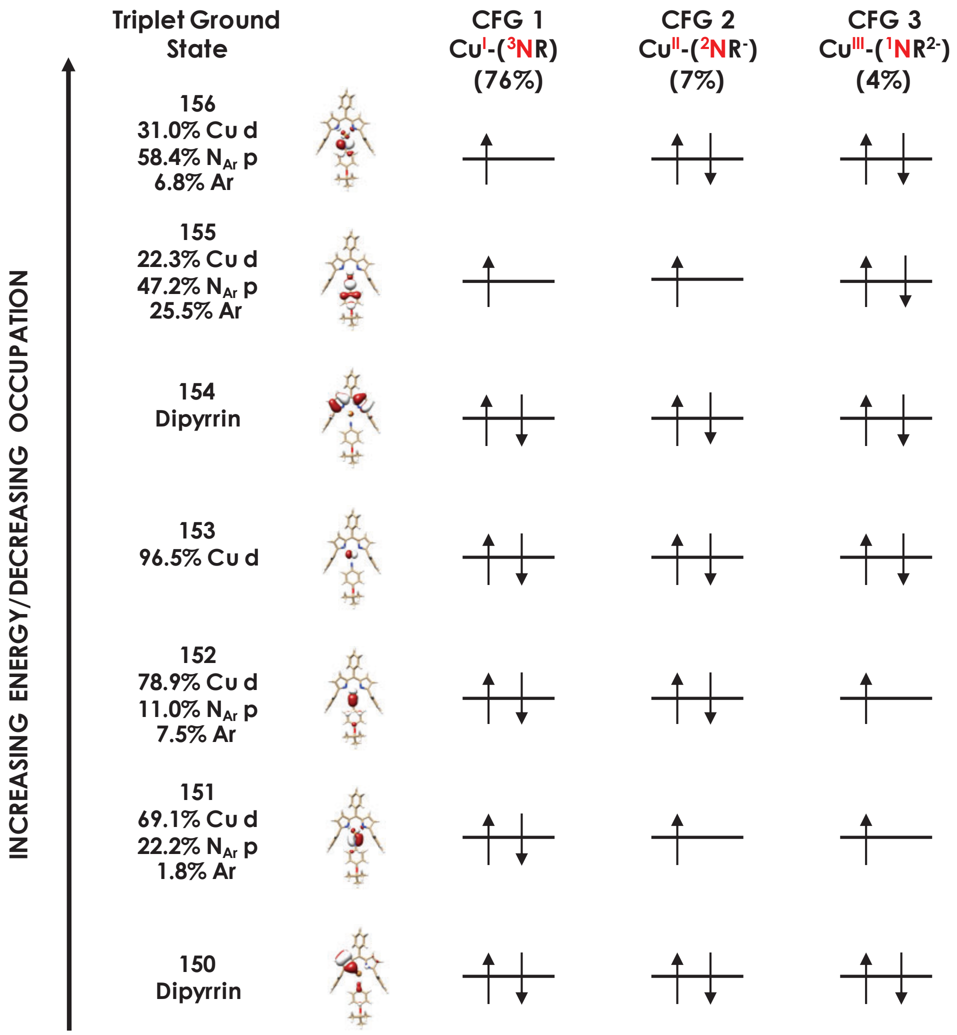

Figure S89. Leading configurations of the triplet ground state of a truncated model derived from the crystal structure of (^{EMind}L)Cu[N($C_6H_4O^tBu$)] generated through a SORCI procedure employing a CAS(12,7) reference (refWeight 0.8822). Configurations with contributions less than 1% have been omitted.

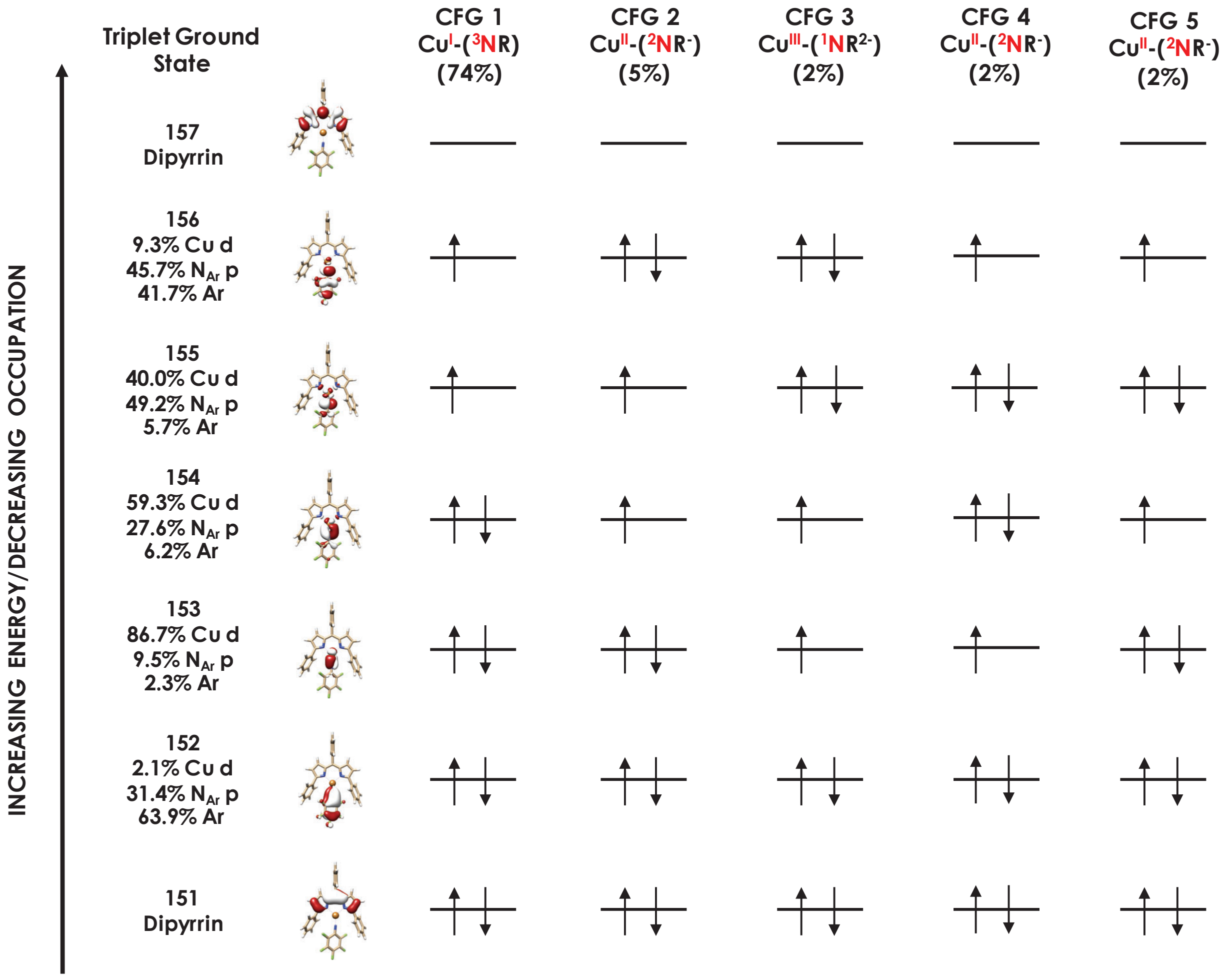

Figure S90. Leading configurations of the triplet ground state of a truncated model derived from the DFT-optimized structure of (^{EMind}L)Cu[N(C_6F_5)] generated through a SORCI procedure employing a CAS(10,7) reference (refWeight 0.8611). Configurations with contributions less than 1% have been omitted.

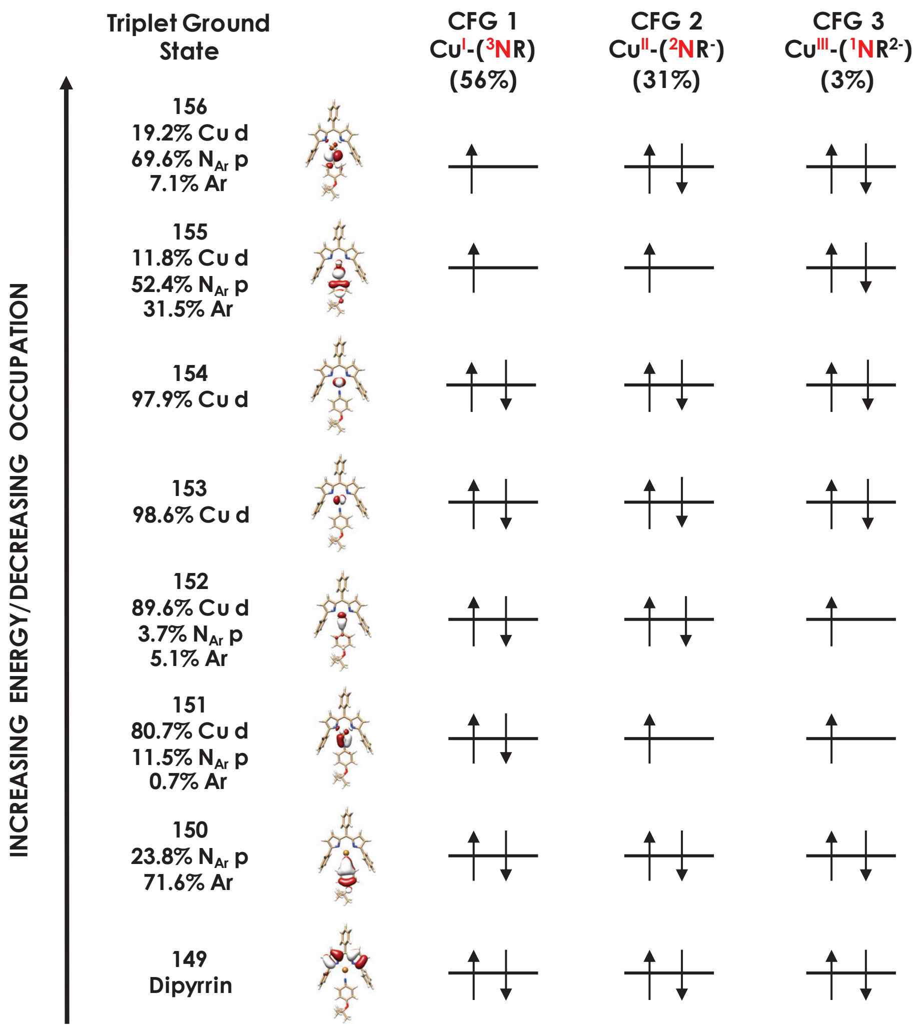

Figure S91. Leading configurations of the triplet ground state of a truncated model derived from the DFT-optimized structure of $(^{EMind}L)Cu[N(C_6H_4O^tBu)]$ generated through a SORCI procedure employing a CAS(14,8) reference (refWeight 0.8920). Configurations with contributions less than 1% have been omitted.

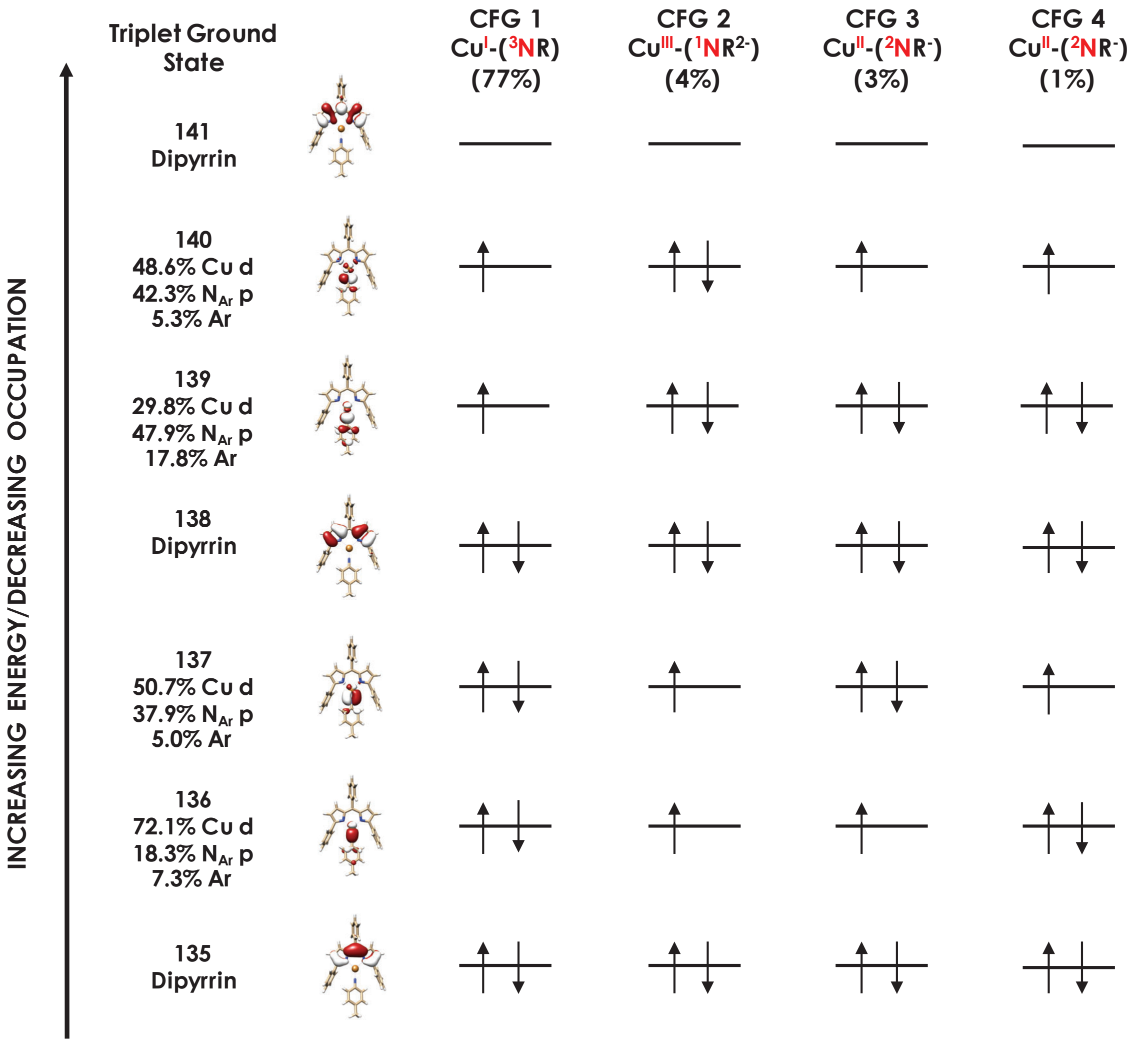

Figure S92. Leading configurations of the triplet ground state of a truncated model derived from the DFT-optimized structure of (^{EMind}L)Cu[N($C_6H_4{}^tBu$)] generated through a SORCI procedure employing a CAS(10,7) reference (refWeight 0.8686). Configurations with contributions less than 1% have been omitted.

Density Functional Theory Calculations.

Calculations were conducted using the Gaussian 16 program[38] to corroborate the kinetic measurements for tetrahydrofuran amination by **4** and elucidate the underlying elementary steps. Hybrid QM/MM calculations (see Figure S93 for an example of the QM/MM partition used) utilized the ONIOM method,[41] with the Universal Force Field (the phenyl groups of the quadraphenyl substituent).[39] The DFT partition utilized the B3LYP functional, and a two-step sequence involving a geometry optimization plus vibrational frequency step using the 6-31+G(d) basis set, followed by larger basis set single point calculations for more accurate energetics utilizing the 6-311++G(d,p) basis set. In general, the results of the two-step scheme mirror the one-step approach (using only the larger basis set) results well, with most free energies only varying by *ca.* 1–2 kcal/mol (Table S13).

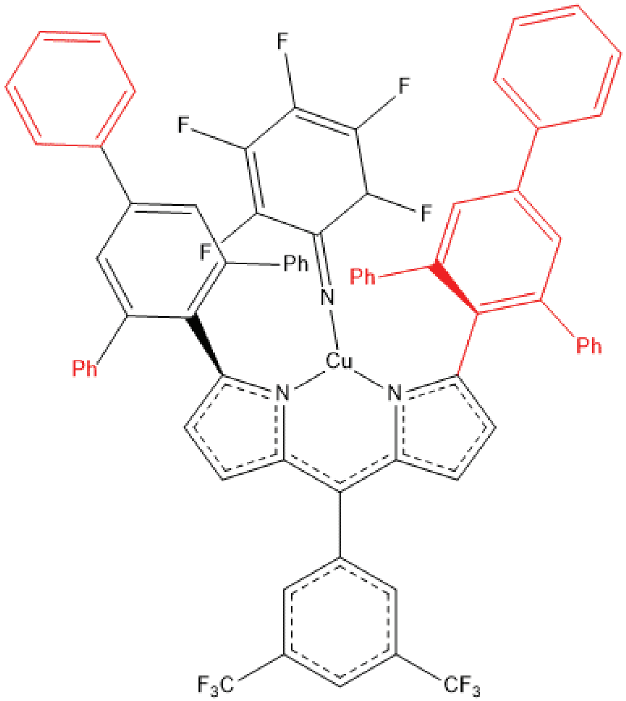

Figure S93. Example of QM/MM partitioning employed in ONIOM(B3LYP/6-31+G(d):UFF) geometry optimization, followed by a single point energy calculation done using ONIOM(B3LYP/6-311++G(d,p):UFF), shown here for ^{3}D (nitrene intermediate with triplet spin). Portions of the molecule treated with QM are given here in black, while portions treated with MM are in red. This partitioning is used throughout the DFT calculations, regardless of stationary point or DFT functional used.

Table S13. Comparison of Relative Gibbs Free Energies Using One- and Two-Step Calculations.

Chemical Species	G-relative (kcal mol^{-1}) 1-Step Result	2-Step Result	Difference (kcal mol^{-1})
k^1-N-int	9.2	10.6	1.4
k^1-N-term	2.8	3.1	0.2
$^1N_2$ elim. TS	24.1	24.6	0.4
$^3N_2$ elim. TS	24.8	25.8	1.0
^{1}[Cu]=Ar	−32.4	−31.9	0.5
^{3}[Cu]=Ar	−35.5	−35.5	0.1
^{1}HAT TS	−2.0	−4.5	−2.5
^{3}HAT TS	−7.3	−7.2	0.1
^{2}[Cu]-NHAr + thf-2-yl	−35.6	−36.6	−1.0
^{2}[Cu]-NHAr + thf-3-yl	−31.6	−32.7	−1.2
amination product	−62.8	−63.6	−0.8

Table S14. CPU Time Comparison for One-Step and Two-Step Computations.

Chemical Species	One-Step CPUs (days)	Two-Step CPUs (days)
k^1-N-int	116	16
k^1-N-term	32	16
$^1N_2$ elim. TS	215	39
^{1}[Cu]=Ar	83	37
^{3}[Cu]=Ar	66	29
^{1}HAT TS	192	143
^{3}HAT TS	219	85

Table S15. Relative Gibbs Free Energies Obtained Using the wB97XD Functional.

Chemical Species	G-relative (kcal mol^{-1}) 2-Step Result
κ^1-N-int	0.6
κ^1-N-term	−0.3
$^1N_2$ elim. TS	–
$^3N_2$ elim. TS	–
^{1}[Cu]=Ar	−26.8
^{3}[Cu]=Ar	−40.6
^{1}HAT TS	−13.9
^{3}HAT TS	−18.4
^{2}[Cu]-NHAr + THF-2-yl	−41.5
^{2}[Cu]-NHAr + THF-3-yl	−37.3
amination product	−71.2

Table S16. Relative Gibbs Free Energies Obtained Using the PCM and B3LYP functional.

Chemical Species	G relative (kcal mol^{-1})
κ^1-N-int	11.0
κ^1-N-term	3.7
$^1N_2$ elim. TS	25.9
$^3N_2$ elim. TS	–
^{1}[Cu]=Ar	−30.5
^{3}[Cu]=Ar	−34.0
^{1}HAT TS	−1.4
^{3}HAT TS	−19.4
^{2}[Cu]-NHAr + THF-2-yl	−49.5
^{2}[Cu]-NHAr + THF-3-yl	−45.7
amination product	−77.3

A: (^{Ar_F}L)Cu

Spin = 0, Charge = 0

Cu	0.544924000	-0.142043000	0.670101000
N	-0.628855000	-1.420787000	-0.221617000
F	-8.195017000	1.234099000	-2.217913000
N	-0.717765000	1.363453000	0.449562000
F	-8.331807000	-1.541699000	1.825702000
C	1.690734000	0.891276000	2.077559000
C	2.439739000	-0.264063000	1.702616000
H	3.166950000	-0.196856000	0.902836000
C	2.423988000	-1.431967000	2.502601000
H	3.023041000	-2.282852000	2.197419000
C	1.664421000	-1.484443000	3.652915000
H	1.651436000	-2.379934000	4.262528000
C	0.899660000	-0.361786000	4.027459000
H	0.304720000	-0.396299000	4.933365000
C	0.908885000	0.791655000	3.269455000
H	0.333779000	1.653493000	3.586443000
C	3.276885000	2.671309000	1.453948000
H	4.020416000	1.985840000	1.846134000
F	-7.160438000	2.898028000	-1.279744000
C	-1.523926000	-3.265584000	-1.218328000
H	-1.574171000	-4.226713000	-1.706767000
C	-0.475247000	2.598705000	0.894883000
F	-6.283639000	1.913422000	-3.005024000
C	-2.714557000	2.356096000	0.929127000
H	-3.773060000	2.511687000	1.070103000
C	-2.682821000	-0.058217000	0.056135000
F	-7.150483000	-3.166757000	0.999534000
C	-0.351022000	-2.596538000	-0.774430000
C	-4.174509000	-0.083325000	-0.010929000
C	-2.571597000	-2.415705000	-0.916141000
H	-3.615761000	-2.573967000	-1.136308000
C	1.947748000	2.240446000	1.459835000
C	0.924543000	3.088481000	0.956545000
C	3.646704000	3.931936000	0.979942000
F	-6.477867000	-2.143664000	2.792674000
C	1.966343000	-2.190529000	-1.655344000

C	2.635461000	4.765811000	0.495156000
H	2.886370000	5.753394000	0.118396000
C	1.049979000	-3.030333000	-0.966670000
C	0.608730000	-5.130765000	0.376069000
C	0.392410000	-6.463928000	-0.009211000
H	0.863319000	-6.859873000	-0.900664000
C	-0.445180000	-7.288309000	0.748520000
H	-0.614448000	-8.312970000	0.443869000
C	-1.064811000	-6.790785000	1.898384000
H	-1.713048000	-7.430303000	2.483350000
C	-0.844115000	-5.469226000	2.295631000
H	-1.319430000	-5.086477000	3.189493000
C	-0.006876000	-4.641953000	1.541787000
H	0.164136000	-3.623200000	1.867388000
C	-2.086272000	1.159998000	0.448480000
C	-2.011320000	-1.244932000	-0.302290000
C	-4.907603000	-0.955335000	0.799415000
H	-4.386629000	-1.610243000	1.486079000
C	3.305710000	-2.588671000	-1.779586000
H	3.999008000	-1.955469000	-2.320852000
C	0.312997000	5.267749000	-0.175518000
C	0.113447000	6.563595000	0.328576000
H	0.665770000	6.905260000	1.195384000
C	-0.811363000	7.419816000	-0.277221000
H	-0.966610000	8.414986000	0.118911000
C	-1.536340000	6.991981000	-1.393001000
H	-2.252371000	7.655749000	-1.859994000
C	-1.334599000	5.708596000	-1.907887000
H	-1.893462000	5.379114000	-2.774211000
C	-0.410045000	4.850284000	-1.306202000
H	-0.259986000	3.860639000	-1.717204000
C	-4.861283000	0.765190000	-0.884609000
H	-4.303374000	1.435001000	-1.526372000
C	-1.705862000	3.257124000	1.208683000
H	-1.817927000	4.258189000	1.598373000
C	5.067294000	4.367095000	0.987602000
C	5.903918000	4.060652000	2.077667000
H	5.520202000	3.521249000	2.934470000
C	7.241120000	4.469184000	2.080681000
H	7.876344000	4.233107000	2.924656000
C	7.757909000	5.184946000	0.998511000
H	8.793454000	5.499736000	1.002715000
C	6.938376000	5.494073000	-0.089277000
H	7.341192000	6.045022000	-0.929353000
C	5.600171000	5.088921000	-0.097322000
H	4.988260000	5.323411000	-0.959244000
C	5.172094000	-4.214948000	-1.392948000
C	5.510875000	-5.544422000	-1.708267000
H	4.739713000	-6.286669000	-1.872419000
C	6.851092000	-5.922124000	-1.835674000
H	7.102974000	-6.945460000	-2.082382000
C	7.865617000	-4.980499000	-1.649007000
H	8.902383000	-5.275191000	-1.747657000
C	7.542164000	-3.658674000	-1.334596000
H	8.329367000	-2.930807000	-1.186091000
C	6.203712000	-3.275110000	-1.206845000

H	5.975771000	-2.249191000	-0.945539000
C	3.750067000	-3.810691000	-1.255260000
C	1.297131000	4.361771000	0.464237000
C	1.549272000	-0.899980000	-2.270753000
C	2.244152000	0.287163000	-1.975050000
H	3.110026000	0.272098000	-1.328723000
C	1.817519000	1.508557000	-2.504545000
H	2.345108000	2.420232000	-2.254274000
C	0.712124000	1.553507000	-3.357050000
H	0.385236000	2.498686000	-3.770482000
C	0.035799000	0.376701000	-3.686494000
H	-0.814292000	0.411400000	-4.355403000
C	0.455745000	-0.846002000	-3.154563000
H	-0.074483000	-1.749267000	-3.428837000
C	-6.300075000	-0.970449000	0.739478000
C	2.837170000	-4.641158000	-0.590034000
H	3.179622000	-5.571155000	-0.151027000
C	-6.253316000	0.734717000	-0.947443000
C	1.498357000	-4.258533000	-0.425684000
C	-6.983331000	-0.129143000	-0.134939000
H	-8.063665000	-0.146845000	-0.182823000
C	-6.973038000	1.688073000	-1.865754000
C	-7.065803000	-1.948753000	1.591824000

B: (^{ArF}L)Cu(κ^1-N$_γ$-C$_6$F$_5$N$_3$)
Spin = 0, Charge = 0

N	-1.471743000	-1.368814000	-0.041953000
F	-6.748221000	0.764958000	-4.004002000
N	-1.797039000	1.443729000	0.582357000
F	-9.434747000	-1.353416000	1.268026000
C	-0.288189000	2.410805000	3.414799000
C	0.395252000	1.331971000	3.994416000
H	1.345475000	1.016054000	3.578141000
C	-0.134841000	0.650483000	5.087240000
H	0.405473000	-0.188420000	5.511874000
C	-1.360272000	1.037970000	5.625191000
H	-1.776448000	0.506881000	6.473685000
C	-2.047538000	2.112115000	5.062434000
H	-2.998004000	2.426639000	5.478864000
C	-1.517233000	2.791680000	3.968668000
H	-2.056039000	3.631719000	3.548322000
C	1.637050000	3.593264000	2.392577000
H	2.143682000	3.379762000	3.327779000
F	-8.744134000	0.216853000	-3.332647000
C	-2.208622000	-3.425077000	-0.698163000
H	-2.181179000	-4.469373000	-0.968463000
C	-1.716233000	2.754350000	0.835661000
F	-7.885903000	2.126088000	-2.751900000
C	-3.888517000	2.356125000	0.437003000
H	-4.955192000	2.452899000	0.307224000
C	-3.605091000	-0.123716000	-0.059959000
F	-8.126439000	-3.078517000	1.090619000
C	-1.107800000	-2.619473000	-0.314665000
C	-5.075967000	-0.238273000	-0.318968000
C	-3.310353000	-2.587952000	-0.654863000

H	-4.330298000	-2.846283000	-0.893415000
C	0.315314000	3.146849000	2.265626000
C	-0.377874000	3.372019000	1.056263000
C	2.294614000	4.263837000	1.359531000
F	-7.733058000	-1.574011000	2.605977000
C	1.184468000	-2.992382000	-1.262149000
C	1.580584000	4.524364000	0.184716000
H	2.055869000	5.063523000	-0.630167000
C	0.295234000	-3.049491000	-0.163752000
C	-0.138310000	-3.604477000	2.262060000
C	-0.478629000	-4.855553000	2.800339000
H	-0.085808000	-5.765086000	2.362470000
C	-1.341924000	-4.937735000	3.897271000
H	-1.607515000	-5.904542000	4.304843000
C	-1.867228000	-3.773129000	4.464187000
H	-2.539338000	-3.838498000	5.309972000
C	-1.522621000	-2.524010000	3.941169000
H	-1.930191000	-1.624075000	4.381527000
C	-0.652620000	-2.436765000	2.850428000
H	-0.379700000	-1.461935000	2.467045000
C	-3.131585000	1.146714000	0.315965000
C	-2.848567000	-1.296179000	-0.237141000
C	-5.905283000	-0.889772000	0.596181000
H	-5.480747000	-1.304686000	1.501748000
C	2.512886000	-3.414109000	-1.099053000
H	3.188679000	-3.376178000	-1.945787000
C	-0.387385850	4.344059390	-1.285246719
C	-0.504342464	5.631467282	-1.976427453
H	-0.432985000	6.460059000	-1.091423000
C	-1.319949000	5.822938000	-2.942607000
H	-1.547732000	6.830256000	-3.266339000
C	-1.660192000	4.735716000	-3.752859000
H	-2.150994000	4.901880000	-4.703053000
C	-1.362669000	3.434428000	-3.338741000
H	-1.621736000	2.593984000	-3.969323000
C	-0.727910000	3.217995000	-2.112372000
H	-0.490357000	2.205553000	-1.809575000
C	-5.635419000	0.300262000	-1.479618000
H	-4.998342000	0.798829000	-2.199248000
C	-2.998287000	3.365109000	0.746828000
H	-3.214646000	4.411544000	0.900749000
C	3.727385000	4.631910000	1.483166000
C	4.230578000	5.161912000	2.685564000
H	3.573399000	5.341085000	3.527284000
C	5.587024000	5.479965000	2.804941000
H	5.966141000	5.890567000	3.731855000
C	6.454165000	5.268267000	1.730376000
H	7.504325000	5.511972000	1.826225000
C	5.966394000	4.740139000	0.532899000
H	6.640294000	4.569598000	-0.296755000
C	4.610439000	4.424748000	0.406546000
H	4.257080000	3.996423000	-0.523234000
C	4.386673000	-4.312666000	0.305059000
C	4.707450000	-5.471538000	1.036579000
H	3.926957000	-6.084136000	1.470303000
C	6.041349000	-5.859919000	1.197217000

H	6.279818000	-6.754561000	1.757794000
C	7.066795000	-5.096766000	0.633452000
H	8.098492000	-5.398352000	0.760172000
C	6.760415000	-3.943958000	-0.093008000
H	7.554850000	-3.349434000	-0.525770000
C	5.428621000	-3.553769000	-0.257771000
H	5.212658000	-2.650040000	-0.807543000
C	2.973546000	-3.889486000	0.138519000
C	0.265115000	4.082156000	0.019798000
C	0.748070000	-2.517915000	-2.596794000
C	0.280329000	-1.204515000	-2.775504000
H	0.245482000	-0.514783000	-1.943971000
C	-0.123198000	-0.765019000	-4.039184000
H	-0.481619000	0.247626000	-4.169740000
C	-0.056293000	-1.629188000	-5.134985000
H	-0.368127000	-1.287255000	-6.113346000
C	0.419348000	-2.932839000	-4.969035000
H	0.474560000	-3.600173000	-5.819324000
C	0.824091000	-3.376158000	-3.706056000
H	1.188015000	-4.389763000	-3.590322000
C	-7.274403000	-0.994377000	0.352992000
C	2.084218000	-3.948652000	1.221170000
H	2.432229000	-4.297697000	2.186550000
C	-7.004111000	0.184756000	-1.719025000
C	0.755729000	-3.524488000	1.085596000
C	-7.833401000	-0.460505000	-0.805177000
H	-8.895179000	-0.546088000	-0.992355000
C	-7.595209000	0.815617000	-2.952955000
C	-8.143484000	-1.742946000	1.329995000
Cu	-0.329573000	0.151078000	0.417417000
N	3.759713000	1.012262000	0.202919000
C	4.698328000	0.426287000	-0.663957000
C	6.046871000	0.472060000	-0.293925000
C	4.389696000	-0.104000000	-1.921237000
C	7.040462000	-0.012039000	-1.134373000
C	5.373627000	-0.592175000	-2.768881000
C	6.706254000	-0.547646000	-2.374087000
F	6.392089000	0.978351000	0.891991000
F	3.111916000	-0.127556000	-2.326725000
F	8.317614000	0.024847000	-0.750608000
F	5.044161000	-1.099038000	-3.958059000
F	7.658717000	-1.020409000	-3.177639000
N	2.607716000	0.558134000	0.243366000
N	1.510251000	0.349257000	0.463017000

C: (^{ArF}L)Cu(κ^1-N_α-$C_6F_5N_3$)
Spin = 0, Charge = 0

N	-0.583780000	-1.572888000	-0.180017000
F	-6.273399000	-4.166352000	-2.871643000
N	-1.675727000	1.295854000	0.070503000
F	-8.407907000	-1.493755000	2.418879000
C	-0.685312000	3.269429000	2.622332000

C	0.391920000	2.794303000	3.372197000
H	1.393132000	2.935015000	2.989585000
C	0.184886000	2.139109000	4.584451000
H	1.029967000	1.764430000	5.148928000
C	-1.113837000	1.962965000	5.062877000
H	-1.281130000	1.452838000	6.004355000
C	-2.191997000	2.444170000	4.324896000
H	-3.202853000	2.319786000	4.697519000
C	-1.977334000	3.089971000	3.114902000
H	-2.825496000	3.468343000	2.556571000
C	0.433398000	5.104043000	1.449766000
H	0.907912000	5.255940000	2.415550000
F	-8.157505000	-3.770794000	-1.826925000
C	-0.951000000	-3.834245000	-0.135423000
H	-0.723197000	-4.888332000	-0.104059000
C	-2.002010000	2.578075000	0.039294000
F	-7.346628000	-2.343332000	-3.273972000
C	-3.940848000	1.465957000	-0.245342000
H	-4.965531000	1.175166000	-0.397224000
C	-2.983709000	-0.829962000	-0.143688000
F	-6.875049000	-2.688317000	3.328144000
C	-0.008398000	-2.776873000	-0.156093000
C	-4.405802000	-1.382551000	-0.173740000
C	-2.184463000	-3.242069000	-0.121816000
H	-3.134442000	-3.750336000	-0.080131000
C	-0.447597000	4.030674000	1.354877000
C	-1.092826000	3.749144000	0.134250000
C	0.692390000	5.958617000	0.382304000
F	-6.532239000	-0.552362000	3.098575000
C	2.173134000	-3.189518000	-1.345144000
C	0.015390000	5.704313000	-0.821866000
H	0.176995000	6.351380000	-1.678074000
C	1.437179000	-3.060420000	-0.147260000
C	1.262285000	-3.343609000	2.364167000
C	1.207621000	-4.479742000	3.195391000
H	1.716861000	-5.392367000	2.911205000
C	0.478401000	-4.455676000	4.387249000
H	0.437535000	-5.336464000	5.014968000
C	-0.204291000	-3.299449000	4.764369000
H	-0.774045000	-3.281476000	5.684541000
C	-0.152838000	-2.165732000	3.952892000
H	-0.685075000	-1.275525000	4.245987000
C	0.580851000	-2.180462000	2.763255000
H	0.612169000	-1.285734000	2.161956000
C	-2.844640000	0.565045000	-0.093823000
C	-1.975097000	-1.820273000	-0.143808000
C	-5.117636000	-1.253978000	1.021870000
H	-4.657677000	-0.763345000	1.869513000
C	3.471897000	-3.720340000	-1.302557000
H	4.004329000	-3.875316000	-2.232861000
C	-1.478629000	4.401823000	-2.310150000
C	-2.186035000	5.446839000	-2.928418000
H	-2.295822000	6.403129000	-2.431663000
C	-2.765675000	5.260659000	-4.187278000
H	-3.311563000	6.069658000	-4.655122000
C	-2.642267000	4.032135000	-4.842283000

H	-3.091923000	3.890815000	-5.816437000
C	-1.936849000	2.987228000	-4.240371000
H	-1.836693000	2.034225000	-4.746573000
C	-1.355141000	3.169594000	-2.984509000
H	-0.809078000	2.346753000	-2.544193000
C	-5.033815000	-2.012828000	-1.257889000
H	-4.504683000	-2.121066000	-2.197868000
C	-3.411160000	2.746455000	-0.178397000
H	-3.922227000	3.693308000	-0.254597000
C	1.654240000	7.079363000	0.549298000
C	1.676145000	7.832828000	1.739792000
H	0.976198000	7.624298000	2.538831000
C	2.592450000	8.876425000	1.902902000
H	2.599677000	9.450743000	2.820416000
C	3.497431000	9.180295000	0.884298000
H	4.207501000	9.986962000	1.013499000
C	3.486399000	8.442279000	-0.300721000
H	4.191795000	8.674880000	-1.088030000
C	2.570942000	7.398595000	-0.470012000
H	2.595427000	6.828591000	-1.388948000
C	5.411797000	-4.723198000	-0.039661000
C	5.703788000	-5.713019000	0.922398000
H	4.949126000	-6.049124000	1.622106000
C	6.968216000	-6.303918000	0.975695000
H	7.179119000	-7.062876000	1.718404000
C	7.956802000	-5.922259000	0.068417000
H	8.936033000	-6.381042000	0.111336000
C	7.679226000	-4.954006000	-0.898846000
H	8.445843000	-4.661705000	-1.604253000
C	6.413939000	-4.361369000	-0.957742000
H	6.227541000	-3.611967000	-1.711941000
C	4.061243000	-4.095287000	-0.082026000
C	-0.863337000	4.618374000	-0.972301000
C	1.578811000	-2.849535000	-2.656889000
C	1.193649000	-1.527423000	-2.934200000
H	1.343530000	-0.750361000	-2.200652000
C	0.617592000	-1.201297000	-4.165321000
H	0.317697000	-0.178891000	-4.370249000
C	0.432061000	-2.193962000	-5.131955000
H	-0.013386000	-1.943059000	-6.085875000
C	0.825305000	-3.510402000	-4.868694000
H	0.682686000	-4.276814000	-5.619435000
C	1.399910000	-3.838949000	-3.635997000
H	1.693505000	-4.863036000	-3.440396000
C	-6.377741000	-1.760987000	1.163365000
C	3.340902000	-3.901386000	1.106159000
H	3.795590000	-4.144122000	2.058694000
C	-6.349785000	-2.537753000	-1.103277000
C	2.030424000	-3.408551000	1.087908000
C	-7.009111000	-2.423602000	0.102595000
H	-8.006255000	-2.821805000	0.213932000
C	-7.031838000	-3.199887000	-2.270223000
C	-7.047415000	-1.635622000	2.502713000
Cu	0.084356000	0.335786000	-0.051530000
N	1.865780000	1.241762000	-0.480633000
C	3.214629000	0.739385000	-0.348139000

C	3.624233000	0.244885000	0.895910000
C	4.174026000	0.699952000	-1.377607000
C	4.908282000	-0.245568000	1.097390000
C	5.476198000	0.264227000	-1.171310000
C	5.845223000	-0.224963000	0.072550000
F	2.784858000	0.254170000	1.915554000
F	3.862584000	1.138166000	-2.608851000
F	5.255565000	-0.718813000	2.294951000
F	6.356101000	0.326210000	-2.171835000
F	7.081876000	-0.663216000	0.287198000
N	1.614535000	2.280390000	-1.122882000
N	1.360847000	3.208790000	-1.697449000

TS1: N_2 Extrusion.
Spin = 0, Charge = 0

N	-0.730639000	-1.483978000	-0.129910000
F	-8.536116000	-1.976581000	-2.351226000
N	-1.662509000	1.294634000	0.337519000
F	-6.003147000	-3.791349000	2.987887000
C	-0.064716000	2.991334000	2.890798000
C	1.078105000	2.389203000	3.434162000
H	2.014635000	2.438958000	2.892928000
C	1.024976000	1.713831000	4.650991000
H	1.920174000	1.245428000	5.044586000
C	-0.173815000	1.636935000	5.356494000
H	-0.217289000	1.113838000	6.305081000
C	-1.318943000	2.229999000	4.827689000
H	-2.256634000	2.180174000	5.369936000
C	-1.265689000	2.899524000	3.607910000
H	-2.159690000	3.371236000	3.220883000
C	1.024444000	4.782528000	1.579254000
H	1.687347000	4.861914000	2.433998000
F	-8.098986000	0.137569000	-2.109818000
C	-1.131611000	-3.706424000	-0.497932000
H	-0.927862000	-4.751790000	-0.672747000
C	-1.908276000	2.592843000	0.563122000
F	-6.833618000	-1.090581000	-3.376710000
C	-3.925780000	1.612252000	0.577955000
H	-4.982808000	1.409531000	0.647426000
C	-3.089676000	-0.719857000	0.092909000
F	-7.354305000	-2.121884000	3.306337000
C	-0.170367000	-2.681323000	-0.331222000
C	-4.513299000	-1.192133000	0.083414000
C	-2.359694000	-3.088295000	-0.381237000
H	-3.330899000	-3.551267000	-0.453336000
C	0.033534000	3.792516000	1.633788000
C	-0.849287000	3.639680000	0.541838000
C	1.148289000	5.650709000	0.493429000
F	-7.978088000	-3.798988000	2.074394000
C	1.981375000	-3.130959000	-1.523663000
C	0.238954000	5.516721000	-0.561482000
H	0.292684000	6.193120000	-1.409831000
C	1.288088000	-2.904220000	-0.310710000
C	1.239595000	-3.002496000	2.222959000
C	1.185070000	-4.120178000	3.074043000

H	1.669689000	-5.047291000	2.793090000
C	0.486059000	-4.054280000	4.283187000
H	0.442268000	-4.920771000	4.930225000
C	-0.165457000	-2.874832000	4.651899000
H	-0.714210000	-2.827505000	5.583558000
C	-0.109066000	-1.756587000	3.817331000
H	-0.620352000	-0.846911000	4.099048000
C	0.601686000	-1.813242000	2.615699000
H	0.647406000	-0.931410000	1.993918000
C	-2.899022000	0.646968000	0.334297000
C	-2.112830000	-1.700447000	-0.138920000
C	-5.037477000	-1.880865000	1.178502000
H	-4.408662000	-2.077741000	2.037843000
C	3.315375000	-3.565763000	-1.487002000
H	3.823415000	-3.775029000	-2.420097000
C	-1.661452000	4.426981000	-1.723560000
C	-2.429193000	5.538561000	-2.110369000
H	-2.363083000	-6.468758000	1.559433000
C	-3.299535000	5.449689000	-3.201178000
H	-3.893003000	6.307390000	-3.490224000
C	-3.406680000	4.254249000	-3.916852000
H	-4.081966000	4.186954000	-4.759964000
C	-2.639913000	3.146744000	-3.546207000
H	-2.719885000	2.222336000	-4.103573000
C	-1.767058000	3.231786000	-2.457996000
H	-1.173216000	2.366993000	-2.191894000
C	-5.329751000	-0.949747000	-1.022690000
H	-4.927687000	-0.424876000	-1.880185000
C	-3.304341000	2.832742000	0.710784000
H	-3.760606000	3.793979000	0.893734000
C	2.206220000	6.693565000	0.463466000
C	2.531531000	7.418616000	1.625549000
H	2.002775000	7.241780000	2.553663000
C	3.529642000	8.397290000	1.593029000
H	3.770684000	8.953248000	2.489881000
C	4.213568000	8.661944000	0.404411000
H	4.986160000	9.419602000	0.381677000
C	3.900586000	7.948619000	-0.754793000
H	4.434162000	8.151680000	-1.674354000
C	2.902746000	6.969574000	-0.728398000
H	2.688659000	6.416159000	-1.634190000
C	5.377549000	-4.292379000	-0.242975000
C	5.793666000	-5.181758000	0.768735000
H	5.102790000	-5.521112000	1.529550000
C	7.104396000	-5.667480000	0.794083000
H	7.412338000	-6.350760000	1.575027000
C	8.015357000	-5.278773000	-0.189306000
H	9.029731000	-5.655606000	-0.167284000
C	7.615795000	-4.408005000	-1.204118000
H	8.322433000	-4.107677000	-1.967006000
C	6.306028000	-3.920868000	-1.235997000
H	6.029832000	-3.238971000	-2.027699000
C	3.980629000	-3.771433000	-0.268240000
C	-0.746309000	4.524670000	-0.557096000
C	1.315443000	-2.983722000	-2.843640000
C	0.672149000	-1.783675000	-3.194284000

H	0.659925000	-0.946606000	-2.511864000
C	0.044006000	-1.654319000	-4.435982000
H	-0.451123000	-0.727804000	-4.696937000
C	0.057008000	-2.718183000	-5.341167000
H	-0.429771000	-2.616681000	-6.302477000
C	0.699961000	-3.912563000	-5.006145000
H	0.709381000	-4.736331000	-5.708094000
C	1.328663000	-4.046239000	-3.764358000
H	1.814075000	-4.982000000	-3.515770000
C	-6.361502000	-2.318746000	1.164239000
C	3.293605000	-3.512766000	0.928429000
H	3.797176000	-3.642461000	1.878672000
C	-6.650664000	-1.396278000	-1.032035000
C	1.953346000	-3.099038000	0.923889000
C	-7.177374000	-2.078808000	0.061616000
H	-8.200143000	-2.430279000	0.048801000
C	-7.527793000	-1.088794000	-2.217864000
C	-6.923448000	-3.012569000	2.378009000
Cu	0.032750000	0.344632000	-0.103511000
N	1.740456000	0.741951000	-0.790731000
C	3.038276000	0.275995000	-0.565841000
C	3.551789000	0.052983000	0.722527000
C	3.903934000	0.005648000	-1.644183000
C	4.677076726	-0.383229323	0.740069763
C	5.203502000	-0.433335000	-1.455281000
C	5.669344000	-0.658752000	-0.161961000
F	2.812236000	0.331674000	1.793660000
F	3.478453000	0.216892000	-2.890967000
F	5.290982000	-0.644834000	2.164804000
F	6.008671000	-0.646720000	-2.497967000
F	6.911579000	-1.092485000	0.031178000
N	1.918346000	2.177321000	-1.472642000
N	1.577771000	2.878639000	-2.269716000

^{3}D: (^{ArF}L)Cu(NC_6F_5)
Spin = 1, Charge = 0

N	1.537080000	-1.370833000	0.230175000
F	8.998195000	1.769169000	1.964754000
N	1.451692000	1.372391000	-0.537812000
F	7.316904000	-0.821921000	-3.400604000
C	-0.159066000	1.880748000	-3.470158000
C	-0.694620000	0.692297000	-3.985334000
H	-1.583495000	0.266963000	-3.538009000
C	-0.086870000	0.033812000	-5.050994000
H	-0.515125000	-0.890008000	-5.423884000
C	1.071344000	0.551696000	-5.626491000
H	1.547408000	0.038000000	-6.454070000
C	1.610962000	1.736897000	-5.130133000
H	2.504730000	2.157019000	-5.578096000
C	1.000695000	2.395524000	-4.065567000
H	1.418896000	3.326992000	-3.706197000
C	-2.221307000	2.898099000	-2.554744000
H	-2.685968000	2.518728000	-3.458409000
F	7.072263000	2.368690000	2.781301000
C	2.564252000	-3.377842000	0.590418000

H	2.679639000	-4.434122000	0.777497000
C	1.188604000	2.622645000	-0.944526000
F	7.886481000	0.409785000	3.243616000
C	3.422887000	2.518983000	-0.745981000
H	4.478682000	2.740774000	-0.760514000
C	3.508662000	0.068209000	-0.094133000
F	9.165034000	-0.402521000	-2.331487000
C	1.345540000	-2.669886000	0.473036000
C	5.004712000	0.121844000	-0.062157000
C	3.559367000	-2.430157000	0.415791000
H	4.625276000	-2.594656000	0.442619000
C	-0.861287000	2.617287000	-2.376166000
C	-0.213021000	3.058387000	-1.199750000
C	-2.955442000	3.628900000	-1.618834000
F	8.130771000	-2.287418000	-2.020484000
C	-0.700649000	-3.217087000	1.807396000
C	-2.280263000	4.125091000	-0.498248000
H	-2.815019000	4.728201000	0.229926000
C	-0.017162000	-3.215648000	0.567678000
C	0.033739000	-3.838049000	-1.890872000
C	0.120436000	-5.115772000	-2.468722000
H	-0.331236000	-5.969824000	-1.978824000
C	0.805057000	-5.301370000	-3.673547000
H	0.873636000	-6.289571000	-4.109607000
C	1.406936000	-4.214109000	-4.311708000
H	1.941561000	-4.360010000	-5.241354000
C	1.316313000	-2.937995000	-3.751371000
H	1.783164000	-2.097234000	-4.246960000
C	0.622485000	-2.745460000	-2.553271000
H	0.544513000	-1.745238000	-2.150800000
C	2.837059000	1.256754000	-0.417187000
C	2.914088000	-1.174497000	0.177027000
C	5.754667000	-0.403951000	-1.116318000
H	5.248132000	-0.842132000	-1.967163000
C	-2.067007000	-3.533043000	1.835637000
H	-2.599522000	-3.505106000	2.779290000
C	-0.292959000	4.372974000	0.962008000
C	-0.327027000	5.749923000	1.241300000
H	-0.796546000	6.438118000	0.549117000
C	0.254535000	6.247217000	2.411790000
H	0.229107000	7.309224000	2.618883000
C	0.867711000	5.374896000	3.315016000
H	1.315905000	5.761120000	4.221267000
C	0.898448000	4.003638000	3.050220000
H	1.366386000	3.326616000	3.753242000
C	0.318859000	3.503592000	1.881135000
H	0.337059000	2.438486000	1.699621000
C	5.667149000	0.699725000	1.022263000
H	5.093326000	1.113910000	1.841840000
C	2.389966000	3.375509000	-1.063487000
H	2.457679000	4.407729000	-1.372084000
C	-4.414939000	3.847035000	-1.794162000
C	-4.954055000	4.114699000	-3.066727000
H	-4.310534000	4.201159000	-3.933018000
C	-6.331516000	4.291841000	-3.228939000
H	-6.737593000	4.500031000	-4.210397000

C	-7.184696000	4.201762000	-2.126885000
H	-8.250890000	4.336482000	-2.255289000
C	-6.661924000	3.937672000	-0.859245000
H	-7.324238000	3.862747000	-0.006475000
C	-5.285361000	3.763305000	-0.690695000
H	-4.905213000	3.539871000	0.297953000
C	-4.225458000	-4.067940000	0.685995000
C	-4.828413000	-5.106548000	-0.047177000
H	-4.224938000	-5.809394000	-0.607678000
C	-6.218905000	-5.254090000	-0.050698000
H	-6.675540000	-6.056755000	-0.615208000
C	-7.020277000	-4.366862000	0.671863000
H	-8.096554000	-4.480599000	0.664186000
C	-6.432843000	-3.332011000	1.402925000
H	-7.054604000	-2.640220000	1.956387000
C	-5.043355000	-3.182697000	1.413757000
H	-4.609857000	-2.359202000	1.968063000
C	-2.754705000	-3.875861000	0.663174000
C	-0.927726000	3.848482000	-0.273548000
C	-0.004004000	-2.967655000	3.096782000
C	0.691212000	-1.769921000	3.336095000
H	0.712072000	-0.987673000	2.593799000
C	1.335503000	-1.555513000	4.557557000
H	1.863598000	-0.627314000	4.733738000
C	1.285692000	-2.530803000	5.555650000
H	1.782086000	-2.362203000	6.502468000
C	0.589158000	-3.721026000	5.333784000
H	0.548361000	-4.475624000	6.108569000
C	-0.054746000	-3.939146000	4.112003000
H	-0.586038000	-4.869720000	3.953803000
C	7.147412000	-0.346965000	-1.083313000
C	-2.039343000	-3.997447000	-0.536634000
H	-2.554413000	-4.286085000	-1.445402000
C	7.060520000	0.749281000	1.049268000
C	-0.677924000	-3.667683000	-0.599303000
C	7.810223000	0.224659000	-0.000092000
H	8.890460000	0.274046000	0.018651000
C	7.755558000	1.328497000	2.254141000
C	7.940183000	-0.957459000	-2.210162000
Cu	0.165665000	0.058509000	0.195125000
N	-1.574827000	0.237646000	0.837580000
C	-2.803756000	0.057745000	0.297920000
C	-2.973377000	-0.678275000	-0.904761000
C	-4.014466000	0.568538000	0.830240000
C	-4.204048000	-0.942232000	-1.474341000
C	-5.255346000	0.312287000	0.272755000
C	-5.364793000	-0.453201000	-0.882617000
F	-1.879873000	-1.133096000	-1.534193000
F	-3.976479000	1.353749000	1.919321000
F	-4.284802000	-1.665193000	-2.598680000
F	-6.360010000	0.815137000	0.840783000
F	-6.562058000	-0.710390000	-1.417383000
H	-1.652590000	0.652409000	2.208438000
O	-1.976248000	2.103153000	3.833127000
C	-1.465114000	0.877944000	3.449464000
C	-2.108348000	-0.204868000	4.308024000

H	-0.373084000	0.925541000	3.452199000
H	-2.251838000	-1.140897000	3.767993000
H	-1.467736000	-0.415814000	5.171284000
C	-3.126926000	1.944273000	4.696347000
H	-2.846664000	2.347449000	5.674387000
H	-3.941807000	2.542953000	4.288119000
C	-3.434402000	0.441995000	4.748636000
H	-3.739134000	0.124347000	5.746720000
H	-4.236179000	0.190563000	4.055044000

^{1}D: (^{ArF}L)Cu(NC$_6$F$_5$)
Spin = 0, Charge = 0

N	1.327408000	1.339097000	-0.333495000
F	8.931456000	-0.258001000	-2.526007000
N	1.423876000	-1.401529000	0.362862000
F	9.014357000	0.748441000	2.277626000
C	-0.205651000	-1.865272000	3.102016000
C	-0.823336000	-0.648672000	3.423306000
H	-1.735818000	-0.363189000	2.913970000
C	-0.264141000	0.212530000	4.365238000
H	-0.752265000	1.155179000	4.586299000
C	0.920647000	-0.132700000	5.011435000
H	1.358601000	0.536740000	5.743168000
C	1.537028000	-1.347140000	4.712033000
H	2.452767000	-1.630829000	5.218916000
C	0.980101000	-2.204745000	3.766235000
H	1.465371000	-3.148088000	3.546711000
C	-2.144715000	-3.197902000	2.313629000
H	-2.639727000	-2.828910000	3.205903000
F	7.926582000	-2.169157000	-2.287982000
C	2.209801000	3.417138000	-0.636829000
H	2.252867000	4.477339000	-0.832634000
C	1.221135000	-2.690302000	0.671595000
F	7.034161000	-0.624655000	-3.526369000
C	3.445383000	-2.461760000	0.453529000
H	4.510503000	-2.632913000	0.434240000
C	3.392413000	0.039459000	-0.000770000
F	7.804313000	2.547988000	2.146280000
C	1.042570000	2.616262000	-0.595871000
C	4.888314000	0.086989000	-0.026236000
C	3.265916000	2.551426000	-0.411202000
H	4.316116000	2.797415000	-0.390949000
C	-0.828606000	-2.777796000	2.095758000
C	-0.147129000	-3.209397000	0.935717000
C	-2.805711000	-4.045612000	1.421841000
F	7.148373000	0.891679000	3.387665000
C	-1.013135000	2.552219000	-2.034548000
C	-2.106655000	-4.493949000	0.295801000
H	-2.592034000	-5.160210000	-0.411450000
C	-0.335147000	3.056171000	-0.895561000
C	-0.371757000	4.424296000	1.231126000
C	-0.217753000	5.799220000	1.471160000
H	-0.543565000	6.523709000	0.734854000
C	0.370779000	6.244262000	2.659093000
H	0.493800000	7.304753000	2.836879000

C	0.801281000	5.321653000	3.616985000
H	1.257025000	5.668227000	4.535431000
C	0.639165000	3.952075000	3.391521000
H	0.967199000	3.238559000	4.136397000
C	0.049877000	3.502914000	2.206481000
H	-0.082761000	2.439011000	2.051436000
C	2.796395000	-1.207831000	0.234037000
C	2.712234000	1.247624000	-0.215513000
C	5.601001000	0.574531000	1.070433000
H	5.067002000	0.910439000	1.950574000
C	-2.339447000	2.940864000	-2.274834000
H	-2.851502000	2.561244000	-3.151156000
C	-0.147149000	-4.517217000	-1.221829000
C	0.019750000	-5.884914000	-1.493948000
H	-0.314236000	-6.629866000	-0.782131000
C	0.632158000	-6.296689000	-2.681708000
H	0.764510000	-7.351665000	-2.883944000
C	1.075335000	-5.347855000	-3.607337000
H	1.550173000	-5.668401000	-4.525566000
C	0.901761000	-3.985518000	-3.349724000
H	1.240662000	-3.252301000	-4.069666000
C	0.287450000	-3.569503000	-2.165093000
H	0.145221000	-2.511071000	-1.985641000
C	5.586607000	-0.353489000	-1.152429000
H	5.040645000	-0.724624000	-2.010742000
C	2.458172000	-3.390933000	0.712286000
H	2.578664000	-4.439956000	0.936144000
C	-4.224960000	-4.422674000	1.642122000
C	-4.689126000	-4.744996000	2.930952000
H	-4.011942000	-4.751965000	3.775824000
C	-6.031305000	-5.079059000	3.136215000
H	-6.379907000	-5.329562000	4.129808000
C	-6.923153000	-5.091072000	2.061233000
H	-7.962117000	-5.347741000	2.222803000
C	-6.474472000	-4.770304000	0.778063000
H	-7.167546000	-4.773888000	-0.053287000
C	-5.133020000	-4.438697000	0.566636000
H	-4.810784000	-4.173955000	-0.432591000
C	-4.421613000	4.193283000	-1.659051000
C	-4.874232000	5.508856000	-1.444133000
H	-4.188044000	6.284866000	-1.129054000
C	-6.216993000	5.838564000	-1.652567000
H	-6.556075000	6.853102000	-1.487520000
C	-7.121649000	4.861747000	-2.074588000
H	-8.161081000	5.118707000	-2.232587000
C	-6.684921000	3.553430000	-2.292622000
H	-7.387446000	2.795620000	-2.614680000
C	-5.343025000	3.219253000	-2.090069000
H	-5.031689000	2.194314000	-2.247609000
C	-3.000853000	3.832096000	-1.417804000
C	-0.796170000	-4.082292000	0.038099000
C	-0.365246000	1.612452000	-2.987484000
C	-0.989899000	0.396163000	-3.315971000
H	-1.948669000	0.137693000	-2.886792000
C	-0.374189000	-0.500924000	-4.193145000
H	-0.857018000	-1.439555000	-4.432613000

C	0.864903000	-0.189539000	-4.757658000
H	1.342042000	-0.886920000	-5.433888000
C	1.483142000	1.026665000	-4.457574000
H	2.437517000	1.272534000	-4.904872000
C	0.868061000	1.929339000	-3.585044000
H	1.351682000	2.875767000	-3.379805000
C	6.994658000	0.617436000	1.038027000
C	-2.314991000	4.345731000	-0.307181000
H	-2.828495000	5.003369000	0.384407000
C	6.979521000	-0.303481000	-1.177816000
C	-0.998821000	3.951889000	-0.025481000
C	7.693183000	0.180973000	-0.084374000
H	8.773882000	0.217716000	-0.106862000
C	7.718260000	-0.831573000	-2.380599000
C	7.742303000	1.194425000	2.212485000
Cu	0.044704000	-0.078514000	0.005142000
N	-1.736222000	-0.075299000	-0.078052000
C	-3.031310000	0.037722000	-0.047258000
C	-3.709587000	1.064057000	0.706320000
C	-3.899085000	-0.864639000	-0.766072000
C	-5.079751000	1.194005000	0.713312000
C	-5.270947000	-0.744711000	-0.738789000
C	-5.866442000	0.287300000	-0.005758000
F	-2.979516000	1.910379000	1.429551000
F	-3.352200000	-1.833886000	-1.489847000
F	-5.678281000	2.164158000	1.411725000
F	-6.051023000	-1.591050000	-1.419314000
F	-7.189848000	0.410678000	0.004720000

[1]TS2: Singlet C–H Activation
Spin = 0, Charge = 0

N	1.571141000	-1.018100000	-0.930563000
F	8.925586000	-2.768970000	0.932111000
N	1.645103000	1.376557000	0.517380000
F	7.900281000	3.101664000	-0.476966000
C	0.271685000	4.126278000	-0.824881000
C	-0.205226000	3.823979000	-2.107654000
H	-1.115563000	3.247348000	-2.217492000
C	0.488807000	4.233687000	-3.243467000
H	0.103293000	3.985147000	-4.226095000
C	1.676577000	4.951040000	-3.118507000
H	2.218724000	5.268339000	-4.002098000
C	2.160452000	5.260453000	-1.848380000
H	3.077718000	5.828571000	-1.739618000
C	1.463656000	4.854125000	-0.712921000
H	1.839408000	5.118470000	0.267745000
C	-1.856345000	4.136631000	0.443462000
H	-2.243516000	4.690271000	-0.405362000
F	7.872104000	-2.194938000	2.742662000
C	2.423627000	-2.716217000	-2.179166000
H	2.454238000	-3.581394000	-2.822847000
C	1.407581000	2.432663000	1.309438000
F	6.943488000	-3.568938000	1.340291000
C	3.610958000	2.025061000	1.479789000

H	4.655132000	2.058715000	1.748715000
C	3.625620000	0.028895000	-0.100540000
F	9.489625000	1.673477000	-0.882174000
C	1.264843000	-2.060266000	-1.701495000
C	5.111189000	-0.079691000	-0.003751000
C	3.493796000	-2.012682000	-1.647535000
H	4.542625000	-2.212742000	-1.801549000
C	-0.516530000	3.736108000	0.381452000
C	0.030083000	2.983136000	1.443973000
C	-2.668713000	3.835658000	1.539344000
F	7.944846000	2.038388000	-2.368517000
C	-0.825838000	-3.246487000	-1.016294000
C	-2.096106000	3.141821000	2.611005000
H	-2.690959000	2.916011000	3.491550000
C	-0.143669000	-2.420007000	-1.938504000
C	-0.159069000	-1.060413000	-4.073935000
C	-0.118814000	-1.506929000	-5.405056000
H	-0.584973000	-2.444564000	-5.682344000
C	0.538221000	-0.752952000	-6.382167000
H	0.572076000	-1.105465000	-7.404896000
C	1.157241000	0.451704000	-6.038577000
H	1.670648000	1.031424000	-6.794686000
C	1.111064000	0.909512000	-4.719554000
H	1.591866000	1.841624000	-4.455386000
C	0.445608000	0.164736000	-3.741730000
H	0.398156000	0.547621000	-2.731228000
C	3.007302000	1.090326000	0.582498000
C	2.955355000	-0.947075000	-0.856688000
C	5.932484000	0.919530000	-0.528649000
H	5.484977000	1.777262000	-1.015799000
C	-2.194974000	-3.490437000	-1.185108000
H	-2.709446000	-4.128360000	-0.476082000
C	-0.257152000	1.900385000	3.705245000
C	-0.188379000	2.460546000	4.990944000
H	-0.488496000	3.487913000	5.157279000
C	0.276187000	1.697772000	6.066852000
H	0.332298000	2.135196000	7.055225000
C	0.665865000	0.370383000	5.868025000
H	1.022879000	-0.219587000	6.702184000
C	0.589147000	-0.197218000	4.593588000
H	0.881674000	-1.227485000	4.443805000
C	0.126366000	0.561914000	3.515042000
H	0.055715000	0.103875000	2.537514000
C	5.701040000	-1.189943000	0.611216000
H	5.072971000	-1.969168000	1.023520000
C	2.608991000	2.855426000	1.940570000
H	2.699817000	3.678978000	2.632367000
C	-4.108121000	4.203340000	1.545950000
C	-4.535703000	5.442395000	1.033241000
H	-3.819128000	6.162293000	0.658434000
C	-5.895019000	5.769871000	1.016292000
H	-6.215527000	6.726049000	0.623232000
C	-6.840381000	4.865630000	1.505823000
H	-7.892236000	5.120044000	1.488887000
C	-6.428303000	3.632877000	2.016538000
H	-7.161778000	2.930297000	2.390615000

C	-5.070459000	3.302240000	2.039995000
H	-4.775550000	2.333856000	2.423661000
C	-4.378255000	-3.083868000	-2.346967000
C	-4.999537000	-3.257262000	-3.598228000
H	-4.411405000	-3.306869000	-4.505803000
C	-6.389095000	-3.385432000	-3.686874000
H	-6.858625000	-3.520002000	-4.652745000
C	-7.172799000	-3.340836000	-2.531679000
H	-8.248452000	-3.436824000	-2.603238000
C	-6.568405000	-3.172481000	-1.284302000
H	-7.176408000	-3.132126000	-0.389752000
C	-5.179807000	-3.047563000	-1.189471000
H	-4.736296000	-2.895587000	-0.213720000
C	-2.906165000	-2.911238000	-2.246238000
C	-0.768845000	2.705428000	2.572162000
C	-0.127254000	-3.888628000	0.120184000
C	0.408863000	-3.117049000	1.164871000
H	0.295981000	-2.041910000	1.168532000
C	1.079844000	-3.735387000	2.223474000
H	1.499531000	-3.137259000	3.020741000
C	1.198634000	-5.126874000	2.260141000
H	1.712641000	-5.604228000	3.084244000
C	0.648530000	-5.902493000	1.236155000
H	0.737373000	-6.980634000	1.267564000
C	-0.013298000	-5.287126000	0.169307000
H	-0.429809000	-5.898340000	-0.621961000
C	7.321019000	0.806184000	-0.444347000
C	-2.212485000	-2.135142000	-3.187878000
H	-2.748633000	-1.678363000	-4.011334000
C	7.086628000	-1.289818000	0.699285000
C	-0.840711000	-1.880473000	-3.044240000
C	7.907604000	-0.293659000	0.171615000
H	8.983773000	-0.378771000	0.233671000
C	7.706224000	-2.457949000	1.422748000
C	8.170231000	1.899439000	-1.040620000
Cu	0.275149000	0.187648000	-0.166737000
N	-1.463211000	0.063747000	0.085634000
C	-2.724234000	0.229609000	-0.234158000
C	-3.181599000	0.702151000	-1.514484000
C	-3.771688000	-0.081418000	0.703865000
C	-4.518000000	0.801497000	-1.838827000
C	-5.110221000	0.056267000	0.398179000
C	-5.481192000	0.486628000	-0.877183000
F	-2.286679000	1.032052000	-2.435596000
F	-3.444344000	-0.505207000	1.914005000
F	-4.914379000	1.210617000	-3.047990000
F	-6.062971000	-0.233173000	1.290169000
F	-6.766520000	0.596400000	-1.180760000
H	-1.697131000	-2.089259000	3.775765000
O	-2.949805000	-3.198135000	5.008667000
C	-1.979890000	-3.133607000	3.959046000
C	-2.645635000	-3.746182000	2.700744000
H	-1.097571000	-3.677739000	4.298726000
H	-2.553500000	-3.063541000	1.855157000
H	-2.185000000	-4.693499000	2.415992000
C	-4.228563000	-3.085250000	4.390159000

H	-4.975573000	-3.435624000	5.103758000
H	-4.441659000	-2.034519000	4.143027000
C	-4.123692000	-3.936937000	3.119725000
H	-4.324771000	-4.983972000	3.357856000
H	-4.829829000	-3.625998000	2.346716000

^{3}TS2: Triplet C–H Activation
Spin = 1, Charge = 0

N	1.537080000	-1.370833000	0.230175000
F	8.998195000	1.769169000	1.964754000
N	1.451692000	1.372391000	-0.537812000
F	7.316904000	-0.821921000	-3.400604000
C	-0.159066000	1.880748000	-3.470158000
C	-0.694620000	0.692297000	-3.985334000
H	-1.583495000	0.266963000	-3.538009000
C	-0.086870000	0.033812000	-5.050994000
H	-0.515125000	-0.890008000	-5.423884000
C	1.071344000	0.551696000	-5.626491000
H	1.547408000	0.038000000	-6.454070000
C	1.610962000	1.736897000	-5.130133000
H	2.504730000	2.157019000	-5.578096000
C	1.000695000	2.395524000	-4.065567000
H	1.418896000	3.326992000	-3.706197000
C	-2.221307000	2.898099000	-2.554744000
H	-2.685968000	2.518728000	-3.458409000
F	7.072263000	2.368690000	2.781301000
C	2.564252000	-3.377842000	0.590418000
H	2.679639000	-4.434122000	0.777497000
C	1.188604000	2.622645000	-0.944526000
F	7.886481000	0.409785000	3.243616000
C	3.422887000	2.518983000	-0.745981000
H	4.478682000	2.740774000	-0.760514000
C	3.508662000	0.068209000	-0.094133000
F	9.165034000	-0.402521000	-2.331487000
C	1.345540000	-2.669886000	0.473036000
C	5.004712000	0.121844000	-0.062157000
C	3.559367000	-2.430157000	0.415791000
H	4.625276000	-2.594656000	0.442619000
C	-0.861287000	2.617287000	-2.376166000
C	-0.213021000	3.058387000	-1.199750000
C	-2.955442000	3.628900000	-1.618834000
F	8.130771000	-2.287418000	-2.020484000
C	-0.700649000	-3.217087000	1.807396000
C	-2.280263000	4.125091000	-0.498248000
H	-2.815019000	4.728201000	0.229926000
C	-0.017162000	-3.215648000	0.567678000
C	0.033739000	-3.838049000	-1.890872000
C	0.120436000	-5.115772000	-2.468722000
H	-0.331236000	-5.969824000	-1.978824000
C	0.805057000	-5.301370000	-3.673547000
H	0.873636000	-6.289571000	-4.109607000
C	1.406936000	-4.214109000	-4.311708000
H	1.941561000	-4.360010000	-5.241354000
C	1.316313000	-2.937995000	-3.751371000

H	1.783164000	-2.097234000	-4.246960000
C	0.622485000	-2.745460000	-2.553271000
H	0.544513000	-1.745238000	-2.150800000
C	2.837059000	1.256754000	-0.417187000
C	2.914088000	-1.174497000	0.177027000
C	5.754667000	-0.403951000	-1.116318000
H	5.248132000	-0.842132000	-1.967163000
C	-2.067007000	-3.533043000	1.835637000
H	-2.599522000	-3.505106000	2.779290000
C	-0.292959000	4.372974000	0.962008000
C	-0.327027000	5.749923000	1.241300000
H	-0.796546000	6.438118000	0.549117000
C	0.254535000	6.247217000	2.411790000
H	0.229107000	7.309224000	2.618883000
C	0.867711000	5.374896000	3.315016000
H	1.315905000	5.761120000	4.221267000
C	0.898448000	4.003638000	3.050220000
H	1.366386000	3.326616000	3.753242000
C	0.318859000	3.503592000	1.881135000
H	0.337059000	2.438486000	1.699621000
C	5.667149000	0.699725000	1.022263000
H	5.093326000	1.113910000	1.841840000
C	2.389966000	3.375509000	-1.063487000
H	2.457679000	4.407729000	-1.372084000
C	-4.414939000	3.847035000	-1.794162000
C	-4.954055000	4.114699000	-3.066727000
H	-4.310534000	4.201159000	-3.933018000
C	-6.331516000	4.291841000	-3.228939000
H	-6.737593000	4.500031000	-4.210397000
C	-7.184696000	4.201762000	-2.126885000
H	-8.250890000	4.336482000	-2.255289000
C	-6.661924000	3.937672000	-0.859245000
H	-7.324238000	3.862747000	-0.006475000
C	-5.285361000	3.763305000	-0.690695000
H	-4.905213000	3.539871000	0.297953000
C	-4.225458000	-4.067940000	0.685995000
C	-4.828413000	-5.106548000	-0.047177000
H	-4.224938000	-5.809394000	-0.607678000
C	-6.218905000	-5.254090000	-0.050698000
H	-6.675540000	-6.056755000	-0.615208000
C	-7.020277000	-4.366862000	0.671863000
H	-8.096554000	-4.480599000	0.664186000
C	-6.432843000	-3.332011000	1.402925000
H	-7.054604000	-2.640220000	1.956387000
C	-5.043355000	-3.182697000	1.413757000
H	-4.609857000	-2.359202000	1.968063000
C	-2.754705000	-3.875861000	0.663174000
C	-0.927726000	3.848482000	-0.273548000
C	-0.004004000	-2.967655000	3.096782000
C	0.691212000	-1.769921000	3.336095000
H	0.712072000	-0.987673000	2.593799000
C	1.335503000	-1.555513000	4.557557000
H	1.863598000	-0.627314000	4.733738000
C	1.285692000	-2.530803000	5.555650000
H	1.782086000	-2.362203000	6.502468000
C	0.589158000	-3.721026000	5.333784000

H	0.548361000	-4.475624000	6.108569000
C	-0.054746000	-3.939146000	4.112003000
H	-0.586038000	-4.869720000	3.953803000
C	7.147412000	-0.346965000	-1.083313000
C	-2.039343000	-3.997447000	-0.536634000
H	-2.554413000	-4.286085000	-1.445402000
C	7.060520000	0.749281000	1.049268000
C	-0.677924000	-3.667683000	-0.599303000
C	7.810223000	0.224659000	-0.000092000
H	8.890460000	0.274046000	0.018651000
C	7.755558000	1.328497000	2.254141000
C	7.940183000	-0.957459000	-2.210162000
Cu	0.165665000	0.058509000	0.195125000
N	-1.574827000	0.237646000	0.837580000
C	-2.803756000	0.057745000	0.297920000
C	-2.973377000	-0.678275000	-0.904761000
C	-4.014466000	0.568538000	0.830240000
C	-4.204048000	-0.942232000	-1.474341000
C	-5.255346000	0.312287000	0.272755000
C	-5.364793000	-0.453201000	-0.882617000
F	-1.879873000	-1.133096000	-1.534193000
F	-3.976479000	1.353749000	1.919321000
F	-4.284802000	-1.665193000	-2.598680000
F	-6.360010000	0.815137000	0.840783000
F	-6.562058000	-0.710390000	-1.417383000
H	-1.652590000	0.652409000	2.208438000
O	-1.976248000	2.103153000	3.833127000
C	-1.465114000	0.877944000	3.449464000
C	-2.108348000	-0.204868000	4.308024000
H	-0.373084000	0.925541000	3.452199000
H	-2.251838000	-1.140897000	3.767993000
H	-1.467736000	-0.415814000	5.171284000
C	-3.126926000	1.944273000	4.696347000
H	-2.846664000	2.347449000	5.674387000
H	-3.941807000	2.542953000	4.288119000
C	-3.434402000	0.441995000	4.748636000
H	-3.739134000	0.124347000	5.746720000
H	-4.236179000	0.190563000	4.055044000

E: (^{ArF}L)Cu(NHC$_6$F$_5$)
Spin = ½, Charge = 0

N	-1.325324000	-1.342081000	-0.320697000
F	-8.928345000	-1.685642000	-1.987556000
N	-1.426055000	1.444665000	0.176332000
F	-9.018596000	1.106872000	2.045509000
C	0.121231000	2.520493000	2.822008000
C	0.640965000	1.379226000	3.446613000
H	1.531693000	0.909491000	3.049040000
C	0.009052000	0.818943000	4.554636000
H	0.420395000	-0.074403000	5.011262000
C	-1.148797000	1.399466000	5.067568000
H	-1.641896000	0.965039000	5.929845000
C	-1.666078000	2.546211000	4.466264000
H	-2.558486000	3.014411000	4.866807000

C	-1.038247000	3.100430000	3.353299000
H	-1.445343000	3.995797000	2.899238000
C	2.157803000	3.475826000	1.765727000
H	2.630746000	3.288956000	2.724134000
F	-7.906684000	-0.213862000	-3.216113000
C	-2.161537000	-3.448406000	-0.074588000
H	-2.182666000	-4.524845000	-0.004276000
C	-1.218716000	2.768990000	0.237037000
F	-7.028521000	-2.178726000	-2.927109000
C	-3.436622000	2.517302000	-0.000373000
H	-4.495544000	2.691140000	-0.110618000
C	-3.394202000	-0.028166000	-0.039791000
F	-7.810328000	-0.232877000	3.255692000
C	-1.019493000	-2.642203000	-0.295748000
C	-4.884963000	-0.091625000	-0.019033000
C	-3.226742000	-2.569480000	0.016154000
H	-4.261370000	-2.818716000	0.191923000
C	0.813167000	3.113315000	1.637994000
C	0.160160000	3.300735000	0.400010000
C	2.872966000	4.039491000	0.706524000
F	-7.155729000	1.810593000	2.922685000
C	1.056145000	-2.893673000	-1.685711000
C	2.196701000	4.272098000	-0.496774000
H	2.721325000	4.731668000	-1.329694000
C	0.370948000	-3.108672000	-0.461462000
C	0.393465000	-3.924173000	1.943328000
C	0.360825000	-5.209637000	2.509729000
H	0.795976000	-6.050954000	1.984297000
C	-0.245163000	-5.418098000	3.752549000
H	-0.272300000	-6.411829000	4.180523000
C	-0.816309000	-4.345674000	4.442795000
H	-1.285151000	-4.509198000	5.404465000
C	-0.779182000	-3.061743000	3.893537000
H	-1.217726000	-2.230613000	4.430716000
C	-0.173542000	-2.848358000	2.652290000
H	-0.146766000	-1.844558000	2.248966000
C	-2.797266000	1.240070000	0.042087000
C	-2.701689000	-1.247536000	-0.128426000
C	-5.604003000	0.376783000	1.084451000
H	-5.072349000	0.785259000	1.934399000
C	2.407819000	-3.257963000	-1.788388000
H	2.927099000	-3.108170000	-2.727384000
C	0.225969000	4.109983000	-1.995046000
C	0.174171000	5.396307000	-2.557399000
H	0.575480000	6.246097000	-2.018864000
C	-0.406218000	5.593590000	-3.814308000
H	-0.448378000	6.587808000	-4.239932000
C	-0.931022000	4.508920000	-4.522333000
H	-1.378734000	4.663505000	-5.495483000
C	-0.873748000	3.224214000	-3.976264000
H	-1.273852000	2.383043000	-4.527851000
C	-0.295480000	3.022735000	-2.720089000
H	-0.249443000	2.018823000	-2.320417000
C	-5.583022000	-0.626589000	-1.106131000
H	-5.035387000	-0.991008000	-1.965924000
C	-2.446374000	3.472784000	0.111665000

H	-2.561182000	4.546005000	0.124103000
C	4.320696000	4.342224000	0.842663000
C	4.828292000	4.900526000	2.030738000
H	4.166629000	5.148975000	2.850979000
C	6.196060000	5.159258000	2.162846000
H	6.578144000	5.591867000	3.078467000
C	7.070480000	4.861019000	1.115277000
H	8.129183000	5.059619000	1.220764000
C	6.578694000	4.305613000	-0.067988000
H	7.257668000	4.069529000	-0.877168000
C	5.211752000	4.048531000	-0.206875000
H	4.855235000	3.600515000	-1.125498000
C	4.536179000	-4.158540000	-0.815360000
C	5.056576000	-5.328517000	-0.229827000
H	4.406409000	-6.028326000	0.279817000
C	6.422572000	-5.615199000	-0.313442000
H	6.813745000	-6.517908000	0.137784000
C	7.283395000	-4.739856000	-0.979156000
H	8.340826000	-4.962403000	-1.040109000
C	6.779425000	-3.577860000	-1.566900000
H	7.447544000	-2.897593000	-2.079123000
C	5.414231000	-3.288615000	-1.490129000
H	5.049843000	-2.373758000	-1.939028000
C	3.089700000	-3.834768000	-0.707446000
C	0.857553000	3.904854000	-0.667269000
C	0.389492000	-2.284786000	-2.871326000
C	1.002848000	-1.223947000	-3.563168000
H	1.976116000	-0.860202000	-3.261910000
C	0.357802000	-0.614054000	-4.642800000
H	0.828321000	0.215213000	-5.155152000
C	-0.892717000	-1.073354000	-5.061315000
H	-1.391485000	-0.599271000	-5.896681000
C	-1.492488000	-2.154216000	-4.411065000
H	-2.453685000	-2.521117000	-4.747080000
C	-0.851992000	-2.764947000	-3.328768000
H	-1.323863000	-3.614400000	-2.852319000
C	-6.996057000	0.306488000	1.097367000
C	2.395495000	-4.072771000	0.487335000
H	2.919967000	-4.490137000	1.338838000
C	-6.975151000	-0.682216000	-1.089266000
C	1.047902000	-3.710712000	0.627020000
C	-7.691653000	-0.219446000	0.011772000
H	-8.772068000	-0.268577000	0.023678000
C	-7.710381000	-1.195245000	-2.300970000
C	-7.746888000	0.752003000	2.326060000
Cu	-0.044452000	0.117545000	-0.255361000
N	1.726416000	0.388047000	-0.794429000
C	2.982566000	0.123075000	-0.344906000
C	3.231278000	-0.574140000	0.853427000
C	4.134880000	0.513660000	-1.063849000
C	4.509444000	-0.868891000	1.294920000
C	5.418796000	0.242250000	-0.630675000
C	5.615894000	-0.458313000	0.557180000
F	2.186111000	-0.971206000	1.599010000
F	3.964324000	1.174596000	-2.225287000
F	4.685839000	-1.547130000	2.434564000

F	6.474460000	0.639474000	-1.349585000
F	6.852597000	-0.738893000	0.979055000
H	1.797137000	0.903039000	-1.667306000

Respective Contributions. K.M.C. and T.A.B. conceived the experimental design, executed syntheses, performed kinetic analysis, and assessed both catalytic and stoichiometric nitrene transfer reactivity. S.N. and T.R.C. conducted computational analysis of nitrene free energy landscape using density functional theory. I.D.M. and K.M.L. conducted multiconfigurational calculations. A.I. and P.V. assisted K.M.C. in substrate synthesis and independent preparation of organic product. S.L.Z. assisted K.M.C. in the refinement of **2**. All authors contributed to the construction of this manuscript.

References.

1. Meyer, E. M.; Gambarotta, S.; Floriani, C.; Chiesi-Villa, A.; Guastini, C., Polynuclear Aryl Derivatives of Group 11 Metals. Synthesis, Solid State-Solution Structural Relationship, and Reactivity with Phosphines. *Organometallics* **1989**, 1067-1079.
2. Yen, S.-P. S., Synthesis of Virtually Monodisperse Linear and Tetrachain Polymers with "Idealized" Structures by Use of a Novel Initiator, α-phenylethylpotassium. *Macromol. Chem. Phys.* **1965**, *81*, 152-160.
3. Carsch, K. M.; DiMucci, I. M.; Iovan, D. A.; Li, A.; Zheng, S.-L.; Titus, C. J.; Lee, S. J.; Irwin, K. D.; Nordlund, D.; Lancaster, K. M.; Betley, T. A., Syntheses of a Copper-Supported Triplet Nitrene Complex Pertinent to Copper-Catalyzed Amination. *Science* **2019**, *365*, 1138-1143.
4. Scriven, E. F. V.; Turnbull, K., Azides: Their Preparation and Synthetic Uses. *Chem. Rev.* **1988**, *88*, 297-368.
5. Politanskaya, L.; Bagryanskaya, I.; Tretyakov, E., Synthesis of Polyfluorinated Arylhydrazines, Arylhydrazones and 3-methyl-1-aryl-1*H*-indazoles. *J. Fluor. Chem.* **2018**, *214*, 48-57.
6. Stoll, S.; Schweiger, A., EasySpin, A Comprehensive Software Package for Spectral Simulation and Analysis in EPR. *J. Magn. Reson.* **2006**, *178*, 42-55.
7. King, E. R.; Hennessy, E. T.; Betley, T. A., Catalytic C−H Bond Amination from High-Spin Iron Imido Complexes. *J. Am. Chem. Soc.* **2011**, *133*, 4917-4923.
8. Liu, J.; Zhang, H.; Yi, H.; Liu, C.; Lei, A., A Facile Access for the C–N Bond Formation by Transition Metal-Free Oxidative Coupling of Benzylic C–H bonds and Amides. *Sci. China Chem.* **2015**, *58*, 1323-1328.
9. Zhang, C.-L.; Gao, Z.-H.; Liang, Z.-Q.; Ye, S., N-Heterocyclic Carbene-Catalyzed Synthesis of Multi-Substituted Benzenes from Enals and α-Cyano-β-methylenones. *Adv. Synth. Catal.* **2016**, *358*, 2862-2866.
10. Li, L.; Xue, M.; Yan, X.; Liu, W.; Xu, K.; Zhang, S., Electrochemical Hofmann rearrangement mediated by NaBr: practical access to bioactive carbamates. *Org. Biomol. Chem.* **2018**, *16*, 4615-4618.
11. Potter, G. T.; Jayson, G. C.; Miller, G. J.; Gardiner, J. M., An Updated Synthesis of the Diazo-Transfer Reagent Imidazole-1-sulfonyl Azide Hydrogen Sulfate. *J. Org. Chem.* **2016**, *81*, 3443-3446.
12. Della, E. W.; Kasum, B.; Kirkbride, K. P., Properties of Bridgehead-Substituted Polycycloalkanes. Synthesis and NMR Analysis of Nitrogen-15-labeled 1-Aminobicycloalkanes and Their Hydrochlorides. *J. Am. Chem. Soc.* **1987**, *109*, 2746-2749.
13. Das, A.; Chen, Y.-S.; Reibenspies, J. H.; Powers, D. C., Characterization of a Reactive Rh_2 Nitrenoid by Crystalline Matrix Isolation. *J. Am. Chem. Soc.* **2019**, *141*, 16232-16236.
14. Sundhoro, M.; Jeon, S.; Park, J.; Ramström, O.; Yan, M., Perfluoroaryl Azide Staudinger Reaction: A Fast and Bioorthogonal Reaction. *Angew. Chem. Int. Ed.* **2017**, *56*, 12117-12121.
15. Peng, Y.; Fan, Y.-H.; Li, S.-Y.; Li, B.; Xue, J.; Deng, Q.-H., Iron-Catalyzed Nitrene Transfer Reaction of 4-Hydroxystilbenes with Aryl Azides: Synthesis of Imines via C═C Bond Cleavage. *Org. Lett.* **2019**, *21*, 8389-8394.
16. Jin, L. M.; Xu, X.; Lu, H.; Cui, X.; Wojtas, L.; Zhang, X. P., Effective Synthesis of Chiral N-Fluoroaryl Aziridines through Enantioselective Aziridination of Alkenes with Fluoroaryl Azides. *Angew. Chem. Int. Ed.* **2013**, *52*, 5309-5313.
17. Hansch, C.; Leo, A.; Taft, R., A Survey of Hammett Substituent Constants and Resonance and Field Parameters. *Chem. Rev.* **1991**, *91*, 165-195.

18. Chan, K.-H.; Guan, X.; Lo, V. K.-Y.; Che, C.-M., Elevated Catalytic Activity of Ruthenium(II)–Porphyrin-Catalyzed Carbene/Nitrene Transfer and Insertion Reactions with N-Heterocyclic Carbene Ligands. *Angew. Chem. Int. Ed.* **2014**, *53*, 2982-2987.
19. Jin, L.-M.; Xu, X.; Lu, H.; Cui, X.; Wojtas, L.; Zhang, X. P., Effective Synthesis of Chiral N-Fluoroaryl Aziridines through Enantioselective Aziridination of Alkenes with Fluoroaryl Azides. *Angew. Chem. Int. Ed.* **2013**, *52*, 5309-5313.
20. Du, Y.-D.; Xu, Z.-J.; Zhou, C.-Y.; Che, C.-M., An Effective [$Fe^{III}(TF_4DMAP)Cl$] Catalyst for C–H Bond Amination with Aryl and Alkyl Azides. *Org. Lett.* **2019**, *21*, 895-899.
21. Luo, Y.-R., *Handbook of Bond Dissociation Energies in Organic Compounds*. CRC press: 2002.
22. Luo, Y.-R.; Kerr, J., Bond Dissociation Energies. *CRC Handb. Chem. Phys.* **2012**, *89*, 89.
23. Xue, X.-S.; Ji, P.; Zhou, B.; Cheng, J.-P., The Essential Role of Bond Energetics in C–H Activation/Functionalization. *Chem. Rev.* **2017**, *117*, 8622-8648.
24. Mazzarella, D.; Crisenza, G. E. M.; Melchiorre, P., Asymmetric Photocatalytic C–H Functionalization of Toluene and Derivatives. *J. Am. Chem. Soc.* **2018**, *140*, 8439-8443.
25. Albone, D. P.; Challenger, S.; Derrick, A. M.; Fillery, S. M.; Irwin, J. L.; Parsons, C. M.; Takada, H.; Taylor, P. C.; Wilson, D. J., Amination of Ethers using Chloramine-T Hydrate and a Copper(I) Catalyst. *Org. Biomol. Chem.* **2005**, *3*, 107-111.
26. Fier, P. S.; Luo, J.; Hartwig, J. F., Copper-Mediated Fluorination of Arylboronate Esters. Identification of a Copper(III) Fluoride Complex. *J. Am. Chem. Soc.* **2013**, *135*, 2552-2559.
27. Kohler, D. G.; Gockel, S. N.; Kennemur, J. L.; Waller, P. J.; Hull, K. L., Palladium-Catalysed Anti-Markovnikov Selective Oxidative Amination. *Nat. Chem.* **2018**, *10*, 333.
28. Chen, Y.; Liu, Y.; Zhang, X.; Lu, D.; Yang, L.; Deng, J.; Deng, S., Copper Catalyzed Direct Synthesis of Nitriles from Acyl Azides. *ChemistrySelect* **2018**, *3*, 12325-12329.
29. Dočekal, V.; Šimek, M.; Dračínský, M.; Veselý, J., Decarboxylative Organocatalytic Allylic Amination of Morita–Baylis–Hillman Carbamates. *Chem. Eur. J.* **2018,** *24*, 13441-13445.
30. Bruker AXS APEX3, Bruker AXS, Madison, Wisconsin, **2015**.
31. Krause, L.; Herbst-Irmer, R.; Sheldrick, G. M.; Stalke, D., Comparison of Silver and Molybdenum Microfocus X-ray Sources for Single-Crystal Structure Determination. *J. Appl. Crystallogr.* **2015**, *48*, 3-10.
32. Sheldrick, G., SHELXT - Integrated Space-Group and Crystal-Structure Determination. *Acta Cryst. Sect. A* **2015**, *71*, 3-8.
33. Sheldrick, G., Crystal Structure Refinement with SHELXL. *Acta Crystallogr. A* **2015**, *71*, 3-8.
34. Dolomanov, O. V.; Bourhis, L. J.; Gildea, R. J.; Howard, J. A. K.; Puschmann, H., OLEX2: A Complete Structure Solution, Refinement and Analysis Program. *J. Appl. Crystallogr.* **2009**, *42*, 339-341.
35. Spek, A., Single-Crystal Structure Validation with the Program PLATON. *J. Appl. Crystallogr.* **2003**, *36*, 7-13.
36. Ramadhar, T. R.; Zheng, S.-L.; Chen, Y.-S.; Clardy, J., Analysis of Rapidly Synthesized Guest-Filled Porous Complexes With Synchrotron Radiation: Practical Guidelines for the Crystalline Sponge Method. *Acta. Cryst. Sect. A.* **2015**, *71*, 46-58.
37. Persistence Of Vision Raytracer. http://www.povray.org/.
38. Gaussian 16, Revision C.01, Frisch, M. J.; Trucks, G. W.; Schlegel, H. B.; Scuseria, G. E.; Robb, M. A.; Cheeseman, J. R.; Scalmani, G.; Barone, V.; Petersson, G. A.; Nakatsuji, H.; Li, X.; Caricato, M.; Marenich, A. V.; Bloino, J.; Janesko, B. G.; Gomperts, R.; Mennucci, B.; Hratchian,

H. P.; Ortiz, J. V.; Izmaylov, A. F.; Sonnenberg, J. L.; Williams-Young, D.; Ding, F.; Lipparini, F.; Egidi, F.; Goings, J.; Peng, B.; Petrone, A.; Henderson, T.; Ranasinghe, D.; Zakrzewski, V. G.; Gao, J.; Rega, N.; Zheng, G.; Liang, W.; Hada, M.; Ehara, M.; Toyota, K.; Fukuda, R.; Hasegawa, J.; Ishida, M.; Nakajima, T.; Honda, Y.; Kitao, O.; Nakai, H.; Vreven, T.; Throssell, K.; Montgomery, J. A., Jr.; Peralta, J. E.; Ogliaro, F.; Bearpark, M. J.; Heyd, J. J.; Brothers, E. N.; Kudin, K. N.; Staroverov, V. N.; Keith, T. A.; Kobayashi, R.; Normand, J.; Raghavachari, K.; Rendell, A. P.; Burant, J. C.; Iyengar, S. S.; Tomasi, J.; Cossi, M.; Millam, J. M.; Klene, M.; Adamo, C.; Cammi, R.; Ochterski, J. W.; Martin, R. L.; Morokuma, K.; Farkas, O.; Foresman, J. B.; Fox, D. J. Gaussian, Inc., Wallingford CT, **2016**.

39. Rappe, A. K.; Casewit, C. J.; Colwell, K. S.; Goddard, W. A.; Skiff, W. M., UFF, a Full Periodic Table Force Field for Molecular Mechanics and Molecular Dynamics Simulations. *J. Am. Chem. Soc.* **1992**, *114*, 10024-10035.

checkCIF/PLATON report

Structure factors have been supplied for datablock(s) 2, 4

THIS REPORT IS FOR GUIDANCE ONLY. IF USED AS PART OF A REVIEW PROCEDURE FOR PUBLICATION, IT SHOULD NOT REPLACE THE EXPERTISE OF AN EXPERIENCED CRYSTALLOGRAPHIC REFEREE.

No syntax errors found. CIF dictionary Interpreting this report

Datablock: 2

Bond precision: C-C = 0.0089 A Wavelength=0.71073

Cell:	a=10.531(3)	b=28.389(9)	c=26.123(13)
	alpha=90	beta=98.954(17)	gamma=90
Temperature:	100 K		

	Calculated	Reported
Volume	7715(5)	7715(5)
Space group	P 21/c	P 21/c
Hall group	-P 2ybc	-P 2ybc
Moiety formula	C75 H96 Cu F6 N5, 2(C4 H10 O)	?
Sum formula	C83 H116 Cu F6 N5 O2	C83 H116 Cu F6 N5 O2
Mr	1393.36	1393.34
Dx,g cm-3	1.200	1.200
Z	4	4
Mu (mm-1)	0.346	0.346
F000	2992.0	2992.0
F000'	2994.52	
h,k,lmax	12,33,31	0,0,0
Nref	13849	13598
Tmin,Tmax	0.932,0.953	0.552,0.801
Tmin'	0.824	

Correction method= # Reported T Limits: Tmin=0.552 Tmax=0.801
AbsCorr = MULTI-SCAN

Data completeness= 0.982 Theta(max)= 25.160

R(reflections)= 0.1095(9453) wR2(reflections)= 0.2643(13598)

S = 1.085 Npar= 1125

The following ALERTS were generated. Each ALERT has the format
test-name_ALERT_alert-type_alert-level.
Click on the hyperlinks for more details of the test.

Alert level C

PLAT082_ALERT_2_C	High R1 Value	0.11	Report
PLAT084_ALERT_3_C	High wR2 Value (i.e. > 0.25)	0.26	Report
PLAT213_ALERT_2_C	Atom C27 has ADP max/min Ratio	3.1	prolat
PLAT220_ALERT_2_C	NonSolvent Resd 1 C Ueq(max)/Ueq(min) Range	4.6	Ratio
PLAT260_ALERT_2_C	Large Average Ueq of Residue Including O1T	0.103	Check
PLAT260_ALERT_2_C	Large Average Ueq of Residue Including O2S	0.133	Check
PLAT260_ALERT_2_C	Large Average Ueq of Residue Including O1S	0.106	Check
PLAT260_ALERT_2_C	Large Average Ueq of Residue Including O2T	0.140	Check
PLAT341_ALERT_3_C	Low Bond Precision on C-C Bonds	0.0089	Ang.
PLAT906_ALERT_3_C	Large K Value in the Analysis of Variance	18.216	Check
PLAT906_ALERT_3_C	Large K Value in the Analysis of Variance	3.477	Check
PLAT910_ALERT_3_C	Missing # of FCF Reflection(s) Below Theta(Min).	9	Note
PLAT911_ALERT_3_C	Missing FCF Refl Between Thmin & STh/L= 0.598	247	Report
PLAT921_ALERT_1_C	R1 in the CIF and FCF Differ by	0.0013	Check
PLAT922_ALERT_1_C	wR2 in the CIF and FCF Differ by	0.0018	Check
PLAT977_ALERT_2_C	Check Negative Difference Density on H1SC	-0.33	eA-3

Alert level G

PLAT002_ALERT_2_G	Number of Distance or Angle Restraints on AtSite	72	Note
PLAT003_ALERT_2_G	Number of Uiso or Uij Restrained non-H Atoms ...	48	Report
PLAT083_ALERT_2_G	SHELXL Second Parameter in WGHT Unusually Large	70.17	Why ?
PLAT171_ALERT_4_G	The CIF-Embedded .res File Contains EADP Records	13	Report
PLAT175_ALERT_4_G	The CIF-Embedded .res File Contains SAME Records	5	Report
PLAT176_ALERT_4_G	The CIF-Embedded .res File Contains SADI Records	9	Report
PLAT178_ALERT_4_G	The CIF-Embedded .res File Contains SIMU Records	3	Report
PLAT187_ALERT_4_G	The CIF-Embedded .res File Contains RIGU Records	6	Report
PLAT301_ALERT_3_G	Main Residue Disorder ....(Resd 1)	31%	Note
PLAT302_ALERT_4_G	Anion/Solvent/Minor-Residue Disorder (Resd 2)	100%	Note
PLAT302_ALERT_4_G	Anion/Solvent/Minor-Residue Disorder (Resd 3)	100%	Note
PLAT302_ALERT_4_G	Anion/Solvent/Minor-Residue Disorder (Resd 4)	100%	Note
PLAT302_ALERT_4_G	Anion/Solvent/Minor-Residue Disorder (Resd 5)	100%	Note
PLAT304_ALERT_4_G	Non-Integer Number of Atoms in ..... (Resd 2)	8.45	Check
PLAT304_ALERT_4_G	Non-Integer Number of Atoms in ..... (Resd 3)	11.12	Check
PLAT304_ALERT_4_G	Non-Integer Number of Atoms in ..... (Resd 4)	6.55	Check
PLAT304_ALERT_4_G	Non-Integer Number of Atoms in ..... (Resd 5)	3.89	Check
PLAT413_ALERT_2_G	Short Inter XH3 .. XHn H63C ..H3SB .	1.97	Ang.
	1+x,y,z =	1_655	Check
PLAT720_ALERT_4_G	Number of Unusual/Non-Standard Labels	40	Note
PLAT811_ALERT_5_G	No ADDSYM Analysis: Too Many Excluded Atoms	!	Info
PLAT860_ALERT_3_G	Number of Least-Squares Restraints	1189	Note
PLAT883_ALERT_1_G	No Info/Value for _atom_sites_solution_primary .	Please	Do !
PLAT909_ALERT_3_G	Percentage of I>2sig(I) Data at Theta(Max) Still	41%	Note
PLAT933_ALERT_2_G	Number of OMIT Records in Embedded .res File ...	9	Note
PLAT941_ALERT_3_G	Average HKL Measurement Multiplicity	1.0	Low
PLAT961_ALERT_5_G	Dataset Contains no Negative Intensities	Please	Check
PLAT978_ALERT_2_G	Number C-C Bonds with Positive Residual Density.	0	Info

---

0 **ALERT level A** = Most likely a serious problem - resolve or explain
0 **ALERT level B** = A potentially serious problem, consider carefully
16 **ALERT level C** = Check. Ensure it is not caused by an omission or oversight
27 **ALERT level G** = General information/check it is not something unexpected

3 ALERT type 1 CIF construction/syntax error, inconsistent or missing data

```
 14 ALERT type 2 Indicator that the structure model may be wrong or deficient
 10 ALERT type 3 Indicator that the structure quality may be low
 14 ALERT type 4 Improvement, methodology, query or suggestion
  2 ALERT type 5 Informative message, check
```

Datablock: 4

```
Bond precision:   C-C = 0.0033 A          Wavelength=0.71073

Cell:             a=14.053(4)     b=14.168(3)        c=25.657(5)
                  alpha=90        beta=94.936(8)     gamma=90
Temperature:      100 K
```

	Calculated	Reported
Volume	5089(2)	5090(2)
Space group	P 21/c	P 21/c
Hall group	-P 2ybc	-P 2ybc
Moiety formula	C65 H41 Cu F6 N2 [+ solvent]	?
Sum formula	C65 H41 Cu F6 N2 [+ solvent]	C65 H41 Cu F6 N2
Mr	1027.55	1027.54
Dx,g cm-3	1.341	1.341
Z	4	4
Mu (mm-1)	0.495	0.495
F000	2112.0	2112.0
F000'	2114.27	
h,k,lmax	16,16,30	16,16,29
Nref	9029	8825
Tmin,Tmax	0.931,0.952	0.931,0.952
Tmin'	0.820	

```
Correction method= # Reported T Limits: Tmin=0.931 Tmax=0.952
AbsCorr = MULTI-SCAN

Data completeness= 0.977          Theta(max)= 25.068

R(reflections)= 0.0454( 5761)     wR2(reflections)= 0.1109( 8825)

S = 1.031                  Npar= 609
```

The following ALERTS were generated. Each ALERT has the format
test-name_ALERT_alert-type_alert-level.
Click on the hyperlinks for more details of the test.

Alert level C

PLAT029_ALERT_3_C _diffrn_measured_fraction_theta_full value Low .   0.977 Why?

```
PLAT213_ALERT_2_C Atom F3              has ADP max/min Ratio .....        3.4 prolat
PLAT213_ALERT_2_C Atom F3'             has ADP max/min Ratio .....        3.4 prolat
PLAT369_ALERT_2_C Long   C(sp2)-C(sp2) Bond  C23       - C36      .      1.55 Ang.
PLAT410_ALERT_2_C Short Intra H...H Contact  H37       ..H65      .      1.95 Ang.
                                               x,y,z  =      1_555 Check
PLAT905_ALERT_3_C Negative K value in the Analysis of Variance ...     -0.050 Report
PLAT911_ALERT_3_C Missing FCF Refl Between Thmin & STh/L=    0.596        184 Report
```

Alert level G

```
PLAT066_ALERT_1_G Predicted and Reported Tmin&Tmax Range Identical          ? Check
PLAT171_ALERT_4_G The CIF-Embedded .res File Contains EADP Records          4 Report
PLAT242_ALERT_2_G Low    'MainMol' Ueq as Compared to Neighbors of        C17 Check
PLAT301_ALERT_3_G Main Residue  Disorder .............(Resd  1  )          5% Note
PLAT605_ALERT_4_G Largest Solvent Accessible VOID in the Structure        181 A**3
PLAT773_ALERT_2_G Check long C-C Bond in CIF: C12      --C16'            1.75 Ang.
PLAT802_ALERT_4_G CIF Input Record(s) with more than 80 Characters          1 Info
PLAT883_ALERT_1_G No Info/Value for _atom_sites_solution_primary .     Please Do !
PLAT909_ALERT_3_G Percentage of I>2sig(I) Data at Theta(Max) Still        40% Note
PLAT910_ALERT_3_G Missing # of FCF Reflection(s) Below Theta(Min).          1 Note
PLAT941_ALERT_3_G Average HKL Measurement Multiplicity ...........        2.4 Low
PLAT978_ALERT_2_G Number C-C Bonds with Positive Residual Density.          4 Info
```

```
   0 ALERT level A = Most likely a serious problem - resolve or explain
   0 ALERT level B = A potentially serious problem, consider carefully
   7 ALERT level C = Check. Ensure it is not caused by an omission or oversight
  12 ALERT level G = General information/check it is not something unexpected

   2 ALERT type 1 CIF construction/syntax error, inconsistent or missing data
   7 ALERT type 2 Indicator that the structure model may be wrong or deficient
   7 ALERT type 3 Indicator that the structure quality may be low
   3 ALERT type 4 Improvement, methodology, query or suggestion
   0 ALERT type 5 Informative message, check
```

It is advisable to attempt to resolve as many as possible of the alerts in all categories. Often the minor alerts point to easily fixed oversights, errors and omissions in your CIF or refinement strategy, so attention to these fine details can be worthwhile. In order to resolve some of the more serious problems it may be necessary to carry out additional measurements or structure refinements. However, the purpose of your study may justify the reported deviations and the more serious of these should normally be commented upon in the discussion or experimental section of a paper or in the "special_details" fields of the CIF. checkCIF was carefully designed to identify outliers and unusual parameters, but every test has its limitations and alerts that are not important in a particular case may appear. Conversely, the absence of alerts does not guarantee there are no aspects of the results needing attention. It is up to the individual to critically assess their own results and, if necessary, seek expert advice.

Publication of your CIF in IUCr journals

A basic structural check has been run on your CIF. These basic checks will be run on all CIFs submitted for publication in IUCr journals (*Acta Crystallographica*, *Journal of Applied Crystallography*, *Journal of Synchrotron Radiation*); however, if you intend to submit to *Acta Crystallographica Section C* or *E* or *IUCrData*, you should make sure that full publication checks are run on the final version of your CIF prior to submission.

Publication of your CIF in other journals

Please refer to the *Notes for Authors* of the relevant journal for any special instructions relating to CIF submission.

PLATON version of 22/03/2021; check.def file version of 19/03/2021

Datablock 2 - ellipsoid plot

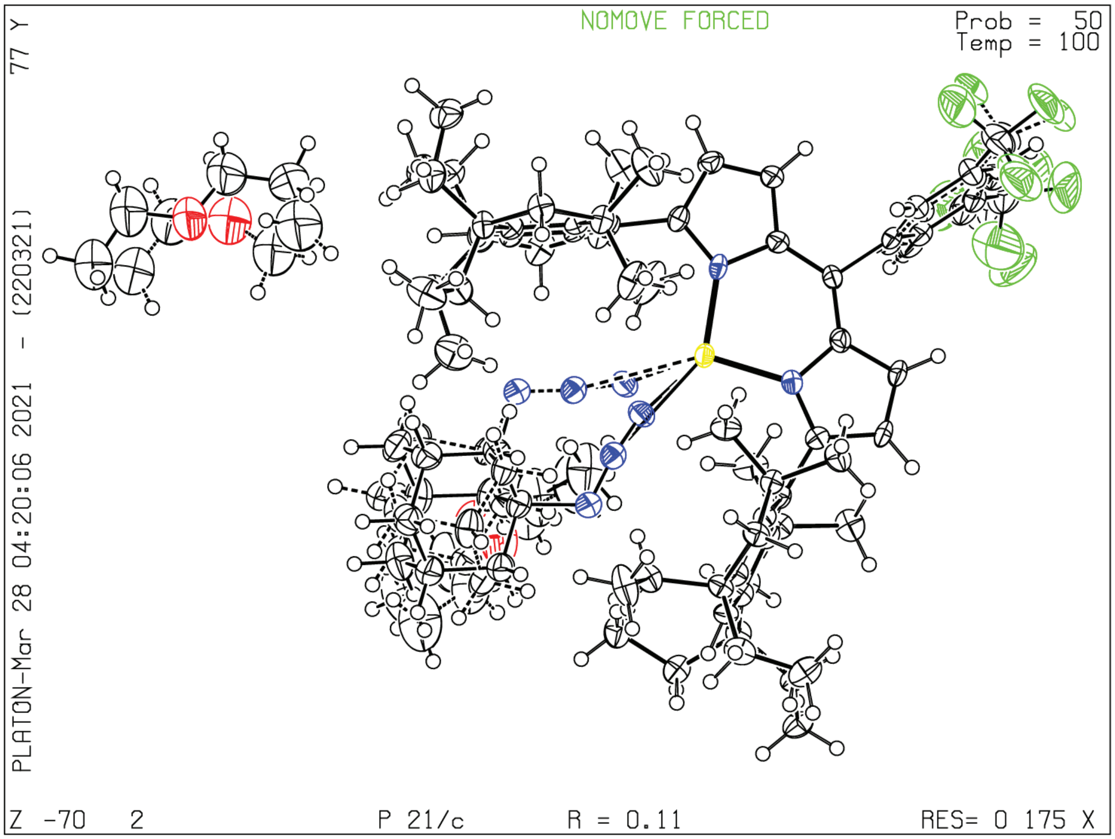

Datablock 4 - ellipsoid plot

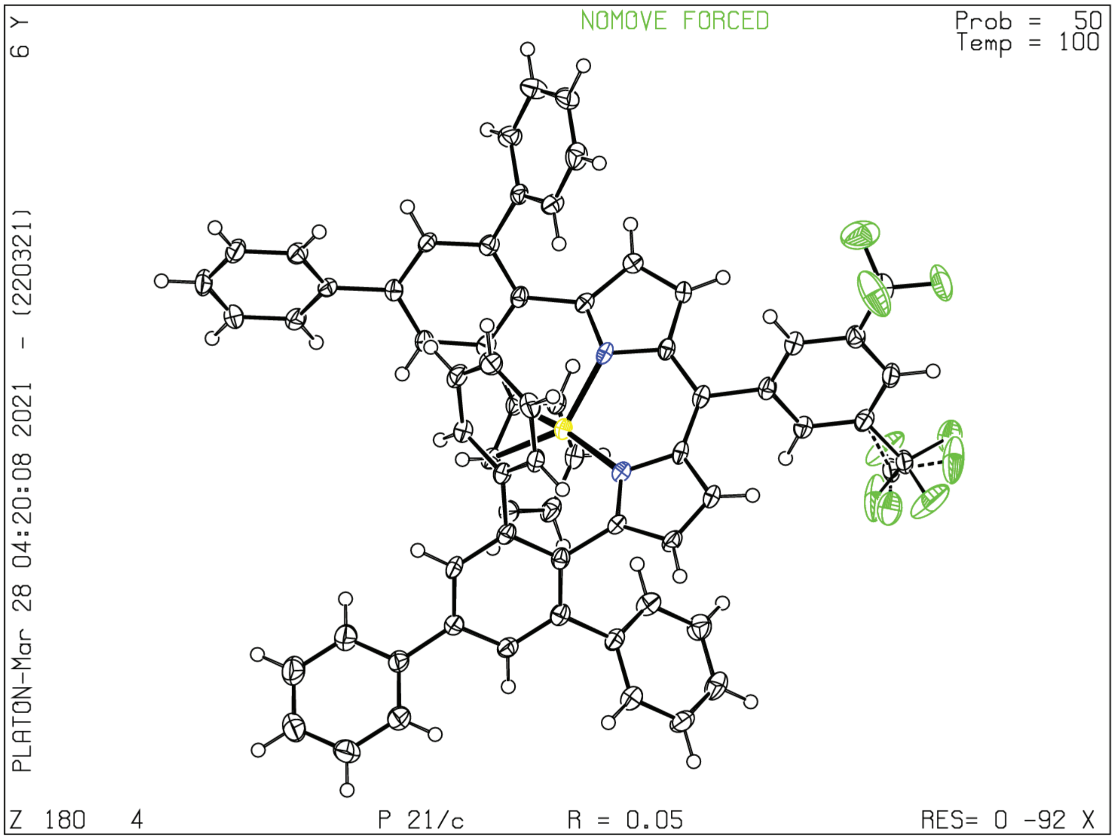

Supplement: SC-014-D3SC03641C-s001 [file SC-014-D3SC03641C-s001.pdf]
